# Structure Elucidation and Total Synthesis of Granolides A–C: Ethyl‐branched Sesquiterpenes From the Tropical Frog *Gephyromantis granulatus*

**DOI:** 10.1002/chem.71139

**Published:** 2026-05-28

**Authors:** Johanna Kuhn, Miguel Vences, Stefan Schulz

**Affiliations:** ^1^ Technische Universität Braunschweig Institute of Organic Chemistry Braunschweig Germany; ^2^ Technische Universität Braunschweig Institute of Zoology Braunschweig Germany

**Keywords:** gas chromatography, mass spectrometry, natural products, structure elucidation, total synthesis

## Abstract

Malagasy frogs of the endemic family *Mantellinae*, among them *Gephyromantis granulatus*, have a unique feature: femoral glands that secrete volatile semiochemicals used for communication. Here, we report the structure elucidation, total synthesis, and determination of the absolute configuration of the main component of the femoral gland secretion of *G. granulatus*: granolide C. The structures of two further macrolides, granolides A and B, were also clarified. Structure elucidation was performed using GC/MS, as NMR analysis was not possible due to the limited amount of available material. Various stereoisomers were synthesized using a flexible synthetic approach that enabled access to different substitution patterns found in granolides. Granolides A–C are unique sesquiterpene macrocyclic lactones with ethyl branches instead of the common methyl branches – a feature that is rarely found in nature.

## Graphical abstract

Males of the tropical frog *Gephyromantis granulatus* carry unique ethyl‐branched sesquiterpenoid macrolactones in their scent glands. Their structure elucidation was performed by GC/MS, leading to the proposed structures of the granolides. A flexible enantioselective synthesis confirmed the structures and allowed determination of their absolute configuration. Ethyl‐branched sesquiterpenes are rare in nature.

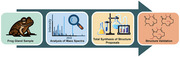

## 1 Introduction

The tropical island of Madagascar is a biodiversity hotspot and home to a wealth of diverse frog species [1]. The Mantellidae family is the largest in Madagascar, with 286 known species, and is endemic to Madagascar and the Comoros island of Mayotte [2, 3]. Among mantellids, the subfamily Mantellinae is characterized by a distinctive feature: femoral glands, which are located on the underside of the frogs' thighs and are clearly distinguishable from the surrounding skin (Figure S1B). They have exclusively been observed in this subfamily, primarily in male frogs [4, 5]. The glands presumably secrete pheromones during mating, which may stimulate the female to lay eggs [6]. Interestingly, the mantellines exhibit a reproductive behavior different from that of most anurans, which may be related to the presence of these femoral glands. During reproduction, the male positions himself above the female, rather than clasping her (amplexus) as occurs in most anurans (Figure S1A) [7].

**FIGURE 1:**
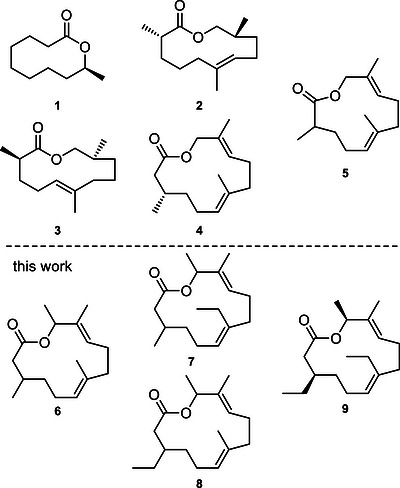
Macrolides found as gland constituents of different frog species of the families Hyperoliidae and Mantellidae: phoracantholide J (**1**), gephyromantolide A (**2**), cinnamomeoventrolide (**3**), frogolide (**4**), and gephyromantolide B (**5**). In this work, the novel ethyl‐branched macrolactones granolide A (**6**), granolide B (**7**), granolide D (**8**), and granolide C (**9**) are described.

Our group has already studied gland secretions from various mantellines and frequently found complex and species‐specific mixtures, often including macrocyclic lactones [8]. Phoracantholide J (**1**) was found in the femoral glands of *Mantidactylus katae* (previously known as *M. multiplicatus*) and was experimentally proven to stimulate activity in these frogs, thus likely having a pheromone function (Figure 1) [9]. It has also been demonstrated that **1**, which also occurs in other mantellines, is perceived by the olfactory organ of *Mantidactylus betsileanus*. Mantelline olfactory organs show a morphology different from that of other amphibians, possibly related to their derived reproductive behavior and femoral glands [10, 11]. Other macrolides found in frogs include gephyromantolide A (**2**) [9], from *Gephyromantis boulengeri*, and its regioisomer, cinnamomeoventrolide (**3**) [12], occurring in the gular gland, another type of scent‐dissimulating organ in frogs, of *Hyperolius cinnamomeoventris* (Hyperoliidae). Frogolide (**4**) was first identified in *Hyperolius viridiflavus* and was subsequently found in several other frog species in the families Hyperoliidae and Mantellidae [13]. Gephyromantolide B (**5**) was also found in *H. viridiflavus* [13]. The presence of several other branched macrolides from frogs [8, 14, 15, 16] suggests that the inventory of these compounds in amphibians is far from being complete. Here, we elucidate the structure and absolute configuration and describe the total synthesis of new macrolides from the mantellid *Gephyromantis granulatus* (Figure S1C), granolides A‐D (**6**‐**9**).

## 2 Results and Discussion

### 2.1 Identification

**FIGURE 2:**
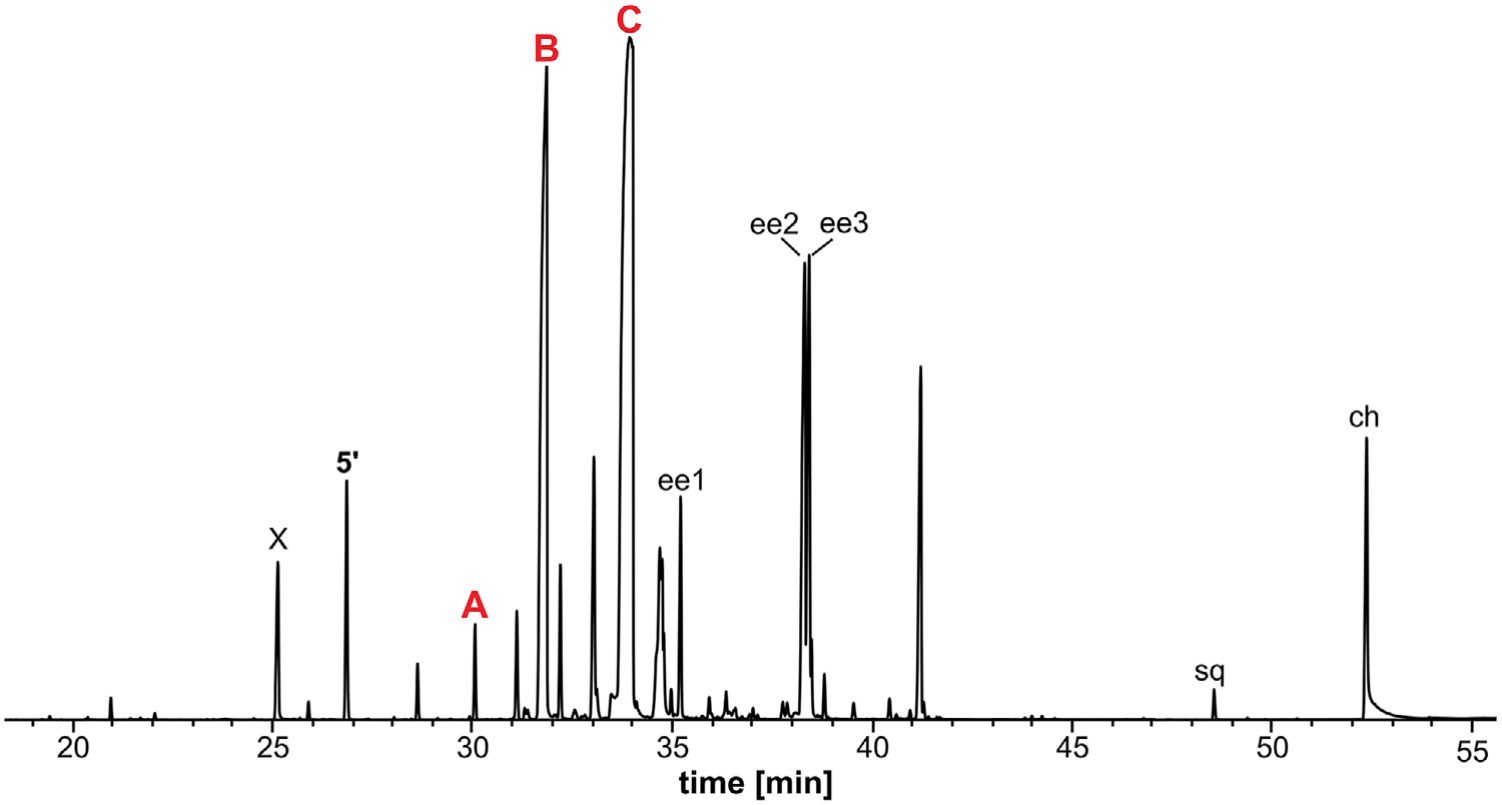
Total ion chromatogram of a femoral gland extract of one *G. granulatus* male (HP5‐MS‐phase, temperature program 50°C – 5 min – 5°C/min – 320°C – 5 min). X, anaesthetic; sq, squalene; ch, cholesterol; diastereomer **5’** of gephyromantolide B (**5**), ethyl palmitate (ee1), ethyl linoleate (ee2), ethyl oleate (ee3). Unknown macrolides **A** (*I* 1737), **B** (*I* 1814), and **C** (*I* 1915).

A femoral gland extract from one *G. granulatus* male collected in 2016 in Marojejy National Park, north‐eastern Madagascar, was analyzed by GC/MS and revealed a complex mixture of compounds (Figure 2). Besides some common fatty acid ethyl esters (ee), squalene (sq), cholesterol (ch), and a diastereomer of gephyromantolide B (**5′**) [17], three unknown compounds **A**‐**C** occurred. We were especially interested in the main component **C**, with a gas chromatographic retention index *I* of 1737, as well as components **A** and **B** (*I* 1814 and 1915), which, based on mass spectral analysis, appeared to share the same structural motif.

The HRMS data showed a molecular mass of *m*/*z* 250.1928 for compound **A** (calcd. 250.1927 for C_16_H_26_O_2_), *m*/*z* 264.2088 for compound **B** (calcd. 264.2084 for C_17_H_28_O_2_), and *m/z* 278.2242 for compound **C** (calcd. 278.2240 for C_18_H_30_O_2_) with four double‐bond equivalents for each compound. Hydrogenation of the natural sample revealed that compounds **A**‐**C** contained two double bonds. Since we did not have sufficient material for NMR analyses due to the difficulties of catching frogs in the rainforest, conservation issues, and the absence of captive breeding colonies of *G. granulatus*, we focused on a careful analysis of the mass spectra. This analysis indicated macrocyclic lactones, since typical macrolide fragments (e.g., loss of two H_2_O molecules and the M‐60 ion) were observed [18, 19]. Interpreting the mass spectra of macrolides can be quite complex because initial fragmentation results only in ring opening, rather than producing smaller fragments. Once the ring has opened, there are more possibilities for secondary fragmentations because the position of the charges and radicals can change [19, 20].

**FIGURE 3:**
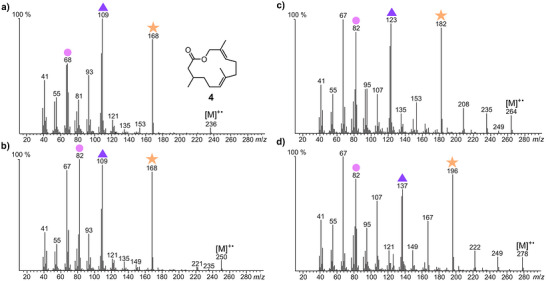
Mass spectra of (a) frogolide (**4**), (b) macrolide **A**, (c) macrolide **B**, and (d) macrolide **C** from *G. granulatus*. The symbols indicate relevant fragments from the fragmentation mechanism in Scheme 1.

**SCHEME 1:**
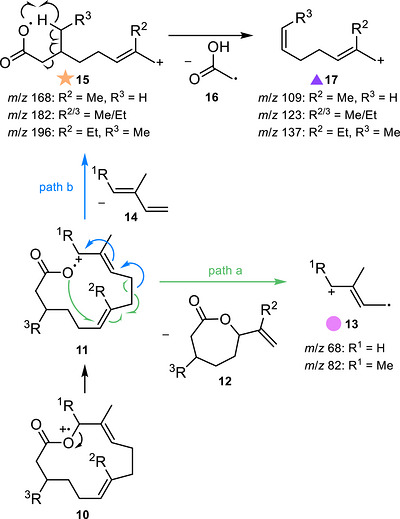
Proposed fragmentation mechanism of frogolide (**4**) and macrolides **A**–**C**.

Some guidelines for interpreting the mass spectra of macrolides were developed in our work group earlier [20], allowing, e.g., to determine the ring size of saturated macrolides or to localize the position of double bonds in monounsaturated macrolides. However, analyzing the mass spectra of diunsaturated macrolides, especially those with branches, is challenging [20]. Fortunately, the mass spectra of macrolides **A**‐**C** seemed to resemble the fragmentation pattern of frogolide (**4**). The molecular ion of **4** is *m/z* 236, and we can observe a continuous mass gain of 14 amu from **4** to **A** (*m/z* 250), then to **B** (*m/z* 264), and finally to **C** (*m/z* 278), indicating additional CH_2_ groups (Figure 3). Characteristic ions also shift, and the analysis of the potential fragmentation mechanism allowed us to postulate the positions of the additional carbons (Scheme 1). First, an inductive cleavage occurs at the C‐O bond. By following path a, ion **11** collapses to form radical cation **13**, and a stable ε‐lactone **12**.

With R^1^ = H, **13** corresponds to *m*/*z* 68 in frogolide (**4**) (Figure 3a, circle). In the three macrolides **A**–**C**, R^1^ is likely a methyl group, resulting in the ion *m*/*z* 82 (Figure 3b–d, circle). The distonic radical cation **15** results from cleavage according to path b with loss of a stable diene **14**, leading to *m*/*z* 168 in both **4** and **A**, indicating that both should have R^2^ = methyl and R^3^ = H (Figure 3a,b, asterisk). A H‐transfer and loss of the acetoxy group finally yields fragment **17**, which corresponds to *m*/*z* 109 in both **4** and **A** (Figure 3a,b, triangle). Macrolide **B** shows fragment ions at *m*/*z* 182 and 123, corresponding to an additional carbon (Figure 3c star and triangle). However, the fragmentation mechanism does not allow us to determine its exact location, either R^2^ or R^3^. For macrolide **C**, fragment ions with *m*/*z* 196 and 137 are observable, indicating two additional carbons, likely with R^2^ = ethyl and R^3^ = methyl (Figure 3d, star and triangle).

**SCHEME 2:**
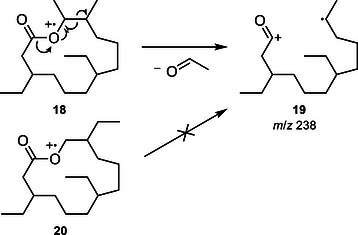
Formation of ion *m*/*z* 238 from hydrogenated macrolide **C**, **18**.

In principle, the additional carbons could be located at several locations of the discussed ions. However, a strong ion M‐29 indicated the presence of ethyl groups, as this ion is absent in **4**. Therefore, we postulated that compound **A** is the homofrogolide **6**, **B** the bishomofrogolide **7** or **8**, and **C** the trishomofrogolide **9**. Alternatively, a ring closure on the terminal methyl group seemed also to be possible, as in **20** (Scheme 2). To clarify this ambiguity, a small part of the natural extract was hydrogenated. The major product, saturated **C**, showed a mass spectrum with an M‐44 ion (*m/z* 238, Scheme 2 and Figure S2). This ion indicates an ω‐1 lactone ring closure consistent with structure **9** [20], which would not be the case for the derivative **20** with an ω‐2 connectivity. Therefore, and because of the relatively small M‐29 ion in **A**, we assumed that structures ‐**6**–**9** represent the most likely compounds **A**–**C**. These macrolides are sesquiterpenes, but the presence of an ethyl rather than a methyl branch is a unique feature rarely observed in nature. Therefore, we decided to synthesize the proposed structures to prove the constitution, elucidate the stereochemical issues of the target molecules, and determine the absolute configuration of the natural compounds **A**–**C**.

### 2.2 Synthesis

**SCHEME 3:**
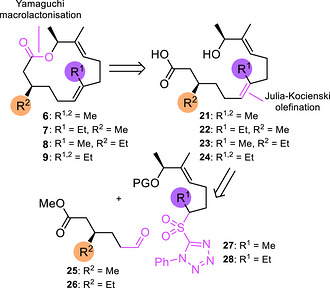
Modular synthesis to access all different substitution patterns of the macrolides **A**‐**C**. R^1,2 ^= Me, Et; PG = protecting group.

We developed an enantioselective, modular total synthesis to access all different substitution patterns and stereoisomers of macrolides **A**–**C** (Scheme 3). The synthesis included a Yamaguchi macrolactonization and a Julia–Kocienski olefination as key steps. Macrolides **A**–**C** can all be synthesized by varying the combination of building blocks **25**–**28**, each carrying either methyl or ethyl substituents. The establishment of sterically demanding trisubstituted double bonds can be challenging; therefore, we favored a Julia–Kocienski olefination over a Wittig or metathesis approach [21]. Carrying out the synthesis with a mixture of all double‐bond isomers, followed by separation using argentation chromatography, provided a cost‐ and time‐efficient method for accessing all stereoisomers, enabling us to determine the constitution and absolute configuration of macrolides **A**‐**C**.

**SCHEME 4:**
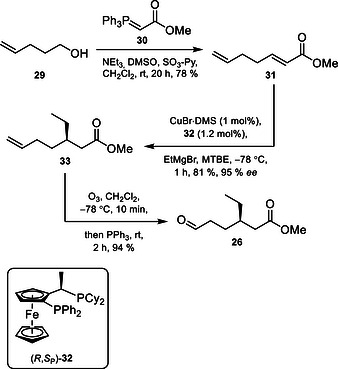
Synthesis of aldehyde building block (*R*)‐**26**.

The synthesis of the ethyl building block **26** commenced with an in situ Parikh–Doering oxidation and *E*‐selective Wittig olefination, resulting in ester **31** (Scheme 4) [22]. The stereogenic center was introduced by an enantioselective copper‐catalyzed conjugate addition with (*R*,*S*_P_)‐Josiphos (**32**) as chiral ligand, according to Feringa and coworkers, delivering (*R*)‐**33** with an *ee* of 95 % [23]. Finally, an ozonolysis completed the aldehyde building block (*R*)‐**26**. The synthesis was also performed with (*S*,*R*_P_)‐Josiphos, giving access to (*S*)‐**26**.

**SCHEME 5:**
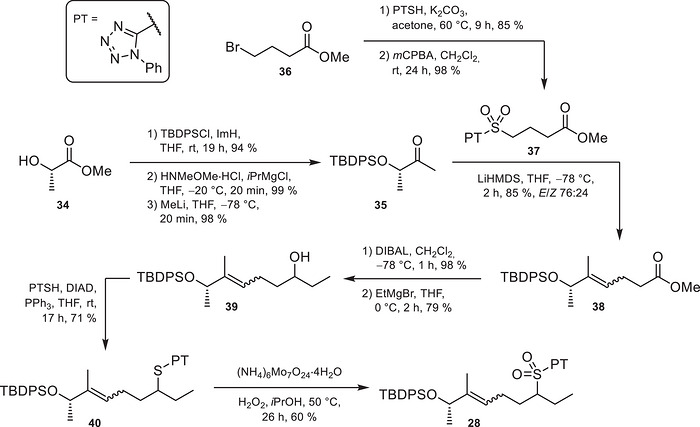
Synthesis of sulfone building block (*S*)‐**28**.

For the preparation of the sulfone building block (*S*)‐**28**, (*S*)‐methyl lactate (**34**) was converted into the silyl ether using *tert*‐butyldiphenylchlorosilane (TBDPSCl), and the ketone (*S*)‐**35** was obtained via Weinreb amide ketone synthesis (Scheme 5). In parallel, the sulfone **37** was synthesized in two steps starting from bromide **36** and 1‐phenyl‐1*H*‐tetrazol‐5‐thiol (PTSH), followed by oxidation with *meta*‐chloroperoxybenzoic acid (*m*CPBA). A Julia–Kocienski olefination of ketone (*S*)‐**35** and sulfone **37** afforded the alkene (*S*)‐**38** in an *E*/*Z* ratio of 76:24 [24]. The alcohol (*S*)‐**39** was then synthesised via reduction with diisobutylaluminium hydride (DIBAL) and a Grignard reaction with ethylmagnesium bromide. A final Mitsunobu reaction followed by oxidation with ammonium heptamolybdate and hydrogen peroxide afforded the sulfone building block (*S*)‐**28**. Using isopropanol instead of the commonly used ethanol and increasing the temperature to 40 °C shortened the reaction time from several days to one day. The reaction was prematurely terminated because the double bond was oxidised to the epoxide over time. The sulfide and the sulfone have almost identical TLC retention factors, which made reaction control difficult. The synthesis was also performed using (*R*)‐methyl lactate (**34**), yielding (*R*)‐**28**.

With the two building blocks (*R*)‐**26** and (*S*)‐**28** in hand, the final Julia–Kocienski olefination was performed, and the diene (3*R*,12*S*)‐**41** was obtained as a mixture of four double bond isomers (ratio GC: 7:8:42:43, Scheme 6) [24]. The hydroxy acid (3*R*,12*S*)‐**24** was synthesized by deprotection with tetra‐*n*‐butylammonium fluoride (TBAF), followed by saponification with LiOH. Finally, (3*R*,12*S*)‐3,7‐diethyl‐11‐methyl‐6,10‐tridecadien‐12‐olide (**9**) was obtained by a Yamaguchi macrolactonization with 2,4,6‐trichlorobenzoyl chloride (TCBC) almost quantitatively [25]. The synthesis was also performed with (*S*)‐**26** and (*R*)‐**28**, giving access to the diastereomer (3*S*,12*S*)‐**9′** and enantiomer (3*S*,12*R*)‐**9**.

**SCHEME 6:**
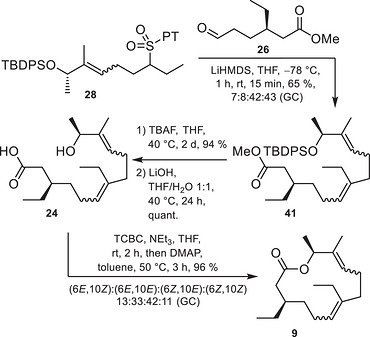
Synthesis of macrolide (3*R*,12*S*)‐**9**.

Fortunately, a comparison of the mass spectra of natural **C** and the four *E*/*Z*‐isomers (3*R*,12*S*)‐**9** showed an excellent match with one of them. The retention index of the matching isomer (*I* 1916) also corresponded well to that of the natural macrolide (*I* 1915). The next step was to clarify the configuration of the double bonds in the fitting isomer. The four isomers were therefore separated by silver nitrate column chromatography. Luckily, the desired isomer could be isolated in pure form for 2D NMR spectroscopy. Figure S7 shows the key ^1^H,^1^H NOESY correlations that were used to determine the double bond configurations (for detailed analysis, see SI). A correlation is observed between H‐10 (5.43–5.39 ppm) and H‐12 (5.36 ppm), but not between H‐17 (1.52–1.50 ppm) and H‐10, confirming an (*E*)‐double bond. The second double bond is also (*E*)‐configured, since H‐6 (5.01 ppm) interacts with one of the H‐8 protons (2.23–2.17 and 1.92–1.85 ppm), but does not interact with the H‐15 protons (2.12–2.04 and 1.90–1.81 ppm). Thus, the double bond configuration of **C** was determined to be (6*E*,10*E*), identical to the configuration of frogolide (**4**), which also contains two (*E*)‐double bonds.

**FIGURE 4:**
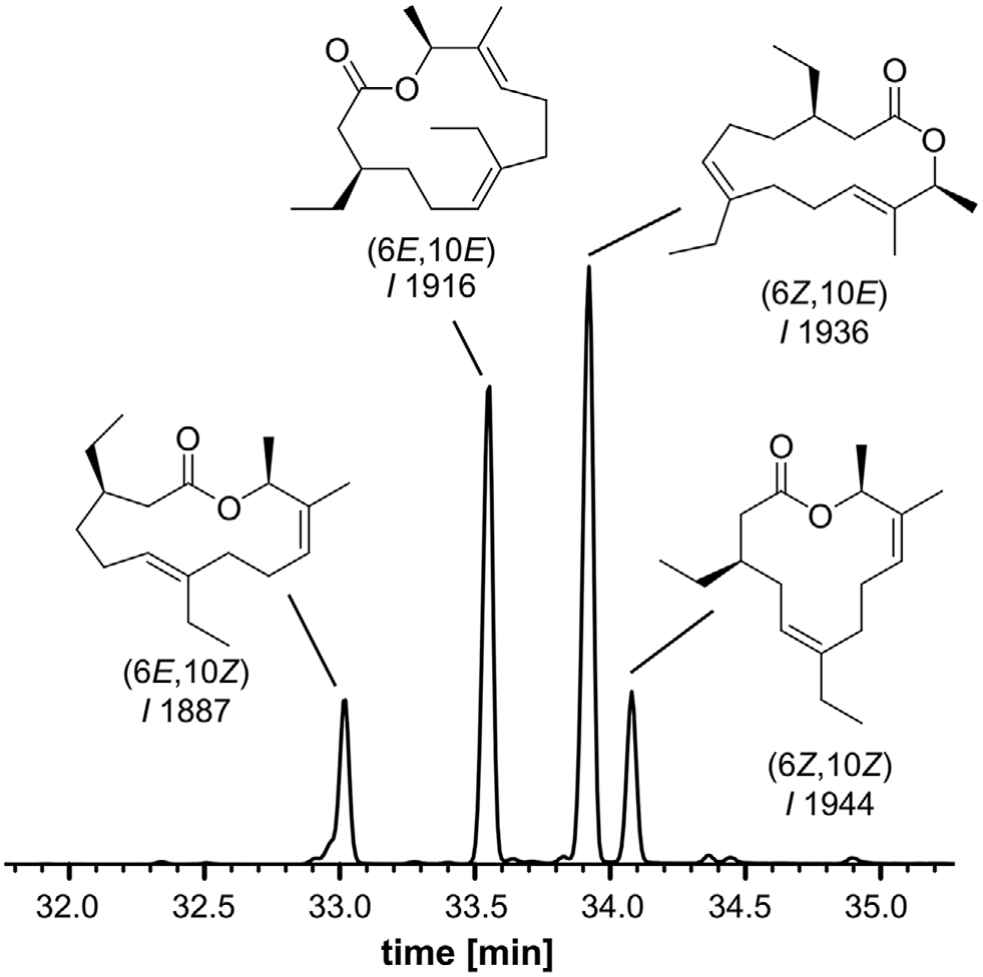
Section of the gas chromatogram of the isomer mixture (3*R*,12*S*)‐**9**. Ratio of the isomers: (6*E*,10*Z*)/(6*E*,10*E*)/(6*Z*,10*E*)/(6*Z*,10*Z*) 13:33:42:11.

Figure 4 shows a section of the gas chromatogram of the isomer mixture (3*R*,12*S*)‐**9**, with each isomer assigned to its corresponding peak. The (6*Z*,10*E*)‐isomer was also isolated and characterized using 2D NMR spectroscopy. There was insufficient pure material of the remaining two isomers for analysis, but the (6*E*,10*Z*) isomer could be isolated during the synthesis of the diastereomer (3*S*,12*S*)‐**9′**. The (6*Z*,10*Z*) isomer was assigned to the peak with the highest retention time, based on the exclusion principle. Thus, the isomers were formed in the ratio of (6*E*,10*Z*)/(6*E*,10*E*)/(6*Z*,10*E*)/(6*Z*,10*Z*) 13:33:42:11. These compounds are all *syn*‐diastereomers.

**FIGURE 5:**
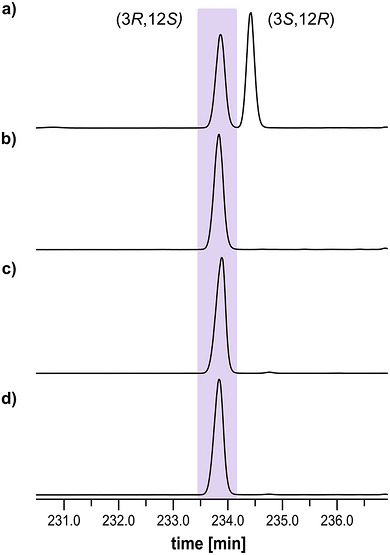
Determination of the absolute configuration of macrolide **C** by GC on a chiral Hydrodex‐β‐6TBDM phase. (a) Co‐injection of (3*R*,12*S*)‐**9** and (3*S*,12*R*)‐**9**, (b) synthetic (3*R*,12*S*)‐**9**, (c) natural sample, (d) co‐injection of (3*R*,12*S*)‐**9** and natural sample.

To exclude coincidental coelution, the *anti‐*diastereomers (3*S*,12*S*)‐**9′** were also synthesized using the appropriate building blocks. Their retention indices of *I* 1859, 1892, 1924, 1941 did not correspond to that of **C** (*I* 1915). Therefore, the absolute configuration of **C** must be either (3*R*,12*S*) or (3*S*,12*R*). Consequently, the (3*S*,12*R*)‐**9** enantiomer was synthesized, and the absolute configuration of macrolide **C** was determined using GC on a chiral Hydrodex‐β‐6TBDM phase (Figure 5).

First, (3*R*,12*S*)‐**9** and (3*S*,12*R*)‐**9** were co‐injected to determine the optimal temperature program for baseline separation of the enantiomers (Figure 5a). To assign the peaks to their respective enantiomers, (3*R*,12*S*)‐**9** was injected alone and assigned to the peak with the lower retention time (Figure 5b). The natural macrolide showed a good match to (3*R*,12*S*)‐**9**, and was co‐injected with this enantiomer for a final confirmation (Figure 5c,d). Thus, the absolute configuration of this new natural compound, called granolide C by us, could be established as (3*R*,6*E*,10*E*,12*S*)‐**9**. The diastereomer (6*E*,10*E*)‐**9′** of granolide C with known relative configuration also occurs in small amounts in the natural sample (Figure S6).

**SCHEME 7:**
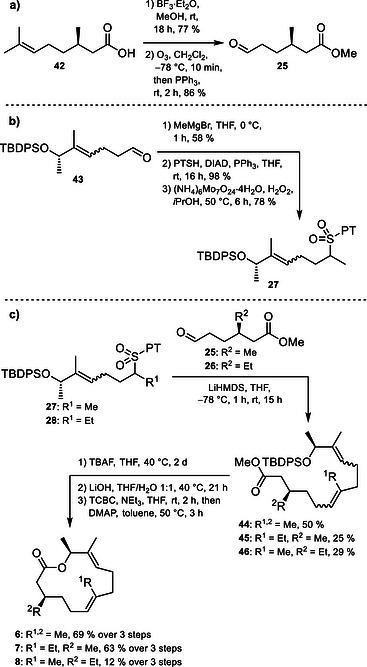
(A) Synthesis of aldehyde building block (*R*)‐**25**. (B) Synthesis of sulfone building block (*S*)‐**27**. (C) Synthesis of the macrolides **6**–**8**.

The synthesis of the remaining macrolides **A** and **B** is shown in Scheme 7. The required methyl aldehyde building block **25** was obtained in two steps, starting from (*R*)‐citronellic acid (**42**) via methylation and ozonolysis (Scheme 7A). Starting from aldehyde **43**, the second sulfone building block **27** was synthesized using methylmagnesium bromide in the Grignard step (Scheme 7B). The structure proposal of macrolide **A**, called granolide A, was confirmed by the synthesis of the macrolide **6** from **25** and **27** (Scheme 7C, see Figure S3 for mass spectra comparison).

Two structures were possible for macrolide **B**, with the ethyl group in either R^1^ or R^2^. Synthesizing both structures proved that the correct structure of granolide B was **7**, which was obtained from **28** and **25** (Scheme 7C, see Figure S4 for mass spectra comparison). We observed that the regioisomer **8** also occurs, but in smaller amounts, in the natural sample, which we named granolide D (Figure S4 and S6). Although the absolute configurations of **A** and **B** were not analyzed, they are likely the same as that of granolide C, since they are obviously derived from the same biosynthetic pathway.

Although we have analyzed more than 20 species of mantellines, we have not yet detected granolides in any other species. They constitute potentially a species‐specific signal used in chemical communication by these frogs. A notable feature of these sesquiterpene macrolides is the presence of ethyl branches rather than the more common methyl branches. Currently, only a few naturally occurring ethyl‐branched terpenes are known, e.g., insect juvenile hormones [26] or faranal [27] from the poison glands of the Pharaoh ant. The ethyl branches are presumably formed biosynthetically through the exchange of an acetyl‐CoA with a propionyl‐CoA (Section S6) [26, 28]. The ethyl groups can also be formed by radical methylation, as is the case in the biosynthesis of sitosterol, for example [29]. However, feeding experiments using isotopically labelled propionyl‐CoA would be needed to test the hypothesis, which is difficult to carry out in frogs. A large number of frogs would be necessary, whose collection would not be permitted under current legislation in Madagascar due to conservation concerns [8]. Examining the biological activity of granolide C would also be interesting, but unfortunately, this presents the same problems of sample size. Furthermore, frogs in captivity are difficult to stimulate to show natural behavior, which complicates bioassays [8, 9].Genome analysis may, in the future, provide information on biosynthetic pathways, but amphibian genomes are, in general, rather large and not yet straightforward to sequence and analyze [30]. Granolide C is presumably used for chemical communication. This compound may therefore contribute to species formation and maintenance of species boundaries, as has been proposed for mantelline femoral gland secretions in general, given that many frog species live sympatrically in relatively small areas of Madagascar [8, 31, 32].

## 3 Conclusion

Here, we reported the structure elucidation and total synthesis of new sesquiterpene macrolides – granolide A‐C. Structure elucidation was performed using gas chromatography with mass spectrometry only, as there was insufficient material for NMR analysis. Total synthesis was needed to unequivocally establish the unique structures of these ethyl‐branched sesquiterpenoid macrocyclic lactones. The absolute configuration was determined to be (3*R*,6*E*,10*E*,12*S*) for granolide C (**9**). Using an enantioselective and modular synthesis approach, the macrolides granolide A and B were also synthesized, and their structures confirmed.

## Author Contributions

**Johanna Kuhn**: formal analysis, investigation, validation, visualization, writing – original draft. **Miguel Vences**: conceptualization, resources. **Stefan Schulz**: conceptualization, methodology, supervision, resources, validation, writing – review and editing.

## Conflicts of Interest

The authors declare no conflicts of interest.

## Supporting information



The authors have cited additional references [33, 34, 35, 36, 37, 38, 39, 40, 41, 42, 43, 44, 45, 46, 47, 48, 49, 50, 51, 52] within the Supporting Information. Mass spectral data for this article will be available after publication in the open‐access mass spectra repository MACE [http://www.oc.tu‐bs.de/schulz/html/MACE.html], located at the Leopard server of TU Braunschweig. **Supporting File 1**: chem71139‐sup‐0001‐SuppMat.pdf.
