# Supplementary material for: Structure Elucidation and Total Synthesis of Granolides A–C: Ethyl‐branched Sesquiterpenes From the Tropical Frog Gephyromantis granulatus
Source: Chemistry. 2026 May 28;32(25):e71139. doi: 10.1002/chem.71139 (PMC13331588; doi:10.1002/chem.71139)
Supplement: Supplementary file 1 — The authors have cited additional references [33, 34, 35, 36, 37, 38, 39, 40, 41, 42, 43, 44, 45, 46, 47, 48, 49, 50, 51, 52] within the Supporting Information. Mass spectral data for this article will be available after publication in the open‐access mass spectra repository MACE [http://www.oc.tu‐bs.de/schulz/html/MACE.html], located at the Leopard server of TU Braunschweig. Supporting File 1: chem71139‐sup‐0001‐SuppMat.pdf. [file CHEM-32-e71139-s001.pdf]

Table of Contents

1 Pictures of mantellid frogs S1
2 Mass spectrum of hydrogenated macrolide **C** S1
3 Mass spectra of the natural and synthetic macrolides **A-D** S2
4 Gas chromatogram of the femoral gland extract S5
5 NOESY correlations of granolide C (**9**) S5
6 Proposed biosynthesis of granolide C (**9**) S6
7 Materials and methods S7
7.1 Origin of the femoral gland sample S7
7.2 General methods S7
8 Synthetic procedures S11
8.1 Synthesis of the aldehyde building blocks **25** and **26** S11
8.2 Synthesis of the sulfone building blocks **27** and **28** S15
8.3 Synthesis of the macrolides **6-9** S28
9 References S50
10 NMR spectra S52

1 Pictures of mantellid frogs

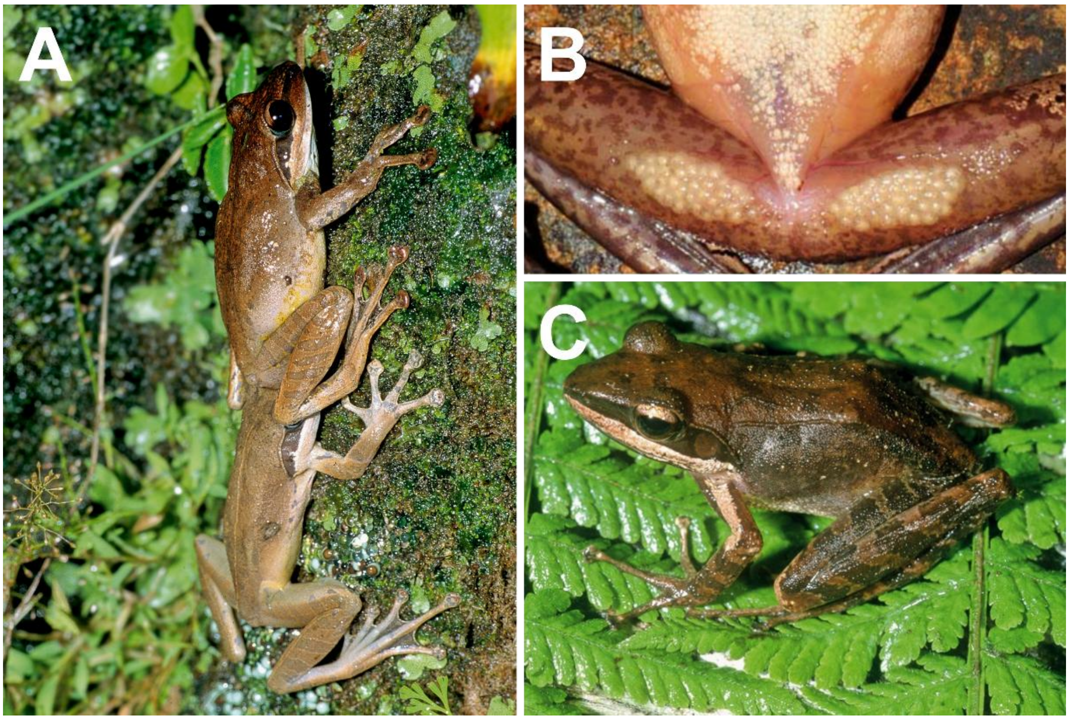

Fig. S1: A) Reproductive behaviour in mantellid frogs of the subfamily Mantellinae: Guibemantis tornieri, B) Femoral glands of a Gephyromantis grosjeani male and C) Gephyromantis granulatus (photos: M. Vences).

2 Mass spectrum of hydrogenated macrolide **C**

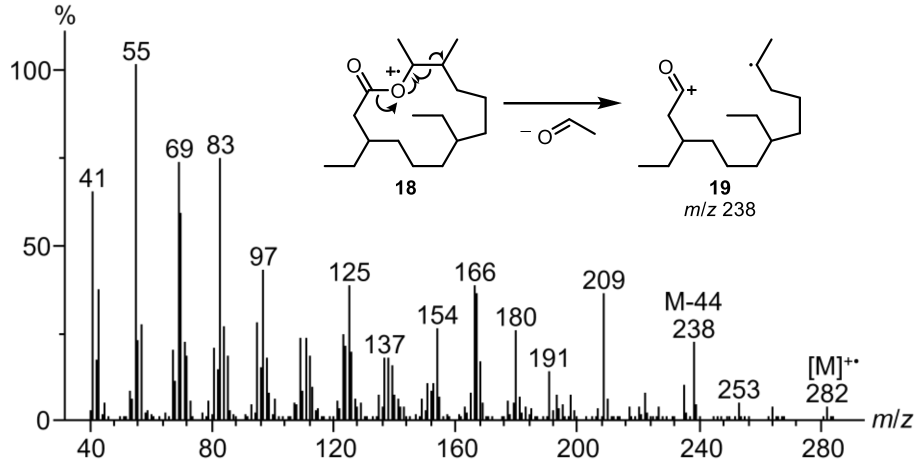

Fig. S2: Mass spectrum of one hydrogenated **C** isomer from the natural gland sample.

3 Mass spectra of the natural and synthetic macrolides **A**-**D**

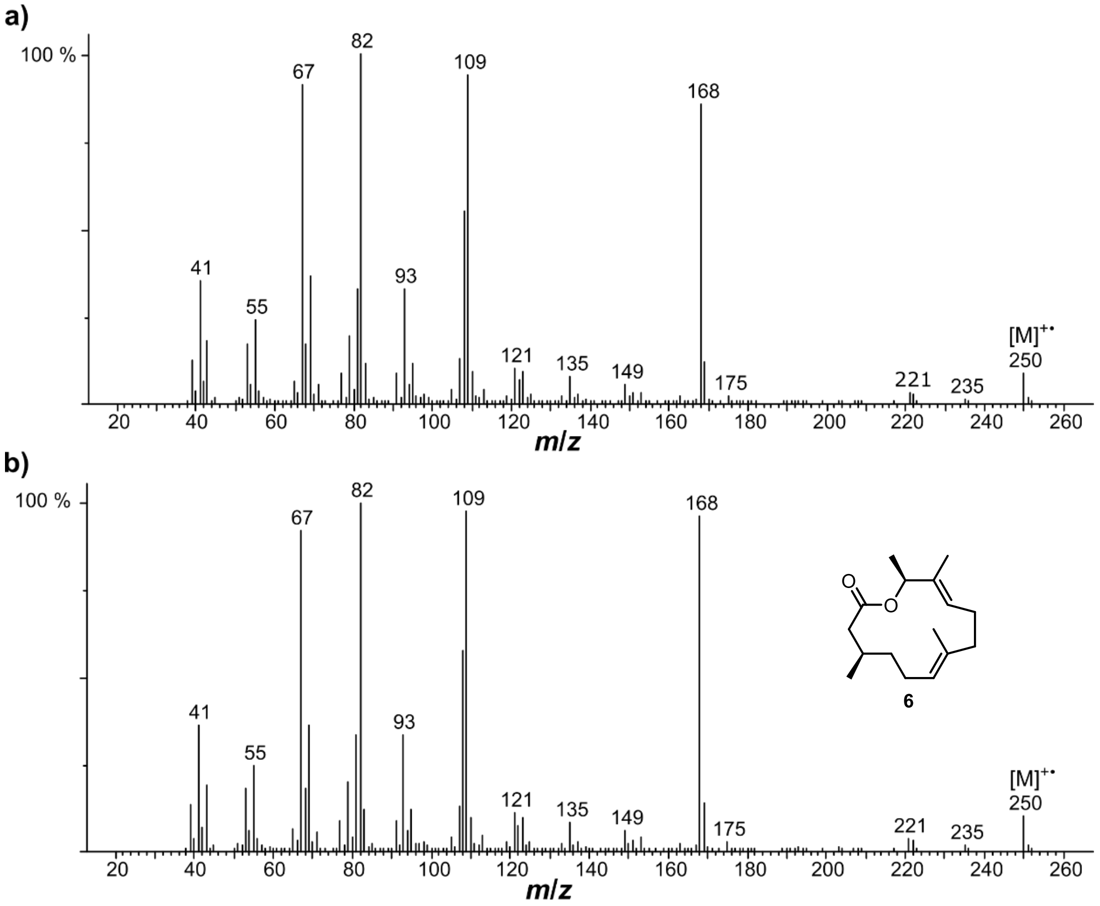

Fig. S3: Mass spectra of a) macrolide **A** from *G. granulatus* and b) macrolide **6**.

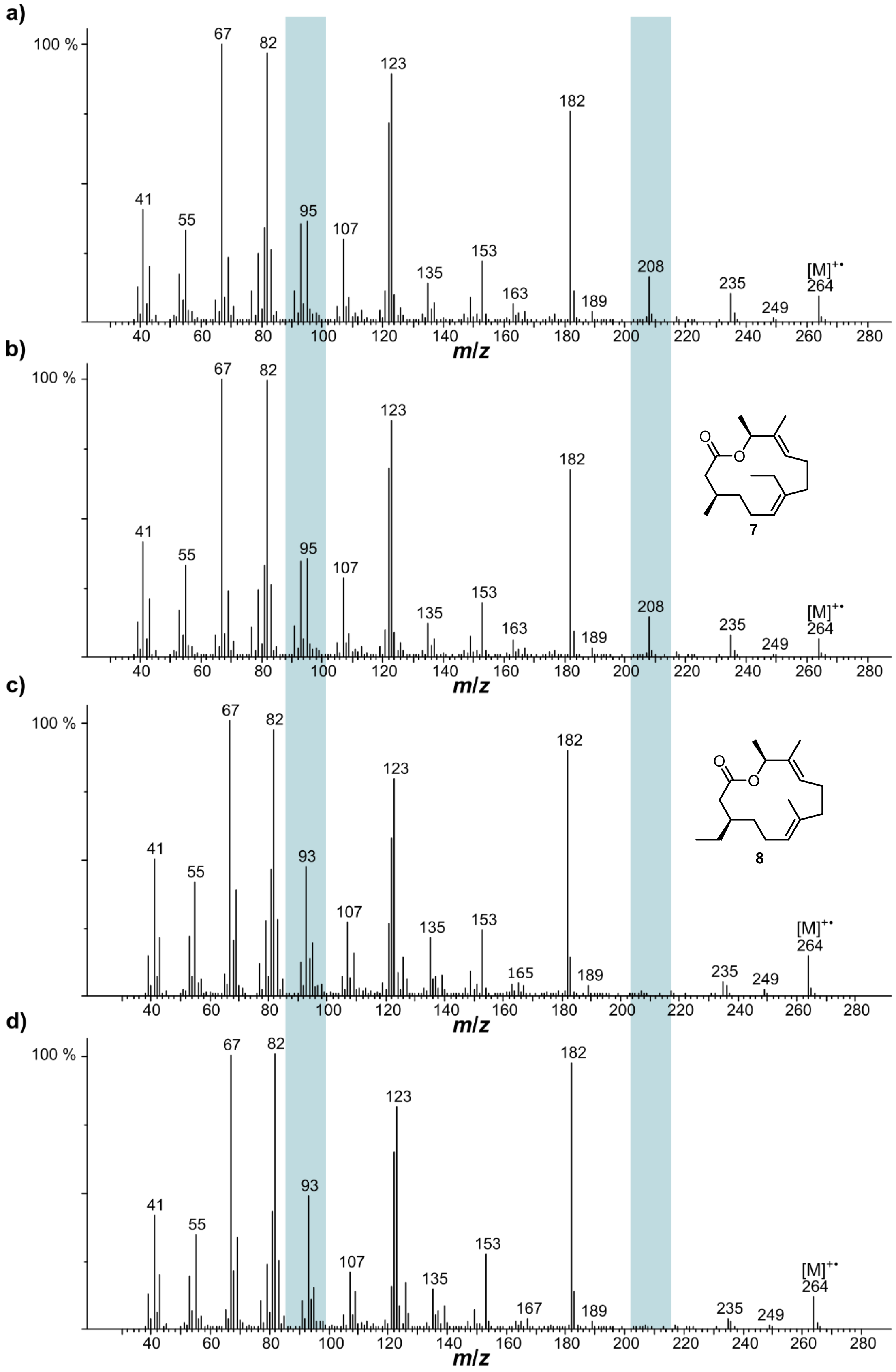

Fig. S4: Mass spectra of a) macrolide **B** from *G. granulatus*, b) macrolide **7**, c) macrolide **8** and d) macrolide **D** from *G. granulatus*. The blue boxes show the significant differences in the mass spectra.

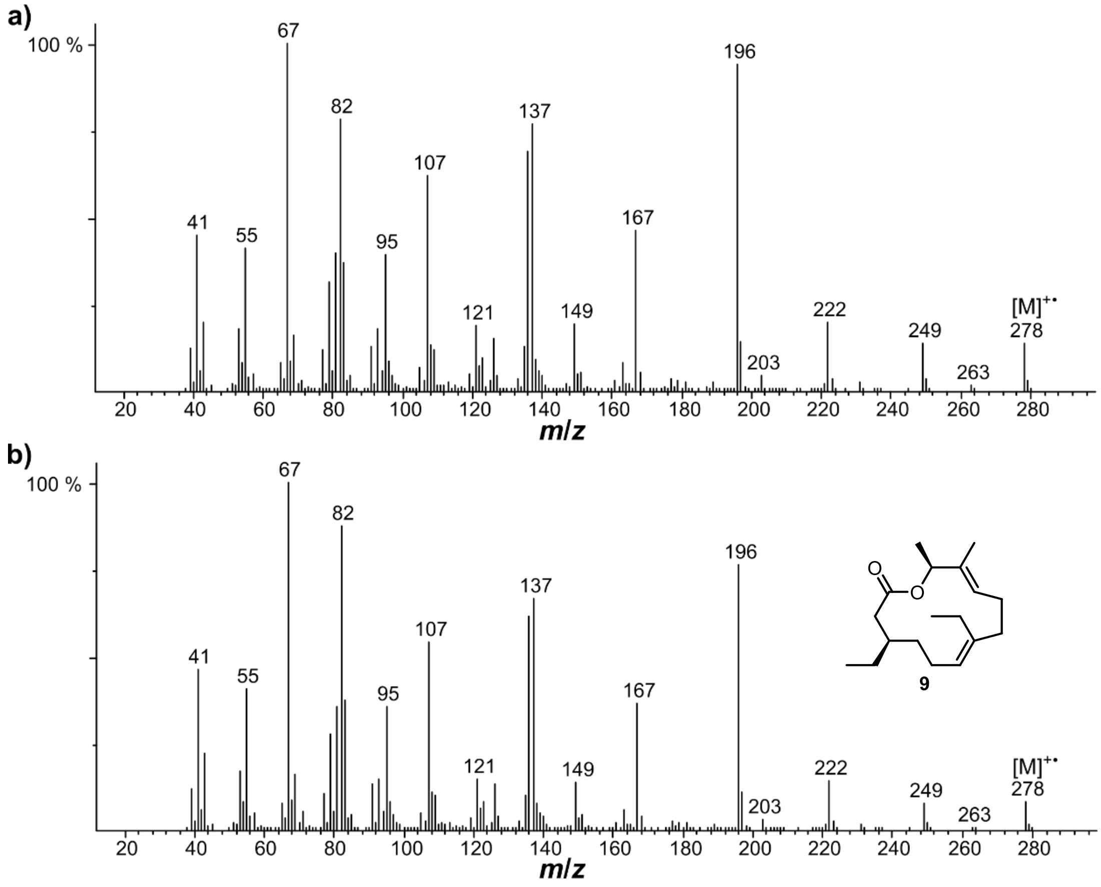

Fig. S5: Mass spectra of a) macrolide **C** from *G. granulatus* and b) macrolide (3*R*,12*S*)-**9**.

4 Gas chromatogram of the femoral gland extract

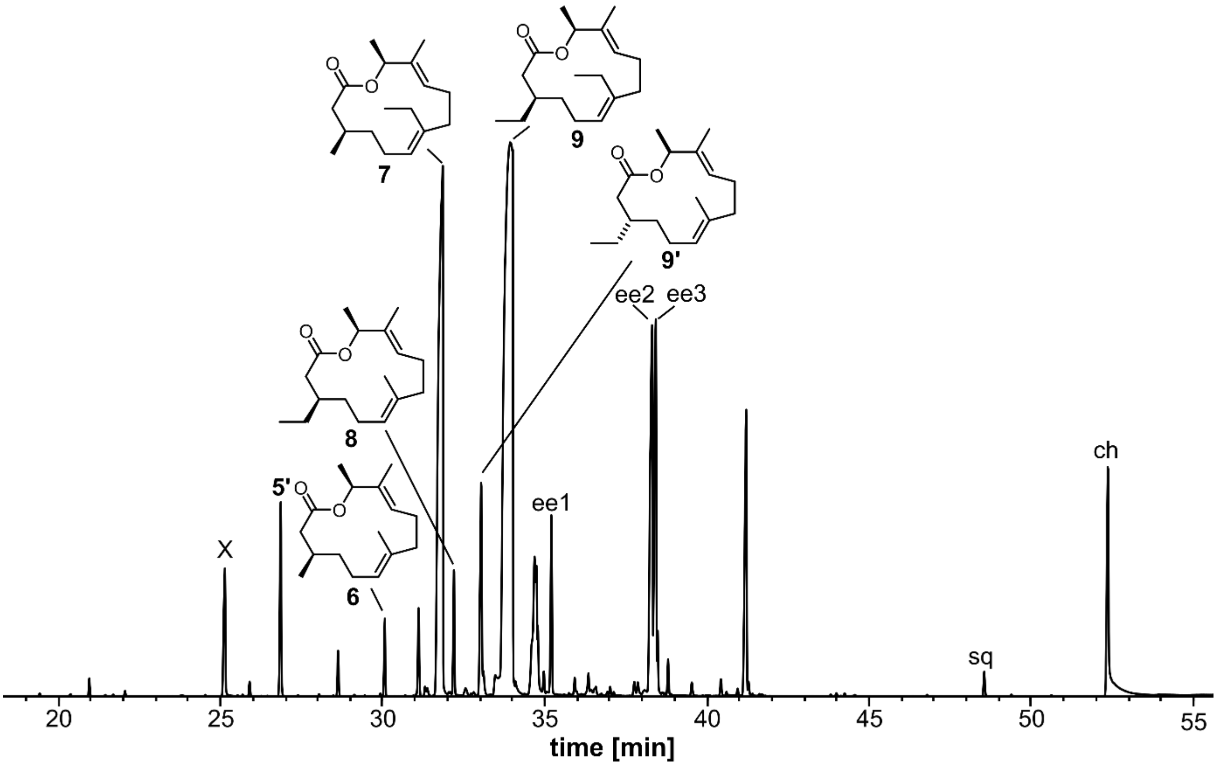

Fig. S6: Total ion chromatogram of a femoral gland extract of one *G. granulatus* male (HP5-MS-phase, temperature program 50 °C – 5 min – 5 °C/min – 320 °C – 5 min). X = anesthetic, sq = squalene, ch = cholesterol, diastereomer **5'** of gephyromantolide B (**5**), ethyl palmitate (ee1), ethyl linoleate (ee2), ethyl oleate (ee3), granolide A (**6**, *I* 1737), granolide B (**7**, *I* 1814), granolide D (**8**, *I* 1839), granolide C (**9**, *I* 1915), diastereomer (6*E*,10*E*)-**9'** (*I* 1885) of **9** with known relative configuration.

5 NOESY correlations of granolide C (**9**)

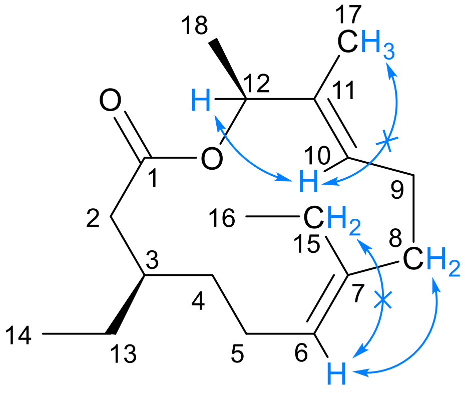

Fig. S7: Key $^1H,^1H$ NOESY correlations (arrows) of macrolide (3*R*,6*E*,10*E*,12*S*)-**9**.

6 Proposed biosynthesis of granolide C (**9**)

The biosynthesis of the granolides proceeds via the mevalonate pathway of the terpene biosynthesis (Scheme S1). The ethyl branches are presumably formed by the exchange of acetyl-CoA with propionyl-CoA, which is likely formed from isoleucine or valine.[1]

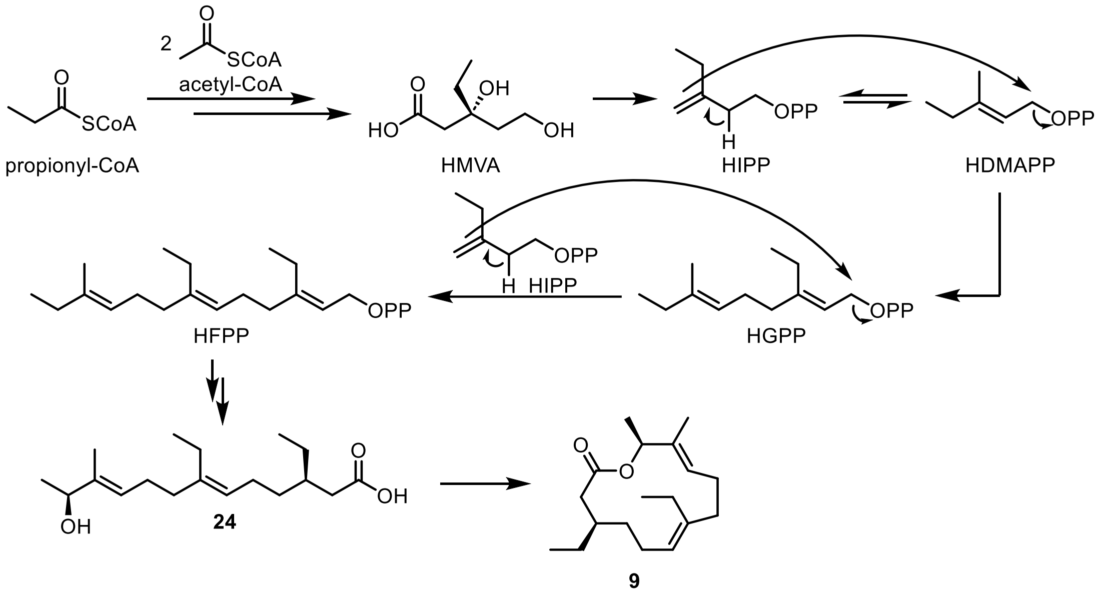

Scheme S1: Proposed biosynthesis of granolide C (**9**). CoA: coenzyme A, HMVA: homomevalonic acid, PP: pyrophosphate, HIPP: homoisopentenyl pyrophosphate, HDMAPP: homodimethylallyl pyrophosphate, HGPP: homogeranyl pyrophosphate, HFPP: homofarnesyl pyrophosphate.

Homomevalonic acid (HMVA) is formed from propionyl-CoA and two acetyl-CoA units in several steps, yielding homoisopentenyl pyrophosphate (HIPP). HIPP isomerizes to homodimethylallyl pyrophosphate (HDMAPP), which forms homogeranyl pyrophosphate (HGPP) with another HIPP unit. Integrating another HIPP unit yields homofarnesyl pyrophosphate (HFPP), and reduction of the C2 double bond, oxidation to the acid and ω–1-oxidation lead to the hydroxy acid **24**. This is finally cyclised to granolide C (**9**).

7 Materials and methods

7.1 Origin of the femoral gland sample

The male *G. granulatus* individual with field number ZCMV 15317 was collected by M. Vences in Marojejy National Park, northeastern Madagascar, in 2016. It was sedated with benzocaine and subsequently euthanised with an overdose of the same substance. Femoral gland tissue was then removed using sterile instruments, before being stored in 1 ml GC vials with Teflon caps under dichloromethane at –80 °C.

7.2 General methods

Chemicals were obtained from the following companies: abcr, Acros Organics, Alfa Aesar, Fisher Scientific, Merck, Sigma-Aldrich, VWR and TCI. Unless otherwise noted, they were used without further purification. Reactions involving moisture- and air-sensitive substances were carried out in oven-dried glassware under a nitrogen atmosphere. Technical-grade solvents were purified by simple distillation. Absolute solvents were dried according to standard methods if necessary. SupraSolv® solvents were used for natural samples.

Thin-layer chromatography (TLC) was performed on Polygram® SIL-G/UV254 silica gel sheets (Macherey & Nagel), using either a molybdophosphoric acid (10 % in ethanol) or a potassium permanganate staining reagent (15 g $KMnO_4$, 10 g K_2CO_3, 2.5 ml 5 wt% NaOH, 150 ml H_2O), followed by heating for detection. Column chromatographic separations were performed using the flash technique on silica gel 60 (Fluka, particle size 0.040–0.063 mm, mesh 230–440 ASTM) with *n*-pentane (Pe) or diethyl ether (Et_2O) as solvent. For column chromatographic separations of silver nitrate on silica gel, a 10 wt% silver nitrate-silica gel mixture was prepared according to a procedure of Li et al.[2] The silver nitrate was dissolved in water and mixed with the silica gel. Sufficient water was added to ensure the silica gel was completely covered, and the mixture was then dried at 120 °C. Silver nitrate column chromatography was performed without compressed air and in the dark.

^{1}H-NMR and ^{13}C-NMR spectra were recorded using following Bruker spectrometers: Avance II 300 (300 MHz for ^{1}H and 75 MHz for ^{13}C), Avance III HD 300N (300 MHz for ^{1}H and 75 MHz for ^{13}C), Avance III 400 (400 MHz for ^{1}H and 100 MHz for ^{13}C), Avance III HD 500 (500 MHz for ^{1}H and 125 MHz for ^{13}C) and Avance III HD 600 (600 MHz for ^{1}H and 150 MHz for ^{13}C). Tetramethylsilane was used as the internal standard (δ = 0 ppm). Multiplicities in the ^{1}H NMR spectrum are described as follows: singlet (s), broad singlet (br. s), doublet (d), triplet (t), quartet (q), quintet (quin), sextet (sext) or septet (sept). Multiplicities in the ^{13}C NMR spectrum are described as primary (CH_3), secondary (CH_2), tertiary (CH) or quaternary (C_q).

GC/MS analyses of the synthesised compounds for reaction control were performed using an Agilent Technologies 5977B coupled with an Agilent Technologies 8860 Series MSD. For chromatography, an HP-5-MS fused silica capillary column (Agilent Technologies, 30 m length, 0.25 mm diameter, 0.25 µm film thickness, 350 °C) with a helium carrier gas at a volume flow rate of 1 ml/min was used. The temperature program started at 50 °C and the temperature was held for five minutes. The column was then heated to 320 °C at 20 °C/min and held at this temperature for an additional five minutes. The synthesis samples were injected in split mode with a split ratio of 35:1. GC/MS analyses of the natural samples were performed using an Agilent Technologies 7890A and an Agilent Technologies 5975 Series MSD. For chromatography, an HP-5-MS fused silica capillary column (Agilent Technologies, 30 m length, 0.25 mm diameter, 0.25 µm film thickness, 350 °C) with a helium carrier gas (volume flow rate: 1.2 ml/min) was used. The temperature program started at 50 °C and was held for five minutes. It was then increased to 320 °C at a rate of 5 °C/min and held at this temperature for a further five minutes. The natural samples were injected in splitless mode and the synthetic samples in split mode with a ratio of 35:1. Mass spectrometry was performed on all instruments in electron ionisation (EI) mode at 70 eV, and the retention indices (*I*) were determined using a homologous series of *n*-alkanes (C_8–C_{40}). GC/FID measurements on chiral stationary phases were performed using Agilent 7890A and Agilent 7820A gas chromatographs with hydrogen (flow rate 1.5 ml/min) as the carrier gas.

The following capillary columns and temperature programs were used:

Methyl (*R*)-3-ethylhept-6-enoate (**33**): β−DEX 225 column (Supelco, 30 m length, 0.25 mm diameter, 0.25 µm film thickness, 230 °C), 50 °C for 130 min, then 20 °C/min to 230 °C for 5 min. t_R = 111.1 min (*R*)-**33**; t_R = 113.4 min (*S*)-**33**.

(3*R*,6*E*,10*E*,12*S*)-3,7-Diethyl-11-methyl-6,10-tridecadien-12-olide (**9**): Hydrodex-β-6TBDM column (Macherey-Nagel, 25 m length, 0.25 mm diameter, 0.25 µm film thickness, 230 °C), 50 °C for 5 min, then 5 °C/min to 100 °C for 210 min, then 5 °C/min to 230 °C for 5 min. t_R = 233.9 min (3*R*,12*S*)-**9**; t_R = 234.4 min (3*S*,12*R*)-**9**.

High-resolution mass spectrometry was performed using a Thermo Fisher Scientific Trace 1310 gas chromatograph. For chromatography, a ZB5-MS fused silica capillary column (Phenomenex, 30 m length, 0.25 mm diameter, 0.25 µm film thickness, 350 °C) was used, with helium (1 ml/min) as the carrier gas. The temperature program began at 50 °C and was held for three minutes. The column was then heated to 310 °C at a rate of 10 °C/min and held for a further three minutes. Samples were injected in split mode with a split ratio of 10:1. Mass spectrometry was performed using an Exactive GC Orbitrap mass spectrometer (Thermo Fisher Scientific). The resolution was set to 60 000 (FWHM, instrument setting at 200 u). The mass range was 50–650 u and two microscans were averaged per data scan. The automatic

gain control (AGC) target was set to 1×10⁶ and the maximum injection time to “auto”. The auxiliary temperatures for transfer lines 1 and 2 were set to 290 °C, and the electron ionisation source temperature to 220 °C. EI was performed at 70 eV in positive mode. Helium (carrier gas) and nitrogen (C-trap supply) were supplied via gas purification cartridges (Thermo Fisher Scientific) to remove moisture and organic impurities. The column base ion at 207.03235 u was used as the barrier mass for internal mass calibration. For positive-mode chemical ionisation (CIP), methane (99.995 %) was used as the CI gas at a flow rate of 1.5 ml/min. For high-resolution mass spectra using positive electrospray ionization (ESI), and LTQ-Orbitrap Velos mass spectrometer (Thermo Fisher Scientific) was used, and the spectra were acquired in direct infusion mode with a typical spray voltage of 2.3–2.8 kV. The tetradecyltrimethylammonium bromide cation (256.29988 amu) was used as the internal standard, and the scan range was set to 130–2000 amu with an acquisition time of 1.6 s and a resolution of 100 000 FWHM at *m*/*z* = 400.

IR spectra were acquired using either a Bruker Tensor 27 (diamond ATR) or a gas chromatograph (Agilent Technologies 7890B) with an HP-5 MS column (Agilent Technologies: 30 m length, 0.25 mm diameter, 0.25 µm film thickness, 350 °C) coupled with a Dani Instruments DiscovIR detector. Helium was used as the carrier gas at a volume flow rate of 1.5 ml/min, and the disc speed was set to 4 mm/min. For synthetic samples, the temperature program started at 50 °C and was held for five minutes. The column was then heated to 320 °C at 20 °C/min and held at this temperature for an additional 5 minutes. For natural samples, the temperature program started at 50 °C and was held for five minutes. The sample was then heated to 320 °C at a rate of 5 °C/min and held at this temperature for five minutes. The natural and synthetic samples were injected in splitless mode. The intensity of a band is given as strong (s), medium (m), weak (w) or broad (br).

Specific rotations $[\alpha]_D^{25}$ were measured using an Anton Paar MCP 150 polarimeter with a 100 mm long cuvette at 589 nm and 25 °C. Dichloromethane was used as the solvent, and the concentration c is given in g/100 ml. The error ranges were determined using the following formulas:

$$[\alpha]_D^{25} = \frac{\alpha}{c \cdot d} = \frac{\alpha \cdot V}{m \cdot d}$$

$$\Delta[\alpha]_D^{25} = \sqrt{\left(\frac{\partial[\alpha]_D^{25}}{\partial m}\,\Delta m\right)^2 + \left(\frac{\partial[\alpha]_D^{25}}{\partial \alpha}\,\Delta\alpha\right)^2 \left(\frac{\partial[\alpha]_D^{25}}{\partial V}\,\Delta V\right)^2}$$

$$= \sqrt{\left(\frac{-\alpha \cdot V}{m^2 \cdot d}\,\Delta m\right)^2 + \left(\frac{V}{m \cdot d}\,\Delta\alpha\right)^2 \left(\frac{\alpha}{m \cdot d}\,\Delta V\right)^2}$$

α = optical rotation, m = mass, d = cuvette length, V = volume. The standard deviations used were: Δm = 0.1 mg for the scale; Δα = 0.004° for the polarimeter and ΔV = 6 µl for the Eppendorf pipette. For macrolides **6**, **7** and **9**, a precision scale (Sartorius MC5) with Δm = 4 µg was used.

For the micro hydrogenation of the natural sample, 20 µl of the sample was transferred to a GC vial with a 200 µl insert, to which palladium on activated carbon was added (tip of a pipette).[3] The sample was kept under a hydrogen atmosphere at room temperature for three hours, with solvent replenished as needed. The resulting solution was transferred to a new GC vial with a 200 µl insert and analysed using GC/MS.

8 Synthetic procedures

8.1 Synthesis of the aldehyde building blocks **25** and **26**

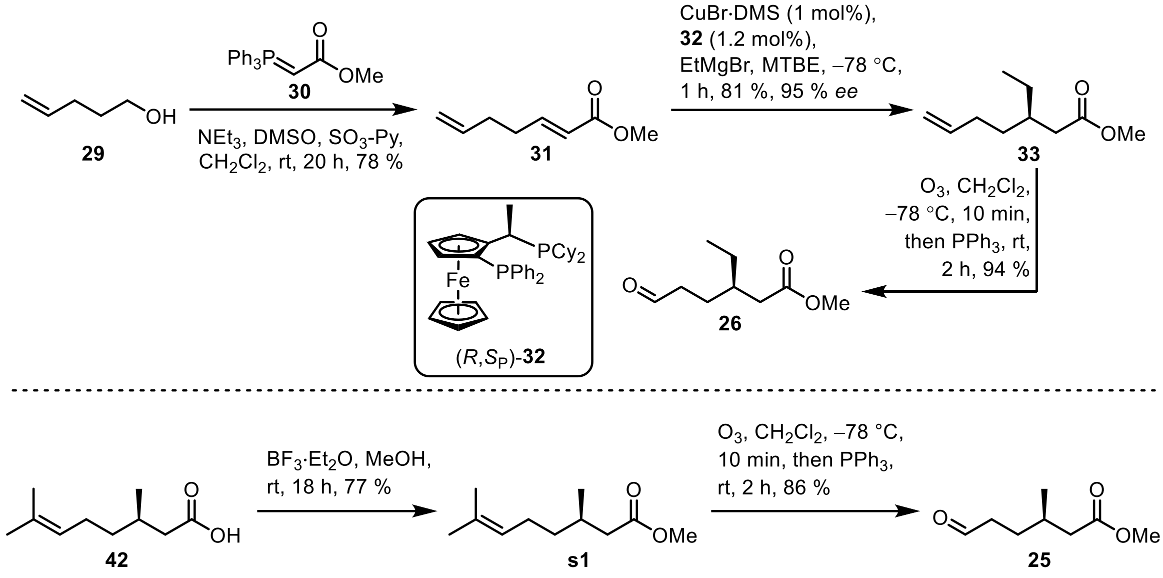

Scheme S2: Synthesis of the aldehyde building blocks **25** and **26**.

8.1.1 Methyl (*E*)-hepta-2,6-dienoate (**31**)

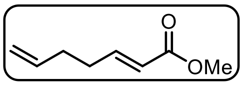

To a solution of 4-penten-1-ol (**29**, 2.07 ml, 20.0 mmol, 1.0 eq.) in dichloromethane (80 ml) was added dimethyl sulfoxide (DMSO, 16 ml), triethylamine (16.6 ml, 120 mmol, 6.0 eq.) and 2-(triphenyl-5-phosphanylidene)methyl acetate (**30**, 13.4 g, 40.0 mmol, 2.0 eq.) at room temperature.[4] After the ylide **30** was completely dissolved, SO_3-pyridine (9.55 g, 60.0 mmol, 3.0 eq.) was added. The dark red mixture was stirred at room temperature for 20 h and then treated with aqueous HCl solution (1 M, 50 ml). The phases were separated, the aqueous phase was extracted with diethyl ether (3×30 ml), the combined organic phases were dried over $MgSO_4$, filtered, and the solvent was removed under reduced pressure. The residue was applied to silica gel, purified by column chromatography (pentane/diethyl ether 20:1), and methyl (*E*)-hepta-2,6-dienoate (**31**) was obtained as a colourless liquid (2.17 g, 15.5 mmol, 78 %). The analytical data are consistent with those published.[5]

R_f = 0.20 (Pe/Et_2O 20:1); **^{1}H NMR** (300 MHz, $CDCl_3$): δ [ppm] = 6.97 (dt, J = 15.7, 6.6 Hz, 1H, =CH), 5.84 (dt, J = 15.7, 1.6 Hz, 1H, =CH), 5.87 – 5.73 (m, 1H, =CH), 5.09 – 4.98 (m, 2H, =CH_2), 3.73 (s, 3H, CH_3), 2.37 – 2.17 (m, 4H, 2×CH_2); **^{13}C NMR** (75 MHz, $CDCl_3$): δ [ppm] = 167.0 (C_q), 148.6 (CH), 137.0 (CH), 121.3 (CH), 115.5 (CH_2), 51.4 (CH_3), 32.0 (CH_2), 31.4 (CH_2); **IR** (ATR, neat): $\tilde{\nu}$ [cm^{-1}] = 3075 (w), 2989 (w), 2943 (w), 2847 (w), 1721 (s), 1653 (m), 1436 (m), 1312 (m), 1272 (m), 1205 (m), 1167 (s), 1037 (m), 987 (m), 914 (m), 853 (m), 814

(m), 713 (w), 634 (w); **EI-MS** (70 eV): *m*/*z* (%) = 140 (1) [M]$^+$, 139 (2), 111 (15), 109 (25), 108 (17), 99 (12), 81 (100), 80 (86), 79 (55), 77 (55), 71 (16), 68 (36), 67 (14), 59 (24), 55 (28), 54 (26), 53 (31), 51 (11), 41 (94), 40 (17), 39 (64); **HRMS** (EI) *m*/*z*: Calculated for $C_8H_{12}O_2$ [M]$^+$ 140.08318, found: 140.08327.

8.1.2 Methyl (*R*)-3-ethylhept-6-enoate (**33**)

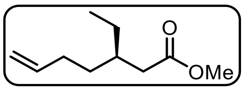

A nitrogen stream was passed through methyl *tert*-butyl ether (MTBE) for 5 min to remove dissolved gases. Subsequently, copper bromide-dimethyl sulfide (CuBr-DMS, 4.4 mg, 21.4 µmol, 1 mol%) and (2*S*$_P$)-1-[(1*R*)-1-(dicyclohexylphosphino)ethyl]-2-(diphenylphosphino)-ferrocene ((*R*,*S*$_P$)-Josiphos, **32**, 16.5 mg, 25.7 µmol, 1.2 mol%) were added to MTBE (13 ml) and stirred at room temperature for 15 min to form the complex.[6] The mixture was then cooled to –78 °C and ethylmagnesium bromide (3 M in Et_2O, 0.75 ml, 2.25 mmol, 1.05 eq.) was added dropwise over 15 min. After stirring for a further 15 minutes, methyl (*E*)-hepta-2,6-dienoate (**31**, 300 mg, 2.14 mmol, 1.00 eq.) in MTBE (4 ml) was added dropwise at –78 °C over one hour using a syringe pump. After the mixture had been stirred at this temperature for one hour, the reaction mixture was quenched by adding methanol (1 ml). The cooling bath was removed, saturated aqueous NH_4Cl solution (20 ml) was added, and the phases were separated. The aqueous phase was extracted with diethyl ether (3×20 ml), the combined organic phases were dried over $MgSO_4$, filtered, and the solvent was removed under reduced pressure. The residue was purified by column chromatography (pentane/diethyl ether 20:1) and methyl (*R*)-3-ethylhept-6-enoate (**33**) was obtained as a colourless liquid (294 mg, 1.73 mmol, 81 %, 95 % *ee*). (*S*)-**33** (95 % *ee*) was obtained under the same conditions by using (2*R*$_P$)-1-[(1*S*)-1-(dicyclohexylphosphino)ethyl]-2-(diphenylphosphino)-ferrocene (**32**).

R$_f$ = 0.41 (Pe/Et_2O 20:1); $[\boldsymbol{\alpha}]_{\mathbf{D}}^{25}$ = +3.17 ± 0.40 (c = 1.01, CH_2Cl_2, (*R*)-**33**); –3.30 ± 0.40 (c = 1.00, CH_2Cl_2, (*S*)-**33**); **^{1}H NMR** (300 MHz, $CDCl_3$): *δ* [ppm] = 5.80 (ddt, *J* = 16.8, 10.2, 6.6 Hz, 1H, =CH), 5.05 – 4.92 (m, 2H, =CH_2), 3.67 (s, 3H, CH_3), 2.29 – 2.23 (m, 2H, CH_2), 2.11 – 2.00 (m, 2H, CH_2), 1.84 (sept, *J* = 6.5 Hz, 1H, CH), 1.50 – 1.24 (m, 4H, 2×CH_2), 0.88 (t, *J* = 7.4 Hz, 3H, CH_3); **^{13}C NMR** (75 MHz, $CDCl_3$): *δ* [ppm] = 173.9 (C_q), 138.7 (CH), 114.4 (CH_2), 51.4 (CH_3), 38.4 (CH_2), 35.9 (CH), 32.6 (CH_2), 30.8 (CH_2), 26.1 (CH_2), 10.7 (CH_3); **IR** (ATR, neat): $\tilde{\nu}$ [cm^{-1}] = 3075 (w), 2926 (m), 2868 (w), 1737 (s), 1641 (w), 1442 (m), 1370 (w), 1249 (m), 1163 (s), 1004 (m), 910 (m), 845 (w), 775 (w), 714 (w), 635 (w); **EI-MS** (70 eV): *m*/*z* (%) = 170 (<1) [M]$^+$, 141 (7), 139 (9), 138 (10), 110 (12), 97 (23), 96 (100), 87 (18), 83 (14), 81 (49), 74 (77), 69 (40), 68 (17), 67 (20), 59 (32), 55 (78), 54 (15), 53 (13), 43 (31), 41 (56), 39 (29); **HRMS** (CIP) *m*/*z*: Calculated for $C_{10}H_{19}O_2$ [M+H]$^+$ 171.13796, found: 171.13785.

8.1.3 Methyl (*R*)-3-ethyl-6-oxohexanoate (**26**)

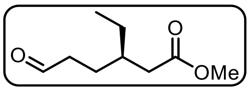

Methyl (*R*)-3-ethylhept-6-enoate (**33**, 250 mg, 1.47 mmol, 1.0 eq.) was dissolved in dichloromethane (15 ml). Ozone was generated using an ozone generator and bubbled through the solution at –78 °C until it turned blue (10 min).[7] Oxygen was passed through the solution until it became colourless, in order to remove excess ozone. Triphenylphosphine (385 mg, 1.47 mmol, 1.0 eq.) was then added and the mixture stirred for 15 min at –78 °C. The cooling was removed, and the reaction mixture was stirred for a further two hours at room temperature. Finally, the solvent was removed under reduced pressure, the residue was applied to silica gel and purified by column chromatography (pentane/diethyl ether 2:1) yielding methyl (*R*)-3-ethyl-6-oxohexanoate (**26**) as a colourless liquid (236 mg, 1.37 mmol, 94 %). (*S*)-**26** was obtained under the same conditions by using methyl (*S*)-3-ethylhept-6-enoate (**33**).

R$_f$ = 0.50 (Pe/Et_2O 1:1); $[\alpha]_D^{25}$ = +7.13 ± 0.40 (c = 1.01, CH_2Cl_2, (*R*)-**26**); –7.20 ± 0.41 (c = 1.00, CH_2Cl_2, (*S*)-**26**); **^{1}H NMR** (300 MHz, $CDCl_3$): *δ* [ppm] = 9.78 (t, *J* = 1.7 Hz, 1H, CHO), 3.67 (s, 3H, CH_3), 2.49 – 2.41 (m, 2H, CH_2), 2.27 (qd, *J* = 15.2, 6.8 Hz, 2H, CH_2), 1.85 (sept, *J* = 6.5 Hz, 1H, CH), 1.75 – 1.55 (m, 2H, CH_2), 1.48 – 1.25 (m, 2H, CH_2), 0.90 (t, *J* = 7.4 Hz, 3H, CH_3); **^{13}C NMR** (75 MHz, $CDCl_3$): *δ* [ppm] = 202.2 (CH), 173.4 (C_q), 51.5 (CH_3), 41.2 (CH_2), 38.1 (CH_2), 35.8 (CH), 26.1 (CH_2), 25.4 (CH_2), 10.7 (CH_3); **IR** (ATR, neat): $\tilde{\nu}$ [cm^{-1}] = 2955 (w), 2724 (w), 1726 (s), 1442 (m), 1375 (w), 1253 (m), 1196 (m), 1162 (m), 1099 (m), 1014 (m), 972 (w), 847 (w), 777 (w), 674 (w), 622 (w); **EI-MS** (70 eV): *m*/*z* (%) = 141 (14), 129 (27), 115 (59), 98 (18), 97 (19), 95 (30), 87 (69), 83 (49), 81 (14), 74 (100), 73 (15), 69 (57), 59 (38), 57 (16), 56 (14), 55 (87), 43 (58), 41 (59), 39 (27); **HRMS** (EI) *m*/*z*: Calculated for $C_9H_{17}O_3$ $[M+H]^+$ 173.11722, found: 173.11714.

8.1.4 Methyl (*R*)-3,7-dimethyloct-6-enoate (**s1**)

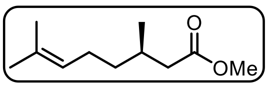

(*R*)-Citronellic acid (**42**, 800 mg, 4.70 mmol, 1.0 eq.) was dissolved in methanol (12 ml), and boron trifluoride etherate (0.60 ml, 4.70 mmol, 1.0 eq.) was added dropwise at 0 °C.[8] The reaction mixture was stirred for 18 hours at room temperature, after which the solvent was removed under reduced pressure. The residue was then dissolved in diethyl ether (20 ml) and saturated aqueous $NaHCO_3$ solution (20 ml) was added. The phases were separated, and the aqueous phase was extracted with diethyl ether (3×20 ml). The combined organic phases were dried over $MgSO_4$, filtered and the solvent removed under reduced pressure. After final purification by column chromatography (pentane/diethyl ether 50:1), methyl (*R*)-3,7-dimethyloct-6-enoate (**s1**) was obtained as a

colourless liquid (667 mg, 3.62 mmol, 77 %). The analytical data are consistent with those published.[9]

R$_f$ = 0.40 (Pe/Et_2O 20:1); $[\boldsymbol{\alpha}]_D^{25}$ = +8.40 ± 0.41 (c = 1.00, CH_2Cl_2); **^{1}H NMR** (300 MHz, $CDCl_3$): *δ* [ppm] = 5.13 – 5.05 (m, 1H, =CH), 3.67 (s, 3H, CH_3), 2.32 (dd, *J* = 14.6, 5.9 Hz, 1H, CH_aH_b), 2.12 (dd, *J* = 14.6, 8.2 Hz, 1H, CH_aH_b), 2.05 – 1.88 (m, 3H, CH, CH_2), 1.70 – 1.67 (m, 3H, CH_3), 1.62 – 1.58 (m, 3H, CH_3), 1.41 – 1.15 (m, 2H, CH_2), 0.94 (d, *J* = 6.6 Hz, 3H, CH_3); **^{13}C NMR** (75 MHz, $CDCl_3$): *δ* [ppm] = 174.1 (C_q), 132.0 (C_q), 124.7 (CH), 51.8 (CH_3), 42.0 (CH_2), 37.2 (CH_2), 30.4 (CH), 26.1 (CH_3), 25.8 (CH_2), 20.0 (CH_3), 18.0 (CH_3); **IR** (ATR, neat): $\tilde{\nu}$ [cm^{-1}] = 2960 (m), 2919 (m), 2861 (m), 1738 (s), 1442 (m), 1371 (m), 1292 (m), 1253 (m), 1192 (m), 1153 (m), 1089 (m), 1012 (m), 875 (w), 829 (m), 736 (w), 637 (w), 586 (m), 559 (w); **EI-MS** (70 eV): *m*/*z* (%) = 184 (7) $[M]^+$, 152 (42), 110 (59), 109 (36), 95 (69), 94 (27), 82 (35), 81 (21), 74 (22), 73 (18), 69 (100), 68 (20), 67 (37), 59 (37), 56 (20), 55 (47), 53 (21), 43 (20), 42 (19), 41 (97), 39 (34); **HRMS** (CIP) *m*/*z*: Calculated for $C_{11}H_{21}O_2$ $[M+H]^+$ 185.15361, found: 185.15343.

8.1.5 Methyl (*R*)-3-methyl-6-oxohexanoate (**25**)

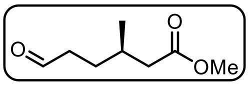

The ozonolysis was carried out analogously to procedure 8.1.3 with methyl (*R*)-3,7-dimethyloct-6-enoate (**s1**, 276 mg, 1.50 mmol, 1.0 eq), triphenylphosphine (393 mg, 1.50 mmol, 1.0 eq.) and dichloromethane (15 ml). Methyl (*R*)-3-methyl-6-oxohexanoate (**25**) was obtained as a colourless liquid (203 mg, 1.28 mmol, 86 %) and the analytical data are consistent with those published.[10]

R$_f$ = 0.30 (Pe/Et_2O 2:1); $[\boldsymbol{\alpha}]_D^{25}$ = +8.00 ± 0.41 (c = 1.00, CH_2Cl_2); **^{1}H NMR** (300 MHz, $CDCl_3$): *δ* [ppm] = 9.78 (t, *J* = 1.7 Hz, 1H, CHO), 3.68 (s, 3H, CH_3), 2.57 – 2.36 (m, 2H, CH_2), 2.32 (dd, *J* = 15.0, 6.4 Hz, 1H, CH_aH_b), 2.18 (dd, *J* = 15.0, 7.5 Hz, 1H, CH_aH_b), 2.08 – 1.91 (m, 1H, CH), 1.78 – 1.65 (m, 1H, CH_aH_b), 1.59 – 1.46 (m, 1H, CH_aH_b), 0.96 (d, *J* = 6.7 Hz, 3H, CH_3); **^{13}C NMR** (75 MHz, $CDCl_3$): *δ* [ppm] = 202.1 (CH), 173.1 (C_q), 51.5 (CH_3), 41.5 (CH_2), 41.2 (CH_2), 29.8 (CH), 28.4 (CH_2), 19.4 (CH_3); **IR** (ATR, neat): $\tilde{\nu}$ [cm^{-1}] = 2956 (w), 2723 (w), 1725 (s), 1441 (m), 1370 (m), 1256 (m), 1203 (m), 1163 (m), 1099 (m), 1009 (m), 883 (w), 839 (w), 728 (w), 671 (w), 589 (w), 552 (w); **EI-MS** (70 eV): *m*/*z* (%) = 158 (<1) $[M]^+$, 127 (31), 115 (40), 101 (86), 98 (18), 87 (90), 84 (34), 83 (30), 82 (18), 81 (56), 74 (100), 73 (75), 69 (69), 59 (89), 57 (25), 56 (21), 55 (100), 43 (88), 42 (36), 41 (85), 39 (48); **HRMS** (CIP) *m*/*z*: Calculated for $C_8H_{15}O_3$ $[M+H]^+$ 159.10157, found: 159.10155.

8.2 Synthesis of the sulfone building blocks **27** and **28**

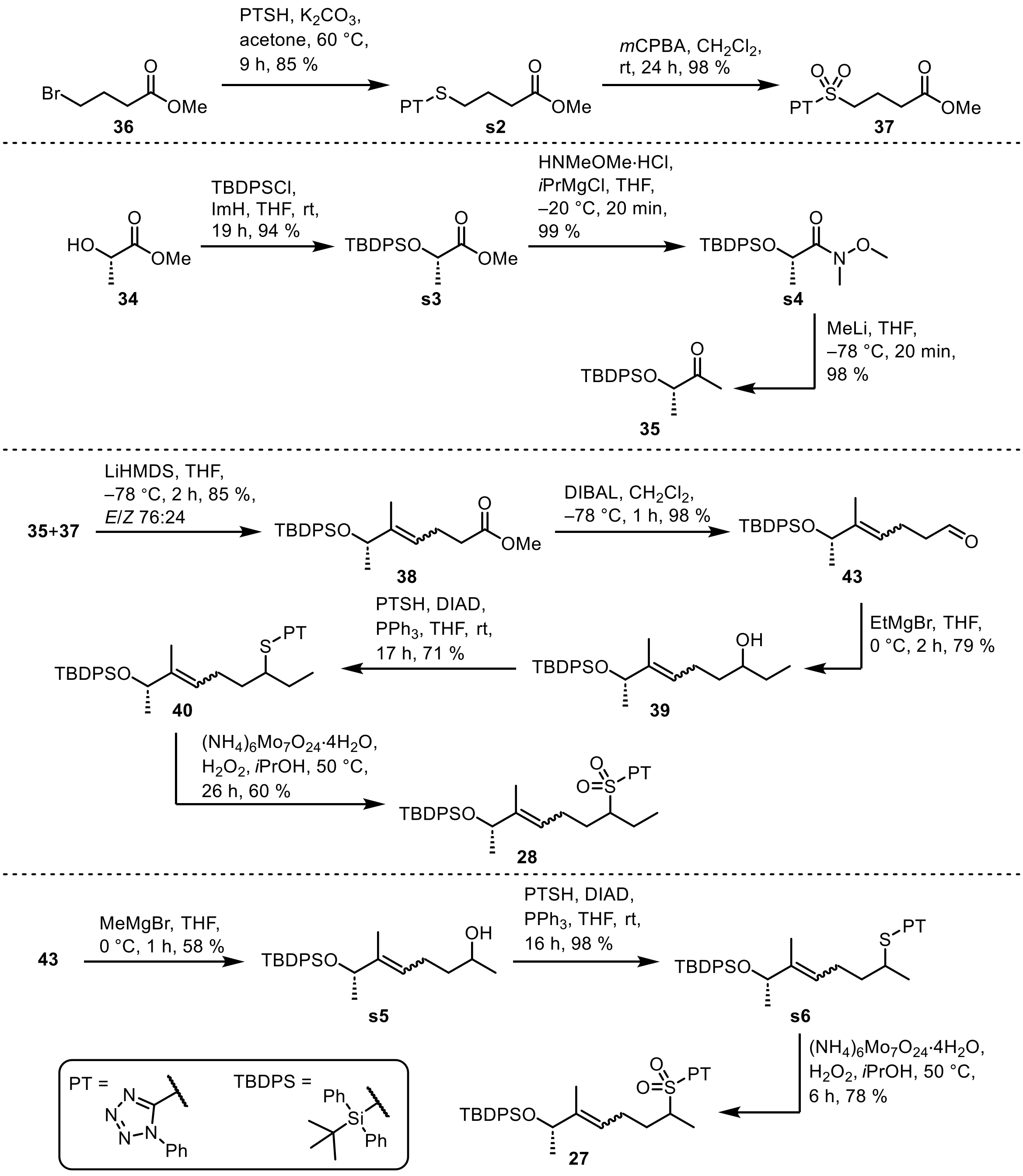

Scheme S3: Synthesis of the sulfone building blocks **27** and **28**.

8.2.1 Methyl 4-((1-phenyl-1*H*-tetrazol-5-yl)thio)butanoate (**s2**)

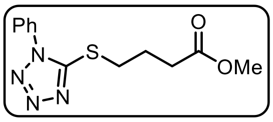

1-Phenyl-1*H*-tetrazole-5-thiol (5.35 g, 30.0 mmol, 1.0 eq.), potassium carbonate (8.30 g, 60.0 mmol, 2.0 eq.), and methyl 4-bromobutanoate (**36**, 4.92 ml, 39.0 mmol, 1.3 eq.) were placed in acetone (100 ml) and stirred at 60 °C for 9 h.[11] The mixture was then cooled to room temperature and filtered through filter paper. The solvent was removed under reduced pressure, the residue was applied to silica gel and purified by column chromatography (pentane/diethyl ether 2:1→1:1). Methyl 4-((1-phenyl-1*H*-tetrazol-5-yl)thio)butanoate (**s2**) was obtained as a colourless oil (7.14 g, 25.7 mmol, 85 %).

R$_f$ = 0.48 (Pe/Et_2O 1:1); **^{1}H NMR** (300 MHz, $CDCl_3$): δ [ppm] = 7.60 – 7.52 (m, 5H, CH_{Ar}), 3.68 (s, 3H, CH_3), 3.46 (t, *J* = 7.2 Hz, 2H, CH_2), 2.50 (t, *J* = 7.2 Hz, 2H, CH_2), 2.24 – 2.13 (m, 2H, CH_2); **^{13}C NMR** (75 MHz, $CDCl_3$): δ [ppm] = 172.9 (C_q), 154.0 (C_q), 133.6 (C_q), 130.1 (CH), 129.8 (2C, CH), 123.8 (2C, CH), 51.7 (CH_3), 32.4 (CH_2), 32.3 (CH_2), 24.3 (CH_2); **IR** (ATR, neat): $\tilde{\nu}$ [cm^{-1}] = 2950 (w), 1731 (s), 1595 (w), 1535 (w), 1498 (m), 1383 (m), 1312 (m), 1276 (m), 1207 (m), 1180 (m), 1084 (m), 1009 (m), 984 (m), 918 (w), 876 (w), 759 (s), 687 (s), 585 (w), 551 (m); **HRMS** (ESI) *m*/*z*: Calculated for $C_{12}H_{14}N_4O_2SNa$ $[M+Na]^+$ 301.07297, found: 301.07315.

8.2.2 Methyl 4-((1-phenyl-1*H*-tetrazol-5-yl)sulfonyl)butanoate (**37**)

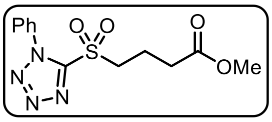

Methyl 4-((1-phenyl-1*H*-tetrazol-5-yl)thio)butanoate (**s2**, 7.06 g, 25.4 mmol, 1.0 eq.) and *m*-chloroperbenzoic acid (*m*CPBA, 70–75 %, 18.8 g, 76.2 mmol, 3.0 eq.) were placed in CH_2Cl_2 (120 ml) and stirred at room temperature for 24 h (starting material and product have similar R$_f$ values on the TLC).[12] The reaction was then quenched by adding saturated aqueous $Na_2S_2O_3$ solution (40 ml) and saturated aqueous Na_2CO_3 solution (40 ml). The phases were separated, and the aqueous phase was extracted with CH_2Cl_2 (3×100 ml). The combined organic phases were washed with saturated aqueous Na_2CO_3 solution (100 ml), dried over $MgSO_4$, filtered, and the solvent removed under reduced pressure. The residue was applied to silica gel and purified by column chromatography (pentane/diethyl ether 2:1→1:1), yielding methyl 4-((1-phenyl-1*H*-tetrazol-5-yl)sulfonyl)butanoate (**37**) as a colourless solid (7.72 g, 24.9 mmol, 98 %).

R$_f$ = 0.43 (Pe/Et_2O 1:1); m.p. 54.1–57.3 °C; **^{1}H NMR** (300 MHz, $CDCl_3$): δ [ppm] = 7.72 – 7.56 (m, 5H, CH_{Ar}), 3.89 – 3.82 (m, 2H, CH_2), 3.70 (s, 3H, CH_3), 2.58 (t, *J* = 7.0 Hz, 2H, CH_2), 2.35 – 2.24 (m, 2H, CH_2); **^{13}C NMR** (75 MHz, $CDCl_3$): δ [ppm] = 172.1 (C_q), 153.3 (C_q), 132.9 (C_q), 131.5 (CH), 129.7 (2C, CH), 125.1 (2C, CH), 54.9 (CH_2), 51.9 (CH_3), 31.6 (CH_2), 17.8 (CH_2); **IR** (ATR, neat): $\tilde{\nu}$ [cm^{-1}] = 3074 (w), 2955 (w), 1732 (s), 1586 (w), 1497 (m), 1433 (m), 1341

(s), 1298 (m), 1212 (m), 1150 (s), 1045 (w), 1009 (w), 893 (w), 842 (w), 763 (m), 690 (m), 631 (w), 560 (w); **HRMS** (ESI) *m*/*z*: Calculated for $C_{12}H_{14}N_4O_4SNa$ $[M+Na]^+$ 333.06280, found: 333.06286.

8.2.3 Methyl (*S*)-2-((*tert*-butyldiphenylsilyl)oxy)propanoate (**s3**)

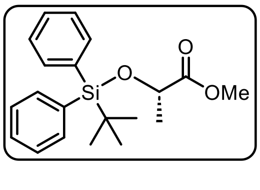

tert-Butyldiphenylchlorosilane (TBDPSCl, 5.07 ml, 19.5 mmol, 1.3 eq.) was added dropwise to a solution of methyl (*S*)-2-hydroxypropanoate (**34**, 1.43 ml, 15.0 mmol, 1.0 eq.) and imidazole (1.53 g, 22.5 mmol, 1.5 eq.) in THF (15 ml) at room temperature.[13] After stirring for 19 hours, the reaction mixture was quenched with water (20 ml) and diethyl ether (20 ml) was added. The resulting phases were separated, and the aqueous phase was extracted with diethyl ether (3×20 ml). The combined organic phases were then dried over $MgSO_4$, filtered and concentrated under reduced pressure. Finally, the residue was purified by column chromatography to give methyl (*S*)-2-((*tert*-butyldiphenylsilyl)oxy)propanoate (**s3**) as a colourless oil (4.83 g, 14.1 mmol, 94 %). The analytical data are consistent with those published.[14] (*R*)-**s3** was obtained under the same conditions by using methyl (*R*)-2-hydroxy-propanoate (**34**).

R$_f$ = 0.23 (Pe/Et_2O 20:1); $[\alpha]_D^{25}$ = –49.0 ± 0.7 (c = 1.00, CH_2Cl_2, (*S*)-**s3**); +49.9 ± 0.7 (c = 1.00, CH_2Cl_2, (*R*)-**s3**); **^{1}H NMR** (300 MHz, $CDCl_3$): *δ* [ppm] = 7.70 – 7.64 (m, 4H, CH_{Ar}), 7.47 – 7.32 (m, 6H, CH_{Ar}), 4.28 (q, *J* = 6.7 Hz, 1H, CH), 3.56 (s, 3H, CH_3), 1.37 (d, *J* = 6.7 Hz, 3H, CH_3), 1.09 (s, 9H, 3×CH_3); **^{13}C NMR** (75 MHz, $CDCl_3$): *δ* [ppm] = 174.2 (C_q), 135.9 (2C, CH), 135.7 (2C, CH), 133.5 (C_q), 133.1 (C_q), 129.8 (2C, CH), 127.6 (2C, CH), 127.6 (2C, CH), 68.9 (CH), 51.6 (CH_3), 26.8 (3C, CH_3), 21.3 (CH_3), 19.2 (C_q); **IR** (ATR, neat): $\tilde{\nu}$ [cm^{-1}] = 3061 (w), 2942 (w), 2894 (w), 2859 (w), 1750 (m), 1460 (w), 1434 (w), 1365 (w), 1274 (w), 1201 (m), 1109 (s), 1061 (m), 984 (m), 822 (w), 780 (w), 739 (m), 697 (s), 608 (m); **EI-MS** (70 eV): *m*/*z* (%) = 286 (8), 285 (35) [M–*t*Bu]$^+$, 257 (11), 215 (6), 214 (23), 213 (100), 197 (8), 184 (8), 183 (40), 182 (8), 181 (25), 180 (5), 153 (31), 135 (12), 105 (17), 57 (6), 41 (6); **HRMS** (EI) *m*/*z*: Calculated for $C_{16}H_{17}O_3Si$ [M–*t*Bu]$^+$ 285.09415, found: 285.09409.

8.2.4 (*S*)-2-((*tert*-Butyldiphenylsilyl)oxy)-*N*-methoxy-*N*-methylpropanamide (**s4**)

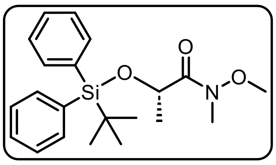

Methyl (*S*)-2-((*tert*-butyldiphenylsilyl)oxy)propanoate (**s3**, 4.74 g, 13.9 mmol, 1.00 eq.) and *N*,*O*-dimethylhydroxylamine hydrochloride (2.09 g, 21.5 mmol, 1.55 eq.) were added to THF (35 ml), and isopropylmagnesium chloride (2 M in THF, 20.8 ml, 41.6 mmol, 3.00 eq.) was added dropwise over 5 min at –20 °C.[15] The brown, clear solution was stirred

for 20 min at –20 °C, after which saturated aqueous NH_4Cl solution (30 ml) was added. Diethyl ether (30 ml) was added, the phases separated, and the aqueous phase was extracted with diethyl ether (3×30 ml). The combined organic phases were washed with saturated aqueous NaCl solution (30 ml), dried over $MgSO_4$, filtered, and the solvent was removed under reduced pressure. (*S*)-2-((*tert*-butyldiphenylsilyl)oxy)-*N*-methoxy-*N*-methylpropanamide (**s4**) was obtained as a colourless solid without further purification (5.11 g, 13.7 mmol, 99 %). The analytical data are consistent with those published.[16] (*R*)-**s4** was obtained under the same conditions by using methyl (*R*)-2-((*tert*-butyldiphenylsilyl)oxy)propanoate (**s3**).

R$_f$ = 0.38 (Pe/Et_2O 1:1); m.p. 66.5–68.1 °C; $[\alpha]_D^{25}$ = –18.8 ± 0.5 (c = 1.00, CH_2Cl_2, (*S*)-**s4**); +17.7 ± 0.5 (c = 1.00, CH_2Cl_2, (*R*)-**s4**); **^{1}H NMR** (300 MHz, $CDCl_3$): δ [ppm] = 7.74 – 7.68 (m, 4H, CH_{Ar}), 7.47 – 7.31 (m, 6H, CH_{Ar}), 4.53 (q, *J* = 6.5 Hz, 1H, CH), 3.07 (br. s, 3H, CH_3), 3.03 (s, 3H, CH_3), 1.37 (d, *J* = 6.6 Hz, 3H, CH_3), 1.09 (s, 9H, 3×CH_3); **^{13}C NMR** (75 MHz, $CDCl_3$): δ [ppm] = 174.1 (C_q), 136.0 (2C, CH), 135.7 (2C, CH), 133.8 (C_q), 133.4 (C_q), 129.6 (2C, CH), 127.6 (2C, CH), 127.5 (2C, CH), 66.7 (CH_3), 60.5 (CH), 32.3 (CH_3), 26.8 (3C, CH_3), 20.7 (CH_3), 19.2 (C_q); **IR** (ATR, neat): $\tilde{\nu}$ [cm^{-1}] = 2936 (w), 2893 (w), 2858 (w), 1666 (m), 1463 (m), 1425 (m), 1387 (w), 1346 (w), 1143 (m), 1097 (s), 1051 (m), 972 (m), 904 (m), 871 (w), 821 (m), 782 (m), 821 (m), 782 (m), 746 (m), 698 (s), 610 (s); **EI-MS** (70 eV): *m*/*z* (%) = 316 (8), 315 (28), 314 (100) [M–*t*Bu]$^+$, 294 (4), 284 (3), 254 (7), 213 (19), 199 (13), 197 (16), 183 (24), 182 (18), 181 (39), 180 (9), 135 (31), 105 (22), 104 (8), 77 (7), 57 (8); **HRMS** (EI) *m*/*z*: Calculated for $C_{17}H_{20}NO_3Si$ [M–*t*Bu]$^+$ 314.12070, found: 314.12039.

8.2.5 (*S*)-3-((*tert*-Butyldiphenylsilyl)oxy)butan-2-one (**35**)

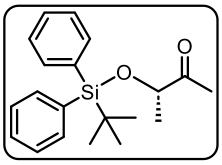

Methyllithium (1.6 M in Et_2O, 5.55 ml, 8.87 mmol, 1.2 eq.) was added dropwise over 2 min at –78 °C to a solution of (*S*)-2-((*tert*-butyldiphenylsilyl)oxy)-*N*-methoxy-*N*-methylpropanamide (**s4**, 2.75 g, 7.39 mmol, 1.0 eq.) in THF (15 ml).[17] The resulting light yellow, clear solution was stirred at this temperature for 20 min, after which the reaction was quenched by the addition of saturated aqueous NH_4Cl solution (20 ml). The cooling was removed, diethyl ether (20 ml) was added, and the phases were separated. The aqueous phase was extracted with diethyl ether (3×20 ml), and the combined organic phases were dried over $MgSO_4$, filtered, and the solvent removed under reduced pressure. (*S*)-3-((*tert*-Butyldiphenylsilyl)oxy)butan-2-one (**35**) was obtained as a colourless oil without further purification (2.37 g, 7.24 mmol, 98 %). The analytical data are consistent with those published.[18] (*R*)-**35** was obtained under the same conditions by using (*R*)-2-((*tert*-butyldiphenylsilyl)oxy)-*N*-methoxy-*N*-methylpropan-amide (**s4**).

R$_f$ = 0.24 (Pe/Et_2O 20:1); $[\alpha]_D^{25}$ = –3.90 ± 0.40 (c = 1.00, CH_2Cl_2, (*S*)-**35**); +3.90 ± 0.40 (c = 1.00, CH_2Cl_2, (*R*)-**35**); **^{1}H NMR** (300 MHz, $CDCl_3$): *δ* [ppm] = 7.68 – 7.60 (m, 4H, CH_{Ar}), 7.47 – 7.33 (m, 6H, CH_{Ar}), 4.17 (q, *J* = 6.8 Hz, 1H, CH), 2.17 (s, 3H, CH_3), 1.19 (d, *J* = 6.8 Hz, 3H, CH_3), 1.11 (s, 9H, 3×CH_3); **^{13}C NMR** (75 MHz, $CDCl_3$): *δ* [ppm] = 211.8 (C_q), 135.7 (4C, CH), 133.6 (C_q), 132.9 (C_q), 130.0 (CH), 129.9 (CH), 127.8 (2C, CH), 127.7 (2C, CH), 75.7 (CH), 26.9 (3C, CH_3), 25.0 (CH_3), 20.6 (CH_3), 19.2 (C_q); **IR** (ATR, neat): $\tilde{\nu}$ [cm^{-1}] = 3062 (w), 2938 (w), 2893 (w), 2860 (w), 1721 (m), 1463 (w), 1426 (m), 1357 (m), 1229 (w), 1192 (w), 1107 (s), 1057 (m), 997 (m), 927 (m), 859 (w), 822 (m), 786 (m), 740 (m), 696 (s), 610 (m); **EI-MS** (70 eV): *m*/*z* (%) = 283 (4), 270 (16), 269 (68) [M–*t*Bu]$^+$, 199 (10), 197 (20), 192 (17), 191 (100), 183 (11), 182 (11), 181 (31), 180 (8), 161 (7), 139 (31), 135 (45), 105 (26), 77 (12), 43 (16); **HRMS** (EI) *m*/*z*: Calculated for $C_{16}H_{17}O_2Si$ [M–*t*Bu]$^+$ 269.09923, found: 269.09888.

8.2.6 Methyl (*S*)-6-((*tert*-butyldiphenylsilyl)oxy)-5-methylhept-4-enoate (**38**)

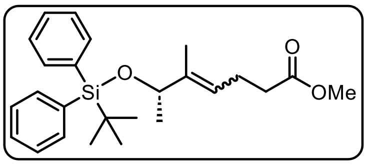

Methyl 4-((1-phenyl-1*H*-tetrazol-5-yl)sulfonyl)butanoate (**37**, 2.05 g, 6.62 mmol, 1.2 eq.) was dissolved in THF (degassed using the freeze-pump-thaw method, 12 ml), and lithium bis(trimethylsilyl)amide (LiHMDS, 1 M in THF, 7.17 ml, 7.17 mmol, 1.3 eq.) was added dropwise at –78 °C over 10 min.[19] The yellow solution was then stirred at this temperature for 30 min, and then (*S*)-3-((*tert*-butyldiphenylsilyl) oxy)butan-2-one (**35**, 1.80 g, 5.51 mmol, 1.0 eq.) in THF (2 ml) was added dropwise at –78 °C over 10 min, causing the solution to decolorize slightly. The reaction mixture was stirred at –78 °C for 2 h and then immediately warmed to room temperature. The reaction was quenched by adding saturated aqueous NH_4Cl solution (20 ml), diethyl ether (20 ml) was added, the phases were separated, and the aqueous phase was extracted with diethyl ether (3×20 ml). The combined organic phases were washed with saturated aqueous NaCl solution (30 ml), dried over $MgSO_4$, filtered, and the solvent removed under reduced pressure. The residue was applied to silica gel, and purification by column chromatography (pentane/diethyl ether 20:1→10:1) yielded methyl (*S*)-6-((*tert*-butyldiphenylsilyl)oxy)-5-methylhept-4-enoate (**38**) as a colourless oil (1.91 g, 4.66 mmol, 85 %) in an *E*/*Z* ratio of 76:24. (*R*)-**38** was obtained under the same conditions by using (*R*)-3-((*tert*-butyldiphenylsilyl) oxy)butan-2-one (**35**).

R$_f$ = (*E*/*Z*): 0.20 (Pe/Et_2O 20:1); ***E* isomer**: **^{1}H NMR** (300 MHz, $CDCl_3$): *δ* [ppm] = 7.71 – 7.59 (m, 4H, CH_{Ar}), 7.45 – 7.29 (m, 6H, CH_{Ar}), 5.16 – 5.06 (m, 1H, =CH), 4.21 – 4.12 (m, 1H, CH), 3.64 (s, 3H, CH_3), 2.28 – 2.20 (m, 4H, 2×CH_2), 1.61 (d, *J* = 1.3 Hz, 3H, CH_3), 1.12 (d, *J* = 6.3 Hz, 3H, CH_3), 1.04 (s, 9H, 3×CH_3); **^{13}C NMR** (75 MHz, $CDCl_3$): *δ* [ppm] = 173.7 (C_q), 139.9 (C_q), 135.9 (2C, CH), 135.8 (2C, CH), 134.7 (C_q), 134.2 (C_q), 129.4 (CH), 129.4 (CH), 127.4 (2C, CH), 127.3 (2C, CH), 122.1 (CH), 74.6 (CH), 51.5 (CH_3), 33.9 (CH_2), 27.0 (3C, CH_3), 23.0

(CH_3), 23.0 (CH_2), 19.3 (C_q), 11.3 (CH_3); **IR** (GC/FTIR): $\tilde{\nu}$ [cm^{-1}] = 3073 (w), 2957 (m), 2932 (m), 2895 (m), 2858 (m), 1740 (s), 1590 (w), 1473 (m), 1429 (m), 1390 (w), 1362 (m), 1261 (w), 1195 (m), 1156 (m), 1113 (s), 1081 (s), 1055 (m), 983 (m), 941 (w), 889 (w), 825 (m), 780 (w), 743 (m), 703 (s); **EI-MS** (70 eV): *m*/*z* (%) = 354 (4), 353 (13) [M–*t*Bu]$^+$, 321 (3), 275 (17), 213 (29), 200 (19), 199 (100), 197 (17), 183 (14), 181 (17), 155 (13), 153 (16), 135 (24), 105 (13), 95 (38), 81 (25), 79 (12), 77 (31), 67 (13), 41 (18); **HRMS** (EI) *m*/*z*: Calculated for $C_{21}H_{25}O_3Si$ [M–*t*Bu]$^+$ 353.15675, found: 353.15677; ***Z* isomer**: **^{1}H NMR** (300 MHz, $CDCl_3$): δ [ppm] = 7.71 – 7.59 (m, 4H, CH_{Ar}), 7.45 – 7.29 (m, 6H, CH_{Ar}), 4.97 – 4.88 (m, 1H, =CH), 4.73 – 4.64 (m, 1H, CH), 3.61 (s, 3H, CH_3), 2.13 – 2.06 (m, 2H, CH_2), 2.06 – 1.83 (m, 2H, CH_2), 1.79 – 1.76 (m, 3H, CH_3), 1.13 (d, *J* = 6.4 Hz, 3H, CH_3), 1.05 (s, 9H, 3×CH_3); **^{13}C NMR** (75 MHz, $CDCl_3$): δ [ppm] = 173.5 (C_q), 140.0 (C_q), 135.8 (2C, CH), 135.8 (2C, CH), 134.6 (C_q), 134.1 (C_q), 129.5 (CH), 129.5 (CH), 127.5 (2C, CH), 127.4 (2C, CH), 122.2 (CH), 67.3 (CH), 51.4 (CH_3), 34.0 (CH_2), 26.9 (3C, CH_3), 22.5 (CH_3), 22.5 (CH_2), 19.3 (C_q), 17.6 (CH_3); **IR** (GC/FTIR): $\tilde{\nu}$ [cm^{-1}] = 3074 (w), 3053 (w), 2969 (m), 2934 (m), 2893 (m), 2858 (m), 1741 (s), 1591 (w), 1474 (m), 1429 (m), 1365 (m), 1261 (m), 1195 (m), 1157 (m), 1112 (s), 1078 (s), 1045 (m), 998 (m), 983 (m), 944 (w), 825 (m), 805 (w), 742 (m), 703 (s); **EI-MS** (70 eV): *m*/*z* (%) = 354 (12), 353 (44) [M–*t*Bu]$^+$, 321 (7), 301 (4), 213 (26), 200 (18), 199 (100), 197 (20), 183 (16), 153 (21), 135 (21), 123 (12), 105 (14), 95 (47), 81 (16), 77 (27), 67 (14), 55 (11), 41 (18); **HRMS** (EI) *m*/*z*: Calculated for $C_{21}H_{25}O_3Si$ [M–*t*Bu]$^+$ 353.15675, found: 353.15674.

8.2.7 (*S*)-6-((*tert*-Butyldiphenylsilyl)oxy)-5-methylhept-4-enal (**43**)

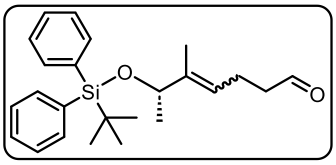

Methyl (*S*)-6-((*tert*-butyldiphenylsilyl)oxy)-5-methylhept-4-enoate (**38**, 1.42 g, 3.45 mmol, 1.0 eq.) was dissolved in CH_2Cl_2 (14 ml), and diisobutylaluminium hydride (DIBAL, 1 M in hexanes, 3.80 ml, 3.80 mmol, 1.1 eq.) was added dropwise over 10 min at –78 °C.[20] After stirring at –78 °C for one hour, the cooling bath was immediately removed and saturated aqueous Rochelle salt solution (20 ml) was slowly added. Stirring continued at room temperature for one hour, and then the phases were separated. The aqueous phase was extracted with CH_2Cl_2 (3×20 ml), and the combined organic phases were washed with saturated aqueous NaCl solution (30 ml), dried over $MgSO_4$, filtered, and the solvent was removed under reduced pressure. (*S*)-6-((*tert*-Butyldiphenylsilyl)oxy)-5-methylhept-4-enal (**43**) was obtained as a colourless oil without further purification (1.28 g, 3.37 mmol, 98 %). (*R*)-**43** was obtained under the same conditions by using methyl (*R*)-6-((*tert*-butyldiphenylsilyl)oxy)-5-methylhept-4-enoate (**38**).

R$_f$ = (*E*/*Z*): 0.16 (Pe/Et_2O 20:1); ***E* isomer**: **^{1}H NMR** (300 MHz, $CDCl_3$): δ [ppm] = 9.70 (t, *J* = 1.6 Hz, 1H, CHO), 7.71 – 7.59 (m, 4H, CH_{Ar}), 7.45 – 7.30 (m, 6H, CH_{Ar}), 5.13 – 5.05 (m, 1H,

=CH), 4.16 (q, J = 6.2 Hz, 1H, CH), 2.38 – 1.81 (m, 4H, 2×CH_2), 1.63 – 1.59 (m, 3H, CH_3), 1.14 (d, J = 6.3 Hz, 3H, CH_3), 1.04 (s, 9H, 3×CH_3); **^{13}C NMR** (75 MHz, $CDCl_3$): δ [ppm] = 202.3 (CH), 140.0 (C_q), 135.9 (2C, CH), 135.8 (2C, CH), 134.6 (C_q), 134.2 (C_q), 129.5 (CH), 129.4 (CH), 127.4 (2C, CH), 127.3 (2C, CH), 121.9 (CH), 74.6 (CH), 43.6 (CH_2), 27.0 (3C, CH_3), 23.0 (CH_3), 20.2 (CH_2), 19.3 (C_q), 11.3 (CH_3); **IR** (GC/FTIR): $\tilde{\nu}$ [cm^{-1}] = 3073 (w), 2964 (m), 2932 (m), 2891 (m), 2857 (m), 2723 (w), 1724 (s), 1591 (w), 1474 (m), 1463 (m), 1428 (m), 1390 (m), 1363 (m), 1313 (w), 1262 (w), 1191 (w), 1112 (s), 1083 (s), 1062 (s), 985 (m), 939 (w), 825 (m), 792 (w), 743 (m), 703 (s); **EI-MS** (70 eV): m/z (%) = 323 (30) [M–*t*Bu]$^+$, 267 (17), 239 (9), 226 (11), 225 (55), 201 (11), 200 (17), 199 (100), 197 (16), 183 (25), 181 (19), 139 (15), 135 (16), 107 (9), 105 (11), 81 (9), 77 (21), 55 (10), 41 (14); **HRMS** (EI) m/z: Calculated for $C_{20}H_{23}O_2Si$ [M–*t*Bu]$^+$ 323.14618, found: 323.14618; ***Z* isomer**: **^{1}H NMR** (300 MHz, $CDCl_3$): δ [ppm] = 9.56 (t, J = 1.6 Hz, 1H, CHO), 7.71 – 7.59 (m, 4H, CH_{Ar}), 7.45 – 7.30 (m, 6H, CH_{Ar}), 4.94 – 4.87 (m, 1H, =CH), 4.71 – 4.63 (m, 1H, CH), 2.38 – 1.81 (m, 4H, 2×CH_2), 1.79 – 1.76 (m, 3H, CH_3), 1.14 (d, J = 6.4 Hz, 3H, CH_3), 1.04 (s, 9H, 3×CH_3); **^{13}C NMR** (75 MHz, $CDCl_3$): δ [ppm] = 202.2 (CH), 140.2 (C_q), 135.9 (2C, CH), 135.8 (2C, CH), 134.6 (C_q), 134.2 (C_q), 129.6 (CH), 129.5 (CH), 127.5 (2C, CH), 127.4 (2C, CH), 121.9 (CH), 67.2 (CH), 43.7 (CH_2), 26.9 (3C, CH_3), 22.5 (CH_3), 19.7 (CH_2), 19.3 (C_q), 17.6 (CH_3); **IR** (GC/FTIR): $\tilde{\nu}$ [cm^{-1}] = 3074 (w), 3049 (w), 2969 (m), 2933 (m), 2892 (m), 2858 (m), 2821 (w), 2724 (w), 1726 (s), 1591 (w), 1474 (m), 1429 (m), 1408 (w), 1389 (w), 1263 (w), 1190 (w), 1112 (s), 1078 (s), 1045 (m), 996 (m), 943 (w), 825 (m), 796 (w), 743 (m), 704 (s); **EI-MS** (70 eV): m/z (%) = 324 (11), 323 (38) [M–*t*Bu]$^+$, 225 (29), 207 (12), 201 (11), 200 (18), 199 (100), 197 (17), 183 (36), 181 (20), 139 (19), 135 (17), 105 (14), 77 (28), 57 (10), 55 (12), 41 (16); **HRMS** (EI) m/z: Calculated for $C_{20}H_{23}O_2Si$ [M–*t*Bu]$^+$ 323.14618, found: 323.14636.

8.2.8 (8*S*)-8-((*tert*-Butyldiphenylsilyl)oxy)-7-methylnon-6-en-3-ol (**39**)

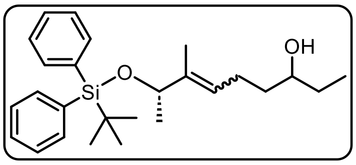

(*S*)-6-((*tert*-Butyldiphenylsilyl)oxy)-5-methylhept-4-enal (**43**, 1.28 g, 3.36 mmol, 1.0 eq.) was dissolved in THF (7 ml), and ethylmagnesium bromide (3 M in diethyl ether, 1.35 ml, 4.04 mmol, 1.2 eq.) was added dropwise over 5 min at 0 °C.[21] After stirring for two hours at 0 °C, the reaction mixture was quenched by adding saturated aqueous NH_4Cl solution (20 ml). Subsequently, diethyl ether (20 ml) was added, the phases were separated, and the aqueous phase was extracted with diethyl ether (3×20 ml). The combined organic phases were dried over $MgSO_4$, filtered, and the solvent removed under reduced pressure. The crude product was purified by column chromatography (pentane/diethyl ether 5:1→2:1) to afford (8*S*)-8-((*tert*-butyldiphenylsilyl)oxy)-7-methylnon-6-en-3-ol (**39**) as a colourless oil in a diastereomeric (3*S*,8*S* : 3*R*,8*S*) ratio of 1:1 (1.10 g, 2.67 mmol, 79 %). (*R*)-

39 was obtained under the same conditions by using (*R*)-6-((*tert*-butyldiphenylsilyl)oxy)-5-methylhept-4-enal (**43**).

$\mathbf{R_f}$ = (*E*/*Z*): 0.40 (Pe/Et_2O 2:1); ***E* isomer**: **^{1}H NMR** (300 MHz, $CDCl_3$): δ [ppm] = 7.74 – 7.60 (m, 4H, CH_{Ar}), 7.45 – 7.29 (m, 6H, CH_{Ar}), 5.20 – 5.09 (m, 1H, =CH), 4.18 (q, *J* = 6.3 Hz, 1H, CH), 3.53 – 3.39 (m, 1H, CH), 2.14 – 1.89 (m, 2H, CH_2), 1.64 – 1.60 (m, 3H, CH_3), 1.60 – 1.16 (m, 5H, 2×CH_2, OH), 1.14/1.13 (d, *J* = 6.3/6.3 Hz, 3H, CH_3), 1.05 (s, 9H, 3×CH_3), 0.93/0.92 (t, *J* = 7.5/7.4 Hz, 3H, CH_3); **^{13}C NMR** (75 MHz, $CDCl_3$): δ [ppm] = 138.8 (C_q), 135.9 (2C, CH), 135.8 (2C, CH), 134.8/134.7 (C_q), 134.4/134.3 (C_q), 129.4 (CH), 129.4 (CH), 127.4 (2C, CH), 127.3 (2C, CH), 124.0/123.9 (CH), 74.9/74.8 (CH), 72.9/72.9 (CH), 36.5/36.5 (CH_2), 30.2 (CH_2), 27.0 (3C, CH_3), 23.7/23.6 (CH_2), 23.1/23.1 (CH_3), 19.3 (C_q), 11.3/11.2 (CH_3), 9.9 (CH_3); **IR** (GC/FTIR): $\tilde{\nu}$ [cm^{-1}] = 3303 (br. s), 3073 (w), 3050 (w), 2963 (s), 2932 (s), 2858 (s), 1591 (w), 1473 (m), 1462 (m), 1428 (m), 1389 (w), 1363 (w), 1334 (w), 1112 (s), 1075 (s), 1043 (m), 985 (m), 940 (m), 824 (m), 777 (w), 740 (m), 703 (s); **EI-MS** (70 eV): *m*/*z* (%) = 353 (2) [M–*t*Bu]$^+$, 201 (6), 200 (19), 199 (100), 197 (7), 181 (8), 139 (21), 137 (14), 135 (11), 95 (10), 81 (16), 78 (5), 77 (15), 69 (6), 67 (6), 57 (9), 55 (10), 45 (6), 41 (11); **HRMS** (CIP) *m*/*z*: Calculated for $C_{26}H_{37}O_2Si$ [M–H]$^+$ 409.25573, found: 409.25543; ***Z* isomer**: **^{1}H NMR** (300 MHz, $CDCl_3$): δ [ppm] = 7.74 – 7.60 (m, 4H, CH_{Ar}), 7.45 – 7.29 (m, 6H, CH_{Ar}), 5.03 – 4.94 (m, 1H, =CH), 4.76 – 4.66 (m, 1H, CH), 3.39 – 3.23 (m, 1H, CH), 1.82 – 1.77 (m, 3H, CH_3), 1.78 – 1.63 (m, 2H, CH_2), 1.60 – 1.16 (m, 5H, 2×CH_2, OH), 1.13/1.12 (d, *J* = 6.4/6.4 Hz, 3H, CH_3), 1.05 (s, 9H, 3×CH_3), 0.86/0.85 (t, *J* = 7.4/7.4 Hz, 3H, CH_3); **^{13}C NMR** (75 MHz, $CDCl_3$): δ [ppm] = 139.0/139.0 (C_q), 135.8 (4C, CH), 134.7/134.6 (C_q), 134.3/134.2 (C_q), 129.6/129.5 (CH), 129.5/129.4 (CH), 127.7/127.5 (2C, CH), 127.5/127.4 (2C, CH), 124.1/124.0 (CH), 72.8/72.6 (CH), 67.3 (CH), 36.9/36.8 (CH_2), 30.0 (CH_2), 26.9 (3C, CH_3), 23.2/23.2 (CH_2), 22.6/22.5 (CH_3), 19.3 (C_q), 17.5 (CH_3), 9.8/9.8 (CH_3); **IR** (GC/FTIR): $\tilde{\nu}$ [cm^{-1}] = 3292 (br. s), 3072 (w), 3049 (w), 2964 (s), 2931 (s), 2858 (m), 1592 (w), 1474 (w), 1463 (w), 1429 (m), 1379 (w), 1365 (w), 1263 (w), 1192 (w), 1115 (s), 1080 (s), 1045 (m), 986 (m), 945 (w), 826 (m), 805 (w), 742 (m), 703 (s); **EI-MS** (70 eV): *m*/*z* (%) = 353 (3) [M–*t*Bu]$^+$, 200 (18), 199 (100), 197 (8), 181 (8), 139 (19), 137 (25), 135 (11), 95 (25), 81 (21), 78 (6), 77 (16), 69 (8), 67 (7), 57 (12), 55 (12), 45 (6), 43 (6), 41 (13); **HRMS** (CIP) *m*/*z*: Calculated for $C_{26}H_{37}O_2Si$ [M–H]$^+$ 409.25573, found: 409.25565.

8.2.9 5-(((8*S*)-8-((*tert*-Butyldiphenylsilyl)oxy)-7-methylnon-6-en-3-yl)thio)-1-phenyl-1*H*-tetrazole (**40**)

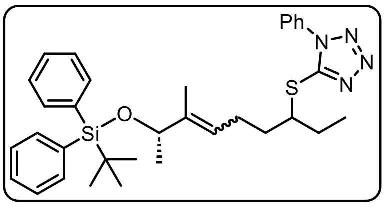

Diisopropyl azodicarboxylate (DIAD, 0.80 ml, 4.06 mmol, 1.5 eq.) was added dropwise to a solution of (8*S*)-8-((*tert*-butyldiphenylsilyl)oxy)-7-methylnon-6-en-3-ol (**39**, 1.11 g, 2.71 mmol, 1.0 eq.), triphenylphosphine (1.07 g, 4.06 mmol, 1.5 eq.), and 1-phenyl-1*H*-tetrazole-5-thiol (724 mg, 4.06 mmol, 1.5 eq.) in THF (11 ml) at 0 °C.[22] The reaction mixture was stirred at room temperature for 17 h, and after completion of the reaction, the solvent was removed under reduced pressure. Finally, the residue was applied to silica gel and purified by column chromatography (pentane/diethyl ether 20:1→10:1) yielding 5-(((8*S*)-8-((*tert*-butyldiphenylsilyl)oxy)-7-methylnon-6-en-3-yl)thio)-1-phenyl-1*H*-tetrazole (**40**) as a colourless oil (1.10 g, 1.93 mmol, 71 %). (*R*)-**40** was obtained under the same conditions by using (8*R*)-8-((*tert*-butyldiphenylsilyl)oxy)-7-methylnon-6-en-3-ol (**39**).

R$_f$ = (*E*/*Z*): 0.44 (Pe/Et_2O 5:1); ***E* isomer**: **^{1}H NMR** (300 MHz, $CDCl_3$): δ [ppm] = 7.70 – 7.51 (m, 9H, CH_{Ar}), 7.45 – 7.26 (m, 6H, CH_{Ar}), 5.12 – 5.04 (m, 1H, =CH), 4.16 (q, *J* = 6.3 Hz, 1H, CH), 3.92/3.91 (quin, *J* = 6.5/6.5 Hz, 1H, CH), 2.11 – 1.99 (m, 2H, CH_2), 1.91 – 1.61 (m, 4H, 2×CH_2), 1.58 – 1.54 (m, 3H, CH_3), 1.12/1.12 (d, *J* = 6.3/6.3 Hz, 3H, CH_3), 1.04 (s, 9H, 3×CH_3), 1.00/1.00 (t, *J* = 7.4/7.4 Hz, 3H, CH_3); **^{13}C NMR** (75 MHz, $CDCl_3$): δ [ppm] = 154.3/154.2 (C_q), 139.5/139.4 (C_q), 135.9 (2C, CH), 135.8 (2C, CH), 134.7 (C_q), 134.3/134.3 (C_q), 133.8 (C_q), 130.0 (CH), 129.7 (2C, CH), 129.4 (CH), 129.4/129.4 (CH), 127.4 (2C, CH), 127.3/127.3 (2C, CH), 124.1 (2C, CH), 122.8 (CH), 74.7/74.7 (CH), 51.6/51.6 (CH), 33.6/33.6 (CH_2), 27.7/27.5 (CH_2), 27.0 (3C, CH_3), 24.8/24.7 (CH_2), 23.1/23.0 (CH_3), 19.3 (C_q), 11.3/11.3 (CH_3), 11.0 (CH_3); **IR** (ATR, neat): $\tilde{\nu}$ [cm^{-1}] = (*E*/*Z*): 3060 (w), 2963 (w), 2858 (w), 1594 (w), 1499 (w), 1459 (w), 1420 (w), 1386 (m), 1318 (w), 1237 (w), 1075 (m), 980 (m), 820 (w), 748 (m), 695 (s), 610 (m), 555 (w); **HRMS** (ESI) *m*/*z*: (*E*/*Z*): Calculated for $C_{33}H_{42}N_4OSSiNa$ $[M+Na]^+$ 593.27408, found: 593.27426; ***Z* isomer**: **^{1}H NMR** (300 MHz, $CDCl_3$): δ [ppm] = 7.70 – 7.51 (m, 9H, CH_{Ar}), 7.45 – 7.26 (m, 6H, CH_{Ar}), 4.99 – 4.90 (m, 1H, =CH), 4.68 – 4.58 (m, 1H, CH), 3.86 – 3.73 (m, 1H, CH), 1.91 – 1.61 (m, 6H, 3×CH_2), 1.79 – 1.76 (m, 3H, CH_3), 1.11/1.08 (d, *J* = 6.3/6.4 Hz, 3H, CH_3), 1.03 (s, 9H, 3×CH_3), 0.91/0.90 (t, *J* = 7.3/7.4 Hz, 3H, CH_3); **^{13}C NMR** (75 MHz, $CDCl_3$): δ [ppm] = 154.2/154.2 (C_q), 139.7/139.6 (C_q), 135.8/135.7 (4C, CH), 134.6 (C_q), 134.1 (C_q), 133.8 (C_q), 123.0 (CH), 129.7 (2C, CH), 129.5/129.5 (CH), 129.4 (CH), 127.5/127.5 (2C, CH), 127.4 (2C, CH), 124.0/124.0 (2C, CH), 122.9/122.9 (CH), 67.3/67.3 (CH), 51.4/51.2 (CH), 33.9/33.6 (CH_2), 27.4/27.3 (CH_2), 26.9 (3C, CH_3), 24.3/24.2 (CH_2), 22.5/22.5 (CH_3), 19.3 (C_q), 17.6/17.5 (CH_3), 10.8 (CH_3).

8.2.10 5-(((8*S*)-8-((*tert*-Butyldiphenylsilyl)oxy)-7-methylnon-6-en-3-yl)sulfonyl)-1-phenyl-1*H*-tetrazole (**28**)

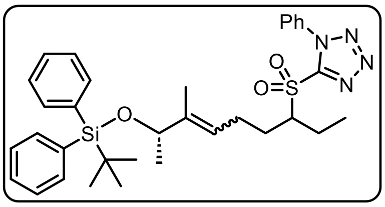

Hydrogen peroxide (30 wt% in water, 1.12 ml, 11.0 mmol, 5.00 eq.) was added dropwise to ammonium heptamolybdate tetrahydrate (135 mg, 0.110 mmol, 0.05 eq.) at 0 °C.[19] The neon-yellow solution was stirred for 15 min at 0 °C and then added to a solution of 5-(((8*S*)-8-((*tert*-butyldiphenylsilyl)oxy)-7-methylnon-6-en-3-yl)thio)-1-phenyl-1*H*-tetrazole (**40**, 1.25 g, 2.19 mmol, 1.00 eq.) in isopropanol (11 ml) at 0 °C, resulting in clouding. The reaction mixture was then heated to 50 °C and stirred for 26 h (starting material and product have similar R_f values on the TLC). Saturated aqueous $Na_2S_2O_3$ solution (30 ml) and diethyl ether (30 ml) were then added, the phases were separated, and the aqueous phase was extracted with diethyl ether (3×30 ml). The combined organic phases were dried over $MgSO_4$, filtered, and the solvent was removed under reduced pressure. The residue was applied to silica gel, purified by column chromatography (pentane/diethyl ether 20:1→5:1), and 5-(((8*S*)-8-((*tert*-butyldiphenylsilyl)oxy)-7-methylnon-6-en-3-yl)sulfonyl)-1-phenyl-1*H*-tetrazole (**28**) was obtained as a colourless, viscous oil (796 mg, 1.32 mmol, 60 %). (*R*)-**28** was obtained under the same conditions by using 5-(((8*R*)-8-((*tert*-butyldiphenylsilyl)oxy)-7-methylnon-6-en-3-yl)thio)-1-phenyl-1*H*-tetrazole (**40**).

$\mathbf{R_f}$ = (*E*/*Z*): 0.48 (Pe/Et_2O 5:1); ***E* isomer**: **^{1}H NMR** (500 MHz, $CDCl_3$): *δ* [ppm] = 7.71 – 7.52 (m, 9H, CH_{Ar}), 7.46 – 7.27 (m, 6H, CH_{Ar}), 5.10 – 5.02 (m, 1H, =CH), 4.21 – 4.14 (m, 1H, CH), 3.75 – 3.66 (m, 1H, CH), 2.17 – 1.63 (m, 6H, 3×CH_2), 1.61 – 1.57 (m, 3H, CH_3), 1.14 (d, *J* = 6.3 Hz, 3H, CH_3), 1.06/1.06 (t, *J* = 7.4/7.5 Hz, 3H, CH_3), 1.05/1.04 (s, 9H, 3×CH_3); **^{13}C NMR** (125 MHz, $CDCl_3$): *δ* [ppm] = 153.3/153.2 (C_q), 140.7/140.6 (C_q), 135.8 (2C, CH), 135.8 (2C, CH), 134.6/134.6 (C_q), 134.2/134.2 (C_q), 133.2 (C_q), 131.4 (CH), 129.6 (2C, CH), 129.5/129.5 (2C, CH), 127.4 (2C, CH), 127.4 (2C, CH), 125.4 (2C, CH), 121.6/ 121.6 (CH), 74.6/74.5 (CH), 65.9/65.8 (CH), 27.0 (3C, CH_3), 26.3/26.3 (CH_2), 24.4/24.4 (CH_2), 23.1 (CH_3), 20.8/20.8 (CH_2), 19.3 (C_q), 11.4/11.4 (CH_3), 10.8/10.8 (CH_3); **IR** (ATR, neat): $\tilde{\nu}$ [cm^{-1}] = (*E*/*Z*): 3060 (w), 2935 (m), 2860 (m), 1594 (w), 1461 (m), 1431 (m), 1337 (m), 1149 (m), 1105 (m), 984 (m), 823 (w), 754 (m), 699 (s), 625 (m), 592 (m), 561 (m); **HRMS** (ESI) *m*/*z*: (*E*/*Z*): Calculated for $C_{33}H_{42}N_4O_3SSiNa$ $[M+Na]^+$ 625.26391, found: 625.26405; ***Z* isomer**: **^{1}H NMR** (500 MHz, $CDCl_3$): *δ* [ppm] = 7.71 – 7.52 (m, 9H, CH_{Ar}), 7.46 – 7.27 (m, 6H, CH_{Ar}), 4.95 – 4.88 (m, 1H, =CH), 4.65 – 4.58 (m, 1H, CH), 3.61 – 3.52 (m, 1H, CH), 2.17 – 1.63 (m, 6H, 3×CH_2), 1.81 – 1.78 (m, 3H, CH_3), 1.12/1.12 (d, *J* = 6.2/6.2 Hz, 3H, CH_3), 1.04/1.04 (s, 9H, 3×CH_3), 0.91/0.89 (t, *J* = 7.5/7.5 Hz, 3H, CH_3); **^{13}C NMR** (125 MHz, $CDCl_3$): *δ* [ppm] = 153.3/153.2 (C_q), 140.9/140.6 (C_q), 135.8 (2C, CH), 135.8/135.8 (2C, CH), 134.5 (C_q), 134.0 (C_q), 133.2 (C_q), 131.4 (CH), 129.6/129.6 (4C, CH), 127.7 (CH), 127.5 (2C, CH), 127.5 (CH), 125.4 (2C, CH),

121.9/121.8 (CH), 67.3/67.2 (CH), 65.8/65.6 (CH), 26.9 (3C, CH_3), 26.6/26.5 (CH_2), 24.1/23.8 (CH_3), 22.6/22.5 (CH_2), 20.5/20.4 (CH_2), 19.3 (C_q), 17.6/17.6 (CH_3), 10.7/10.5 (CH_3).

8.2.11 (7*S*)-7-((*tert*-Butyldiphenylsilyl)oxy)-6-methyloct-5-en-2-ol (**s5**)

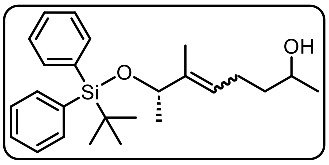

The Grignard reaction was carried out analogously to procedure 8.2.8 with (*S*)-6-((*tert*-butyldiphenylsilyl)oxy)-5-methylhept-4-enal (**43**, 2.20 g, 5.78 mmol, 1.0 eq.), methylmagnesium bromide (3 M in diethyl ether, 2.10 ml, 6.36 mmol, 1.1 eq.) and THF (20 ml). The reaction mixture was stirred for only 1 h and (7*S*)-7-((*tert*-butyldiphenylsilyl)oxy)-6-methyloct-5-en-2-ol (**s5**) was obtained as a colourless oil in a diastereomeric (2*S*,7*S* : 2*R*,7*S*) ratio of 1:1 (1.34 g, 3.38 mmol, 58 %).

R_f = (*E*/*Z*): 0.21 (Pe/Et_2O 2:1); ***E* isomer**: **^{1}H NMR** (300 MHz, $CDCl_3$): δ [ppm] = 7.72 – 7.60 (m, 4H, CH_{Ar}), 7.46 – 7.29 (m, 6H, CH_{Ar}), 5.19 – 5.09 (m, 1H, =CH), 4.18 (q, *J* = 6.3 Hz, 1H, CH), 3.80 – 3.65 (m, 1H, CH), 2.11 – 1.89 (m, 2H, CH_2), 1.64 – 1.60 (m, 3H, CH_3), 1.51 – 1.19 (m, 3H, CH_2, OH), 1.17/1.16 (d, *J* = 6.2/6.2 Hz, 3H, CH_3), 1.14/1.13 (d, *J* = 6.3/6.3 Hz, 3H, CH_3), 1.05 (s, 9H, 3×CH_3); **^{13}C NMR** (75 MHz, $CDCl_3$): δ [ppm] = 138.8 (C_q), 135.8 (4C, CH), 134.7/134.7 (C_q), 134.4/134.3 (C_q), 129.4 (CH), 129.4 (CH), 127.4 (2C, CH), 127.3 (2C, CH), 123.8/123.7 (CH), 74.8/74.8 (CH), 67.8/67.7 (CH), 38.9/38.8 (CH_2), 27.0 (3C, CH_3), 23.8/23.7 (CH_2), 23.5 (CH_3), 23.1/23.1 (CH_3), 19.3 (C_q), 11.3/11.2 (CH_3); **IR** (GC/FTIR): $\tilde{\nu}$ [cm^{-1}] = 3299 (br. s), 3073 (w), 3050 (w), 2965 (s), 2932 (s), 2858 (m), 1591 (w), 1473 (m), 1462 (m), 1428 (m), 1389 (w), 1370 (m), 1334 (w), 1312 (w), 1187 (w), 1112 (s), 1075 (s), 1041 (m), 985 (m), 959 (m), 936 (m), 908 (w), 824 (m), 797 (w), 774 (w), 740 (m), 703 (s); **EI-MS** (70 eV): *m*/*z* (%) = 339 (5) [M–*t*Bu]$^+$, 201 (6), 200 (19), 199 (100), 197 (8), 181 (9), 140 (4), 139 (13), 135 (6), 123 (9), 121 (5), 81 (8), 78 (4), 77 (11), 69 (4), 57 (7), 55 (4), 45 (9), 43 (4), 41 (7); **HRMS** (EI) *m*/*z*: Calculated for $C_{21}H_{27}O_2Si$ [M–*t*Bu]$^+$ 339.17748, found: 339.17734; ***Z* isomer**: **^{1}H NMR** (300 MHz, $CDCl_3$): δ [ppm] = 7.72 – 7.60 (m, 4H, CH_{Ar}), 7.46 – 7.29 (m, 6H, CH_{Ar}), 5.02 – 4.94 (m, 1H, =CH), 4.75 – 4.65 (m, 1H, CH), 3.65 – 3.49 (m, 1H, CH), 1.82 – 1.77 (m, 3H, CH_3), 1.77 – 1.57 (m, 2H, CH_2), 1.51 – 1.19 (m, 3H, CH_2, OH), 1.13/1.12 (d, *J* = 6.4/6.4 Hz, 3H, CH_3), 1.06 – 1.02 (m, 3H, CH_3), 1.05 (s, 9H, 3×CH_3); **^{13}C NMR** (75 MHz, $CDCl_3$): δ [ppm] = 139.1/139.0 (C_q), 135.9 (4C, CH), 134.7/134.6 (C_q), 134.3/134.2 (C_q), 129.5/129.5 (CH), 129.4 (CH), 127.5/127.5 (2C, CH), 127.4 (2C, CH), 123.9/123.8 (CH), 67.6/67.5 (CH), 67.3 (CH), 39.2/39.1 (CH_2), 26.9 (3C, CH_3), 23.4/23.3 (CH_2), 23.3 (CH_3), 22.5/22.5 (CH_3), 19.3/19.3 (C_q), 17.5 (CH_3); **IR** (GC/FTIR): $\tilde{\nu}$ [cm^{-1}] = 3305 (br. s), 3073 (w), 3051 (w), 2966 (m), 2932 (m), 2895 (m), 2858 (m), 1591 (w), 1473 (w), 1428 (m), 1376 (w), 1190 (w), 1113 (s), 1079 (s), 1045 (m), 986 (m), 961 (w), 939 (w), 858 (w), 825 (m), 800 (w), 741 (m), 703 (s); **EI-MS** (70 eV): *m*/*z* (%) = 339 (5) [M–*t*Bu]$^+$, 201 (5), 200 (18), 199 (100), 197 (8), 181 (9), 139 (13), 135 (7), 123 (21),

121 (5), 81 (16), 78 (5), 77 (13), 69 (6), 67 (5), 57 (8), 55 (5), 45 (9), 43 (6), 41 (9); **HRMS** (EI) *m*/*z*: Calculated for $C_{21}H_{27}O_2Si$ [M–*t*Bu]$^+$ 339.17748, found: 339.17752.

8.2.12 5-(((7*S*)-7-((*tert*-Butyldiphenylsilyl)oxy)-6-methyloct-5-en-2-yl)thio)-1-phenyl-1*H*-tetrazole (**s6**)

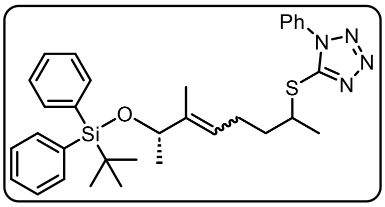

The Mitsunobu reaction was carried out analogously to procedure 8.2.9 with DIAD (0.74 ml, 3.78 mmol, 1.5 eq.), (7*S*)-7-((*tert*-butyldiphenylsilyl)oxy)-6-methyloct-5-en-2-ol (**s5**, 1.00 g, 2.52 mmol, 1.0 eq.), triphenylphosphine (991 mg, 3.78 mmol, 1.5 eq.), and 1-phenyl-1*H*-tetrazole-5-thiol (674 mg, 3.78 mmol, 1.5 eq.) in THF (10 ml). 5-(((7*S*)-7-((*tert*-butyldiphenylsilyl)oxy)-6-methyloct-5-en-2-yl)thio)-1-phenyl-1*H*-tetrazole (**s6**) was obtained as a colourless oil (1.37 g, 2.46 mmol, 98 %).

R$_f$ = (*E*/*Z*): 0.32 (Pe/Et_2O 5:1); ***E* isomer**: **^{1}H NMR** (300 MHz, $CDCl_3$): δ [ppm] = 7.73 – 7.48 (m, 9H, CH_{Ar}), 7.45 – 7.26 (m, 6H, CH_{Ar}), 5.13 – 5.04 (m, 1H, =CH), 4.17 (q, *J* = 6.3 Hz, 1H, CH), 3.99/3.98 (sext, *J* = 6.8/6.8 Hz, 1H, CH), 2.06 (q, *J* = 7.6 Hz, 2H, CH_2), 1.81 – 1.55 (m, 2H, CH_2), 1.58 (s, 3H, CH_3), 1.51/1.50 (d, *J* = 6.8/6.8 Hz, 3H, CH_3), 1.13/1.12 (d, *J* = 6.3/6.3 Hz, 3H, CH_3), 1.04/1.04 (s, 9H, 3×CH_3); **^{13}C NMR** (75 MHz, $CDCl_3$): δ [ppm] = 154.0/154.0 (C_q), 139.6/139.5 (C_q), 135.9 (2C, CH), 135.8 (2C, CH), 134.6 (C_q), 134.3 (C_q), 133.8 (C_q), 130.0 (CH), 130.0 (2C, CH), 129.4 (CH), 129.4/129.4 (CH), 127.4 (2C, CH), 127.3/127.3 (2C, CH), 124.0 (2C, CH), 122.6 (CH), 74.7/74.7 (CH), 44.5 (CH), 36.1/36.1 (CH_2), 27.0 (3C, CH_3), 24.9/24.8 (CH_2), 23.1/23.0 (CH_3), 21.5/21.4 (CH_3), 19.3 (C_q), 11.3/11.3 (CH_3); **IR** (ATR, neat): $\tilde{\nu}$ [cm^{-1}] = (*E*/*Z*): 3060 (w), 2961 (w), 2931 (w), 2858 (w), 1594 (w), 1499 (w), 1459 (w), 1420 (w), 1386 (m), 1318 (w), 1276 (w), 1238 (w), 1185 (w), 1076 (m), 982 (m), 909 (m), 855 (w), 820 (w), 733 (m), 694 (s), 611 (m), 544 (s); **HRMS** (ESI) *m*/*z*: (*E*/*Z*) Calculated for $C_{32}H_{40}N_4OSSiNa$ [M+Na]$^+$ 579.25843, found: 579.25876; ***Z* isomer**: **^{1}H NMR** (300 MHz, $CDCl_3$): δ [ppm] = 7.73 – 7.48 (m, 9H, CH_{Ar}), 7.45 – 7.26 (m, 6H, CH_{Ar}), 4.98 – 4.90 (m, 1H, =CH), 4.68 – 4.58 (m, 1H, CH), 3.86/3.86 (sext, *J* = 6.8/6.8 Hz, 1H, CH), 1.81 – 1.55 (m, 4H, 2×CH_2), 1.80 – 1.77 (m, 3H, CH_3), 1.36/1.35 (d, *J* = 6.4/6.4 Hz, 3H, CH_3), 1.12/1.09 (d, *J* = 6.8/6.8 Hz, 3H, CH_3), 1.03 (s, 9H, 3×CH_3); **^{13}C NMR** (75 MHz, $CDCl_3$): δ [ppm] = 153.9 (C_q), 139.8/139.7 (C_q), 135.8 (4C, CH), 134.5 (C_q), 134.1/134.0 (C_q), 133.8 (C_q), 130.0 (CH), 129.7 (2C, CH), 129.5/129.5 (CH), 129.4 (CH), 127.5/127.5 (2C, CH), 127.4 (2C, CH), 124.0/124.0 (2C, CH), 122.7/122.7 (CH), 67.3/67.2 (CH), 44.3/44.3 (CH), 36.4/36.2 (CH_2), 26.9 (3C, CH_3), 24.5/24.3 (CH_2), 22.5/22.5 (CH_3), 21.4/21.3 (CH_3), 19.2 (C_q), 17.5 (CH_3).

8.2.13 5-(((7*S*)-7-((*tert*-Butyldiphenylsilyl)oxy)-6-methyloct-5-en-2-yl)sulfonyl)-1-phenyl-1*H*-tetrazole (**27**)

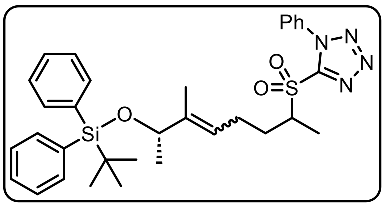

The oxidation was carried out analogously to procedure 8.2.10 with hydrogen peroxide (30 wt% in water, 1.26 ml, 12.3 mmol, 5.00 eq.), ammonium heptamolybdate tetrahydrate (152 mg, 0.123 mmol, 0.05 eq.) and 5-(((7*S*)-7-((*tert*-butyldiphenylsilyl) oxy)-6-methyloct-5-en-2-yl)thio)-1-phenyl-1*H*-tetrazole (**s6**, 1.37 g, 2.46 mmol, 1.00 eq.) in isopropanol (20 ml). The reaction mixture was stirred for only 6 h and 5-(((7*S*)-7-((*tert*-butyldiphenylsilyl)oxy)-6-methyloct-5-en-2-yl)sulfonyl)-1-phenyl-1*H*-tetrazole (**27**) was obtained as a colourless, viscous oil (1.13 g, 1.92 mmol, 78 %).

R_f = (*E*/*Z*): 0.29 (Pe/Et_2O 5:1); ***E* isomer**: **^{1}H NMR** (300 MHz, $CDCl_3$): δ [ppm] = 7.75 – 7.52 (m, 9H, CH_{Ar}), 7.46 – 7.28 (m, 6H, CH_{Ar}), 5.11 – 5.00 (m, 1H, =CH), 4.22 – 4.14 (m, 1H, CH), 3.85 – 3.71 (m, 1H, CH), 2.20 – 1.37 (m, 4H, 2×CH_2), 1.62 – 1.58 (m, 3H, CH_3), 1.47/1.46 (d, *J* = 6.9/6.9 Hz, 3H, CH_3), 1.14 (d, *J* = 6.3 Hz, 3H, CH_3), 1.05 (s, 9H, 3×CH_3); **^{13}C NMR** (75 MHz, $CDCl_3$): δ [ppm] = 152.7 (C_q), 140.9/140.8 (C_q), 135.8 (2C, CH), 135.8 (2C, CH), 134.2/134.1 (C_q), 134.5/134.5 (C_q), 133.1 (C_q), 131.4 (CH), 129.6 (2C, CH), 129.5 (2C, CH), 127.4 (2C, CH), 127.4/127.4 (2C, CH), 125.4 (2C, CH), 121.3/121.2 (CH), 74.5 (CH), 60.8 (CH), 28.1/28.0 (CH_2), 27.0 (3C, CH_3), 24.1/24.0 (CH_2), 23.1 (CH_3), 19.3 (C_q), 12.7/12.7 (CH_3), 11.4/11.4 (CH_3); **IR** (ATR, neat): $\tilde{\nu}$ [cm^{-1}] = (*E*/*Z*): 3062 (w), 2959 (w), 2936 (w), 2891 (w), 2859 (w), 1594 (w), 1498 (w), 1460 (w), 1430 (w), 1335 (m), 1267 (w), 1187 (w), 1148 (m), 1103 (m), 1076 (m), 1046 (m), 983 (m), 856 (w), 821 (m), 738 (m), 694 (s), 616 (s), 546 (m); **HRMS** (ESI) *m*/*z*: (*E*/*Z*) Calculated for $C_{32}H_{40}N_4O_3SSiNa$ $[M+Na]^+$ 611.24826, found: 611.24844; ***E* isomer**: **^{1}H NMR** (300 MHz, $CDCl_3$): δ [ppm] = 7.75 – 7.52 (m, 9H, CH_{Ar}), 7.46 – 7.28 (m, 6H, CH_{Ar}), 4.95 – 4.85 (m, 1H, =CH), 4.65 – 4.55 (m, 1H, CH), 3.69 – 3.56 (m, 1H, CH), 2.20 – 1.37 (m, 4H, 2×CH_2), 1.82 – 1.78 (m, 3H, CH_3), 1.27/1.23 (d, *J* = 6.9/6.9 Hz, 3H, CH_3), 1.13 (d, *J* = 6.3 Hz, 3H, CH_3), 1.04/1.04 (s, 9H, 3×CH_3); **^{13}C NMR** (75 MHz, $CDCl_3$): δ [ppm] = 152.7/152.7 (C_q), 141.1/140.8 (C_q), 135.8 (2C, CH), 135.7 (2C, CH), 134.4/134.4 (C_q), 133.9 (C_q), 133.1 (C_q), 131.4 (CH), 129.6 (2C, CH), 129.6 (2C, CH), 127.7 (CH), 127.5 (CH), 127.5 (CH), 127.4 (CH), 125.4 (2C, CH), 121.7/121.4 (CH), 67.3/67.2 (CH), 60.6/60.5 (CH), 28.2/28.2 (CH_2), 26.9 (3C, CH_3), 23.7/23.4 (CH_2), 22.5/22.5 (CH_3), 19.2 (C_q), 17.7/17.6 (CH_3), 12.4 (CH_3).

8.3 Synthesis of the macrolides **6-9**

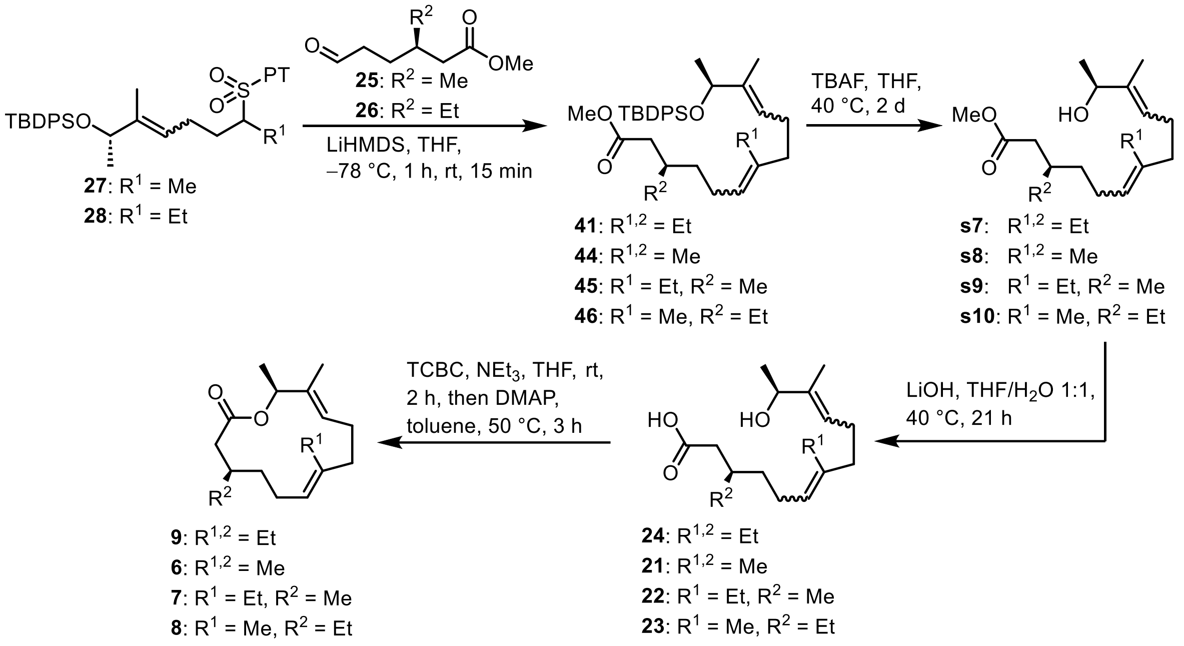

Scheme S4: Synthesis of the macrolides **6**-**9**.

8.3.1 Methyl (3*R*,12*S*)-12-((*tert*-butyldiphenylsilyl)oxy)-3,7-diethyl-11-methyltrideca-6,10-dienoate (**41**)

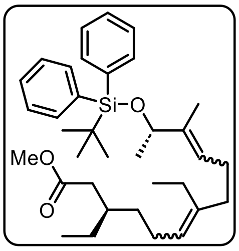

5-(((8*S*)-8-((*tert*-Butyldiphenylsilyl)oxy)-7-methylnon-6-en-3-yl)sulfonyl)-1-phenyl-1*H*-tetrazole (**28**, 45.0 mg, 74.6 µmol, 1.0 eq.) was dissolved in THF (degassed using the freeze-pump-thaw method, 0.7 ml) and LiHMDS (1 M in THF, 0.08 ml, 82.1 µmol, 1.1 eq.) was added dropwise at –78 °C over 10 min.[19] The yellow solution was then stirred at this temperature for one hour, after which methyl (*R*)-3-ethyl-6-oxohexanoate (**26**, 19.3 mg, 0.112 mmol, 1.5 eq.) in THF (degassed using the freeze-pump-thaw method, 0.2 ml) was added dropwise at –78 °C over 10 min, causing the solution to decolorize slightly. The reaction mixture was stirred at –78 °C for one hour, after which it was immediately warmed to room temperature and stirred for a further 15 min. The reaction was quenched by the addition of saturated aqueous NH_4Cl solution (10 ml), diethyl ether was added (10 ml), the phases were separated, and the aqueous phase was extracted with diethyl ether (3×10 ml). The combined organic phases were washed with saturated aqueous NaCl solution (10 ml), dried over $MgSO_4$, filtered, and the solvent removed under reduced pressure. The residue was applied to silica gel, and purification by column chromatography (pentane/diethyl ether 20:1) yielded methyl (3*R*,12*S*)-12-((*tert*-butyldiphenylsilyl)oxy)-3,7-diethyl-11-methyltrideca-6,10-dienoate (**41**) as a colourless oil in an isomer ratio (GC) of 7:8:42:43 (26.5 mg, 48.3 µmol, 65 %).

(3*S*,12*S*)-**41'** (115 mg, 0.210 mmol, 42 %) was obtained using 5-(((8*S*)-8-((*tert*-butyldiphenylsilyl)oxy)-7-methylnon-6-en-3-yl)sulfonyl)-1-phenyl-1*H*-tetrazole (**28**) and methyl (*S*)-3-ethyl-6-oxohexanoate (**26**) and (3*S*,12*R*)-**41** (160 mg, 0.292 mmol, 59 %) using 5-(((8*R*)-8-((*tert*-butyldiphenylsilyl)oxy)-7-methylnon-6-en-3-yl)sulfonyl)-1-phenyl-1*H*-tetrazole (**28**) and methyl (*S*)-3-ethyl-6-oxohexanoate (**26**) under the same conditions as colourless oils.

R_f = 0.38 (Pe/Et_2O 20:1); **^{1}H NMR** (300 MHz, $CDCl_3$): δ [ppm] = isomeric mixture 7.72 – 7.60 (m, 16H, CH_{Ar}), 7.45 – 7.28 (m, 24H, CH_{Ar}), 5.18 – 4.94 (m, 7H, =CH), 4.90 (t, *J* = 7.2 Hz, 1H, =CH), 4.73 – 4.64 (m, 2H, CH), 4.17 (q, *J* = 6.3 Hz, 2H, CH), 3.65 (s, 3H, CH_3), 3.65 (s, 3H, CH_3), 3.65 (s, 6H, CH_3), 2.25 (d, *J* = 6.9 Hz, 4H, CH_2), 2.25 (d, *J* = 6.9 Hz, 2H, CH_2), 2.22 (d, *J* = 7.0 Hz, 2H, CH_2), 2.06 – 1.63 (m, 36H, CH, 4×CH_2), 1.80 – 1.77 (m, 6H, CH_3), 1.62 – 1.59 (m, 6H, CH_3), 1.45 – 1.20 (m, 16H, 2×CH_2), 1.13 (d, *J* = 6.3 Hz, 3H, CH_3), 1.12 (d, *J* = 6.3 Hz, 3H, CH_3), 1.11 (d, *J* = 6.1 Hz, 6H, CH_3), 1.05 (s, 18H, 3×CH_3), 1.04 (s, 18H, 3×CH_3), 0.97 (t, *J* = 7.4 Hz, 3H, CH_3), 0.95 (t, *J* = 7.5 Hz, 3H, CH_3), 0.90 – 0.81 (m, 6H, 2×CH_3), 0.90 – 0.81 (m, 12H, 4×CH_3); **^{13}C NMR** (75 MHz, $CDCl_3$): δ [ppm] = isomeric mixture 174.0 (2C, C_q), 174.0 (2C, C_q), 141.0 (2C, C_q), 140.9 (2C, C_q), 138.3 (2C, C_q), 138.1 (2C, C_q), 135.9 (4C, CH), 135.8 (4C, CH), 135.8 (4C, CH), 135.8 (4C, CH), 134.8 (2C, C_q), 134.8 (2C, C_q), 134.4 (2C, C_q), 134.4 (2C, C_q), 129.4/129.3 (8C, CH), 127.5 (2C, CH), 127.4 (8C, CH), 127.3 (6C, CH), 124.3 (CH), 124.3 (CH), 124.2 (2C, CH), 123.9 (CH), 123.8 (CH), 123.4 (CH), 123.3 (CH), 74.9 (2C, CH), 67.4 (CH), 67.4 (CH), 51.4 (4C, CH_3), 38.6 (CH_2), 38.5 (CH_2), 38.5 (2C, CH_2), 36.4 (CH_2), 36.3 (2C, CH), 36.2 (CH), 36.2 (CH), 36.1 (CH_2), 33.8 (2C, CH_2), 33.8 (2C, CH_2), 30.3 (CH_2), 30.0 (CH_2), 29.5 (CH_2), 29.3 (CH_2), 27.0 (6C, CH_3), 26.9 (6C, CH_3), 26.6 (CH_2), 26.3 (CH_2), 26.2 (2C, CH_2), 26.2 (2C, CH_2), 25.9 (CH_2), 25.8 (CH_2), 24.8 (CH_2), 24.7 (CH_2), 24.7 (2C, CH_2), 23.1 (CH_2), 23.1 (CH_3), 23.1 (CH_3), 23.0 (CH_2), 22.6 (CH_3), 22.5 (CH_3), 19.3 (4C, C_q), 17.5 (CH_3), 17.5 (CH_3), 13.3 (CH_3), 13.1 (CH_3), 12.9 (CH_3), 12.8 (CH_3), 11.2 (CH_3), 11.1 (CH_3), 10.7 (2C, CH_3), 10.7 (2C, CH_3); **IR** (GC/FTIR): $\tilde{\nu}$ [cm^{-1}] = 1. isomer 3073 (w), 2963 (s), 2933 (m), 2858 (m), 1739 (s), 1591 (w), 1462 (m), 1429 (m), 1377 (w), 1259 (w), 1193 (w), 1160 (w), 1113 (m), 1079 (m), 1045 (m), 1009 (w), 979 (w), 940 (w), 825 (m), 742 (m), 703 (s); 2. isomer 3073 (w), 2964 (s), 2932 (m), 2858 (m), 1739 (s), 1591 (w), 1461 (m), 1429 (m), 1377 (w), 1259 (w), 1193 (w), 1156 (w), 1112 (m), 1077 (m), 1044 (m), 1008 (w), 979 (w), 941 (w), 825 (m), 742 (m), 703 (s); 3. isomer 3073 (w), 3049 (w), 2963 (s), 2932 (s), 2858 (m), 1739 (s), 1591 (w), 1462 (m), 1429 (m), 1368 (w), 1311 (w), 1256 (w), 1193 (w), 1160 (w), 1112 (m), 1078 (m), 1046 (m), 982 (m), 939 (w), 858 (w), 824 (m), 774 (w), 741 (m), 703 (s); 4. isomer 3073 (w), 2963 (s), 2932 (s), 2858 (m), 1739 (s), 1591 (w), 1461 (m), 1429 (m), 1369 (w), 1312 (w), 1257 (w), 1192 (w), 1155 (w), 1112 (m), 1079 (m), 1048 (m), 983 (m), 940 (w), 825 (m), 776 (w), 741 (m), 703 (s); **EI-MS** (70 eV): *m/z* (%) = 1. isomer 491 (8) [M–*t*Bu]$^+$, 282 (5), 253 (7), 213 (10), 200 (21), 199 (100), 197 (14), 181 (9), 136 (10), 135 (76), 121 (9), 107 (10), 95 (12), 83 (9), 81 (21), 78 (9), 77 (13), 69 (8), 67 (11), 55 (15), 41 (14); 2. isomer 491 (5) [M–

*t*Bu]$^+$, 213 (6), 201 (7), 200 (16), 199 (100), 197 (9), 181 (9), 137 (10), 136 (10), 135 (71), 121 (10), 107 (10), 95 (12), 83 (8), 81 (16), 79 (8), 77 (13), 69 (9), 67 (10), 55 (15), 41 (14); 3. isomer 491 (6) [M–*t*Bu]$^+$, 200 (19), 199 (100), 197 (9), 181 (8), 137 (10), 136 (9), 135 (58), 121 (8), 107 (9), 95 (13), 93 (8), 83 (8), 81 (21), 79 (9), 77 (14), 69 (9), 67 (13), 55 (17), 41 (15); 4. isomer 491 (5) [M–*t*Bu]$^+$, 213 (5), 201 (6), 200 (17), 199 (100), 197 (9), 181 (7), 137 (9), 135 (47), 121 (9), 107 (9), 95 (11), 93 (8), 83 (8), 81 (18), 79 (9), 77 (15), 69 (8), 67 (11), 55 (16), 45 (8), 41 (15).; **HRMS** (CIP) *m/z*: Calculated for $C_{35}H_{53}O_3Si$ [M+H]$^+$ 549.37585, found: 1. isomer 549.37567, 2. isomer 549.37616, 3. isomer 549.37567, 4. isomer 549.37653.

(3*S*,12*S*)-**41'**:

R$_f$ = 0.38 (Pe/Et_2O 20:1); **^{1}H NMR** (300 MHz, $CDCl_3$): δ [ppm] = isomeric mixture 7.72 – 7.60 (m, 16H, CH_{Ar}), 7.45 – 7.28 (m, 24H, CH_{Ar}), 5.18 – 4.94 (m, 7H, =CH), 4.90 (t, *J* = 7.2 Hz, 1H, =CH), 4.74 – 4.65 (m, 2H, CH), 4.17 (q, *J* = 6.3 Hz, 2H, CH), 3.65 (s, 6H, CH_3), 3.64 (s, 6H, CH_3), 2.25 (d, *J* = 6.9 Hz, 4H, CH_2), 2.25 (d, *J* = 6.9 Hz, 2H, CH_2), 2.22 (d, *J* = 7.0 Hz, 2H, CH_2), 2.06 – 1.63 (m, 36H, CH, 4×CH_2), 1.80 – 1.77 (m, 6H, CH_3), 1.62 – 1.59 (m, 6H, CH_3), 1.45 – 1.20 (m, 16H, 2×CH_2), 1.14 (d, *J* = 6.3 Hz, 3H, CH_3), 1.13 (d, *J* = 6.3 Hz, 3H, CH_3), 1.11 (d, *J* = 6.3 Hz, 6H, CH_3), 1.05 (s, 9H, 3×CH_3), 1.05 (s, 27H, 3×CH_3), 0.97 (t, *J* = 7.3 Hz, 3H, CH_3), 0.95 (t, *J* = 7.5 Hz, 3H, CH_3), 0.90 – 0.81 (m, 6H, 2×CH_3), 0.90 – 0.81 (m, 12H, 4×CH_3); **^{13}C NMR** (75 MHz, $CDCl_3$): δ [ppm] = isomeric mixture 174.0 (2C, C_q), 173.9 (2C, C_q), 140.9 (C_q), 140.9 (C_q), 140.7 (C_q), 140.5 (C_q), 138.6 (C_q), 138.3 (C_q), 138.3 (C_q), 138.1 (C_q), 135.9 (4C, CH), 135.8 (4C, CH), 135.8 (4C, CH), 135.8 (4C, CH), 134.8 (C_q), 134.8 (C_q), 134.8 (C_q), 134.7 (C_q), 134.4 (C_q), 134.3 (C_q), 134.2 (C_q), 134.2 (C_q), 129.4/129.3 (8C, CH), 127.4 (2C, CH), 127.4 (8C, CH), 127.3 (6C, CH), 124.3 (CH), 124.3 (CH), 124.2 (2C, CH), 123.9 (CH), 123.8 (CH), 123.4 (CH), 123.3 (CH), 74.9 (2C, CH), 67.4 (CH), 67.3 (CH), 51.3 (4C, CH_3), 38.5 (2C, CH_2), 38.5 (2C, CH_2), 36.4 (CH_2), 36.3 (2C, CH), 36.2 (CH), 36.2 (CH), 36.1 (CH_2), 33.8 (2C, CH_2), 33.8 (2C, CH_2), 30.3 (CH_2), 30.0 (CH_2), 29.5 (CH_2), 29.3 (CH_2), 27.0 (6C, CH_3), 26.9 (6C, CH_3), 26.6 (CH_2), 26.3 (CH_2), 26.2 (2C, CH_2), 26.2 (CH_2), 26.1 (CH_2), 25.9 (CH_2), 25.8 (CH_2), 24.8 (CH_2), 24.7 (CH_2), 24.7 (CH_2), 24.6 (CH_2), 23.1 (CH_2), 23.1 (CH_3), 23.1 (CH_3), 23.0 (CH_2), 22.6 (CH_3), 22.5 (CH_3), 19.3 (4C, C_q), 17.5 (CH_3), 17.5 (CH_3), 13.2 (CH_3), 13.1 (CH_3), 12.9 (CH_3), 12.8 (CH_3), 11.2 (CH_3), 11.1 (CH_3), 10.7 (4C, CH_3); **IR** (GC/FTIR): $\tilde{\nu}$ [cm^{-1}] = 1. isomer 3073 (w), 3053 (w), 2963 (s), 2933 (s), 2858 (m), 1739 (s), 1591 (w), 1461 (m), 1429 (m), 1377 (w), 1365 (w), 1259 (w), 1193 (w), 1160 (w), 1113 (m), 1079 (m), 1045 (m), 1008 (w), 979 (w), 941 (w), 825 (m), 742 (m), 703 (s); 2. isomer 3073 (w), 3049 (w), 2964 (s), 2932 (s), 2858 (m), 1739 (s), 1591 (w), 1461 (m), 1429 (m), 1377 (w), 1365 (w), 1259 (w), 1193 (w), 1156 (w), 1112 (m), 1077 (m), 1044 (m), 1008 (w), 979 (w), 941 (w), 825 (m), 742 (m), 703 (s); 3. isomer 3073 (w), 3051 (w), 2963 (s), 2932 (s), 2858 (m), 1739 (s), 1591 (w), 1461 (m), 1429 (m), 1368 (w), 1311 (w), 1257 (w), 1193 (w), 1160 (w), 1112 (m), 1078 (m), 1046 (m), 982 (m),

939 (w), 859 (w), 825 (m), 774 (w), 742 (m), 703 (s); 4. isomer 3073 (w), 3050 (w), 2964 (s), 2932 (s), 2858 (m), 1739 (s), 1591 (w), 1461 (m), 1429 (m), 1369 (w), 1313 (w), 1257 (w), 1192 (w), 1156 (w), 1112 (m), 1079 (m), 1048 (m), 983 (m), 939 (w), 855 (w), 825 (m), 774 (w), 742 (m), 703 (s); **EI-MS** (70 eV): *m*/*z* (%) = 1. isomer 491 (12) [M–*t*Bu]$^+$, 213 (8), 200 (17), 199 (100), 197 (11), 181 (8), 137 (8), 136 (9), 135 (71), 121 (8), 107 (9), 95 (11), 83 (6), 81 (16), 79 (6), 77 (10), 69 (7), 67 (9), 55 (11), 41 (8); 2. isomer 491 (10) [M–*t*Bu]$^+$, 213 (6), 200 (18), 199 (100), 197 (10), 181 (8), 137 (8), 136 (9), 135 (63), 121 (7), 107 (8), 95 (10), 83 (6), 81 (14), 79 (6), 77 (8), 69 (6), 67 (9), 55 (10), 41 (7); 3. isomer 491 (14) [M–*t*Bu]$^+$, 213 (8), 200 (19), 199 (100), 197 (10), 181 (7), 137 (9), 136 (9), 135 (60), 121 (7), 107 (8), 95 (11), 83 (6), 81 (17), 79 (6), 77 (8), 69 (7), 67 (6), 55 (10), 41 (7); 4. isomer 491 (14) [M–*t*Bu]$^+$, 213 (6), 200 (19), 199 (100), 197 (10), 181 (7), 137 (9), 136 (8), 135 (54), 121 (7), 107 (7), 95 (10), 83 (6), 81 (16), 79 (5), 77 (8), 69 (6), 67 (9), 55 (9), 41 (7); **HRMS** (CIP) *m*/*z*: Calculated for $C_{35}H_{53}O_3Si$ [M+H]$^+$ 549.37585, found: 1. isomer 549.37616, 2. isomer 549.37610, 3. isomer 549.37567 , 4. isomer 549.37683.

8.3.2 Methyl (3*R*,12*S*)-12-((*tert*-butyldiphenylsilyl)oxy)-3,7,11-trimethyltrideca-6,10-dien-oate (**44**)

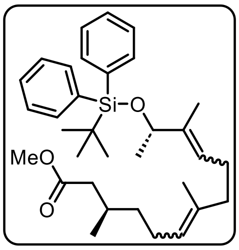

The Julia-Kocienski olefination was carried out analogously to procedure 8.3.1 with 5-(((7*S*)-7-((*tert*-butyldiphenylsilyl)oxy)-6-methyloct-5-en-2-yl)sulfonyl)-1-phenyl-1*H*-tetrazole (**27**, 500 mg, 0.849 mmol, 1.00 eq.), LiHMDS (1 M in THF, 0.89 ml, 0.891 mmol, 1.05 eq.) in degassed THF (7 ml) and methyl (*R*)-3-methyl-6-oxohexanoate (**25**, 148 mg, 0.934 mmol, 1.10 eq.) in degassed THF (2 ml). The reaction mixture was stirred for 15 h over night at room temperature and methyl (3*R*,12*S*)-12-((*tert*-butyldiphenylsilyl)oxy)-3,7,11-trimethyltrideca-6,10-dien-oate (**44**) was obtained as a colourless oil in an isomer ratio (GC) of 8:14:34:43 (220 mg, 0.422 mmol, 50 %). 5-(((7*S*)-7-((*tert*-Butyldiphenylsilyl)oxy)-6-methyloct-5-en-2-yl)sulfonyl)-1-phenyl-1*H*-tetrazole (**27**, 192 mg) was recovered.

R$_f$ = 0.25 (Pe/Et_2O 20:1); **^{1}H NMR** (300 MHz, $CDCl_3$): *δ* [ppm] = isomeric mixture 7.71 – 7.61 (m, 16H, CH_{Ar}), 7.45 – 7.29 (m, 24H, CH_{Ar}), 5.17 – 4.91 (m, 8H, =CH), 4.74 – 4.65 (m, 2H, CH), 4.22 –4.13 (m, 2H, CH), 3.65 (s, 6H, CH_3), 3.65 (s, 6H, CH_3), 2.36 – 2.24 (m, 4H, CH_aH_b), 2.16 – 2.06 (m, 4H, CH_aH_b), 2.06 – 1.67 (m, 28H, CH, 3×CH_2), 1.80 – 1.76 (m, 3H, CH_3), 1.67 – 1.64 (m, 3H, CH_3), 1.63 – 1.59 (m, 6H, CH_3), 1.59 – 1.56 (m, 6H, CH_3), 1.52 – 1.49 (m, 3H, CH_3), 1.46 – 1.42 (m, 3H, CH_3), 1.40 – 1.10 (m, 8H, CH_2), 1.13 (d, *J* = 6.4 Hz, 3H, CH_3), 1.12 (d, *J* = 6.3 Hz, 3H, CH_3), 1.11 (d, *J* = 6.6 Hz, 6H, CH_3), 1.05 (s, 18H, 3×CH_3), 1.05 (s, 9H, 3×CH_3), 1.05 (s, 9H, 3×CH_3), 0.94 (d, *J* = 6.6 Hz, 3H, CH_3), 0.93 (d, *J* = 6.6 Hz, 3H, CH_3), 0.93 (d, *J* = 6.6 Hz, 3H, CH_3), 0.89 (d, *J* = 6.6 Hz, 3H, CH_3); **^{13}C NMR** (75 MHz, $CDCl_3$): *δ* [ppm] =

isomeric mixture 173.7 (C_q), 173.7 (C_q), 173.6 (2C, C_q), 138.6 (C_q), 138.4 (C_q), 138.3 (C_q), 138.1 (C_q), 135.9 (4C, CH), 135.8 (4C, CH), 135.8 (4C, CH), 135.8 (4C, CH), 135.3 (C_q), 135.0 (C_q), 134.9 (C_q), 134.8 (C_q), 134.8 (2C, C_q), 134.8 (C_q), 134.7 (C_q), 134.4 (C_q), 134.3 (C_q), 134.2 (C_q), 134.2 (C_q), 129.5 (CH), 129.4 (CH), 129.4 (2C, CH), 129.4 (2C, CH), 129.3 (CH), 129.3 (CH), 127.4 (4C, CH), 127.4 (8C, CH), 127.3 (4C, CH), 125.1 (CH), 124.9 (CH), 124.2 (CH), 124.2 (CH), 124.1 (4C, CH), 74.9 (2C, CH), 67.4 (CH), 67.3 (CH), 51.3 (4C, CH_3), 41.6 (CH_2), 41.6 (3C, CH_2), 39.6 (CH_2), 39.3 (CH_2), 37.0 (CH_2), 37.0 (CH_2), 36.8 (CH_2), 36.7 (CH_2), 31.8 (CH_2), 31.5 (CH_2), 30.1 (CH), 30.1 (CH), 30.0 (CH), 30.0 (CH), 27.0 (6C, CH_3), 26.9 (6C, CH_3), 26.1 (CH_2), 26.0 (CH_2), 25.6 (CH_2), 25.5 (CH_2), 25.3 (CH_2), 25.3 (CH_2), 25.2 (CH_2), 25.1 (CH_2), 23.4 (CH_3), 23.2 (CH_3), 23.1 (CH_3), 23.1 (CH_3), 22.6 (CH_3), 22.5 (CH_3), 19.6 (2C, CH_3), 19.6 (CH_3), 19.6 (CH_3), 19.5 (4C, C_q), 17.5 (CH_3), 17.5 (CH_3), 15.9 (CH_3), 15.8 (CH_3), 11.2 (CH_3), 11.2 (CH_3); **IR** (GC/FTIR): $\tilde{\nu}$ [cm^{-1}] = 1. isomer 3072 (w), 3049 (w), 2963 (s), 2931 (s), 2856 (m), 1740 (s), 1592 (w), 1474 (m), 1462 (m), 1430 (m), 1379 (m), 1366 (w), 1291 (w), 1264 (w), 1200 (w), 1163 (w), 1114 (s), 1079 (m), 1047 (m), 1009 (w), 989 (w), 942 (w), 827 (m), 800 (w), 744 (m), 705 (s); 2. isomer 3072 (w), 3049 (w), 2962 (s), 2931 (s), 2856 (m), 1740 (s), 1591 (w), 1474 (m), 1462 (m), 1430 (m), 1379 (m), 1366 (m), 1291 (w), 1264 (w), 1198 (w), 1158 (w), 1114 (s), 1096 (m), 1078 (m), 1046 (m), 1009 (w), 999 (w), 943 (w), 827 (m), 800 (w), 743 (m), 705 (s); 3. isomer 3072 (w), 3049 (w), 2960 (s), 2931 (s), 2856 (m), 1740 (s), 1591 (w), 1462 (m), 1430 (m), 1370 (w), 1314 (w), 1263 (w), 1200 (w), 1162 (w), 1114 (s), 1082 (m), 1052 (m), 1009 (w), 987 (m), 942 (w), 826 (m), 775 (w), 743 (m), 705 (s); 4. isomer 3072 (w), 3049 (w), 2960 (s), 2931 (s), 2856 (m), 1739 (s), 1591 (w), 1474 (m), 1462 (m), 1430 (m), 1379 (m), 1369 (w), 1263 (w), 1198 (w), 1156 (m), 1114 (s), 1082 (m), 1009 (w), 986 (m), 942 (w), 826 (m), 776 (w), 743 (m), 705 (s); **EI-MS** (70 eV): *m*/*z* (%) = 1. isomer 463 (7) [M–*t*Bu]$^+$, 279 (1), 265 (3), 253 (1), 233 (1), 213 (9), 200 (18), 199 (100), 197 (10), 181 (8), 135 (22), 122 (8), 121 (57), 109 (9), 107 (7), 105 (7), 95 (8), 93 (8), 81 (16), 77 (11), 69 (10), 67 (11), 55 (8), 41 (9); 2. isomer 463 (10) [M–*t*Bu]$^+$, 279 (2), 265 (3), 253 (1), 233 (1), 213 (7), 200 (18), 199 (100), 197 (10), 181 (8), 135 (19), 122 (7), 121 (50), 109 (9), 107 (6), 105 (5), 95 (6), 93 (6), 81 (13), 77 (9), 69 (8), 67 (8), 55 (6), 41 (7); 3. isomer 463 (10) [M–*t*Bu]$^+$, 279 (1), 265 (3), 253 (1), 233 (1), 213 (9), 200 (18), 199 (100), 197 (10), 181 (8), 135 (20), 122 (7), 121 (45), 109 (11), 107 (6), 105 (6), 95 (7), 93 (7), 81 (17), 77 (10), 69 (9), 67 (10), 55 (7), 41 (8); 4. isomer 463 (10) [M–*t*Bu]$^+$, 279 (1), 265 (3), 253 (1), 233 (1), 213 (7), 200 (18), 199 (100), 197 (10), 181 (8), 135 (19), 122 (6), 121 (41), 109 (10), 107 (6), 105 (5), 95 (6), 93 (6), 81 (15), 77 (10), 69 (8), 67 (9), 55 (6), 41 (7); **HRMS** (CIP) *m*/*z*: Calculated for $C_{33}H_{49}O_3Si$ [M+H]$^+$ 521.34455, found: 1. isomer 521.34454, 2. isomer 521.34473, 3. isomer 521.34497, 4. isomer 521.34479.

8.3.3 Methyl (3*R*,12*S*)-12-((*tert*-butyldiphenylsilyl)oxy)-7-ethyl-3,11-dimethyltrideca-6,10-dienoate (**45**)

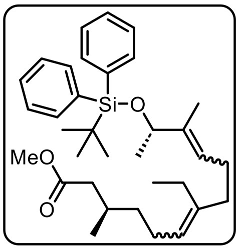

The Julia-Kocienski olefination was carried out analogously to procedure 8.3.1 with 5-(((8*S*)-8-((*tert*-butyldiphenylsilyl)oxy)-7-methylnon-6-en-3-yl)sulfonyl)-1-phenyl-1*H*-tetrazole (**28**, 133 mg, 0.221 mmol, 1.00 eq.), LiHMDS (1 M in THF, 0.23 ml, 0.232 mmol, 1.05 eq.) in degassed THF (2 ml) and methyl (*R*)-3-methyl-6-oxohexanoate (**25**, 38.4 mg, 0.243 mmol, 1.10 eq.) in degassed THF (1 ml). The reaction mixture was stirred for 15 h over night at room temperature and methyl (3*R*,12*S*)-12-((*tert*-butyldiphenylsilyl)oxy)-7-ethyl-3,11-dimethyltrideca-6,10-dienoate (**45**) was obtained as a colourless oil in an isomer ratio (GC) of 9:13:39:39 (29.1 mg, 54.4 µmol, 25 %). 5-(((8*S*)-8-((*tert*-Butyldiphenylsilyl)oxy)-7-methylnon-6-en-3-yl)sulfonyl)-1-phenyl-1*H*-tetrazole (**28**, 74.4 mg) was recovered.

R_f = 0.26 (Pe/Et_2O 20:1); **^{1}H NMR** (300 MHz, $CDCl_3$): δ [ppm] = isomeric mixture 7.73 – 7.60 (m, 16H, CH_{Ar}), 7.45 – 7.29 (m, 24H, CH_{Ar}), 5.18 – 4.94 (m, 7H, =CH), 4.89 (t, J = 7.1 Hz, 1H, =CH), 4.74 – 4.64 (m, 2H, CH), 4.17 (q, J = 6.3 Hz, 2H, CH), 3.66 (s, 6H, CH_3), 3.65 (s, 6H, CH_3), 2.38 – 2.24 (m, 4H, CH_aH_b), 2.18 – 2.05 (m, 4H, CH_aH_b), 2.05 – 1.64 (m, 36H, CH, 4×CH_2), 1.81 – 1.75 (m, 6H, CH_3), 1.63 – 1.58 (m, 6H, CH_3), 1.44 – 1.08 (m, 8H, CH_2), 1.14 (d, J = 6.3 Hz, 3H, CH_3), 1.12 (d, J = 6.3 Hz, 3H, CH_3), 1.10 (d, J = 6.3 Hz, 6H, CH_3), 1.05 (s, 18H, 3×CH_3), 1.05 (s, 18H, 3×CH_3), 1.01 – 0.81 (m, 24H, 2×CH_3); **^{13}C NMR** (75 MHz, $CDCl_3$): δ [ppm] = 173.7 (2C, C_q), 173.7 (2C, C_q), 141.0 (C_q), 141.0 (C_q), 140.7 (C_q), 140.5 (C_q), 138.6 (C_q), 138.3 (C_q), 138.3 (C_q), 138.1 (C_q), 135.9 (4C, CH), 135.8 (4C, CH), 135.8 (4C, CH), 135.8 (4C, CH), 134.8 (C_q), 134.8 (C_q), 134.8 (C_q), 134.7 (C_q), 134.4 (C_q), 134.4 (C_q), 134.2 (C_q), 134.2 (C_q), 129.4 (2C, CH), 129.4 (4C, CH), 129.4 (CH), 129.3 (CH), 127.4 (4C, CH), 127.4 (8C, CH), 127.3 (4C, CH), 124.3 (CH), 124.3 (CH), 124.2 (2C, CH), 123.7 (CH), 123.7 (CH), 123.3 (CH), 123.2 (CH), 74.9 (2C, CH), 67.4 (CH), 67.3 (CH), 51.3 (4C, CH_3), 41.6 (2C, CH_2), 41.6 (2C, CH_2), 37.1 (3C, CH_2), 37.0 (CH_2), 36.4 (CH_2), 36.1 (CH_2), 30.3 (CH_2), 30.1 (2C, CH), 30.1 (2C, CH), 30.0 (CH_2), 29.5 (CH_2), 29.3 (CH_2), 27.0 (6C, CH_3), 26.9 (6C, CH_3), 26.5 (CH_2), 26.3 (CH_2), 25.9 (CH_2), 25.8 (CH_2), 25.1 (CH_2), 25.0 (2C, CH_2), 24.9 (CH_2), 23.1 (CH_2), 23.1 (CH_3), 23.1 (CH_3), 23.0 (CH_2), 22.6 (CH_3), 22.5 (CH_3), 19.7 (2C, CH_3), 19.6 (CH_3), 19.6 (CH_3), 19.3 (4C, C_q), 17.5 (CH_3), 17.5 (CH_3), 13.2 (CH_3), 13.1 (CH_3), 12.9 (CH_3), 12.8 (CH_3), 11.2 (CH_3), 11.1 (CH_3); **IR** (GC/FTIR): $\tilde{\nu}$ [cm^{-1}] = 1. isomer 2963 (s), 2932 (m), 2857 (m), 1740 (s), 1463 (m), 1430 (m), 1379 (w), 1366 (w), 1198 (w), 1161 (w), 1114 (s), 1080 (s), 1046 (m), 1009 (w), 827 (m), 744 (m), 705 (s); 2. isomer 2963 (s), 2932 (m), 2857 (m), 1740 (s), 1474 (w), 1462 (m), 1430 (m), 1379 (w), 1366 (w), 1262 (m), 1196 (w), 1156 (w), 1114 (s), 1079 (s), 1045 (m), 1026 (m), 981 (w), 825 (m), 805 (m), 743 (m), 705 (s); 3. isomer 2962 (s), 2931 (s), 2857 (m), 1740 (s), 1463 (m), 1430 (m), 1370 (m), 1263 (w), 1198 (w), 1161 (w), 1114 (m), 1079 (m), 1048 (m), 1008 (w), 1000 (w), 984 (m), 941 (w), 866 (w), 826 (m), 743 (m), 705 (s); 4. isomer

2964 (s), 2933 (s), 2857 (m), 1740 (s), 1462 (m), 1430 (m), 1370 (w), 1315 (w), 1291 (w), 1264 (w), 1196 (w), 1156 (w), 1114 (s), 1080 (s), 1049 (m), 985 (m), 941 (w), 826 (m), 743 (m), 705 (s); **EI-MS** (70 eV): *m*/*z* (%) = 1. isomer 477 (6) $[M–tBu]^+$, 281 (2), 265 (2), 253 (3), 229 (3), 213 (5), 200 (17), 199 (100), 197 (9), 181 (7), 136 (7), 135 (48), 123 (7), 121 (9), 107 (10), 95 (8), 93 (8), 81 (14), 79 (8), 77 (13), 67 (10), 55 (10), 41 (11); 2. isomer 477 (5) $[M–tBu]^+$, 281 (2), 265 (2), 253 (2), 229 (3), 213 (4), 200 (18), 199 (100), 197 (8), 181 (7), 136 (7), 135 (42), 123 (7), 121 (9), 107 (10), 95 (8), 93 (8), 81 (13), 79 (8), 77 (13), 67 (9), 55 (10), 41 (10); 3. isomer 477 (5) $[M–tBu]^+$, 281 (1), 265 (1), 253 (1), 229 (2), 213 (4), 200 (18), 199 (100), 197 (7), 181 (7), 136 (7), 135 (36), 123 (9), 121 (9), 107 (9), 95 (8), 93 (8), 81 (17), 79 (9), 77 (14), 67 (10), 55 (10), 41 (12); 4. isomer 477 (4) $[M–tBu]^+$, 281 (1), 265 (2), 253 (1), 229 (2), 213 (3), 200 (19), 199 (100), 197 (7), 181 (7), 136 (6), 135 (31), 123 (8), 121 (9), 107 (9), 95 (8), 93 (8), 81 (15), 79 (9), 77 (15), 67 (9), 55 (10), 41 (11); **HRMS** (CIP) *m*/*z*: Calculated for $C_{34}H_{51}O_3Si$ $[M+H]^+$ 535.36020, found: 1. isomer 535.36078, 2. isomer 535.36121, 3. isomer 535.36041, 4. isomer 535.36053.

8.3.4 Methyl (3*R*,12*S*)-12-((*tert*-butyldiphenylsilyl)oxy)-3-ethyl-7,11-dimethyltrideca-6,10-dienoate (**46**)

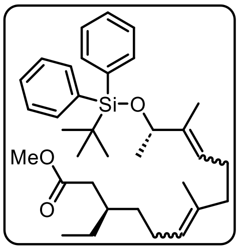

The Julia-Kocienski olefination was carried out analogously to procedure 8.3.1 with 5-(((7*S*)-7-((*tert*-butyldiphenylsilyl)oxy)-6-methyloct-5-en-2-yl)sulfonyl)-1-phenyl-1*H*-tetrazole (**27**, 144 mg, 0.244 mmol, 1.20 eq.), LiHMDS (1 M in THF, 0.23 ml, 0.233 mmol, 1.15 eq.) in degassed THF (2 ml) and methyl (*R*)-3-ethyl-6-oxohexanoate (**26**, 35.0 mg, 0.203 mmol, 1.00 eq.) in degassed THF (1 ml). The reaction mixture was stirred for 15 h over night at room temperature and methyl (3*R*,12*S*)-12-((*tert*-butyldiphenylsilyl)oxy)-3-ethyl-7,11-dimethyltri-deca-6,10-dienoate (**46**) was obtained as a colourless oil in an isomer ratio (GC) of 15:8:43:34 (31.1 mg, 58.1 µmol, 29 %).

R_f = 0.26 (Pe/Et_2O 20:1); **^{1}H NMR** (300 MHz, $CDCl_3$): δ [ppm] = isomeric mixture 7.72 – 7.60 (m, 16H, CH_{Ar}), 7.45 – 7.29 (m, 24H, CH_{Ar}), 5.17 – 4.91 (m, 8H, 2×=CH), 4.75 – 4.64 (m, 2H, CH), 4.22 – 4.12 (m, 2H, CH), 3.65 (s, 6H, CH_3), 3.64 (s, 6H, CH_3), 2.25 (d, J = 6.9 Hz, 4H, CH_2), 2.24 (d, J = 7.0 Hz, 2H, CH_2), 2.22 (d, J = 6.9 Hz, 2H, CH_2), 2.07 – 1.67 (m, 28H, CH, 3×CH_2), 1.80 – 1.76 (m, 3H, CH_3), 1.68 – 1.64 (m, 3H, CH_3), 1.63 – 1.59 (m, 6H, CH_3), 1.59 – 1.55 (m, 6H, CH_3), 1.52 – 1.49 (m, 3H, CH_3), 1.46 – 1.43 (m, 3H, CH_3), 1.43 – 1.16 (m, 16H, 2×CH_2), 1.13 (d, J = 6.4 Hz, 3H, CH_3), 1.12 (d, J = 6.3 Hz, 3H, CH_3), 1.11 (d, J = 6.4 Hz, 6H, CH_3), 1.07 – 1.02 (m, 36H, 3×CH_3), 0.91 – 0.81 (m, 12H, CH_3); **^{13}C NMR** (75 MHz, $CDCl_3$): δ [ppm] = isomeric mixture 174.0 (C_q), 174.0 (C_q), 173.9 (2C, C_q), 138.6 (C_q), 138.4 (C_q), 138.3 (C_q), 138.1 (C_q), 135.9 (4C, CH), 135.8 (4C, CH), 135.8 (4C, CH), 135.8 (4C, CH), 135.3 (C_q),

135.0 (2C, C_q), 134.8 (2C, C_q), 134.8 (C_q), 134.8 (C_q), 134.8 (C_q), 134.4 (C_q), 134.3 (C_q), 134.2 (C_q), 134.2 (C_q), 129.5 (CH), 129.4 (CH), 129.4 (2C, CH), 129.4 (2C, CH), 129.4 (CH), 129.3 (CH), 127.5 (4C, CH), 127.4 (8C, CH), 127.3 (4C, CH), 125.2 (CH), 125.0 (CH), 124.3 (CH), 124.2 (CH), 124.2 (CH), 124.2 (CH), 124.1 (2C, CH), 74.9 (2C, CH), 67.4 (CH), 67.4 (CH), 51.3 (4C, CH_3), 39.6 (CH_2), 39.3 (CH_2), 38.5 (3C, CH_2), 38.5 (CH_2), 36.2 (CH), 36.2 (CH), 36.2 (CH), 36.1 (CH), 33.7 (CH_2), 33.7 (CH_2), 33.5 (CH_2), 33.4 (CH_2), 31.8 (CH_2), 31.5 (CH_2), 27.0 (6C, CH_3), 26.9 (6C, CH_3), 26.2 (2C, CH_2), 26.1 (CH_2), 26.1 (CH_2), 26.1 (CH_2), 26.1 (CH_2), 25.6 (CH_2), 25.5 (CH_2), 25.0 (CH_2), 25.0 (CH_2), 24.9 (CH_2), 24.8 (CH_2), 23.4 (CH_3), 23.2 (CH_3), 23.1 (CH_3), 23.1 (CH_3), 22.6 (CH_3), 22.5 (CH_3), 19.3 (4C, C_q), 17.5 (CH_3), 17.5 (CH_3), 15.9 (CH_3), 15.8 (CH_3), 11.2 (CH_3), 11.1 (CH_3), 10.7 (2C, CH_3), 10.7 (2C, CH_3); **IR** (GC/FTIR): $\tilde{\nu}$ [cm^{-1}] = 1. isomer 2963 (s), 2931 (s), 2857 (m), 1739 (s), 1430 (m), 1378 (w), 1261 (w), 1194 (w), 1162 (w), 1114 (s), 1079 (s), 1046 (m), 994 (w), 942 (w), 827 (m), 743 (m), 705 (s); 2. isomer 2964 (s), 2932 (s), 2858 (m), 1739 (s), 1430 (m), 1378 (w), 1365 (w), 1344 (w), 1311 (w), 1259 (w), 1194 (w), 1160 (w), 1114 (m), 1079 (m), 1047 (m), 988 (w), 942 (w), 827 (m), 743 (m), 705 (s); 3. isomer 2964 (s), 2932 (s), 2857 (m), 1739 (s), 1430 (m), 1371 (w), 1342 (w), 1312 (w), 1259 (w), 1194 (w), 1162 (w), 1114 (m), 1079 (m), 1050 (m), 1011 (w), 987 (m), 942 (w), 827 (m), 774 (w), 743 (m), 705 (s); 4. isomer 2964 (s), 2932 (s), 2858 (m), 1739 (s), 1430 (m), 1370 (w), 1342 (w), 1312 (w), 1259 (w), 1194 (w), 1157 (w), 1114 (m), 1081 (m), 1053 (m), 1010 (w), 987 (m), 942 (w), 826 (m), 774 (w), 743 (m), 705 (s); **EI-MS** (70 eV): *m*/*z* (%) = 1. isomer 477 (10) [M–*t*Bu]$^+$, 355 (10), 213 (9), 209 (9), 207 (21), 200 (20), 199 (100), 197 (10), 135 (18), 123 (9), 122 (10), 121 (47), 107 (9), 105 (9), 81 (10), 77 (13), 59 (9), 55 (8), 44 (9), 41 (10); 2. isomer 477 (4) [M–*t*Bu]$^+$, 355 (5), 327 (6), 229 (5), 213 (10), 200 (18), 199 (100), 198 (12), 197 (12), 181 (9), 135 (18), 123 (12), 122 (5), 121 (46), 105 (6), 81 (11), 77 (14), 73 (16), 67 (13), 57 (12), 55 (9), 41 (12); 3. isomer 477 (11) [M–*t*Bu]$^+$, 327 (10), 208 (10), 207 (26), 200 (13), 199 (100), 198 (17), 197 (10), 193 (9), 181 (14), 135 (20), 123 (11), 121 (29), 81 (12), 77 (14), 73 (13), 59 (10), 57 (12), 55 (12), 41 (14); 4. isomer 477 (13) [M–*t*Bu]$^+$, 327 (5), 213 (9), 207 (15), 201 (8), 200 (20), 199 (100), 197 (9), 181 (9), 135 (19), 123 (11), 122 (8), 121 (46), 105 (8), 81 (18), 77 (12), 73 (10), 69 (10), 57 (10), 55 (12), 41 (13); **HRMS** (CIP) *m*/*z*: Calculated for $C_{34}H_{51}O_3Si$ [M+H]$^+$ 535.36020, found: 1. isomer 535.35999, 2. isomer 535.35986, 3. isomer 535.36066, 4. isomer 535.36017.

8.3.5 Methyl (3*R*,12*S*)-3,7-diethyl-12-hydroxy-11-methyltrideca-6,10-dienoate (**s7**)

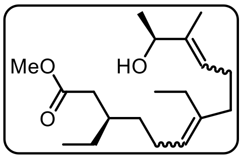

Tetrabutylammonium fluoride (TBAF, 1 M in THF with 5 % water, 0.30 ml, 0.300 mmol, 1.5 eq.) was added dropwise to a solution of methyl (3*R*,12*S*)-12-((*tert*-butyldiphenylsilyl)oxy)-3,7-diethyl-11-methyltrideca-6,10-dienoate (**41**, 110 mg, 0.200 mmol, 1.0 eq.) in THF (4 ml) at 0 °C.[23] The reaction

mixture was stirred at 40 °C for two days, and then quenched with saturated aqueous $NaHCO_3$ solution (10 ml). Diethyl ether (20 ml) was added, the phases were separated, and the aqueous phase was extracted with diethyl ether (3×20 ml). The combined organic phases were then dried over $MgSO_4$, filtered, and the solvent was removed under reduced pressure. The residue was purified by column chromatography (pentane/diethyl ether 2:1) to yield methyl (3*R*,12*S*)-3,7-diethyl-12-hydroxy-11-methyltrideca-6,10-dienoate (**s7**) as a colourless liquid (58.3 mg, 0.188 mmol, 94 %).

(3*S*,12*S*)-**s7'** (55.9 mg, 0.180 mmol, 99 %) was obtained using methyl (3*S*,12*S*)-12-((*tert*-butyldiphenylsilyl)oxy)-3,7-diethyl-11-methyltri-deca-6,10-dienoate (**41'**) and (3*S*,12*R*)-**s7** (37.3 mg, 0.120 mmol, 76 %) using methyl (3*S*,12*R*)-12-((*tert*-butyldiphenylsilyl)oxy)-3,7-diethyl-11-methyltri-deca-6,10-dienoate (**41**) under the same conditions as colourless liquids.

R_f = 0.23 (Pe/Et_2O 2:1); **^{1}H NMR** (300 MHz, $CDCl_3$): δ [ppm] = isomeric mixture 5.46 – 5.33 (m, 2H, =CH), 5.23 – 5.01 (m, 6H, =CH), 4.77 (q, *J* = 6.4 Hz, 1H, CH), 4.75 (q, *J* = 6.4 Hz, 1H, CH), 4.20 (q, *J* = 6.3 Hz, 2H, CH), 3.66 (s, 12H, CH_3), 2.26 (d, *J* = 6.7 Hz, 8H, CH_2), 2.14 – 1.93 (m, 32H, 4×CH_2), 1.83 (sept, *J* = 6.5 Hz, 4H, CH), 1.72 – 1.69 (m, 6H, CH_3), 1.64 – 1.61 (m, 6H, CH_3), 1.58 (br. s, 2H, OH), 1.54 (br. s, 2H, OH), 1.47 – 1.22 (m, 16H, 2×CH_2), 1.25 (d, *J* = 6.4 Hz, 3H, CH_3), 1.24 (d, *J* = 6.4 Hz, 3H, CH_3), 1.24 (d, *J* = 6.5 Hz, 6H, CH_3), 1.02 – 0.92 (m, 12H, CH_3), 0.88 (t, *J* = 7.4 Hz, 12H, CH_3); **^{13}C NMR** (75 MHz, $CDCl_3$): δ [ppm] = isomeric mixture 174.1 (2C, C_q), 174.0 (2C, C_q), 140.7 (C_q), 140.6 (2C, C_q), 140.6 (C_q), 138.6 (C_q), 138.5 (C_q), 138.1 (C_q), 137.8 (C_q), 126.5 (CH), 126.4 (CH), 124.8 (CH), 124.7 (CH), 124.3 (CH), 124.1 (CH), 123.8 (CH), 123.6 (CH), 73.3 (2C, CH), 65.6 (CH), 65.3 (CH), 51.4 (4C, CH_3), 38.5 (2C, CH_2), 38.5 (2C, CH_2), 36.6 (CH_2), 36.3 (CH), 36.3 (CH), 36.2 (CH), 36.1 (CH), 36.0 (CH_2), 33.8 (2C, CH_2), 33.8 (2C, CH_2), 30.5 (CH_2), 30.0 (CH_2), 29.5 (CH_2), 29.4 (CH_2), 26.5 (CH_2), 26.3 (CH_2), 26.2 (CH_2), 26.2 (2C, CH_2), 26.2 (CH_2), 26.1 (CH_2), 26.0 (CH_2), 24.9 (CH_2), 24.8 (CH_2), 24.6 (2C, CH_2), 23.1 (CH_2), 23.1 (CH_2), 21.6 (CH_3), 21.6 (CH_3), 21.2 (CH_3), 21.1 (CH_3), 17.1 (CH_3), 17.0 (CH_3), 13.2 (2C, CH_3), 12.8 (CH_3), 12.8 (CH_3), 11.4 (CH_3), 11.4 (CH_3), 10.8 (CH_3), 10.7 (CH_3), 10.7 (2C, CH_3); **IR** (GC/FTIR): $\tilde{\nu}$ [cm^{-1}] = 1. and 2. isomer 3288 (br. s), 2963 (s), 2930 (s), 2876 (m), 1738 (s), 1459 (m), 1438 (m), 1368 (m), 1311 (w), 1286 (w), 1255 (w), 1194 (m), 1160 (m), 1081 (m), 1013 (w), 958 (w), 895 (w), 858 (w); 3. and 4. isomer 3276 (br. s), 2964 (s), 2930 (s), 2874 (m), 1738 (s), 1438 (m), 1369 (m), 1285 (w), 1193 (m), 1154 (m), 1081 (m), 1011 (w), 958 (w), 896 (w), 855 (w); **EI-MS** (70 eV): *m*/*z* (%) = 1. isomer 292 (5) [M–H_2O]$^+$, 281 (11), 263 (10), 231 (8), 210 (8), 203 (10), 181 (8), 163 (11), 149 (24), 137 (37), 136 (39), 135 (33), 121 (28), 109 (22), 107 (39), 98 (20), 95 (45), 93 (28), 83 (36), 82 (36), 81 (47), 78 (29), 69 (29), 67 (42), 55 (65), 43 (100), 41 (45); 2. isomer 292 (4) [M–H_2O]$^+$, 263 (5), 231 (4), 210 (6), 203 (10), 181 (10), 163 (13), 149 (13), 137 (35), 136 (42), 135 (20), 121 (18), 109 (28), 107 (29), 95 (40), 93 (16), 83 (40), 82 (33), 81 (39), 79 (20), 69 (27), 67 (40), 59 (25), 55

(58), 43 (100), 41 (42); 3. isomer 292 (5) [M–H_2O]$^+$, 263 (4), 231 (6), 210 (10), 203 (9), 181 (8), 163 (8), 149 (27), 137 (42), 136 (44), 135 (39), 121 (32), 109 (29), 107 (44), 98 (26), 95 (60), 93 (25), 83 (54), 82 (39), 81 (47), 79 (26), 69 (40), 67 (53), 55 (87), 43 (100), 41 (67); 4. isomer 292 (3) [M–H_2O]$^+$, 281 (2), 263 (4), 231 (2), 210 (19), 203 (4), 181 (8), 163 (4), 149 (19), 137 (48), 136 (35), 135 (18), 121 (17), 109 (26), 107 (28), 95 (46), 93 (14), 83 (42), 82 (34), 81 (42), 79 (25), 69 (29), 67 (45), 55 (66), 43 (100), 41 (45); **HRMS** (EI) *m*/*z*: Calculated for $C_{19}H_{34}O_3$ [M]$^+$ 310.25025, found: 1. isomer 310.25000, 2. isomer 310.24957, 3. isomer 310.24997, 4. Isomer 310.25009.

(3*S*,12*S*)-**s7'**:

R$_f$ = 0.23 (Pe/Et_2O 2:1); **^{1}H NMR** (300 MHz, $CDCl_3$): *δ* [ppm] = isomeric mixture 5.45 – 5.34 (m, 2H, =CH), 5.22 – 5.02 (m, 6H, =CH), 4.81 – 4.71 (m, 2H, CH), 4.20 (q, *J* = 6.4 Hz, 2H, CH), 3.66 (s, 12H, CH_3), 2.26 (d, *J* = 6.8 Hz, 8H, CH_2), 2.18 – 1.93 (m, 32H, 4×CH_2), 1.90 – 1.75 (m, 4H, CH), 1.72 – 1.69 (m, 6H, CH_3), 1.65 – 1.60 (m, 6H, CH_3), 1.68 – 1.52 (br. s, 4H, OH), 1.47 – 1.22 (m, 16H, 2×CH_2), 1.25 (d, *J* = 6.4 Hz, 3H, CH_3), 1.24 (d, *J* = 6.4 Hz, 3H, CH_3), 1.24 (d, *J* = 6.5 Hz, 6H, CH_3), 1.02 – 0.84 (m, 24H, 2×CH_3); **^{13}C NMR** (75 MHz, $CDCl_3$): *δ* [ppm] = isomeric mixture 174.0 (2C, C_q), 174.0 (2C, C_q), 140.7 (2C, C_q), 140.6 (2C, C_q), 138.6 (C_q), 138.5 (C_q), 138.1 (C_q), 137.8 (C_q), 126.5 (CH), 126.3 (CH), 124.8 (CH), 124.7 (CH), 124.3 (CH), 124.0 (CH), 123.8 (CH), 123.6 (CH), 73.3 (2C, CH), 65.6 (CH), 65.3 (CH), 51.4 (4C, CH_3), 38.5 (2C, CH_2), 38.5 (2C, CH_2), 36.6 (CH_2), 36.3 (CH), 36.3 (CH), 36.1 (CH), 36.1 (CH), 36.0 (CH_2), 33.8 (CH_2), 33.8 (CH_2), 33.8 (CH_2), 33.8 (CH_2), 30.5 (CH_2), 30.0 (CH_2), 29.5 (CH_2), 29.4 (CH_2), 29.1 (CH_2), 26.5 (CH_2), 26.3 (CH_2), 26.2 (CH_2), 26.2 (CH_2), 26.2 (CH_2), 26.2 (CH_2), 25.9 (CH_2), 24.9 (CH_2), 24.9 (CH_2), 24.6 (CH_2), 24.6 (CH_2), 23.1 (2C, CH_2), 21.6 (CH_3), 21.6 (CH_3), 21.1 (CH_3), 21.0 (CH_3), 17.1 (CH_3), 17.0 (CH_3), 13.2 (CH_3), 13.2 (CH_3), 12.8 (CH_3), 12.8 (CH_3), 11.5 (CH_3), 11.4 (CH_3), 10.8 (CH_3), 10.7 (CH_3), 10.7 (2C, CH_3); **IR** (GC/FTIR): $\tilde{\nu}$ [cm^{-1}] = 1. and 2. isomer 3307 (br. s), 2964 (s), 2936 (s), 2877 (m), 2860 (m), 1739 (s), 1460 (m), 1438 (m), 1369 (m), 1310 (m), 1285 (m), 1256 (m), 1194 (m), 1161 (m), 1081 (m), 1029 (w), 959 (w), 894 (w), 859 (w); 3. and 4. isomer 3320 (br. s), 2966 (s), 2935 (s), 2876 (m), 1739 (s), 1457 (m), 1439 (m), 1370 (m), 1313 (m), 1255 (m), 1195 (m), 1155 (m), 1081 (m), 1017 (w), 957 (w), 896 (w), 851 (w); **EI-MS** (70 eV): *m*/*z* (%) = 1. isomer 292 (4) [M–H_2O]$^+$, 263 (5), 231 (8), 210 (7), 203 (12), 189 (6), 181 (6), 163 (11), 149 (28), 137 (31), 136 (54), 135 (31), 121 (22), 109 (26), 107 (36), 98 (25), 95 (42), 93 (18), 83 (42), 82 (33), 81 (36), 69 (31), 67 (47), 55 (64), 43 (100), 41 (42); 2. isomer 292 (5) [M–H_2O]$^+$, 263 (5), 231 (5), 210 (6), 203 (11), 189 (3), 181 (9), 163 (12), 149 (15), 137 (41), 136 (44), 135 (21), 121 (19), 109 (27), 107 (31), 98 (8), 95 (43), 93 (17), 83 (38), 82 (34), 81 (40), 69 (26), 67 (42), 55 (64), 43 (100), 41 (38); 3. isomer 292 (7) [M–H_2O]$^+$, 263 (10), 231 (5), 210 (6), 203 (9), 189 (6), 181 (7), 163 (12), 149 (29), 137 (50), 136 (42), 135 (33), 121 (24), 109 (29), 107 (38), 98 (25), 95 (52), 93 (24), 83 (51), 82 (38), 81

(46), 69 (34), 67 (57), 55 (76), 43 (100), 41 (51); 4. isomer 292 (6) $[M–H_2O]^+$, 263 (7), 231 (3), 210 (23), 203 (4), 189 (4), 181 (9), 163 (7), 149 (20), 137 (57), 136 (46), 135 (25), 121 (18), 109 (33), 107 (30), 98 (9), 95 (51), 93 (18), 83 (45), 82 (38), 81 (48), 69 (29), 67 (47), 55 (67), 43 (100), 41 (42); **HRMS** (CIP) *m*/*z*: Calculated for $C_{19}H_{33}O_3$ $[M–H]^+$ 309.24242, found: 1. isomer 309.24240, 2. isomer 309.24261, 3. isomer 309.24222, 4. isomer 309.24197.

8.3.6 Methyl (3*R*,12*S*)-12-hydroxy-3,7,11-trimethyltrideca-6,10-dienoate (**s8**)

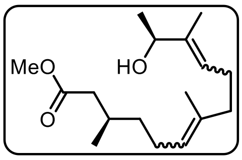

The silyl ether cleavage was carried out analogously to procedure 8.3.5 with TBAF (1 M in THF with 5 % water, 0.60 ml, 0.596 mmol, 1.5 eq.) and methyl (3*R*,12*S*)-12-((*tert*-butyldiphenylsilyl)oxy)-3,7,11-trimethyltrideca-6,10-dienoate (**44**, 207 mg, 0.397 mmol, 1.0 eq.) in THF (8 ml). The reaction mixture was stirred for 27 h after which more TBAF (1 M in THF with 5 % water, 0.20 ml, 0.199 mmol, 0.5 eq.) was added. After stirring for a further 22 h at 40 °C, methyl (3*R*,12*S*)-12-hydroxy-3,7,11-trimethyltrideca-6,10-dienoate (**s8**) was obtained as a colourless liquid (95.5 mg, 0.338 mmol, 85 %).

$\mathbf{R_f}$ = 0.19 (Pe/Et_2O 2:1); **^{1}H NMR** (300 MHz, $CDCl_3$): δ [ppm] = isomeric mixture 5.43 – 5.33 (m, 2H, =CH), 5.21 – 5.06 (m, 6H, =CH), 4.81 – 4.71 (m, 2H, CH), 4.24 – 4.15 (m, 2H, CH), 3.67 (s, 12H, CH_3), 2.36 – 2.27 (m, 4H, CH_aH_b), 2.18 – 1.89 (m, 32H, CH, CH_aH_b, 3×CH_2), 1.78 – 1.50 (m, 4H, OH), 1.72 – 1.69 (m, 6H, CH_3), 1.69 – 1.67 (m, 6H, CH_3), 1.65 – 1.61 (m, 6H, CH_3), 1.61 – 1.58 (m, 6H, CH_3), 1.44 – 1.13 (m, 8H, CH_2), 1.25 (d, *J* = 6.4 Hz, 3H, CH_3), 1.24 (d, *J* = 6.4 Hz, 3H, CH_3), 1.24 (d, *J* = 6.5 Hz, 6H, CH_3), 0.94 (d, *J* = 6.6 Hz, 12H, CH_3); **^{13}C NMR** (75 MHz, $CDCl_3$): δ [ppm] = isomeric mixture 173.8 (2C, C_q), 173.7 (C_q), 173.7 (C_q), 138.7 (C_q), 138.6 (C_q), 138.2 (C_q), 137.8 (C_q), 135.0 (C_q), 134.9 (C_q), 134.8 (C_q), 134.7 (C_q), 126.3 (CH), 126.3 (CH), 125.5 (CH), 125.2 (CH), 124.6 (CH), 124.6 (CH), 124.6 (CH), 124.4 (CH), 73.3 (2C, CH), 65.6 (CH), 65.3 (CH), 51.4 (4C, CH_3), 41.5 (2C, CH_2), 41.5 (2C, CH_2), 39.8 (CH_2), 39.2 (CH_2), 37.0 (CH_2), 36.9 (CH_2), 36.6 (2C, CH_2), 32.0 (CH_2), 31.5 (CH_2), 30.0 (CH), 30.0 (CH), 30.0 (CH), 29.9 (CH), 26.0 (CH_2), 25.9 (CH_2), 25.7 (CH_2), 25.6 (CH_2), 25.2 (CH_2), 25.2 (2C, CH_2), 25.2 (CH_2), 23.4 (CH_3), 23.3 (CH_3), 21.6 (2C, CH_3), 21.1 (CH_3), 21.0 (CH_3), 19.6 (CH_3), 19.6 (CH_3), 19.6 (2C, CH_3), 17.1 (CH_3), 17.0 (CH_3), 16.0 (CH_3), 15.9 (CH_3), 11.4 (CH_3), 11.4 (CH_3); **IR** (GC/FTIR): $\tilde{\nu}$ [cm^{-1}] = 1. isomer 3303 (br. s), 2966 (s), 2921 (m), 2853 (m), 1739 (s), 1439 (m), 1378 (m), 1292 (w), 1201 (m), 1163 (m), 1111 (m), 1076 (m), 1036 (m), 1014 (w), 903 (w), 840 (w); 2. isomer 3286 (br. s), 2963 (s), 2929 (m), 2872 (m), 2855 (m), 1739 (s), 1440 (m), 1371 (m), 1291 (m), 1264 (w), 1201 (m), 1163 (m), 1083 (m), 1031 (w), 1011 (w), 899 (w), 857 (w); 3. isomer 3282 (br. s), 2968 (s), 2920 (m), 2850 (m), 1739 (s), 1439 (m), 1378 (m), 1292 (m), 1264 (w), 1200 (m), 1157 (m), 1096 (m), 1074 (w), 1036 (m), 1013 (w), 902 (w), 842 (w); 4. isomer 3287 (br. s), 2967 (m), 2928 (m), 2851 (m), 1739 (s), 1439 (m),

1370 (m), 1291 (m), 1199 (m), 1156 (m), 1083 (m), 1011 (w), 958 (w), 898 (w), 873 (w); **EI-MS** (70 eV): *m/z* (%) = 1. isomer 281 (2) $[M–H]^+$, 264 (5) $[M–H_2O]^+$, 235 (4), 203 (3), 182 (7), 175 (8), 163 (5), 149 (10), 135 (24), 122 (37), 121 (48), 109 (59), 108 (37), 107 (30), 95 (34), 93 (36), 91 (20), 82 (31), 81 (34), 79 (24), 69 (41), 67 (48), 59 (23), 55 (44), 43 (100), 41 (40); 2. isomer 281 (1) $[M–H]^+$, 264 (9) $[M–H_2O]^+$, 235 (6), 203 (4), 182 (13), 175 (10), 163 (5), 149 (12), 135 (20), 122 (43), 121 (43), 109 (83), 108 (43), 107 (26), 95 (34), 93 (33), 91 (17), 82 (34), 81 (45), 79 (28), 69 (48), 67 (46), 59 (25), 55 (42), 43 (100), 41 (38); 3. isomer 281 (1) $[M–H]^+$, 264 (9) $[M–H_2O]^+$, 235 (6), 203 (3), 182 (10), 175 (7), 163 (5), 149 (10), 135 (33), 122 (38), 121 (53), 109 (87), 108 (36), 107 (35), 95 (37), 93 (38), 91 (20), 82 (33), 81 (41), 79 (28), 69 (60), 67 (51), 59 (27), 55 (45), 43 (100), 41 (43); 4. isomer 281 (1) $[M–H]^+$, 264 (8) $[M–H_2O]^+$, 235 (6), 203 (3), 182 (41), 175 (4), 163 (4), 149 (6), 135 (28), 122 (39), 121 (37), 109 (100), 108 (39), 107 (30), 95 (34), 93 (33), 91 (19), 82 (35), 81 (43), 79 (28), 69 (60), 67 (50), 59 (28), 55 (45), 43 (98), 41 (41); **HRMS** (CIP) *m/z*: Calculated for $C_{17}H_{29}O_3$ $[M–H]^+$ 281.21112, found: 1. isomer 281.21118, 2. isomer 281.21057, 3. isomer 281.21045, 4. isomer 281.21100.

8.3.7 Methyl (3*R*,12*S*)-7-ethyl-12-hydroxy-3,11-dimethyltrideca-6,10-dienoate (**s9**)

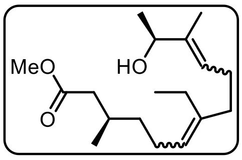

The silyl ether cleavage was carried out analogously to procedure 8.3.5 with TBAF (1 M in THF with 5 % water, 0.21 ml, 0.214 mmol, 1.5 eq.) and methyl (3*R*,12*S*)-12-((*tert*-butyldiphenylsilyl)oxy)-7-ethyl-3,11-dimethyltrideca-6,10-dienoate (**45**, 76.3 mg, 0.143 mmol, 1.0 eq.) in THF (3 ml). The reaction mixture was stirred for 22 h after which more TBAF (1 M in THF with 5 % water, 0.07 ml, 71.5 µmol, 0.5 eq.) was added. After stirring for a further 7 h at 40 °C, methyl (3*R*,12*S*)-7-ethyl-12-hydroxy-3,11-dimethyltrideca-6,10-dienoate (**s9**) was obtained as a colourless liquid (34.4 mg, 0.116 mmol, 81 %).

$\mathbf{R_f}$ = 0.20 (Pe/Et_2O 2:1); **^{1}H NMR** (300 MHz, $CDCl_3$): δ [ppm] = isomeric mixture 5.45 – 5.34 (m, 2H, =CH), 5.23 – 5.01 (m, 6H, =CH), 4.76 (q, *J* = 6.6 Hz, 2H, CH), 4.25 – 4.14 (m, 2H, CH), 3.67 (s, 12H, CH_3), 2.34 (d, *J* = 6.0 Hz, 2H, CH_aH_b), 2.30 (d, *J* = 6.0 Hz, 2H, CH_aH_b), 2.18 – 1.90 (m, 40H, CH, CH_aH_b, 4×CH_2), 1.78 – 1.51 (m, 4H, OH), 1.72 – 1.69 (m, 6H, CH_3), 1.64 – 1.61 (m, 6H, CH_3), 1.43 – 1.16 (m, 8H, CH_2), 1.25 (d, *J* = 6.4 Hz, 3H, CH_3), 1.24 (d, *J* = 6.4 Hz, 3H, CH_3), 1.24 (d, *J* = 6.5 Hz, 6H, CH_3), 1.02 – 0.92 (m, 24H, 2×CH_3); **^{13}C NMR** (75 MHz, $CDCl_3$): δ [ppm] = isomeric mixture 173.8 (C_q), 173.7 (C_q), 173.7 (2C, C_q), 140.7 (C_q), 140.6 (2C, C_q), 140.6 (C_q), 138.6 (C_q), 138.5 (C_q), 138.1 (C_q), 137.8 (C_q), 126.5 (CH), 126.4 (CH), 124.8 (CH), 124.7 (CH), 124.1 (CH), 123.9 (CH), 123.7 (CH), 123.4 (CH), 73.3 (2C, CH), 65.6 (CH), 65.3 (CH), 51.4 (4C, CH_3), 41.6 (2C, CH_2), 41.5 (2C, CH_2), 37.1 (CH_2), 37.0 (CH_2), 37.0 (2C, CH_2), 36.6 (CH_2), 36.0 (CH_2), 30.5 (CH_2), 30.1 (CH), 30.1 (CH), 30.0 (CH), 30.0 (CH_2), 29.9 (CH), 29.5 (CH_2), 29.4 (CH_2), 26.5 (CH_2), 26.1 (CH_2), 26.1 (CH_2), 25.9 (CH_2), 25.2

(CH_2), 25.1 (CH_2), 24.9 (CH_2), 24.9 (CH_2), 23.1 (CH_2), 23.1 (CH_2), 21.6 (CH_3), 21.6 (CH_3), 21.1 (CH_3), 21.1 (CH_3), 19.7 (CH_3), 19.7 (CH_3), 19.6 (2C, CH_3), 17.1 (CH_3), 17.0 (CH_3), 13.2 (CH_3), 13.2 (CH_3), 12.8 (CH_3), 12.8 (CH_3), 11.5 (CH_3), 11.4 (CH_3); **IR** (GC/FTIR): $\tilde{\nu}$ [cm^{-1}] = 1. isomer 3281 (br. s), 2964 (s), 2933 (m), 2877 (m), 2851 (m), 1740 (s), 1460 (m), 1439 (m), 1372 (m), 1292 (m), 1200 (m), 1162 (m), 1113 (m), 1081 (m), 1037 (m), 1008 (w), 902 (w), 849 (w); 2. isomer 3279 (br. s), 2963 (s), 2932 (m), 2874 (m), 1740 (s), 1460 (m), 1439 (m), 1370 (m), 1316 (w), 1291 (m), 1264 (w), 1199 (m), 1160 (m), 1113 (w), 1082 (m), 1036 (w), 1009 (w), 957 (w), 899 (w), 849 (w); 3. isomer 3296 (br. s), 2964 (s), 2932 (m), 2874 (m), 1740 (s), 1460 (m), 1439 (m), 1370 (m), 1316 (w), 1291 (m), 1264 (w), 1199 (m), 1160 (m), 1113 (w), 1082 (m), 1037 (w), 1009 (w), 957 (w), 899 (w), 849 (w); 4. isomer 3300 (br. s), 2964 (s), 2931 (m), 2874 (m), 1740 (s), 1459 (m), 1439 (m), 1370 (m), 1316 (w), 1291 (m), 1265 (w), 1215 (m), 1198 (m), 1156 (m), 1112 (w), 1083 (m), 1010 (w), 957 (w), 898 (w), 857 (w); **EI-MS** (70 eV): *m*/*z* (%) = 1. isomer 296 (<1) $[M]^+$, 278 (14) $[M–H_2O]^+$, 249 (13), 189 (10), 163 (12), 149 (35), 136 (29), 135 (60), 123 (55), 122 (43), 121 (35), 107 (62), 95 (53), 93 (50), 91 (32), 83 (38), 82 (35), 81 (64), 79 (48), 69 (42), 67 (61), 59 (36), 55 (72), 43 (100), 41 (63); 2. isomer 296 (<1) $[M]^+$, 278 (12) $[M–H_2O]^+$, 249 (12), 189 (9), 163 (12), 149 (27), 136 (31), 135 (55), 123 (73), 122 (47), 121 (32), 107 (54), 95 (58), 93 (49), 91 (29), 83 (43), 82 (36), 81 (87), 79 (58), 69 (42), 67 (66), 59 (38), 55 (78), 43 (100), 41 (67); 3. isomer 296 (<1) $[M]^+$, 278 (24) $[M–H_2O]^+$, 249 (23), 189 (12), 163 (12), 149 (47), 136 (33), 135 (69), 123 (88), 122 (46), 121 (41), 107 (63), 95 (63), 93 (56), 91 (35), 83 (53), 82 (38), 81 (85), 79 (59), 69 (47), 67 (67), 59 (40), 55 (80), 43 (100), 41 (65); 4. isomer 296 (<1) $[M]^+$, 278 (13) $[M–H_2O]^+$, 249 (14), 189 (6), 163 (7), 149 (39), 136 (25), 135 (48), 123 (81), 122 (41), 121 (34), 107 (55), 95 (59), 93 (51), 91 (34), 83 (51), 82 (36), 81 (82), 79 (56), 69 (47), 67 (65), 59 (41), 55 (83), 43 (100), 41 (71); **HRMS** (EI) *m*/*z*: Calculated for $C_{18}H_{30}O_2$ $[M–H_2O]^+$ 278.22403, found: 1. isomer 278.22446, 2. isomer 278.22430, 3. isomer 278.22446, 4. isomer 278.22397.

8.3.8 Methyl (3*R*,12*S*)-3-ethyl-12-hydroxy-7,11-dimethyltrideca-6,10-dienoate (**s10**)

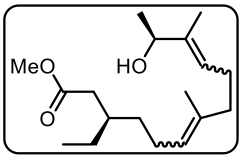

The silyl ether cleavage was carried out analogously to procedure 8.3.5 with TBAF (1 M in THF with 5 % water, 0.08 ml, 78.9 µmol, 1.5 eq.) and methyl (3*R*,12*S*)-12-((*tert*-butyldiphenylsilyl)oxy)-3-ethyl-7,11-dimethyltrideca-6,10-dienoate (**46**, 28.1 mg, 52.6 µmol, 1.0 eq.) in THF (1.5 ml). After stirring for 16 h at 40 °C, methyl (3*R*,12*S*)-3-ethyl-12-hydroxy-7,11-dimethyltrideca-6,10-dienoate (**s10**) was obtained as a colourless liquid (13.1 mg, 44.2 µmol, 84 %).

$\mathbf{R_f}$ = 0.20 (Pe/Et_2O 2:1); **^{1}H NMR** (300 MHz, $CDCl_3$): δ [ppm] = isomeric mixture 5.44 – 5.33 (m, 2H, =CH), 5.22 – 5.06 (m, 6H, =CH), 4.82 – 4.71 (m, 2H, CH), 4.25 – 4.15 (m, 2H, CH), 3.66 (s, 6H, CH_3), 3.66 (s, 6H, CH_3), 2.26 (d, *J* = 7.0 Hz, 8H, CH_2), 2.23 – 1.89 (m, 24H, 3×CH_2),

1.89 – 1.74 (m, 4H, CH), 1.72 – 1.69 (m, 6H, CH_3), 1.70 – 1.67 (m, 6H, CH_3), 1.65 – 1.61 (m, 6H, CH_3), 1.61 – 1.58 (m, 6H, CH_3), 1.56 (br. s, 2H, OH), 1.50 (br. s, 2H, OH), 1.45 – 1.25 (m, 16H, 2×CH_2), 1.24 (d, J = 6.4 Hz, 6H, CH_3), 1.24 (d, J = 6.5 Hz, 3H, CH_3), 1.23 (d, J = 6.4 Hz, 3H, CH_3), 0.88 (t, J = 7.4 Hz, 12H, CH_3); **^{13}C NMR** (75 MHz, $CDCl_3$): δ [ppm] = isomeric mixture 174.1 (C_q), 174.1 (C_q), 174.1 (C_q), 174.0 (C_q), 138.7 (C_q), 138.6 (C_q), 138.2 (C_q), 137.8 (C_q), 134.9 (C_q), 134.9 (C_q), 134.7 (C_q), 134.7 (C_q), 126.4 (CH), 126.3 (CH), 125.6 (CH), 125.3 (CH), 124.8 (CH), 124.7 (CH), 124.6 (CH), 124.5 (CH), 73.3 (2C, CH), 65.6 (CH), 65.4 (CH), 51.4 (4C, CH_3), 39.8 (CH_2), 39.3 (2C, CH_2), 38.5 (3C, CH_2), 36.2 (CH), 36.2 (CH), 36.1 (CH), 36.0 (CH), 33.7 (2C, CH_2), 33.4 (2C, CH_2), 32.1 (CH_2), 31.5 (CH_2), 26.3 (CH_2), 26.2 (CH_2), 26.2 (2C, CH_2), 26.0 (CH_2), 25.9 (CH_2), 25.7 (CH_2), 25.6 (CH_2), 25.0 (CH_2), 25.0 (2C, CH_2), 24.9 (CH_2), 23.4 (CH_3), 23.3 (CH_3), 21.6 (2C, CH_3), 21.1 (CH_3), 21.0 (CH_3), 17.1 (CH_3), 17.0 (CH_3), 16.0 (CH_3), 15.9 (CH_3), 11.4 (2C, CH_3), 10.7 (CH_3), 10.7 (2C, CH_3), 10.7 (CH_3); **IR** (GC/FTIR): $\tilde{\nu}$ [cm^{-1}] = 1. isomer 3289 (br. s), 2968 (s), 2920 (s), 2859 (m), 1739 (s), 1439 (m), 1377 (m), 1285 (w), 1256 (w), 1196 (m), 1163 (m), 1098 (m), 1076 (m), 1036 (m), 1015 (w), 903 (w), 845 (w); 2. isomer 3291 (br. s), 2964 (s), 2929 (m), 2878 (m), 2860 (m), 1739 (s), 1440 (m), 1376 (m), 1312 (w), 1286 (w), 1258 (w), 1196 (m), 1163 (m), 1082 (m), 1031 (w), 1016 (w), 957 (w), 900 (w), 851 (w); 3. isomer 3296 (br. s), 2968 (s), 2920 (s), 2857 (m), 1739 (s), 1439 (m), 1379 (m), 1285 (w), 1254 (w), 1195 (m), 1181 (m), 1156 (m), 1097 (m), 1073 (m), 1036 (m), 1016 (w), 903 (w); 4. isomer 3291 (br. s), 2967 (s), 2920 (m), 2879 (m), 2856 (m), 1739 (s), 1440 (m), 1383 (w), 1370 (w), 1345 (w), 1286 (w), 1256 (w), 1195 (m), 1181 (w), 1156 (m), 1083 (m), 1027 (w), 1016 (w), 957 (w), 898 (w), 850 (w); **EI-MS** (70 eV): m/z (%) = 1. isomer 278 (14) $[M–H_2O]^+$, 249 (2), 196 (2), 149 (23), 136 (25), 135 (28), 123 (63), 122 (66), 121 (35), 98 (32), 95 (44), 93 (25), 83 (26), 82 (42), 81 (28), 79 (44), 77 (29), 69 (56), 67 (63), 55 (74), 43 (100), 41 (31); 2. isomer 278 (11) $[M–H_2O]^+$, 249 (11), 196 (19), 149 (19), 136 (30), 123 (84), 122 (64), 121 (37), 109 (29), 107 (27), 95 (43), 93 (36), 83 (25), 82 (42), 81 (52), 79 (19), 69 (46), 67 (40), 55 (49), 43 (100), 41 (39); 3. isomer 278 (8) $[M–H_2O]^+$, 249 (11), 196 (13), 136 (46), 135 (43), 123 (100), 122 (80), 121 (74), 107 (45), 98 (39), 95 (46), 94 (34), 93 (50), 82 (44), 81 (62), 79 (35), 69 (58), 67 (65), 55 (49), 43 (97), 41 (49); 4. isomer 278 (7) $[M–H_2O]^+$, 249 (5), 196 (66), 136 (26), 135 (25), 123 (100), 122 (65), 121 (38), 109 (26), 107 (30), 95 (34), 93 (34), 83 (25), 82 (45), 81 (63), 79 (23), 69 (58), 67 (40), 55 (48), 43 (87), 41 (32); **HRMS** (CIP) m/z: Calculated for $C_{18}H_{31}O_3$ $[M–H]^+$ 295.22677, found: 1. isomer 295.22641, 2. isomer 295.22684, 3. isomer 295.22684, 4. isomer 295.22659.

8.3.9 (3*R*,6*E*,10*E*,12*S*)-3,7-Diethyl-11-methyl-6,10-tridecadien-12-olide (**9**)

Lithium hydroxide (21.2 mg, 0.885 mmol, 5.0 eq.) was added to a solution of methyl (3*R*,12*S*)-3,7-diethyl-12-hydroxy-11-methyltrideca-6,10-dienoate (**s7**, 55.0 mg, 0.177 mmol, 1.0 eq.) in

THF/H_2O 1:1 (0.8 ml) at room temperature and stirred for 24 h at 40 °C.[24] After the reaction was complete, the mixture was acidified with aqueous HCl solution (1 M, 2 ml), diethyl ether (10 ml) was added, and the phases were separated. The aqueous phase was extracted with diethyl ether (3×10 ml), the combined organic phases were dried over $MgSO_4$, filtered, and the solvent was removed under reduced pressure. (3*R*,12*S*)-3,7-diethyl-12-hydroxy-11-methyltrideca-6,10-dienoic acid (**24**) was obtained as a light-yellow liquid (52.4 mg, 0.177 mmol, 100 %) and was used in the next synthesis step without further purification.

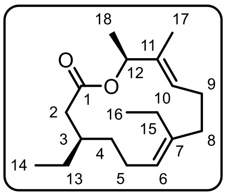

Triethylamine (14 µl, 0.102 mmol, 3.0 eq.) and 2,4,6-trichlorobenzoyl chloride (TCBC, 16 µl, 0.102 mmol, 3.0 eq.) were dissolved separately in a little THF (each 0.20 ml) and added sequentially at 0 °C to a solution of (3*R*,12*S*)-3,7-diethyl-12-hydroxy-11-methyltrideca-6,10-dienoic acid (**24**, 10.0 mg, 34.0 µmol, 1.0 eq.) dissolved in THF (0.25 ml).[25] The cloudy reaction mixture was stirred at room temperature for two hours and then diluted with toluene (6.5 ml). In another flask, 4-(dimethylamino)pyridine (DMAP, 16.6 mg, 0.136 mmol, 4.0 eq.) was dissolved in toluene (17 ml) and heated to 50 °C. The mixed anhydride from the first flask was added dropwise to the DMAP solution over 2 h at 50 °C using a syringe pump. Stirring was continued for one more hour at 50 °C, the mixture was cooled to room temperature, and the reaction was quenched by adding saturated aqueous $NaHCO_3$ solution (5 ml). Diethyl ether (5 ml) was added, the phases were separated, and the aqueous phase was extracted with diethyl ether (3×5 ml). The combined organic phases were dried over $MgSO_4$, filtered, and the solvent was removed under reduced pressure. The residue was applied to silica gel and purified by column chromatography (pentane/diethyl ether 20:1) to afford the isomer mixture as a colourless liquid (9.0 mg, 32.3 µmol, 96 %). (3*R*,6*E*,10*E*,12*S*)-3,7-Diethyl-11-methyl-6,10-tridecadien-12-olide (**9**, 2.10 mg, 7.54 µmol, 22 %) was obtained by column chromatography with silver nitrate on silica gel (pentane/diethyl ether 100:1→50:1).

(3*S*,12*S*)-**9’** (mixed isomers: 25.9 mg, 93.0 µmol, 67 %; (*E*,*E*)-isomer: 1.35 mg, 4.85 µmol, 4 %) and (3*S*,12*R*)-**9** (mixed isomers: 17.8 mg, 63.9 µmol, 73 %; (*E*,*E*)-isomer: 1.81 mg, 6.50 µmol, 7 %) were obtained as colourless liquids under the same conditions using methyl (3*S*,12*S*)-3,7-diethyl-12-hydroxy-11-methyltrideca-6,10-dienoate (**s7’**) and methyl (3*S*,12*R*)-3,7-diethyl-12-hydroxy-11-methyltrideca-6,10-dienoate (**s7**).

R_f = 0.33 (Pe/Et_2O 20:1); $[\alpha]_D^{25}$ = +65.9 ± 3.0 (c = 0.135, CH_2Cl_2, (3*R*,12*S*)-**9**); –64.1 ± 2.2 (c = 0.181, CH_2Cl_2, (3*S*,12*R*)-**9**); **^{1}H NMR** (500 MHz, $CDCl_3$): *δ* [ppm] = 5.43 – 5.39 (m, 1H, 10-H), 5.36 (q, *J* = 6.6 Hz, 1H, 12-H), 5.01 (dd, *J* = 9.7, 5.5 Hz, 1H, 6-H), 2.26 – 2.21 (m, 1H, 2-H_a), 2.23 – 2.17 (m, 1H, 8-H_a), 2.17 – 2.09 (m, 1H, 9-H_a), 2.14 – 2.07 (m, 1H, 5-H_a), 2.12 – 2.04 (m, 1H, 15-H_a), 2.05 – 1.97 (m, 1H, 9-H_b), 1.97 – 1.90 (m, 1H, 2-H_b), 1.92 – 1.85 (m, 1H, 8-H_b), 1.90 – 1.81 (m, 1H, 15-H_b), 1.81 – 1.72 (m, 1H, 5-H_b), 1.74 – 1.67 (m, 1H, 3-H), 1.53 – 1.46

(m, 1H, 4-H_a), 1.52 – 1.50 (m, 3H, 17-H), 1.50 – 1.41 (m, 1H, 13-H_a), 1.27 (d, *J* = 6.7 Hz, 3H, 18-H), 1.27 – 1.18 (m, 1H, 13-H_b), 1.24 – 1.17 (m, 1H, 4-H_b), 0.94 (t, *J* = 7.6 Hz, 3H, 16-H), 0.91 (t, *J* = 7.4Hz, 3H, 14-H); **^{13}C NMR** (125 MHz, $CDCl_3$): *δ* [ppm] = 173.6 (C_q, 1-C), 137.9 (C_q, 7-C), 134.9 (C_q, 11-C), 129.1 (CH, 10-C), 126.2 (CH, 6-C), 75.4 (CH, 12-C), 40.3 (CH_2, 2-C), 37.3 (CH, 3-C), 35.4 (CH_2, 8-C), 34.2 (CH_2, 4-C), 28.9 (CH_2, 13-C), 25.0 (CH_2, 9-C), 24.8 (CH_2, 5-C), 22.1 (CH_2, 15-C), 18.4 (CH_3, 18-C), 13.4 (CH_3, 16-C), 11.8 (CH_3, 17-C), 11.4 (CH_3, 14-C); **IR** (GC/FTIR): $\tilde{\nu}$ [cm^{-1}] = 2963 (s), 2933 (s), 2861 (m), 1729 (s), 1458 (m), 1438 (m), 1378 (w), 1359 (w), 1338 (m), 1301 (w), 1257 (m), 1232 (m), 1181 (m), 1141 (m), 1109 (w), 1064 (m), 1044 (m), 1021 (w), 993 (w), 952 (w), 899 (w), 832 (m); **EI-MS** (70 eV): *m*/*z* (%) = 278 (4) $[M]^+$, 249 (5), 222 (9), 196 (47), 167 (24), 137 (46), 136 (43), 121 (12), 107 (44), 95 (30), 93 (13), 83 (34), 82 (75), 81 (34), 79 (28), 77 (11), 69 (16), 67 (100), 55 (43), 53 (20), 43 (25), 41 (55), 39 (14); **HRMS** (EI) *m*/*z*: Calculated for $C_{18}H_{30}O_2$ $[M]^+$ 278.22403, found: 278.22403; ***I*** (HP-5-MS): 1916.

Table 1: NMR data (500 MHz, $CDCl_3$) of (3*R*,12*S*)-**9**.

atom	^{1}H, δ [ppm]	^{13}C, δ [ppm]	COSY	NOESY	HMBC
1	-	173.6	-	-	2, 12
2	2.26 – 2.21, 1.97 – 1.90	40.3	3	3, 13, 14	3, 4, 13, 14
3	1.74 – 1.67	37.3	2, 13	2, 13, 14	2, 4, 5, 13, 14
4	1.53 – 1.46, 1.24 – 1.17	34.2	5	5	2, 3, 5, 6, 13
5	2.14 – 2.07, 1.81 – 1.72	24.8	4, 6	4, 6	3, 4, 6, 8
6	5.01	126.2	5, 8, 15	5, 8	4, 5, 8, 15
7	-	137.9	-	-	5, 8, 15, 16
8	2.23 – 2.17, 1.92 – 1.85	35.4	6	6, 10	5, 6, 9, 10, 15
9	2.17 – 2.09, 2.05 – 1.97	25.0	10, 17	17	8, 10
10	5.43 – 5.39	129.1	9, 12, 17	8, 12	8, 9, 12, 17
11	-	134.9	-	-	12, 17, 18
12	5.36	75.4	10, 17, 18	10, 17, 18	10, 17, 18
13	1.50 – 1.41, 1.27 – 1.18	28.9	3, 14	2, 14	2, 3, 4, 14
14	0.91	11.4	13	2, 3, 13	2, 3, 13
15	2.12 – 2.04,	22.1	6, 16	16	6, 8, 16

atom	^{1}H, δ [ppm]	^{13}C, δ [ppm]	COSY	NOESY	HMBC
	1.90 – 1.81				
16	0.94	13.4	15	15	15
17	1.52 – 1.50	11.8	9, 10, 12	9, 12	10, 12
18	1.27	18.4	12	12	12

(3*S*,12*S*)-**9'**:

R$_f$ = 0.33 (Pe/Et_2O 20:1); $[\alpha]_D^{25}$ = –30.7 ± 2.6 (c = 0.153, CH_2Cl_2); **^{1}H NMR** (600 MHz, $CDCl_3$): *δ* [ppm] = 5.33 – 5.28 (m, 1H, 10-H), 5.20 (q, *J* = 6.6 Hz, 1H, 12-H), 4.95 (t, *J* = 7.8 Hz, 1H, 6-H), 2.42 (dd, *J* = 12.0, 2.0 Hz, 1H, 2-H_a), 2.21 – 2.14 (m, 1H, 8-H_a), 2.20 – 2.07 (m, 2H, 9-H), 2.10 – 2.04 (m, 1H, 8-H_b), 2.01 – 1.95 (m, 2H, 5-H), 1.96 – 1.90 (m, 2H, 15-H), 1.77 (dd, *J* = 11.7, 11.7 Hz, 1H, 2-H_b), 1.63 – 1.61 (m, 3H, 17-H), 1.63 – 1.56 (m, 1H, 3-H), 1.63 – 1.56 (m, 1H, 13-H_a), 1.40 – 1.34 (m, 1H, 4-H_a), 1.33 – 1.27 (m, 1H, 4-H_b), 1.26 (d, *J* = 6.6 Hz, 3H, 18-H), 1.18 – 1.11 (m, 1H, 13-H_b), 0.95 (t, *J* = 7.5 Hz, 3H, 16-H), 0.92 (t, *J* = 7.2 Hz, 3H, 14-H); **^{13}C NMR** (150 MHz, $CDCl_3$): *δ* [ppm] = 172.5 (C_q, 1-C), 139.1 (C_q, 7-C), 135.2 (C_q, 11-C), 126.4 (CH, 6-C), 124.8 (CH, 10-C), 73.2 (CH, 12-C), 40.3 (CH_2, 2-C), 35.5 (CH_2, 8-C), 35.2 (CH, 3-C), 33.5 (CH_2, 4-C), 27.0 (CH_2, 13-C), 24.1 (CH_2, 5-C), 23.9 (CH_2, 9-C), 21.5 (CH_2, 15-C), 19.7 (CH_3, 18-C), 13.7 (CH_3, 17-C), 13.1 (CH_3, 16-C), 11.2 (CH_3, 14-C); **IR** (GC/FTIR): $\tilde{\nu}$ [cm^{-1}] = 2965 (s), 2934 (s), 2874 (m), 2859 (m), 1733 (s), 1458 (m), 1440 (m), 1380 (w), 1322 (w), 1299 (m), 1271 (m), 1237 (m), 1218 (m), 1181 (w), 1145 (m), 1073 (m), 1027 (w), 1010 (w), 946 (w), 869 (w), 834 (w); **EI-MS** (70 eV): *m*/*z* (%) = 278 (6) $[M]^+$, 249 (7), 222 (13), 196 (59), 167 (30), 137 (60), 136 (55), 121 (16), 107 (53), 95 (37), 93 (17), 83 (38), 82 (79), 81 (38), 79 (31), 77 (11), 69 (17), 67 (100), 55 (44), 53 (18), 43 (23), 41 (47), 39 (12); **HRMS** (EI) *m*/*z*: Calculated for $C_{18}H_{30}O_2$ $[M]^+$ 278.22403, found: 278.22421; ***I*** (HP-5-MS): 1892.

Table 2: NMR data (600 MHz, $CDCl_3$) of (3*S*,12*S*)-**9'**.

atom	^{1}H, δ [ppm]	^{13}C, δ [ppm]	COSY	NOESY	HMBC
1	-	172.5	-	-	2, 12
2	2.42, 1.77	40.3	3	3, 13, 14	3, 4, 13, 14
3	1.63 – 1.56	35.2	2, 13	2, 13, 14	2, 4, 5, 13, 14
4	1.40 – 1.34, 1.33 – 1.27	33.5	5	5	2, 3, 5, 6, 13
5	2.01 – 1.95	24.1	4, 6	4, 6	3, 4, 6, 8
6	4.95	126.4	5, 8, 15	5, 8	4, 5, 8, 15
7	-	139.1	-	-	5, 8, 15, 16
8	2.21 – 2.14,	35.5	6	6, 10	5, 6, 9, 10, 15

atom	^{1}H, δ [ppm]	^{13}C, δ [ppm]	COSY	NOESY	HMBC
	2.10 – 2.04				
9	2.20 – 2.07	23.9	10, 17	17	8, 10
10	5.33 – 5.28	124.8	9, 12, 17	8, 12	8, 9, 12, 17
11	-	135.2	-	-	12, 17, 18
12	5.20	73.2	10, 17, 18	10, 17, 18	10, 17, 18
13	1.63 – 1.56,	27.0	3, 14	2, 14	2, 3, 4, 14
	1.18 – 1.11				
14	0.92	11.2	13	2, 3, 13	2, 3, 13
15	1.96 – 1.90	21.5	6, 16	16	6, 8, 16
16	0.95	13.1	15	15	15
17	1.63 – 1.61	13.7	9, 10, 12	9, 12	10, 12
18	1.26	19.7	12	12	12

8.3.10 (3*R*,6*E*,10*E*,12*S*)-3,7,11-Trimethyl-6,10-tridecadien-12-olide (**6**)

The saponification was carried out analogously to procedure 8.3.9 with lithium hydroxide (35.0 mg, 1.46 mmol, 5.0 eq.) and methyl (3*R*,12*S*)-12-hydroxy-3,7,11-trimethyltrideca-6,10-dienoate (**s8**, 82.3 mg, 0.291 mmol, 1.0 eq.) in THF/H_2O 1:1 (1.6 ml). After stirring for 21 h at 40 °C, (3*R*,12*S*)-12-hydroxy-3,7,11-trimethyltrideca-6,10-dienoic acid (**21**) was obtained as a light-yellow liquid (78.0 mg, 0.291 mmol, 100 %) and was used in the next synthesis step without further purification.

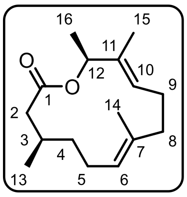

The Yamaguchi macrolactonization was carried out analogously to procedure 8.3.9 with triethylamine (56.5 mg, 0.558 mmol, 2.0 eq.) in THF (0.2 ml), TCBC (136 mg, 0.558 mmol, 2.0 eq.) in THF (0.2 ml), (3*R*,12*S*)-12-hydroxy-3,7,11-trimethyltrideca-6,10-dienoic acid (**21**, 75.0 mg, 0.279 mmol, 1.0 eq.) in THF (5.5 ml), toluene (54 ml) and DMAP (137 mg, 1.12 mmol, 4.0 eq.) in toluene (140 ml). The isomer mixture was obtained as a colourless liquid (56.3 mg, 0.225 mmol, 81 %). (3*R*,6*E*,10*E*,12*S*)-3,7,11-Trimethyl-6,10-tridecadien-12-olide (**6**, 11.3 mg, 45.1 µmol, 16 %) was obtained by column chromatography with silver nitrate on silica gel.

R$_f$ = 0.30 (Pe/Et_2O 20:1); $[\boldsymbol{\alpha}]_{\mathbf{D}}^{25}$ = +40.8 ± 0.4 (c = 1.09, CH_2Cl_2).; **^{1}H NMR** (500 MHz, $CDCl_3$): *δ* [ppm] = 5.41 – 5.36 (m, 1H, 10-H), 5.33 (q, *J* = 6.7 Hz, 1H, 12-H), 5.12 – 5.07 (m, 1H, 6-H), 2.23 – 2.14 (m, 1H, 9-H$_a$), 2.16 – 2.11 (m, 1H, 2-H$_a$), 2.16 – 2.10 (m, 1H, 8-H$_a$), 2.14 – 2.06 (m, 1H, 5-H$_a$), 2.08 – 2.02 (m, 1H, 2-H$_b$), 2.08 – 1.99 (m, 1H, 9-H$_b$), 2.03 – 1.95 (m, 1H, 8-H$_b$), 1.93 – 1.85 (m, 1H, 3-H), 1.83 – 1.75 (m, 1H, 5-H$_b$), 1.55 (s, 6H, 14,15-H), 1.47 – 1.39 (m, 1H, 4-H$_a$), 1.33 – 1.26 (m, 1H, 4-H$_b$), 1.27 (d, *J* = 6.7 Hz, 3H, 16-H), 0.99 (d, *J* = 6.8 Hz, 3H, 13-H); **^{13}C NMR** (125 MHz, $CDCl_3$): *δ* [ppm] = 173.3 (C$_q$, 1-C), 135.1 (C$_q$, 11-C), 132.4 (C$_q$, 7-C), 128.3

(CH, 10-C), 126.8 (CH, 6-C), 75.1 (CH, 12-C), 43.0 (CH_2, 2-C), 38.9 (CH_2, 8-C), 36.8 (CH_2, 4-C), 30.7 (CH, 3-C), 24.9 (CH_2, 5-C), 24.5 (CH_2, 9-C), 22.2 (CH_3, 13-C), 18.5 (CH_3, 16-C), 15.3 (CH_3, 14-C), 12.1 (CH_3, 15-C); **IR** (GC/FTIR): $\tilde{\nu}$ [cm^{-1}] = 2955 (m), 2928 (m), 2858 (m), 1727 (s), 1452 (m), 1442 (m), 1378 (w), 1357 (w), 1330 (w), 1286 (m), 1241 (m), 1184 (m), 1145 (m), 1072 (m), 1043 (w), 1023 (w), 972 (w), 955 (w), 942 (w), 931 (w), 832 (w); **EI-MS** (70 eV): *m*/*z* (%) = 250 (12) $[M]^+$, 235 (2), 221 (4), 169 (14), 168 (100), 109 (96), 108 (56), 107 (13), 95 (11), 93 (33), 82 (88), 81 (31), 79 (19), 69 (32), 68 (16), 67 (86), 55 (22), 53 (17), 43 (17), 41 (33), 39 (13); **HRMS** (EI) *m*/*z*: Calculated for $C_{16}H_{26}O_2$ $[M]^+$ 250.19273, found: 250.19279; ***I*** (HP-5-MS): 1737.

Table 3: NMR data (500 MHz, $CDCl_3$) of (3*R*,12*S*)-**6**.

atom	^{1}H, δ [ppm]	^{13}C, δ [ppm]	COSY	NOESY	HMBC
1	-	173.3	-	-	2, 12
2	2.16 – 2.11, 2.08 – 2.02	43.0	3, 13	3, 4, 13	3, 4, 13
3	1.93 – 1.85	30.7	2, 4, 13	2, 4, 6, 13	2, 13
4	1.47 – 1.39, 1.33 – 1.26	36.8	3, 5	2, 3, 5, 6, 13	2, 5, 6, 13
5	2.14 – 2.06, 1.83 – 1.75	24.9	4, 6, 14	4, 6, 13	4, 6
6	5.12 – 5.07	126.8	5, 14	3, 4, 5, 8	4, 5, 8, 14
7	-	132.4	-	-	5, 8, 9, 14
8	2.16 – 2.10, 2.03 – 1.95	38.9	9	6, 9, 10	6, 9, 10, 14
9	2.23 – 2.14, 2.08 – 1.99	24.5	8, 10, 14, 15	8, 10, 14, 15	8, 10
10	5.41 – 5.36	128.3	9, 12, 15	8, 9, 12	8, 9, 12, 15
11	-	135.1	-	-	9, 12, 15, 16
12	5.33	75.1	10, 16	10, 15, 16	10, 15, 16
13	0.99	22.2	2, 3	2, 3, 4, 5	2, 3, 4
14	1.55	15.3	5, 6, 9	9	6, 8
15	1.55	12.1	9, 10	9, 12, 16	10, 12
16	1.27	18.5	12	12, 15	12

8.3.11 (3*R*,6*E*,10*E*,12*S*)-7-Ethyl-3,11-dimethyl-6,10-tridecadien-12-olide (**7**)

The saponification was carried out analogously to procedure 8.3.9 with lithium hydroxide (12.5 mg, 0.520 mmol, 5.0 eq.) and methyl (3*R*,12*S*)-7-ethyl-12-hydroxy-3,11-dimethyltrideca-

6,10-dienoate (**s9**, 30.8 mg, 0.104 mmol, 1.0 eq.) in THF/H_2O 1:1 (1 ml). After stirring for 24 h at 40 °C, (3*R*,12*S*)-7-ethyl-12-hydroxy-3,11-dimethyltrideca-6,10-dienoic acid (**22**) was obtained as a light-yellow liquid (29.5 mg, 0.104 mmol, 100 %) and was used in the next synthesis step without further purification.

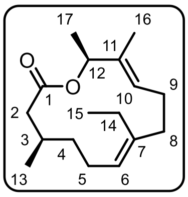

The Yamaguchi macrolactonization was carried out analogously to procedure 8.3.9 with triethylamine (18.6 mg, 0.184 mmol, 2.0 eq.) in THF (0.2 ml), TCBC (44.9 mg, 0.184 mmol, 2.0 eq.) in THF (0.2 ml), (3*R*,12*S*)-7-ethyl-12-hydroxy-3,11-dimethyltrideca-6,10-dienoic acid (**22**, 26.0 mg, 92.1 μmol, 1.0 eq.) in THF (2 ml), toluene (18 ml) and DMAP (45.0 mg, 0.368 mmol, 4.0 eq.) in toluene (50 ml). The isomer mixture was obtained as a colourless liquid (18.9 mg, 71.5 μmol, 78 %). (3*R*,6*E*,10*E*,12*S*)-7-Ethyl-3,11-dimethyl-6,10-tridecadien-12-olide (**7**, 2.01 mg, 7.60 μmol, 8 %) was obtained by column chromatography with silver nitrate on silica gel.

R_f = 0.30 (Pe/Et_2O 20:1); $[\boldsymbol{\alpha}]_{\mathbf{D}}^{25}$ = +46.8 ± 2.0 (c = 0.201, CH_2Cl_2); **^{1}H NMR** (600 MHz, $CDCl_3$): *δ* [ppm] = 5.42 – 5.38 (m, 1H, 10-H), 5.35 (q, *J* = 6.7 Hz, 1H, 12-H), 5.02 (dd, *J* = 9.5, 5.8 Hz, 1H, 6-H), 2.23 – 2.17 (m, 1H, 8-H_a), 2.17 – 2.11 (m, 1H, 9-H_a), 2.16 – 2.12 (m, 1H, 2-H_a), 2.15 – 2.08 (m, 1H, 5-H_a), 2.11 – 2.03 (m, 1H, 14-H_a), 2.07 – 2.01 (m, 1H, 2-H_b), 2.05 – 1.99 (m, 1H, 9-H_b), 1.95 – 1.88 (m, 1H, 8-H_b), 1.92 – 1.84 (m, 1H, 3-H), 1.91 – 1.83 (m, 1H, 14-H_b), 1.81 – 1.74 (m, 1H, 5-H_b), 1.53 – 1.52 (m, 3H, 16-H), 1.44 – 1.37 (m, 1H, 4-H_a), 1.33 – 1.27 (m, 1H, 4-H_b), 1.27 (d, *J* = 6.7 Hz, 3H, 17-H), 0.99 (d, *J* = 6.8 Hz, 3H, 13-H), 0.94 (t, *J* = 7.6 Hz, 3H, 15-H); **^{13}C NMR** (150 MHz, $CDCl_3$): *δ* [ppm] = 173.3 (C_q, 1-C), 138.1 (C_q, 7-C), 135.0 (C_q, 11-C), 128.7 (CH, 10-C), 126.4 (CH, 6-C), 75.3 (CH, 12-C), 43.1 (CH_2, 2-C), 36.7 (CH_2, 4-C), 35.4 (CH_2, 8-C), 30.7 (CH, 3-C), 24.9 (CH_2, 9-C), 24.5 (CH_2, 5-C), 22.2 (CH_3, 13-C), 22.0 (CH_2, 14-C), 18.5 (CH_3, 17-C), 13.4 (CH_3, 15-C), 12.0 (CH_3, 16-C); **IR** (GC/FTIR): $\tilde{\nu}$ [cm^{-1}] = 2961 (s), 2931 (m), 2868 (m), 1728 (s), 1457 (m), 1439 (m), 1378 (w), 1356 (w), 1331 (w), 1286 (m), 1261 (m), 1240 (m), 1182 (m), 1145 (m), 1132 (m), 1064 (s), 1045 (s), 958 (w), 932 (w), 859 (w), 803 (s), 710 (w); **EI-MS** (70 eV): *m*/*z* (%) = 264 (6) $[M]^+$, 235 (7), 208 (14), 182 (64), 153 (19), 123 (84), 122 (67), 107 (28), 95 (35), 93 (34), 83 (26), 82 (99), 81 (32), 79 (24), 69 (23), 67 (100), 55 (33), 53 (17), 43 (20), 41 (41); **HRMS** (EI) *m*/*z*: Calculated for $C_{17}H_{28}O_2$ $[M]^+$ 264.20838, found: 264.20779; ***I*** (HP-5-MS): 1811.

Table 4: NMR data (600 MHz, $CDCl_3$) of (3*R*,12*S*)-**7**.

atom	^{1}H, δ [ppm]	^{13}C, δ [ppm]	COSY	NOESY	HMBC
1	-	173.3	-	-	2, 12
2	2.16 – 2.12, 2.07 – 2.01	43.1	3	4, 13	3, 4, 13
3	1.92 – 1.84	30.7	2, 13	4, 13	2, 4, 5, 13

atom	^{1}H, δ [ppm]	^{13}C, δ [ppm]	COSY	NOESY	HMBC
4	1.44 – 1.37, 1.33 – 1.27	36.7	5, 13	2, 3, 5, 6, 13	2, 3, 5, 6, 13
5	2.15 – 2.08, 1.81 – 1.74	24.5	4, 6	4, 6, 13	3, 4, 6
6	5.02	126.4	5	4, 5, 8	4, 5, 8, 14
7	-	138.1	-	-	5, 8, 14, 15
8	2.23 – 2.17, 1.95 – 1.88	35.4	9	6, 9, 10, 15	6, 9, 10, 14
9	2.17 – 2.11, 2.05 – 1.99	24.9	8, 10, 16	8, 10, 16	8, 10, 16
10	5.42 – 5.38	128.7	9, 16	8, 9, 12	8, 9, 12, 16
11	-	135.0	-	-	9, 12, 16, 17
12	5.35	75.3	17	10, 16, 17	10, 16, 17
13	0.99	22.2	3, 4	2, 3, 4, 5	2, 3, 4
14	2.11 – 2.03, 1.91 – 1.83	22.0	15	15	6, 8, 15
15	0.94	13.4	14	8, 14	14
16	1.53 – 1.52	12.0	9, 10	9, 12	9, 10, 12
17	1.27	18.5	12	12	12

8.3.12 (3*R*,12*S*)-3-Ethyl-7,11-dimethyl-6,10-tridecadien-12-olide (**8**)

The saponification was carried out analogously to procedure 8.3.9 with lithium hydroxide (4.8 mg, 0.202 mmol, 5.0 eq.) and methyl (3*R*,12*S*)-3-ethyl-12-hydroxy-7,11-dimethyltrideca-6,10-dienoate (**s10**, 12.0 mg, 40.5 µmol, 1.0 eq.) in THF/H_2O 1:1 (0.6 ml). After stirring for 24 h at 40 °C, (3*R*,12*S*)-3-ethyl-12-hydroxy-7,11-dimethyltrideca-6,10-dienoic acid (**23**) was obtained as a light-yellow liquid (3.0 mg, 10.6 µmol, 26 %) and was used in the next synthesis step without further purification.

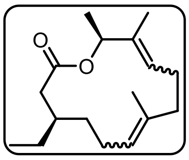

The Yamaguchi macrolactonization was carried out analogously to procedure 8.3.9 with triethylamine (2.1 mg, 21.2 µmol, 2.0 eq.) in THF (0.2 ml), TCBC (5.2 mg, 21.2 µmol, 2.0 eq.) in THF (0.2 ml), (3*R*,12*S*)-3-ethyl-12-hydroxy-7,11-dimethyltrideca-6,10-dienoic acid (**23**, 3.0 mg, 10.6 µmol, 1.0 eq.) in THF (1 ml), toluene (2 ml) and DMAP (5.2 mg, 42.4 µmol, 4.0 eq.) in toluene (6 ml). (3*R*,12*S*)-3-Ethyl-7,11-dimethyl-6,10-tridecadien-12-olide (**8**) was obtained as a colourless liquid (1.6 mg, 6.05 µmol, 57 %).

R$_f$ = 0.30 (Pe/Et_2O 20:1); **^{1}H NMR** (600 MHz, $CDCl_3$): δ [ppm] = isomeric mixture 5.55 – 5.04 (m, 12H, 2×=CH, CH), 2.47 – 1.84 (m, 36H, 4×CH_2, CH), 1.79 – 1.77 (m, 3H, CH_3), 1.78 – 1.76 (m, 3H, CH_3), 1.69 – 1.68 (m, 3H, CH_3), 1.69 – 1.67 (m, 3H, CH_3), 1.67 – 1.65 (m, 3H, CH_3), 1.58 – 1.57 (m, 3H, CH_3), 1.56 – 1.55 (m, 3H, CH_3), 1.55 – 1.54 (m, 3H, CH_3), 1.45 – 1.15 (m, 28H, 2×CH_2, CH_3), 0.99 – 0.75 (m, 12H, CH_3); **^{13}C NMR** (150 MHz, $CDCl_3$): δ [ppm] = isomeric mixture 173.6 (C_q), 173.5 (C_q), 173.3 (C_q), 173.2 (C_q), 135.6 (C_q), 135.4 (C_q), 135.0 (C_q), 134.8 (C_q), 134.3 (C_q), 133.8 (C_q), 133.4 (C_q), 132.1 (C_q), 131.7 (CH), 130.0 (CH), 128.7 (CH), 128.0 (CH), 126.7 (CH), 126.5 (CH), 126.3 (CH), 126.3 (CH), 75.3 (CH), 74.4 (CH), 69.9 (CH), 69.8 (CH), 40.4 (CH_2), 40.2 (CH_2), 39.3 (CH_2), 38.9 (CH_2), 38.1 (CH_2), 38.0 (CH), 38.0 (CH_2), 37.2 (CH), 35.0 (CH_2), 34.2 (CH_2), 33.1 (CH_2), 32.3 (CH_2), 32.3 (CH_2), 31.9 (CH_2), 31.4 (CH), 31.3 (CH_2), 30.2 (CH), 29.7 (CH_2), 29.4 (CH_2), 29.1 (CH_2), 28.8 (CH_2), 28.8 (CH_2), 27.8 (CH_2), 25.2 (CH_2), 24.8 (CH_2), 24.6 (CH_2), 24.6 (CH_2), 22.7 (CH_2), 21.6 (CH_3), 21.1 (CH_3), 20.9 (CH_3), 19.2 (CH_3), 18.6 (CH_3), 18.4 (CH_3), 18.3 (CH_3), 17.9 (CH_3), 15.3 (CH_3), 14.1 (2C, CH_3), 13.4 (CH_3), 11.9 (CH_3), 11.8 (CH_3), 11.6 (CH_3), 11.4 (CH_3); **IR** (GC/FTIR): $\tilde{\nu}$ [cm^{-1}] = 1. isomer 2962 (s), 2933 (s), 2875 (m), 2856 (m), 1735 (s), 1451 (m), 1414 (w), 1379 (m), 1342 (w), 1323 (w), 1304 (w), 1269 (w), 1237 (w), 1219 (w), 1185 (m), 1146 (m), 1094 (w), 1076 (m), 1053 (w), 1036 (w), 946 (w), 869 (w), 855 (w); 2. isomer 2962 (s), 2932 (s), 2876 (m), 2856 (m), 1736 (s), 1451 (m), 1414 (w), 1379 (m), 1341 (w), 1328 (w), 1283 (w), 1258 (w), 1233 (w), 1185 (m), 1146 (m), 1094 (m), 1078 (m), 1052 (m), 1037 (m), 1000 (w), 946 (w); 3. isomer 2962 (s), 2930 (s), 2871 (m), 2858 (m), 1728 (s), 1461 (m), 1449 (m), 1378 (m), 1341 (w), 1303 (w), 1264 (w), 1184 (w), 1163 (w), 1143 (m), 1102 (w), 1074 (m), 1051 (w), 1015 (w), 974 (w), 955 (w), 940 (w), 903 (w), 857 (w); 4. isomer 2962 (s), 2930 (s), 2871 (m), 2858 (m), 1728 (s), 1462 (m), 1377 (m), 1343 (w), 1302 (w), 1268 (w), 1248 (m), 1221 (w), 1180 (w), 1166 (m), 1143 (m), 1102 (w), 1081 (m), 1051 (m), 1014 (w), 974 (w), 956 (w), 915 (w), 856 (m), 836 (w); **EI-MS** (70 eV): *m/z* (%) = 1. isomer 264 (14) [M]$^+$, 235 (4), 182 (100), 153 (27), 126 (16), 123 (77), 122 (62), 121 (16), 107 (20), 93 (45), 83 (23), 82 (91), 81 (40), 79 (23), 69 (32), 68 (20), 67 (93), 55 (34), 53 (20), 43 (20), 41 (44); 2. isomer 264 (14) [M]$^+$, 235 (7), 182 (64), 153 (23), 135 (19), 123 (76), 122 (52), 121 (21), 107 (22), 93 (44), 83 (25), 82 (91), 81 (42), 79 (24), 69 (36), 68 (19), 67 (100), 55 (35), 53 (20), 43 (22), 41 (46); 3. isomer 264 (15) [M]$^+$, 235 (5), 182 (93), 153 (26), 135 (20), 123 (81), 122 (60), 121 (24), 107 (25), 93 (49), 83 (26), 82 (98), 81 (46), 79 (27), 69 (37), 68 (21), 67 (100), 55 (41), 53 (22), 43 (20), 41 (48); 4. isomer 264 (20) [M]$^+$, 235 (12), 182 (37), 153 (25), 135 (25), 123 (76), 122 (59), 121 (26), 107 (25), 93 (54), 83 (32), 82 (96), 81 (53), 79 (30), 69 (35), 67 (100), 55 (42), 53 (23), 43 (25), 41 (50); **HRMS** (EI) *m/z*: Calculated for $C_{17}H_{28}O_2$ [M]$^+$ 264.20838, found: 1. isomer 264.20831, 2. isomer 264.20865, 3. isomer 264.20840, 4. isomer 264.20844; ***I*** (HP-5-MS): 1. isomer 1813, 2. isomer 1842, 3. isomer 1851, 4. isomer 1854.

9 References

[1] a) D. A. Schooley, K. J. Judy, B. J. Bergot, M. S. Hall, J. B. Siddall, *Proc. Natl. Acad. Sci. U.S.A.* **1973**, *70*, 2921; b) P. A. Brindle, F. C. Baker, L. W. Tsai, C. C. Reuter, D. A. Schooley, *Proc. Natl. Acad. Sci. U.S.A.* **1987**, *84*, 7906.

[2] T.-S. Li, J.-T. Li, H.-Z. Li, *J. Chromatogr. A* **1995**, *715*, 372.

[3] A. Möllerke, J. Bello, H. P. Leinaas, S. Schulz, *J. Nat. Prod.* **2024**, *87*, 85.

[4] F. R. Pinacho Crisóstomo, R. Carrillo, L. G. León, T. Martín, J. M. Padrón, V. S. Martín, *J. Org. Chem.* **2006**, *71*, 2339.

[5] A. Tsimelzon, R. Braslau, *J. Org. Chem.* **2005**, *70*, 10854.

[6] B. ter Horst, B. L. Feringa, A. J. Minnaard, *Chem. Commun.* **2007**, 489.

[7] E. E. Kwan, J. R. Scheerer, D. A. Evans, *J. Org. Chem.* **2013**, *78*, 175.

[8] K. H. N. Tang, K. Uchida, K. Nishihara, M. Ito, T. Shibata, *Org. Lett.* **2022**, *24*, 1313.

[9] S. D. Laffoon, Z. Wu, K. L. Hull, *Chem. Commun.* **2018**, *54*, 7814.

[10] Y. Naoshima, H. Mukaidani, *J. Chem. Ecol.* **1987**, *13*, 325.

[11] X. Chen, Y. Zhang, H. Wan, W. Wang, S. Zhang, *Chem. Commun.* **2016**, *52*, 3532.

[12] C. Tran, B. Flamme, A. Chagnes, M. Haddad, P. Phansavath, V. Ratovelomanana-Vidal, *Synlett* **2018**, *29*, 1622.

[13] L. F. Toneto Novaes, C. Martins Avila, K. J. Pelizzaro-Rocha, D. B. Vendramini-Costa, M. Pereira Dias, D. B. Barbosa Trivella, J. Ernesto de Carvalho, C. V. Ferreira-Halder, R. A. Pilli, *ChemMedChem* **2015**, *10*, 1687.

[14] Y. Ichikawa, K. Okumura, Y. Matsuda, T. Hasegawa, M. Nakamura, A. Fujimoto, T. Masuda, K. Nakano, H. Kotsuki, *Org. Biomol. Chem.* **2012**, *10*, 614.

[15] E. Moulin, C. Nevado, J. Gagnepain, G. Kelter, H.-H. Fiebig, A. Fürstner, *Tetrahedron* **2010**, *66*, 6421.

[16] W. Xie, B. Zou, D. Pei, D. Ma, *Org. Lett.* **2005**, *7*, 2775.

[17] P. A. Wender, M. F. T. Koehler, M. Sendzik, *Org. Lett.* **2003**, *5*, 4549.

[18] L. E. Overman, G. M. Rishton, *Org. Synth.* **1993**, *71*, 56.

[19] S. R. Rizos, Z. V. Peitsinis, A. E. Koumbis, *J. Org. Chem.* **2021**, *86*, 10440.

[20] A. J. Bunt, C. D. Bailey, B. D. Cons, S. J. Edwards, J. D. Elsworth, T. Pheko, C. L. Willis, *Angew. Chem. Int. Ed.* **2012**, *51*, 3901.

[21] M. D. Pungente, L. Weiler, *Org. Lett.* **2001**, *3*, 643.

[22] S. G. Taher, J. R. Al Dulayymi, H. G. Tima, H. M. Ali, M. Romano, M. S. Baird, *Tetrahedron* **2016**, *72*, 3863.

[23] Y. Schmidt, K. Lehr, U. Breuninger, G. Brand, T. Reiss, B. Breit, *J. Org. Chem.* **2010**, *75*, 4424.

[24] S. T. Nguyen, E. A. McLoughlin, J. H. Cox, B. P. Fors, R. R. Knowles, *J. Am. Chem. Soc.* **2021**, *143*, 12268.

[25] A. Yurek-George, F. Habens, M. Brimmell, G. Packham, A. Ganesan, *J. Am. Chem. Soc.* **2004**, *126*, 1030.

10 NMR spectra

Methyl (*E*)-hepta-2,6-dienoate (**31**)

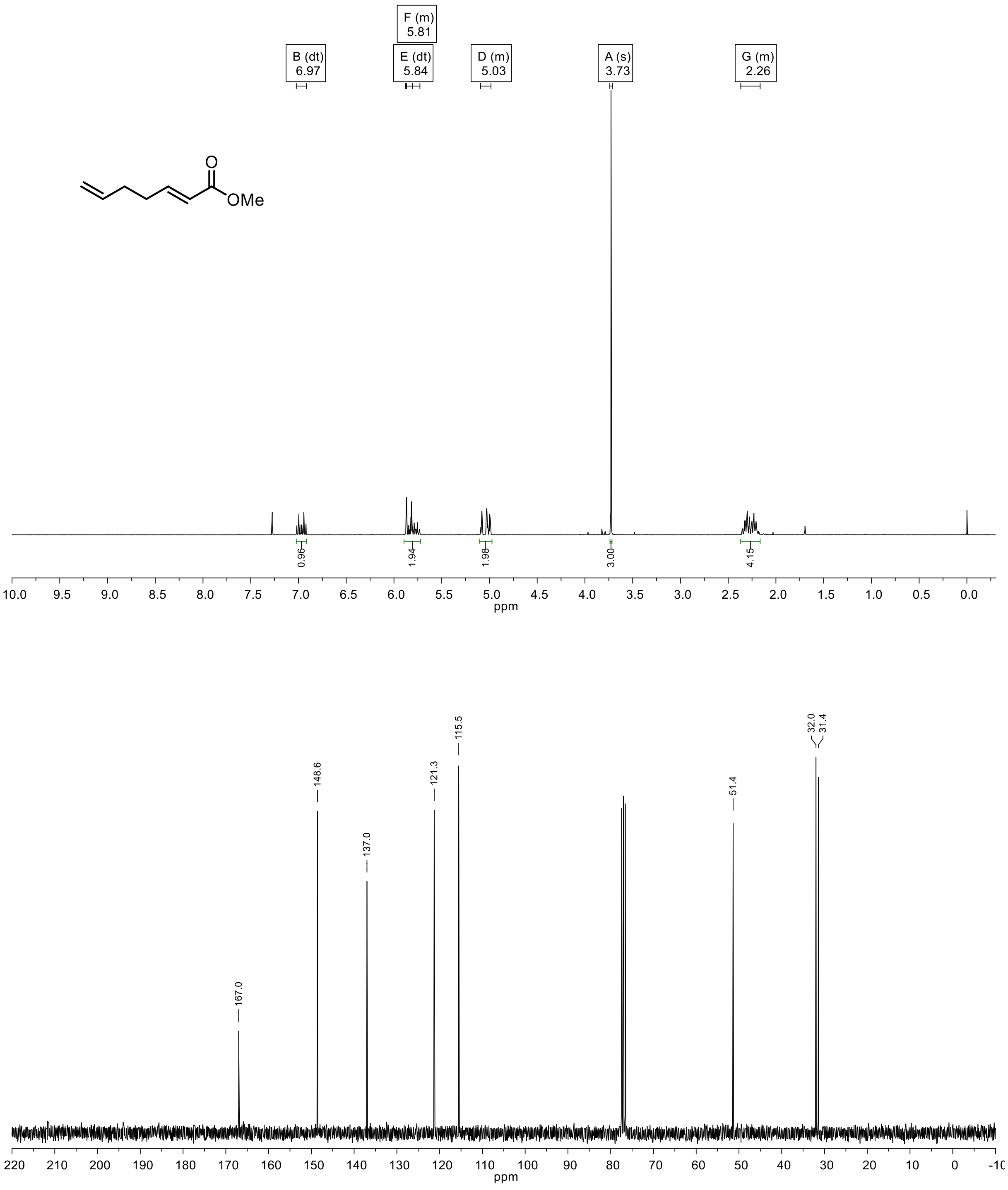

Fig. S8: Top: ^{1}H NMR (300 MHz, $CDCl_3$) and bottom: ^{13}C NMR spectrum (75 MHz, $CDCl_3$) of **31**.

Methyl (*R*)-3-ethylhept-6-enoate (**33**)

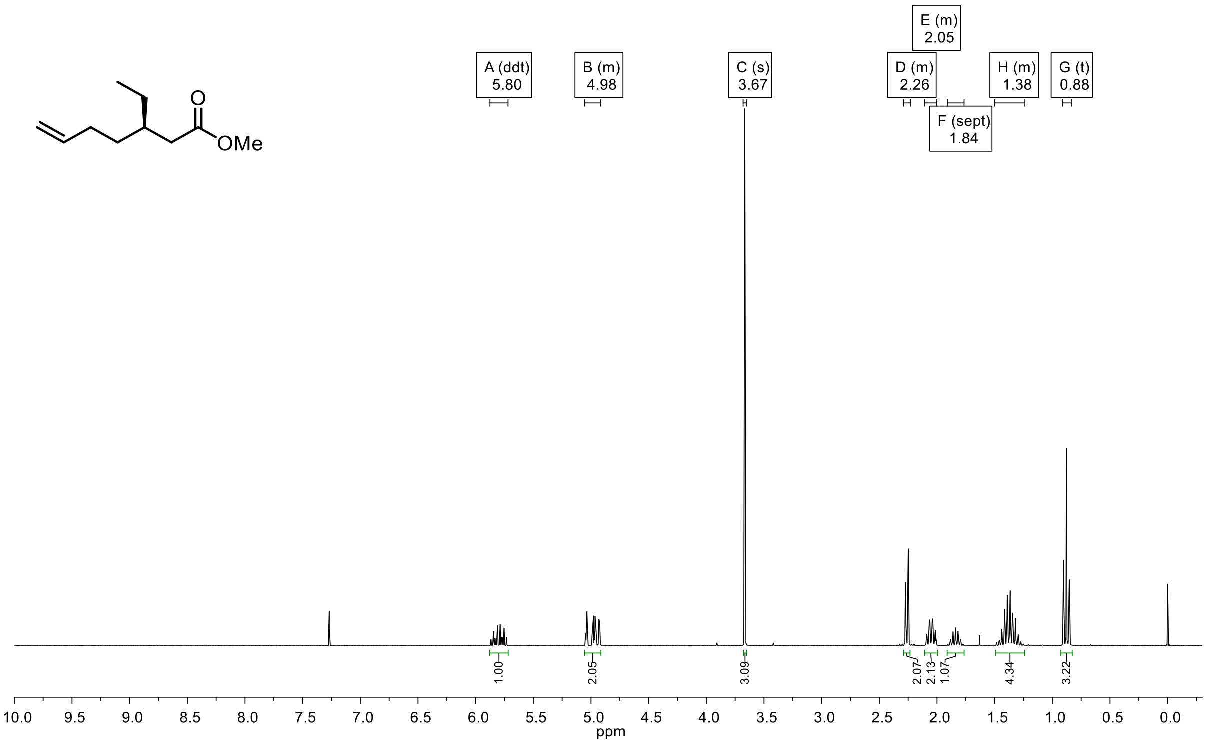

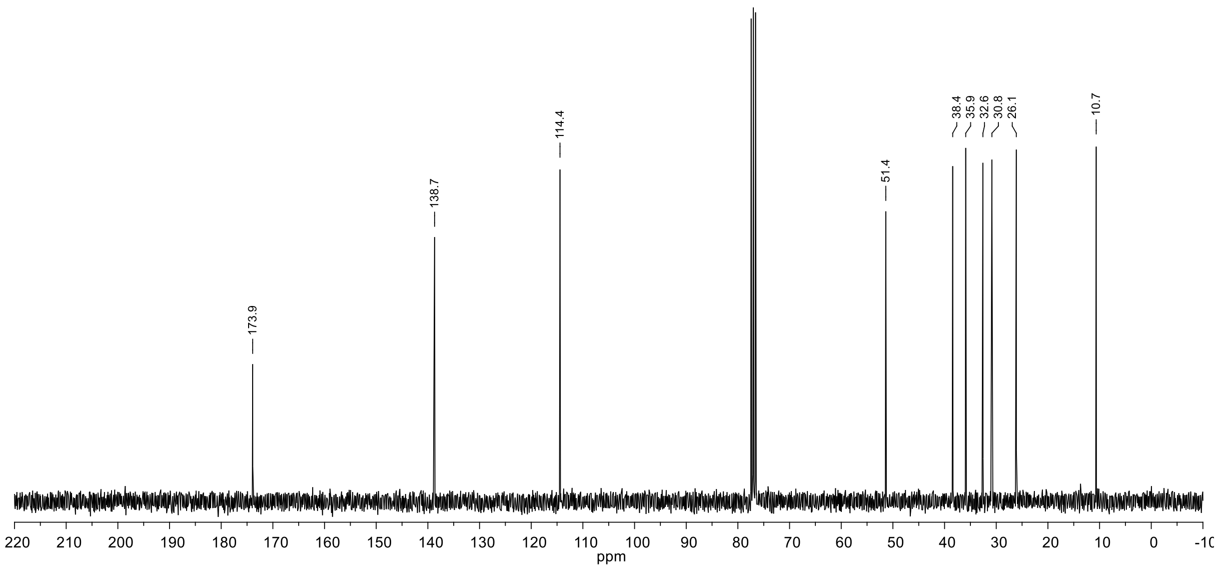

Fig. S9: Top: ^{1}H NMR (300 MHz, $CDCl_3$) and bottom: ^{13}C NMR spectrum (75 MHz, $CDCl_3$) of **33**.

Methyl (*R*)-3-ethyl-6-oxohexanoate (**26**)

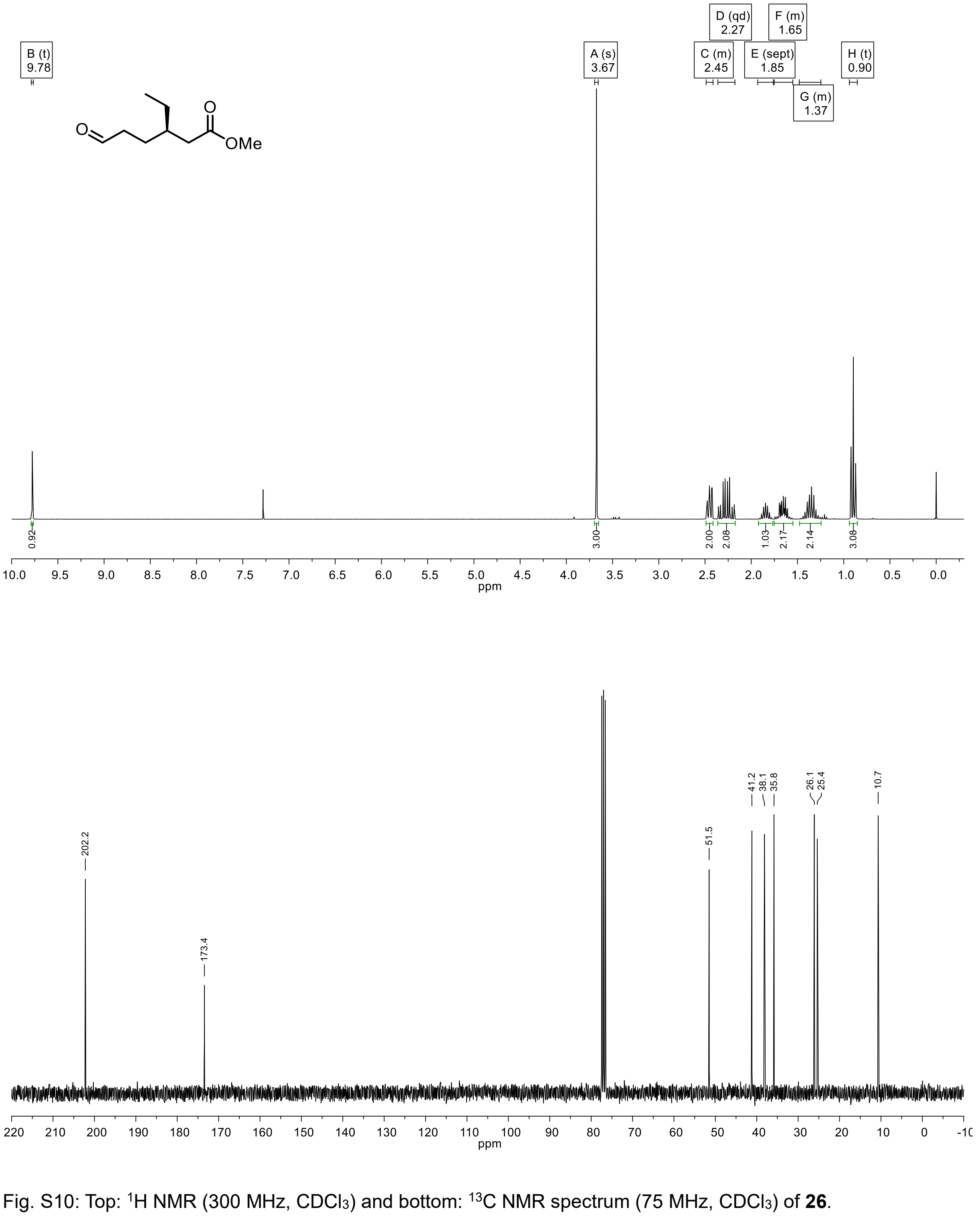

Fig. S10: Top: ^{1}H NMR (300 MHz, $CDCl_3$) and bottom: ^{13}C NMR spectrum (75 MHz, $CDCl_3$) of **26**.

Methyl (*R*)-3,7-dimethyloct-6-enoate (**s1**)

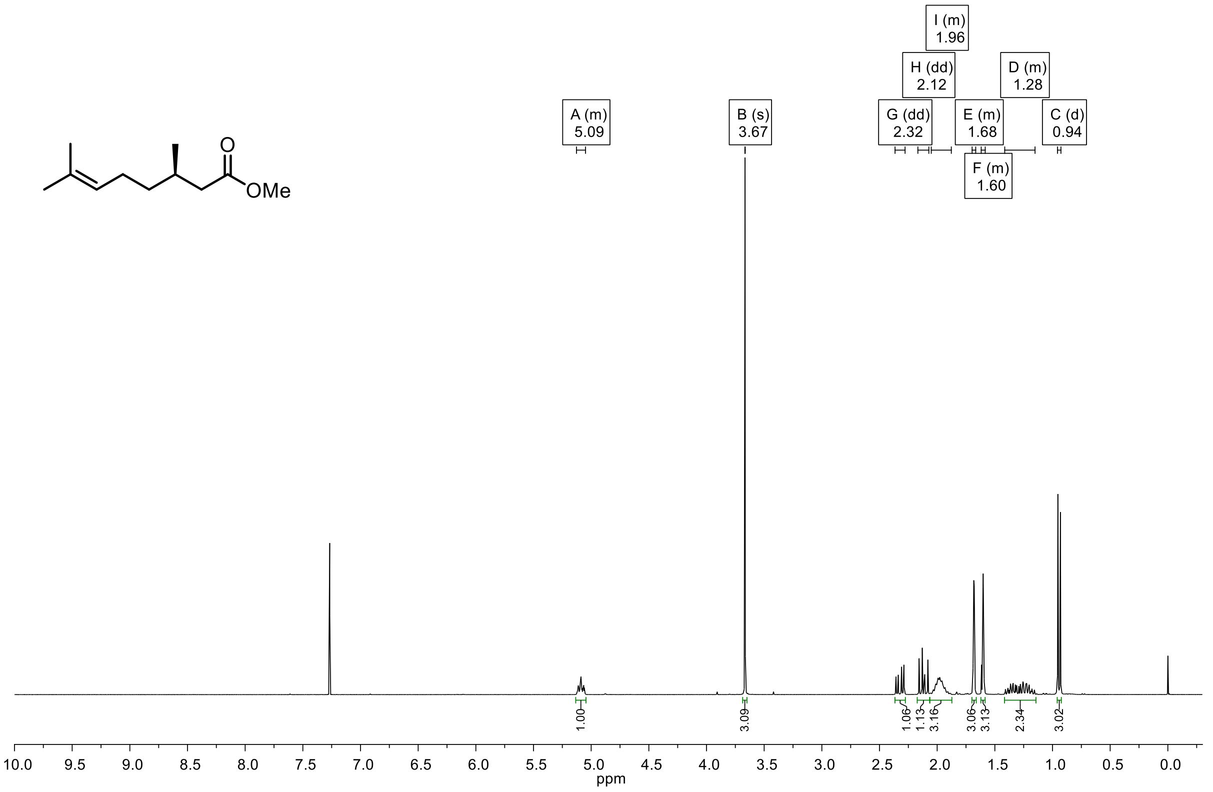

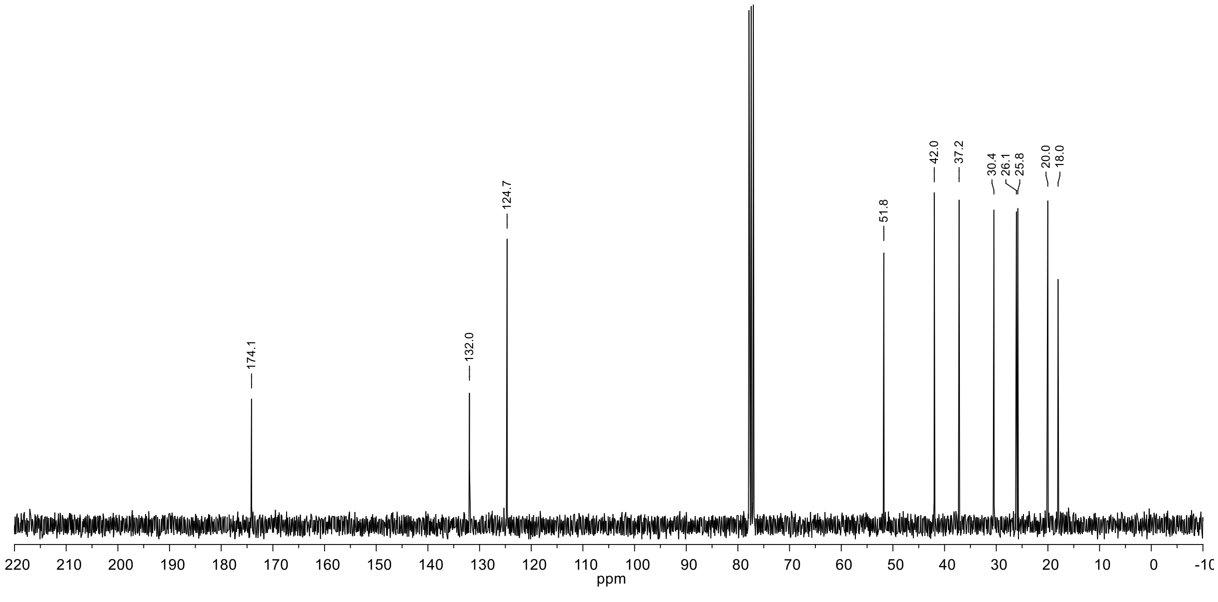

Fig. S11: Top: ^{1}H NMR (300 MHz, $CDCl_3$) and bottom: ^{13}C NMR spectrum (75 MHz, $CDCl_3$) of **s1**.

Methyl (*R*)-3-methyl-6-oxohexanoate (**25**)

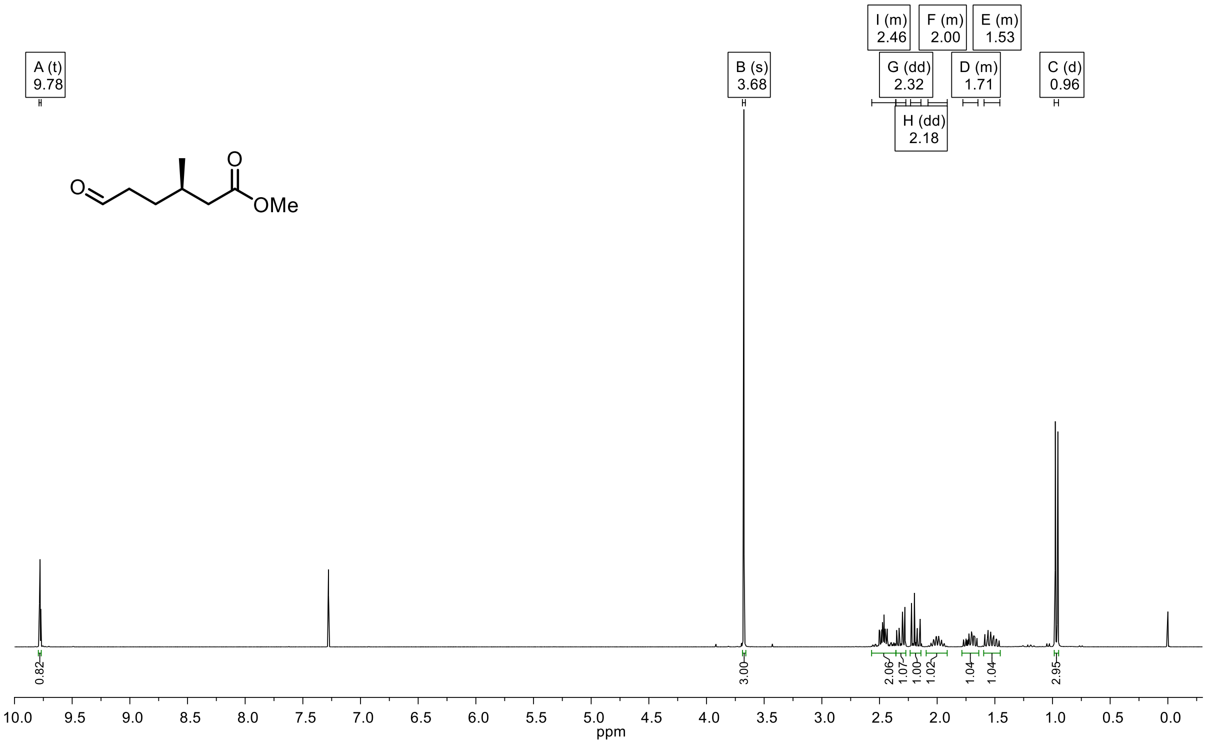

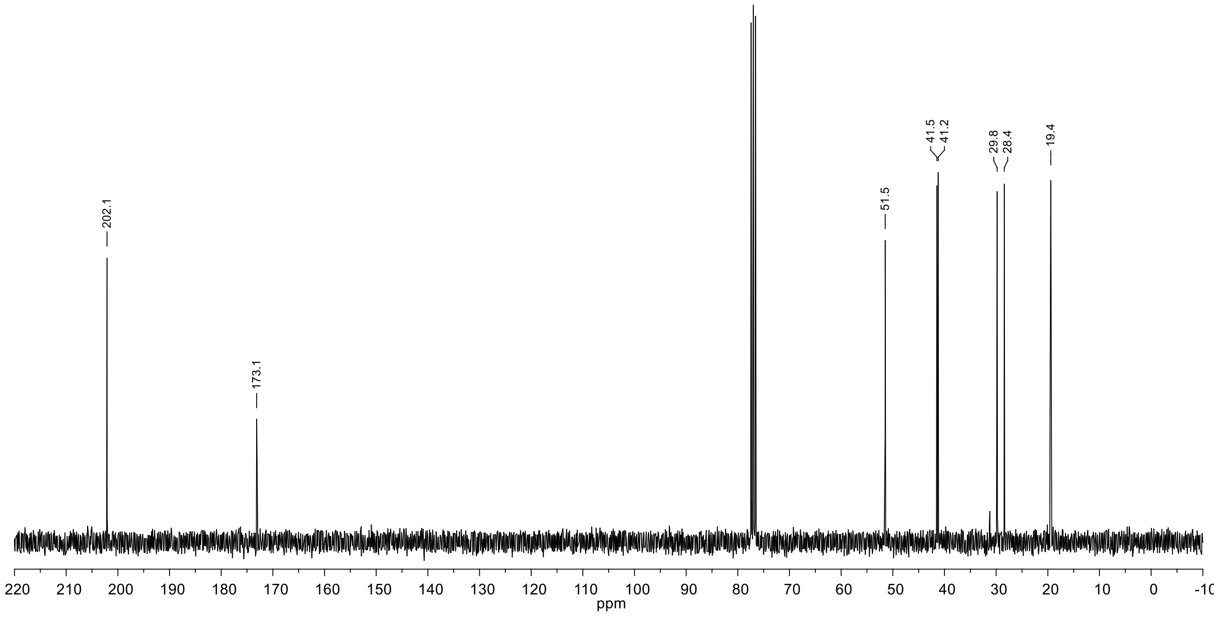

Fig. S12: Top: ^{1}H NMR (300 MHz, $CDCl_3$) and bottom: ^{13}C NMR spectrum (75 MHz, $CDCl_3$) of **25**.

Methyl 4-((1-phenyl-1*H*-tetrazol-5-yl)thio)butanoate (**s2**)

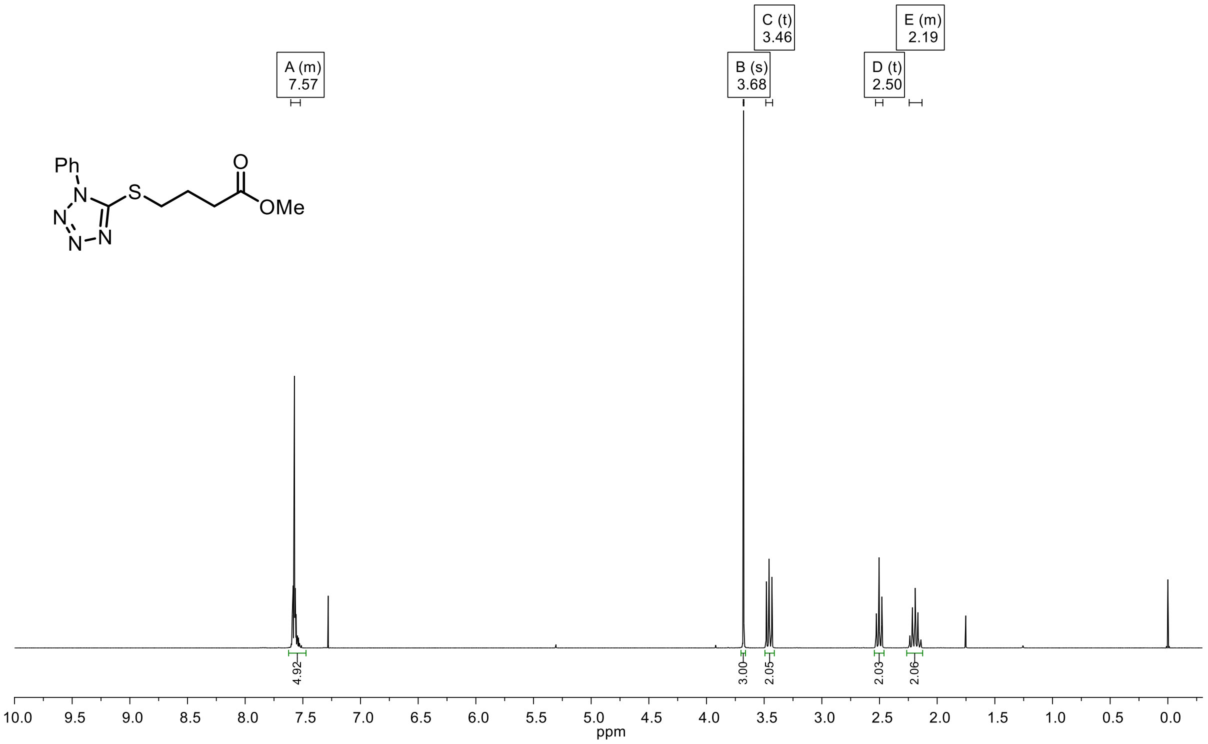

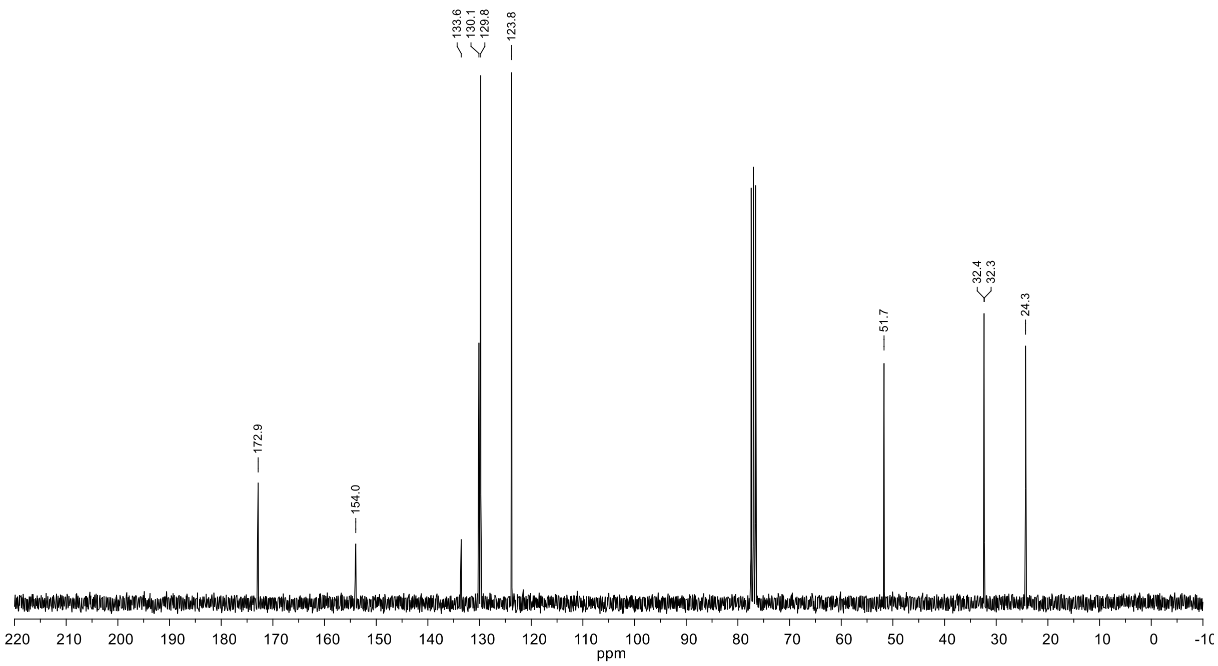

Fig. S13: Top: ^{1}H NMR (300 MHz, $CDCl_3$) and bottom: ^{13}C NMR spectrum (75 MHz, $CDCl_3$) of **s2**.

Methyl 4-((1-phenyl-1*H*-tetrazol-5-yl)sulfonyl)butanoate (**37**)

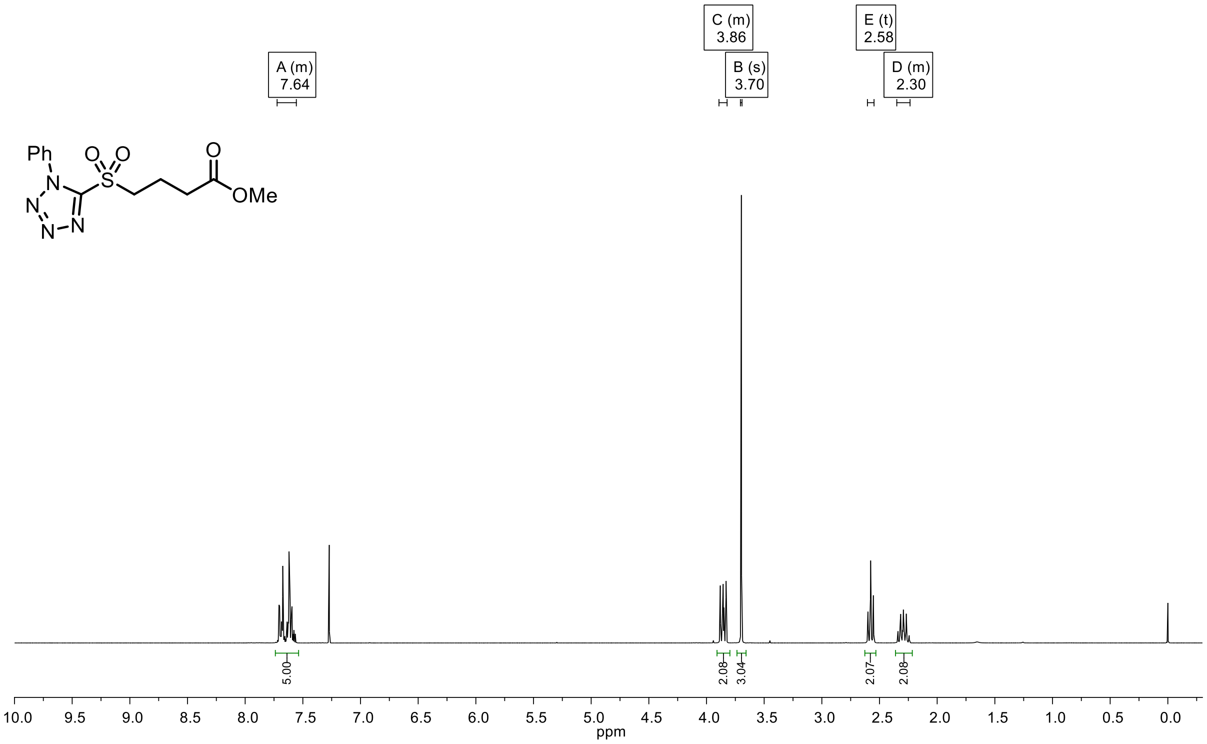

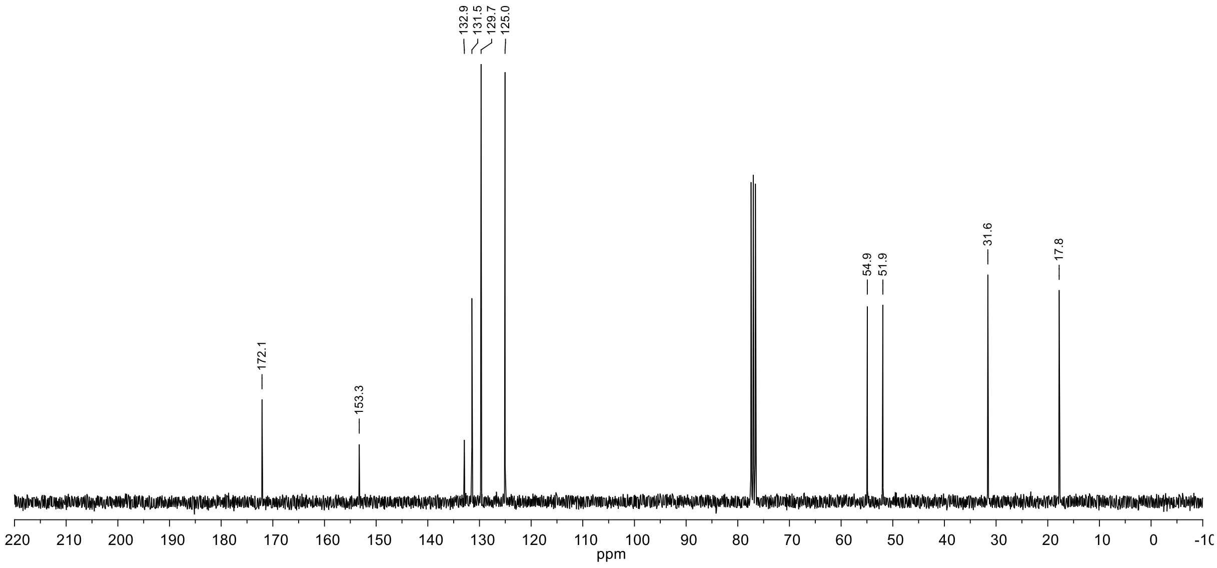

Fig. S14: Top: ^{1}H NMR (300 MHz, $CDCl_3$) and bottom: ^{13}C NMR spectrum (75 MHz, $CDCl_3$) of **37**.

Methyl (*S*)-2-((*tert*-butyldiphenylsilyl)oxy)propanoate (**s3**)

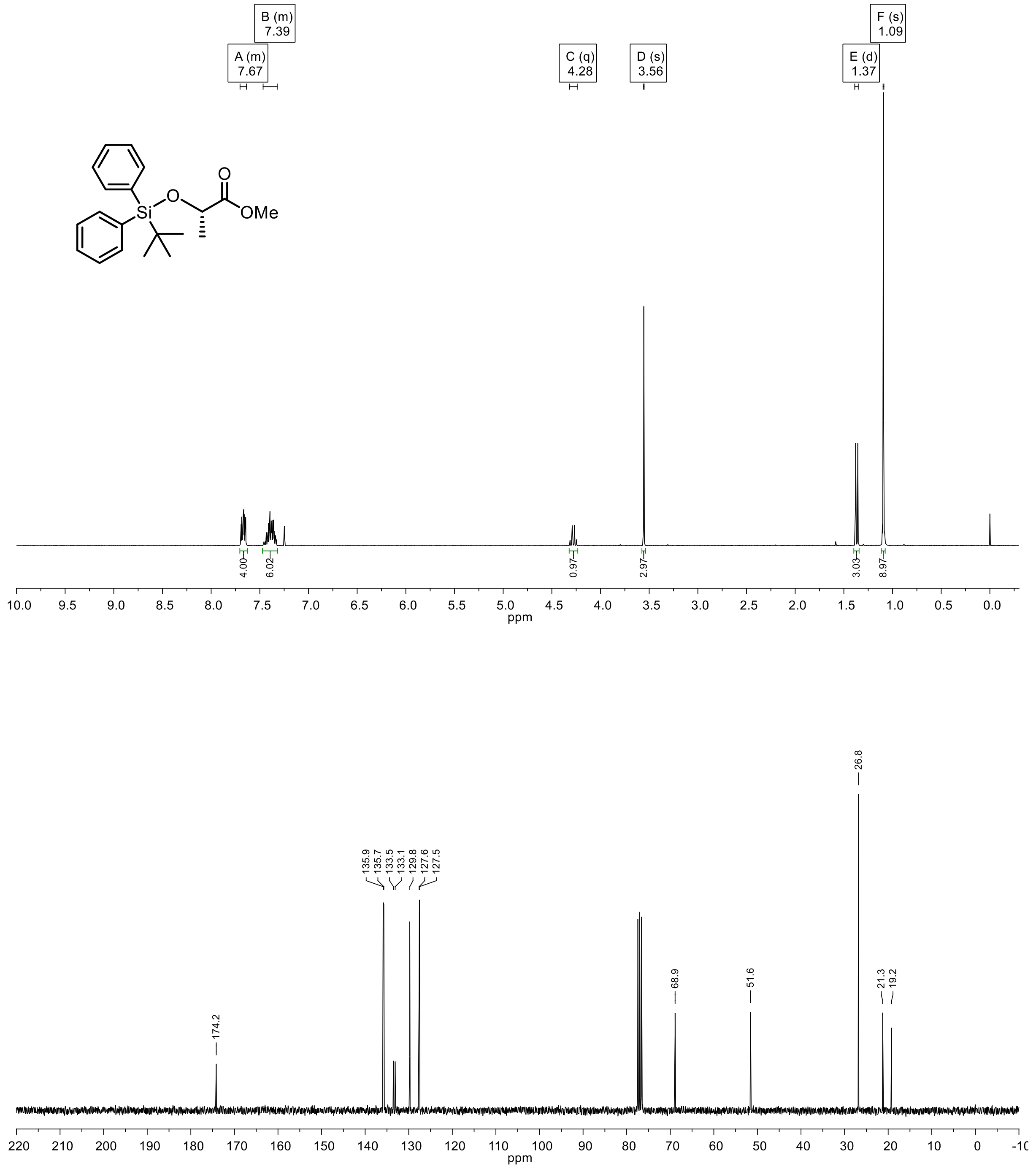

Fig. S15: Top: ^{1}H NMR (300 MHz, $CDCl_3$) and bottom: ^{13}C NMR spectrum (75 MHz, $CDCl_3$) of **s3**.

(*S*)-2-((*tert*-Butyldiphenylsilyl)oxy)-*N*-methoxy-*N*-methylpropanamide (**s4**)

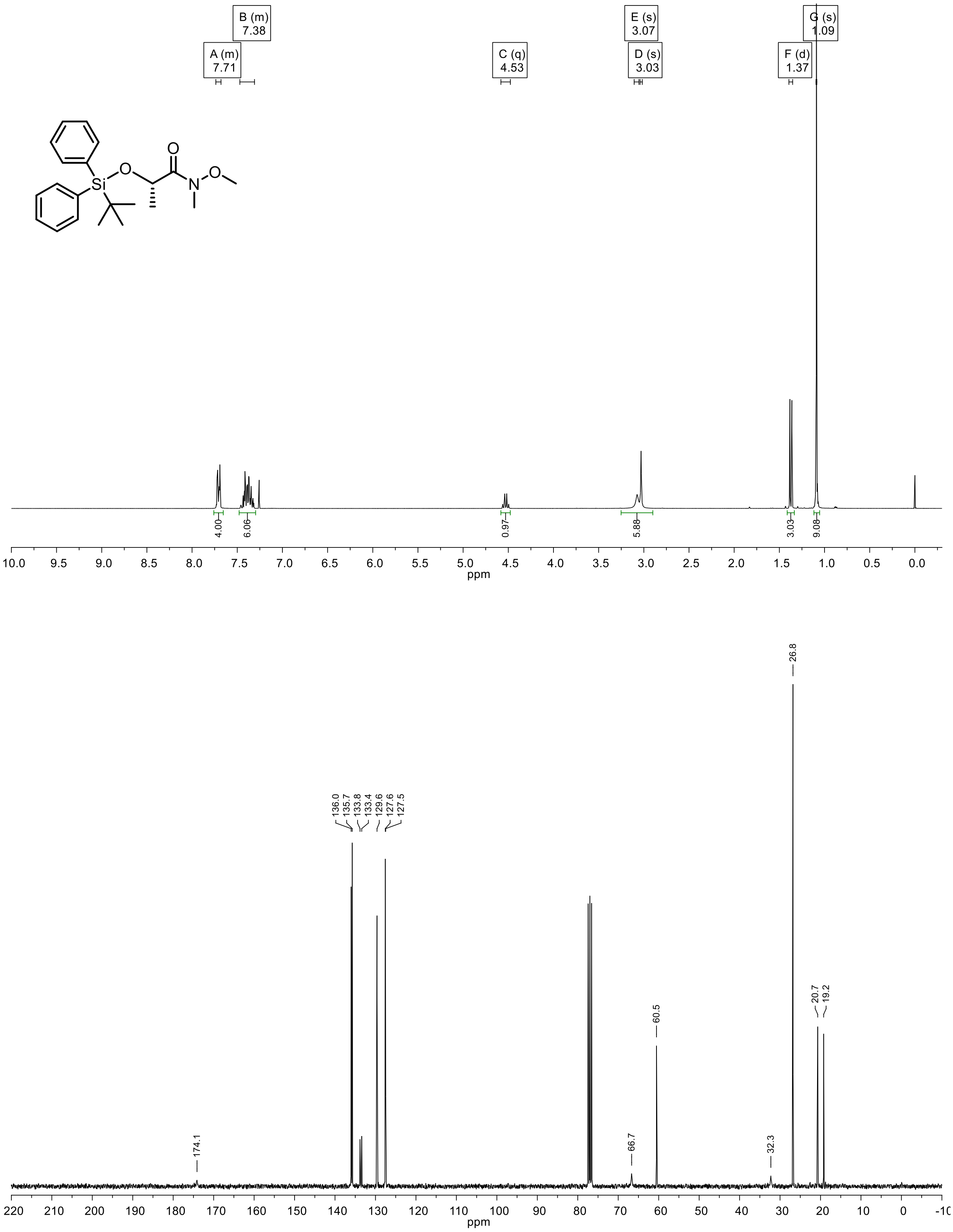

Fig. S16: Top: ^{1}H NMR (300 MHz, $CDCl_3$) and bottom: ^{13}C NMR spectrum (75 MHz, $CDCl_3$) of **s4**.

(*S*)-3-((*tert*-Butyldiphenylsilyl)oxy)butan-2-one (**35**)

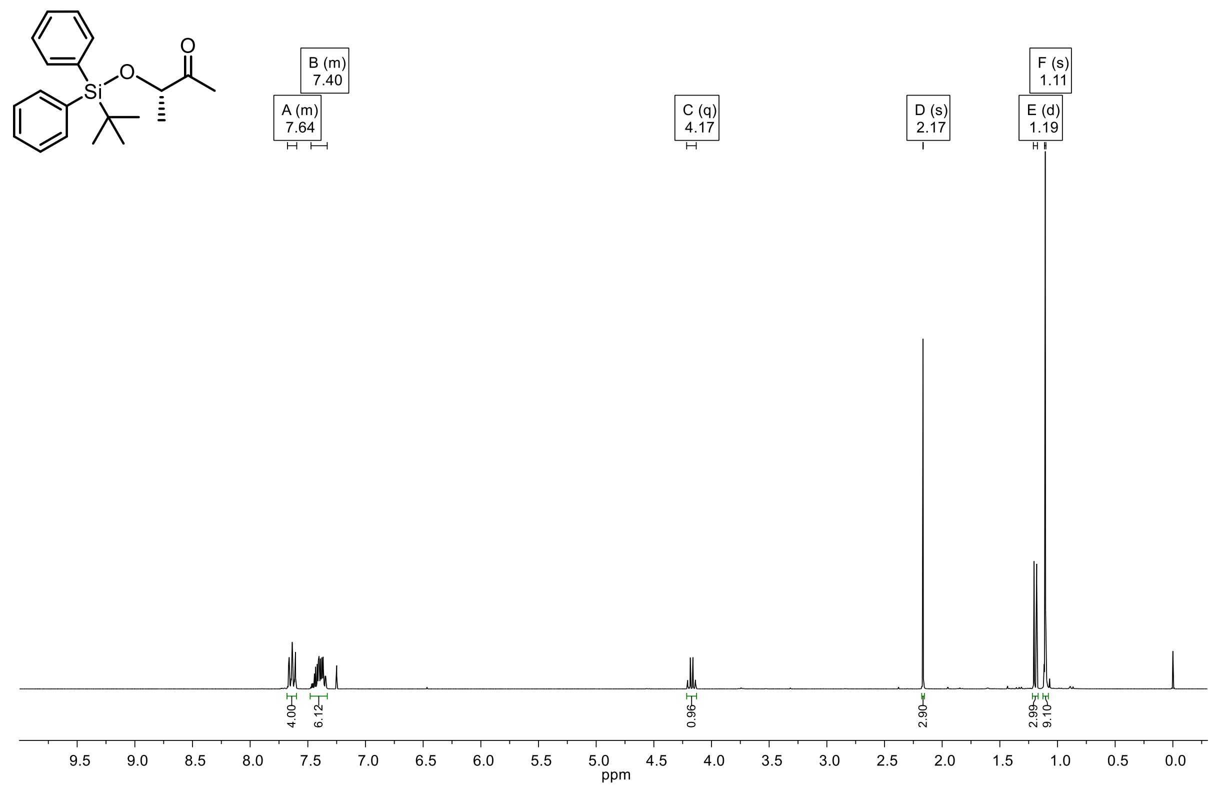

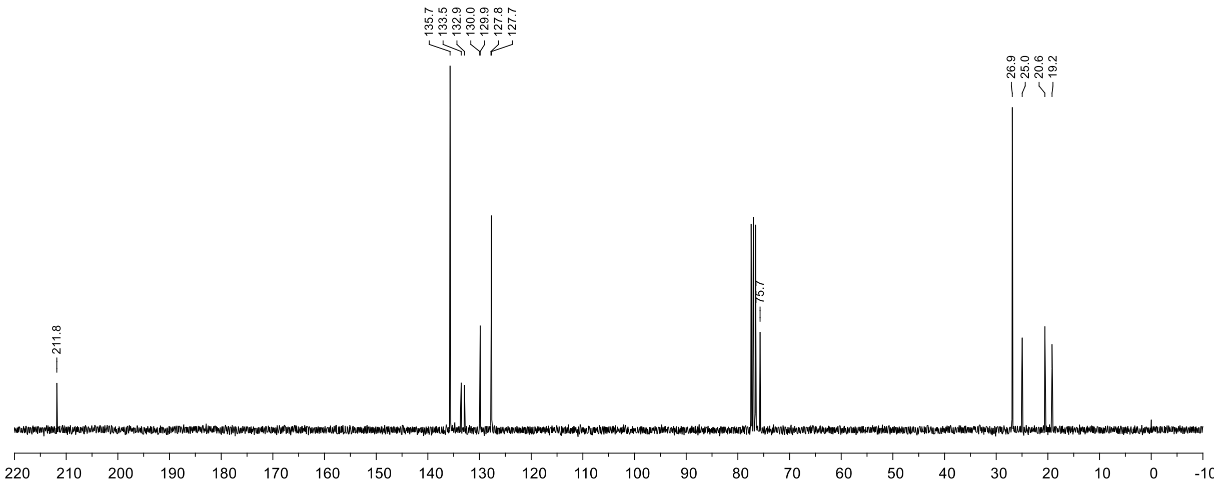

Fig. S17: Top: ^{1}H NMR (300 MHz, $CDCl_3$) and bottom: ^{13}C NMR spectrum (75 MHz, $CDCl_3$) of **35**.

Methyl (*S*)-6-((*tert*-butyldiphenylsilyl)oxy)-5-methylhept-4-enoate (**38**)

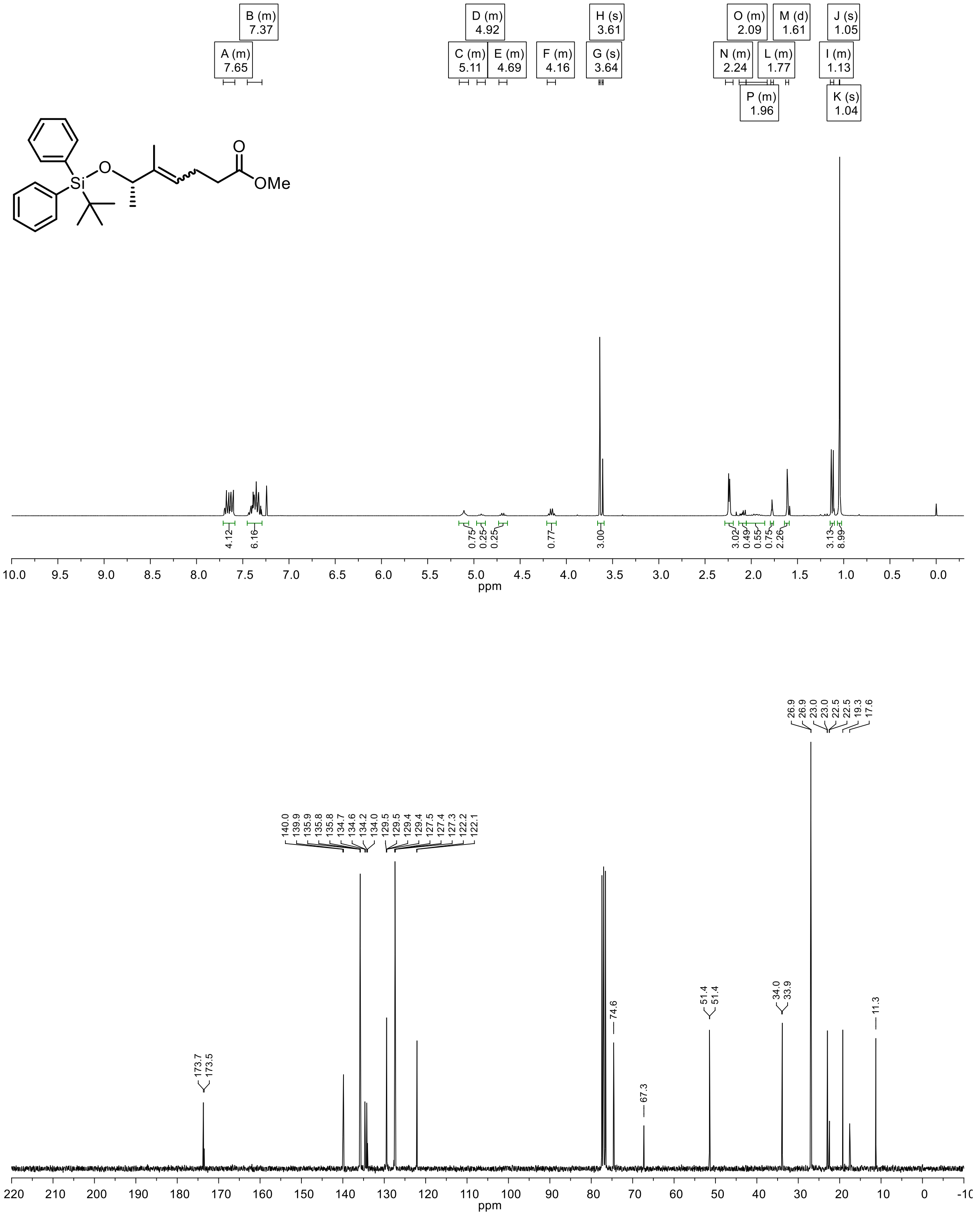

Fig. S18: Top: ^{1}H NMR (300 MHz, $CDCl_3$) and bottom: ^{13}C NMR spectrum (75 MHz, $CDCl_3$) of **38**.

(*S*)-6-((*tert*-Butyldiphenylsilyl)oxy)-5-methylhept-4-enal (**43**)

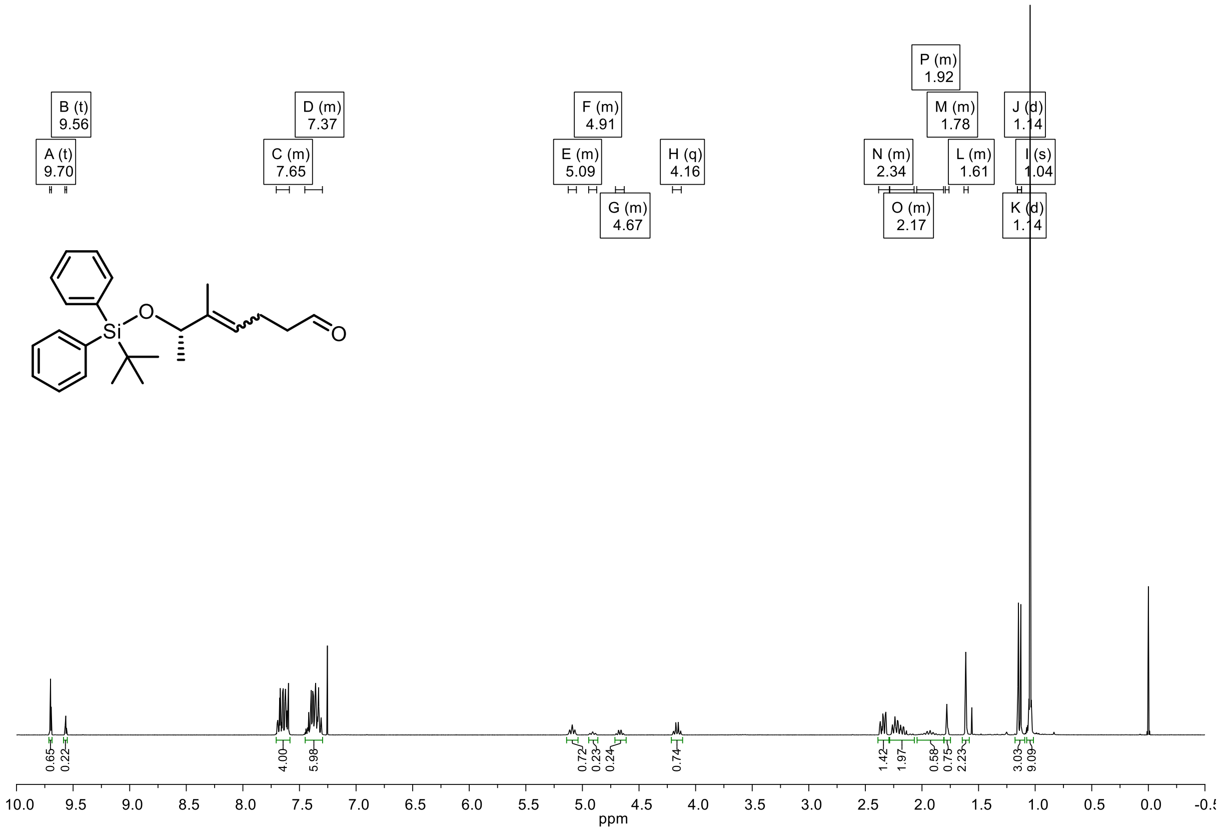

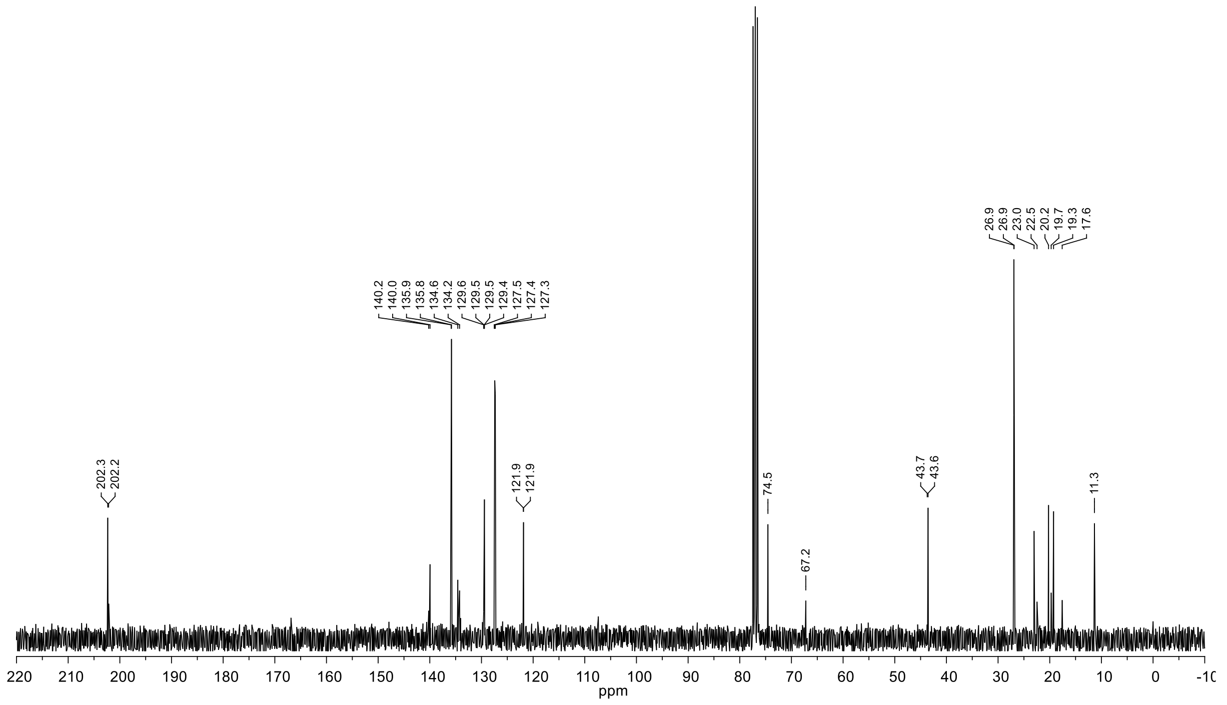

Fig. S19: Top: ^{1}H NMR (300 MHz, $CDCl_3$) and bottom: ^{13}C NMR spectrum (75 MHz, $CDCl_3$) of **43**.

(8*S*)-8-((*tert*-Butyldiphenylsilyl)oxy)-7-methylnon-6-en-3-ol (**39**)

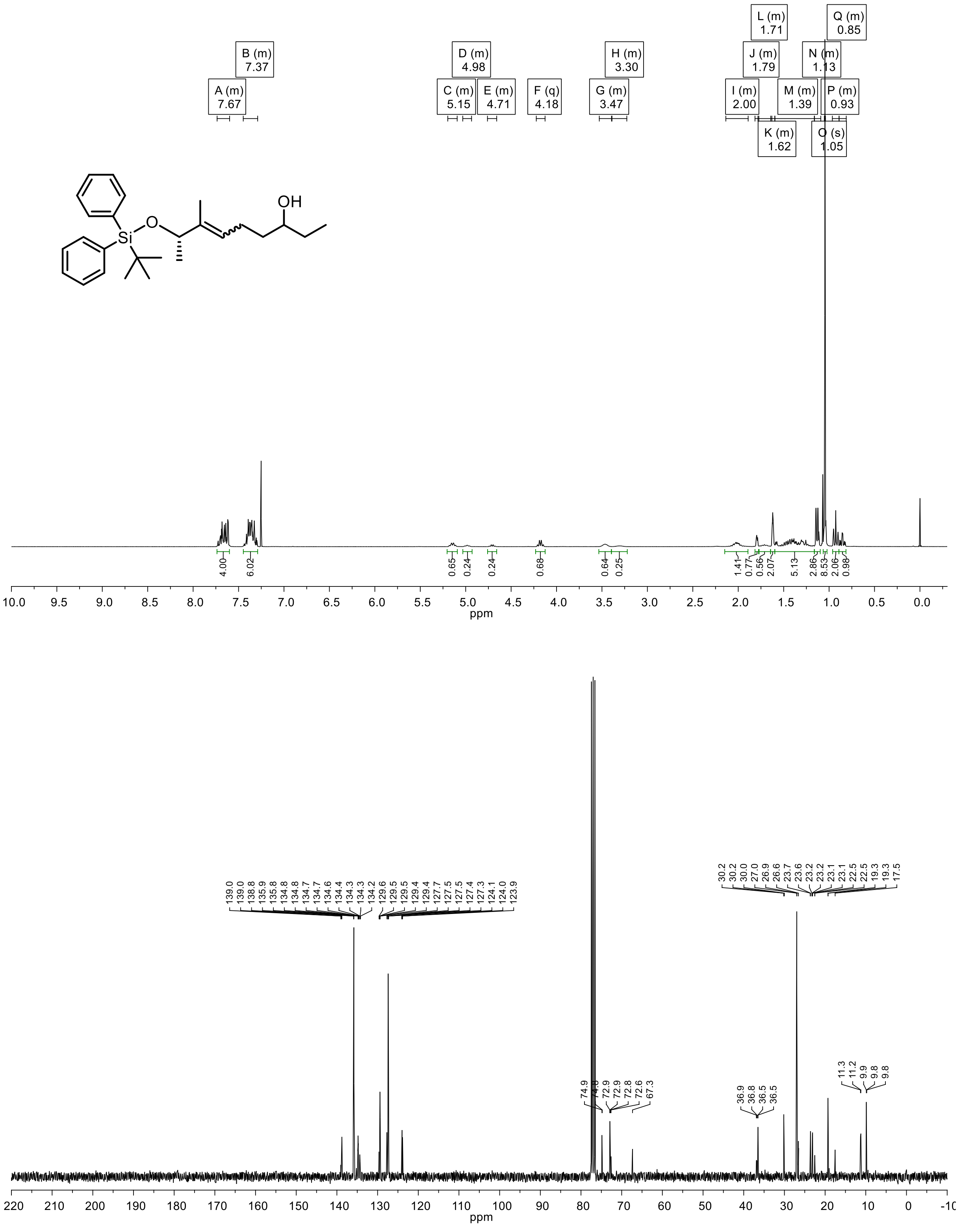

Fig. S20: Top: ^{1}H NMR (300 MHz, $CDCl_3$) and bottom: ^{13}C NMR spectrum (75 MHz, $CDCl_3$) of **39**.

5-(((8*S*)-8-((*tert*-Butyldiphenylsilyl)oxy)-7-methylnon-6-en-3-yl)thio)-1-phenyl-1*H*-tetrazole (**40**)

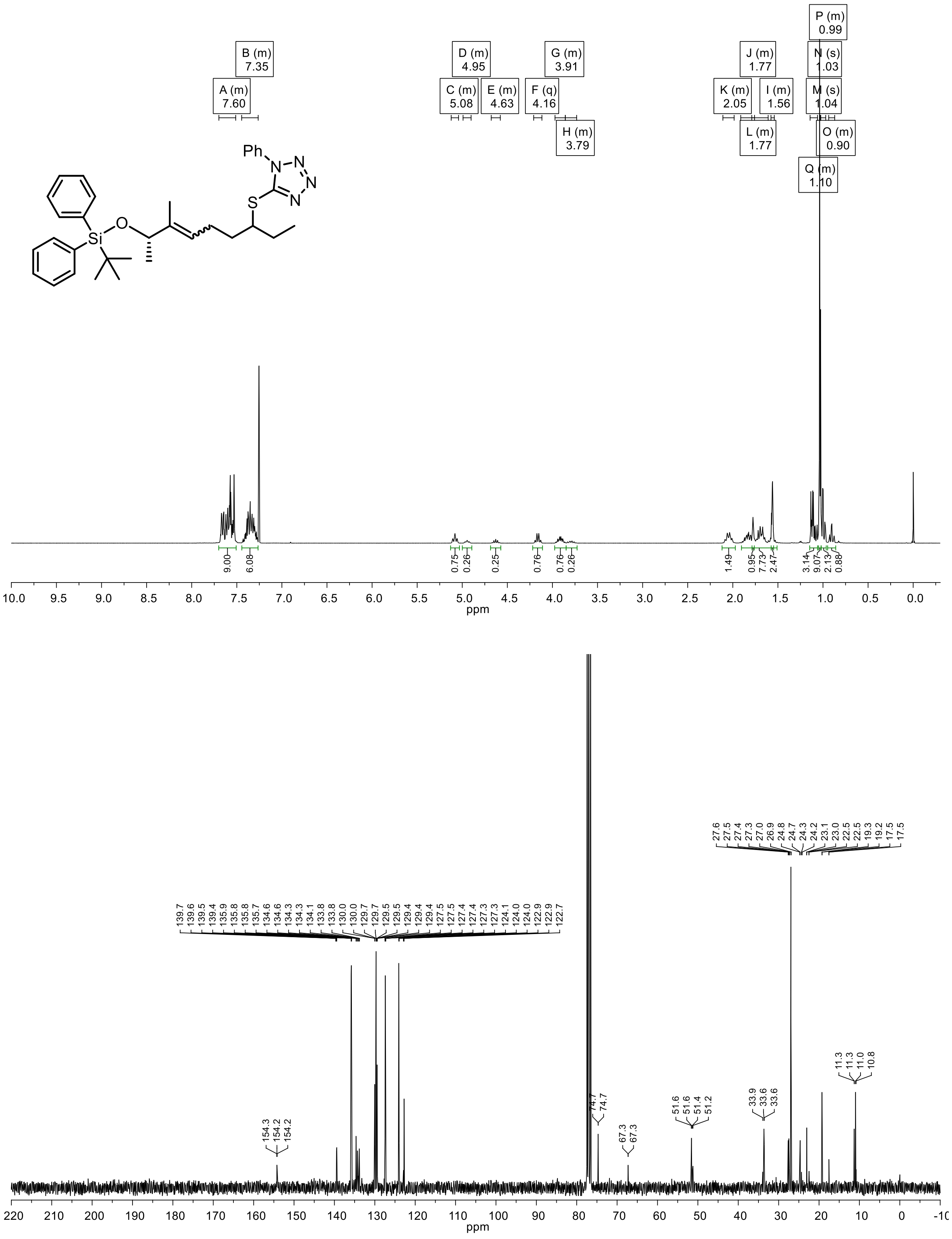

Fig. S21: Top: ^{1}H NMR (300 MHz, $CDCl_3$) and bottom: ^{13}C NMR spectrum (75 MHz, $CDCl_3$) of **40**.

5-(((8*S*)-8-((*tert*-Butyldiphenylsilyl)oxy)-7-methylnon-6-en-3-yl)sulfonyl)-1-phenyl-1*H*-tetrazole (**28**)

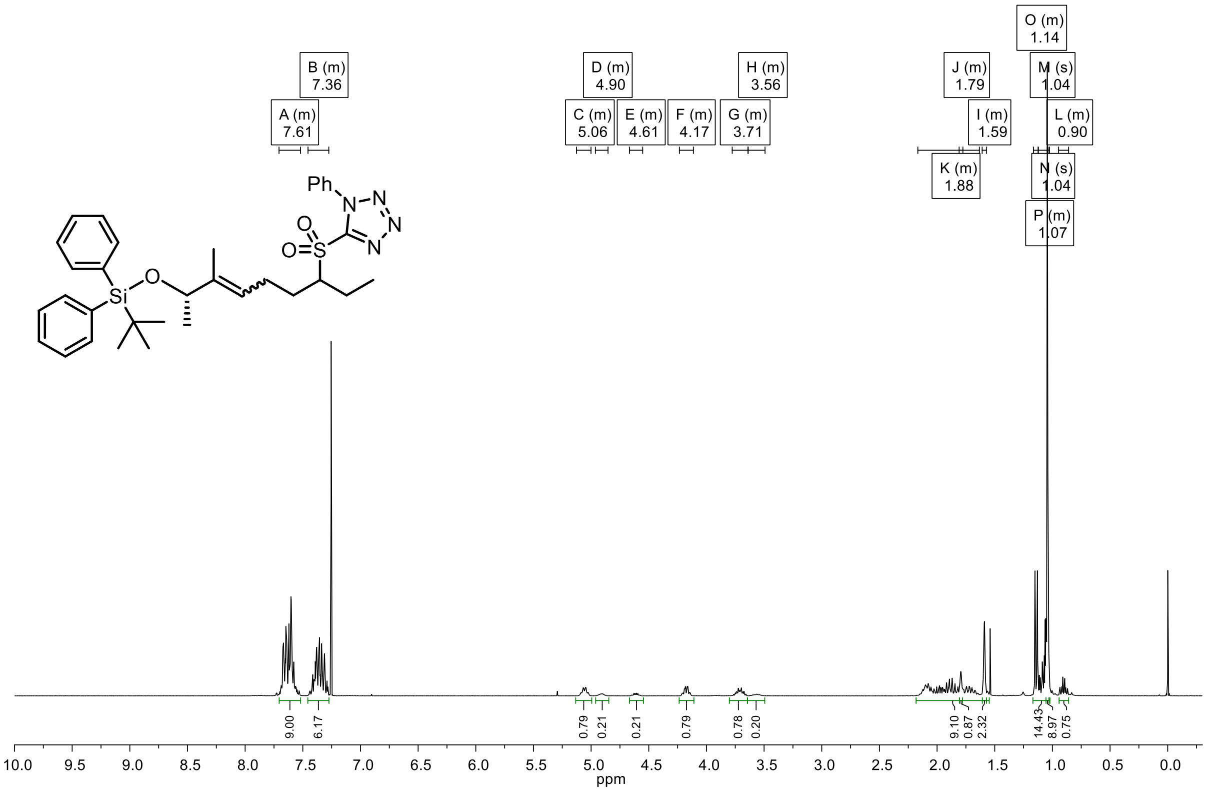

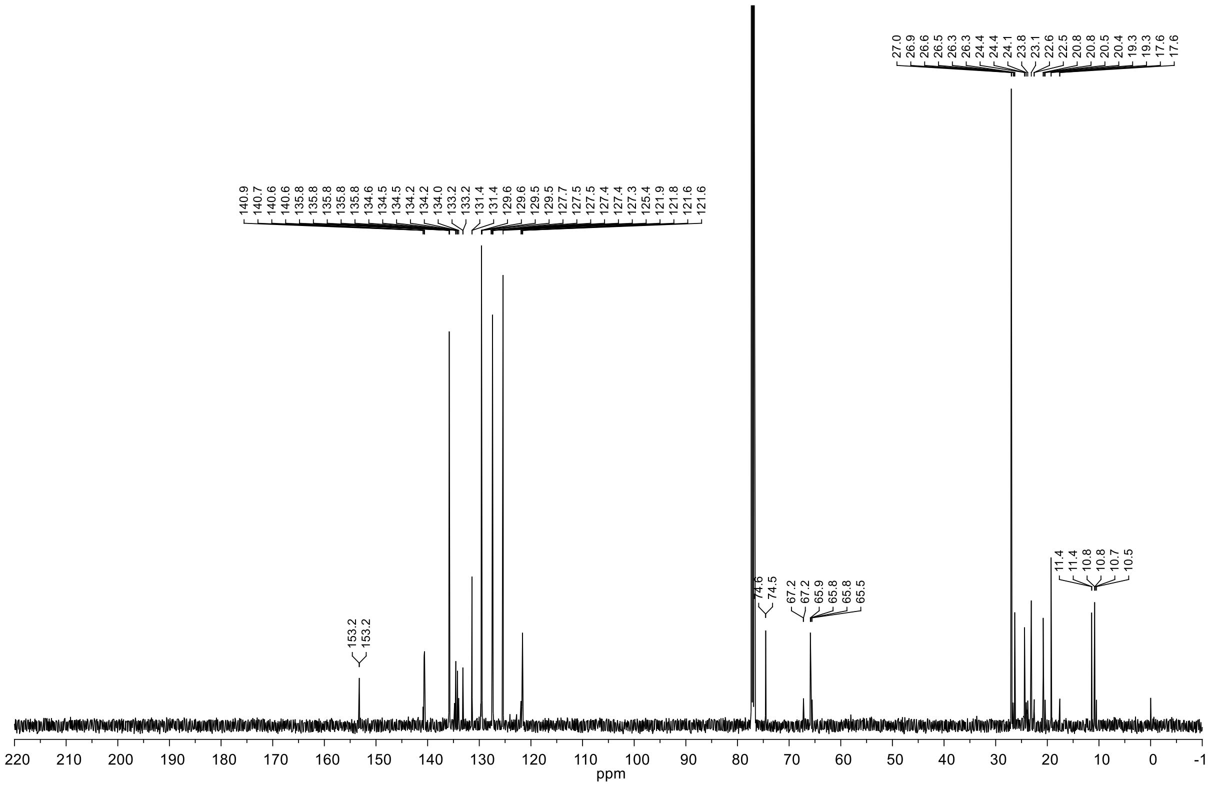

Fig. S22: Top: ^{1}H NMR (500 MHz, $CDCl_3$) and bottom: ^{13}C NMR spectrum (125 MHz, $CDCl_3$) of **28**.

(7*S*)-7-((*tert*-Butyldiphenylsilyl)oxy)-6-methyloct-5-en-2-ol (**s5**)

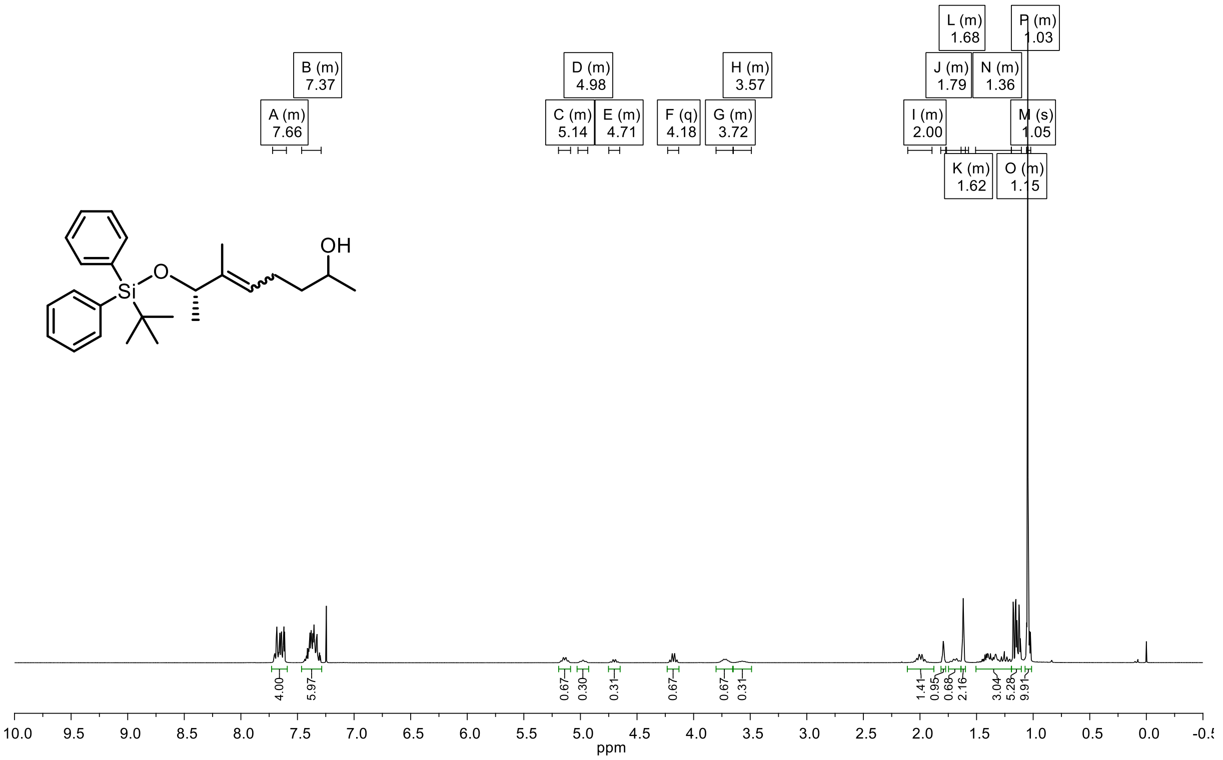

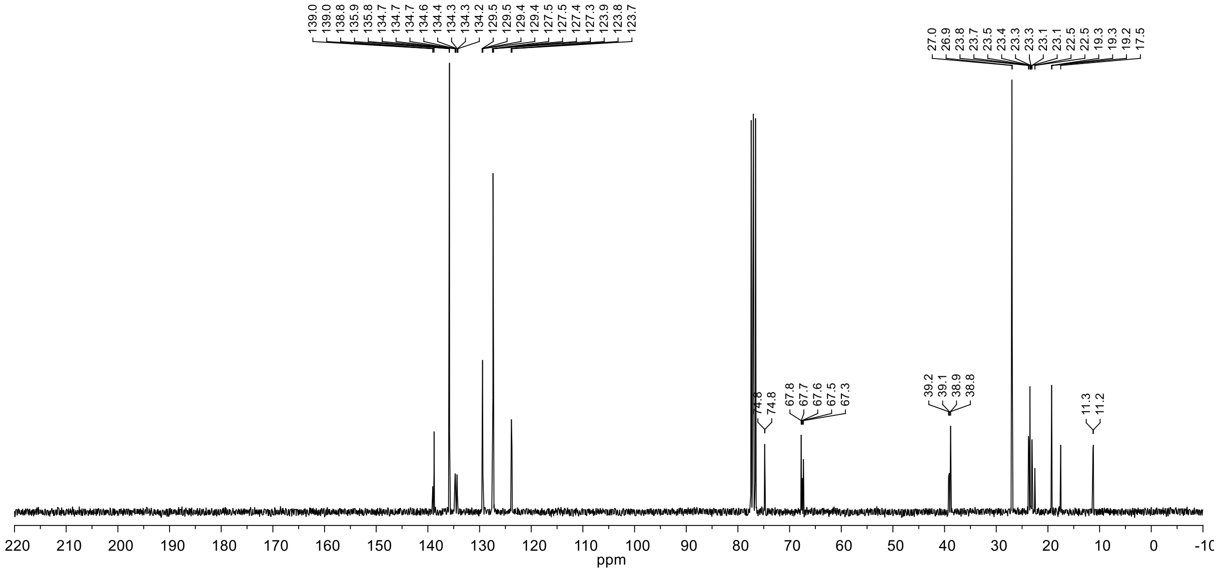

Fig. S23: Top: ^{1}H NMR (300 MHz, $CDCl_3$) and bottom: ^{13}C NMR spectrum (75 MHz, $CDCl_3$) of **s5**.

5-(((7*S*)-7-((*tert*-Butyldiphenylsilyl)oxy)-6-methyloct-5-en-2-yl)thio)-1-phenyl-1*H*-tetrazole (**s6**)

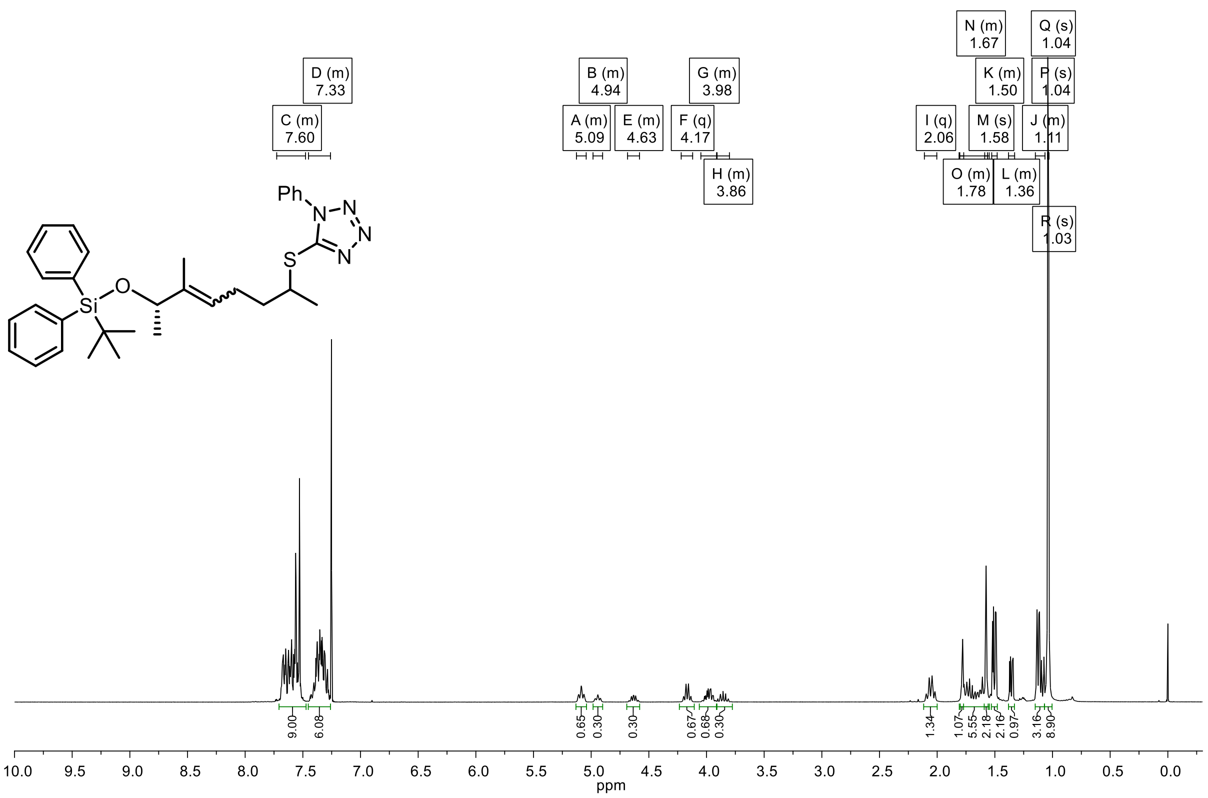

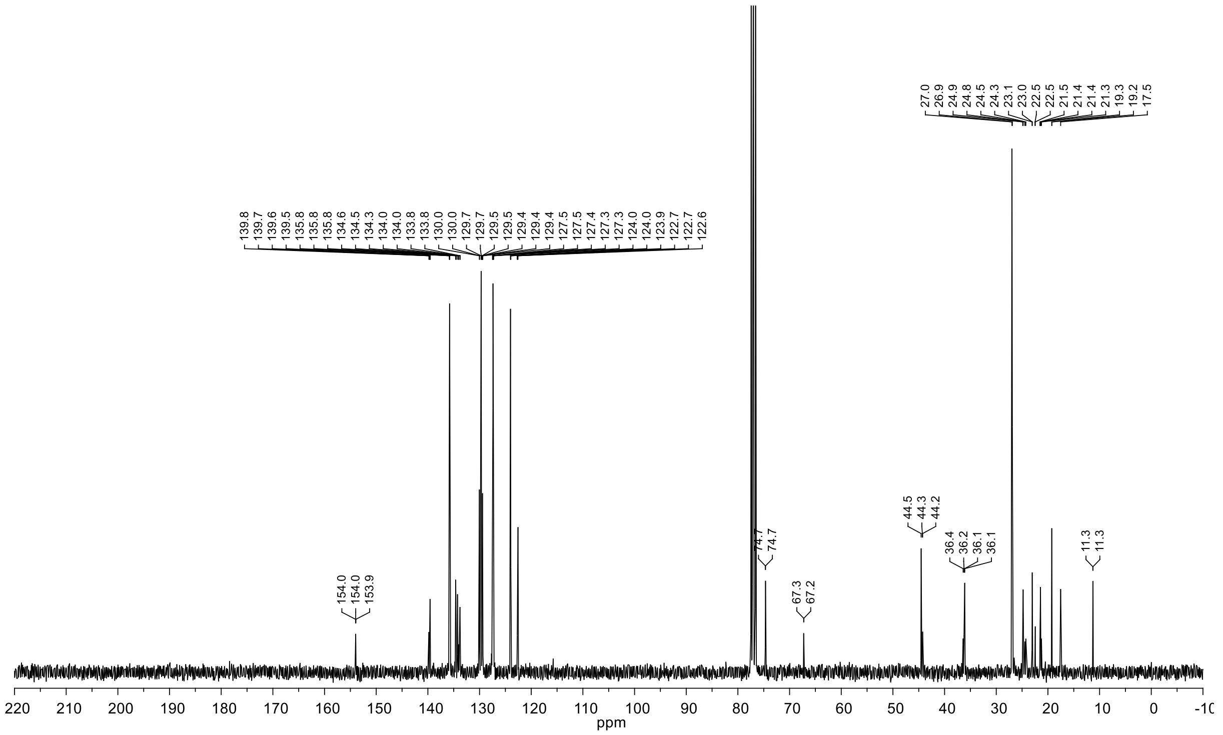

Fig. S24: Top: ^{1}H NMR (300 MHz, $CDCl_3$) and bottom: ^{13}C NMR spectrum (75 MHz, $CDCl_3$) of **s6**.

5-(((7*S*)-7-((*tert*-Butyldiphenylsilyl)oxy)-6-methyloct-5-en-2-yl)sulfonyl)-1-phenyl-1*H*-tetrazole (**27**)

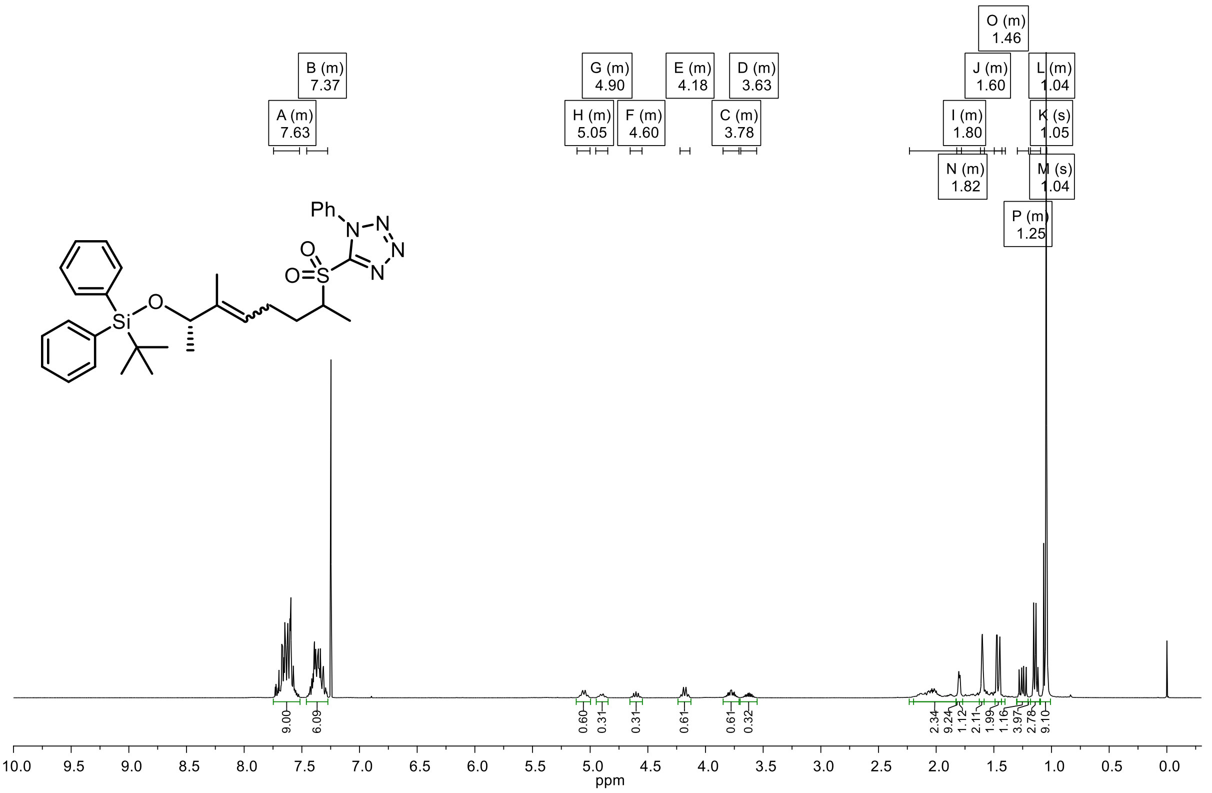

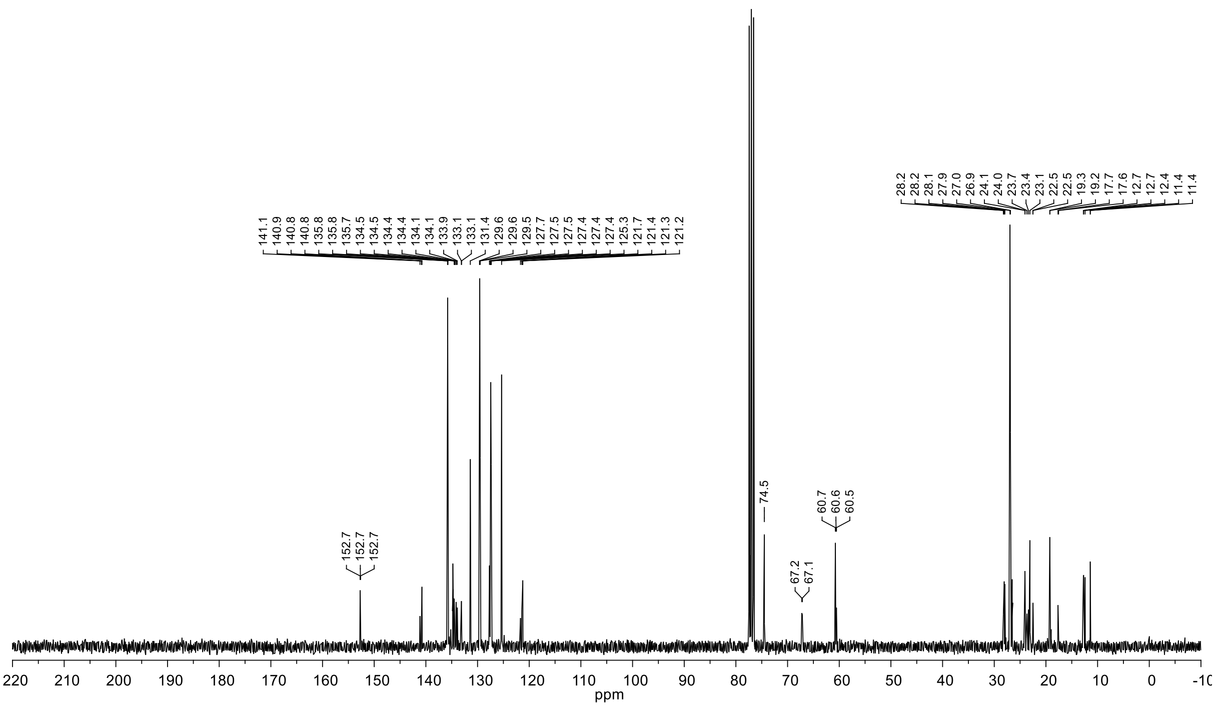

Fig. S25: Top: ^{1}H NMR (300 MHz, $CDCl_3$) and bottom: ^{13}C NMR spectrum (75 MHz, $CDCl_3$) of **27**.

Methyl (3*R*,12*S*)-12-((*tert*-butyldiphenylsilyl)oxy)-3,7-diethyl-11-methyltrideca-6,10-di-enoate (**41**)

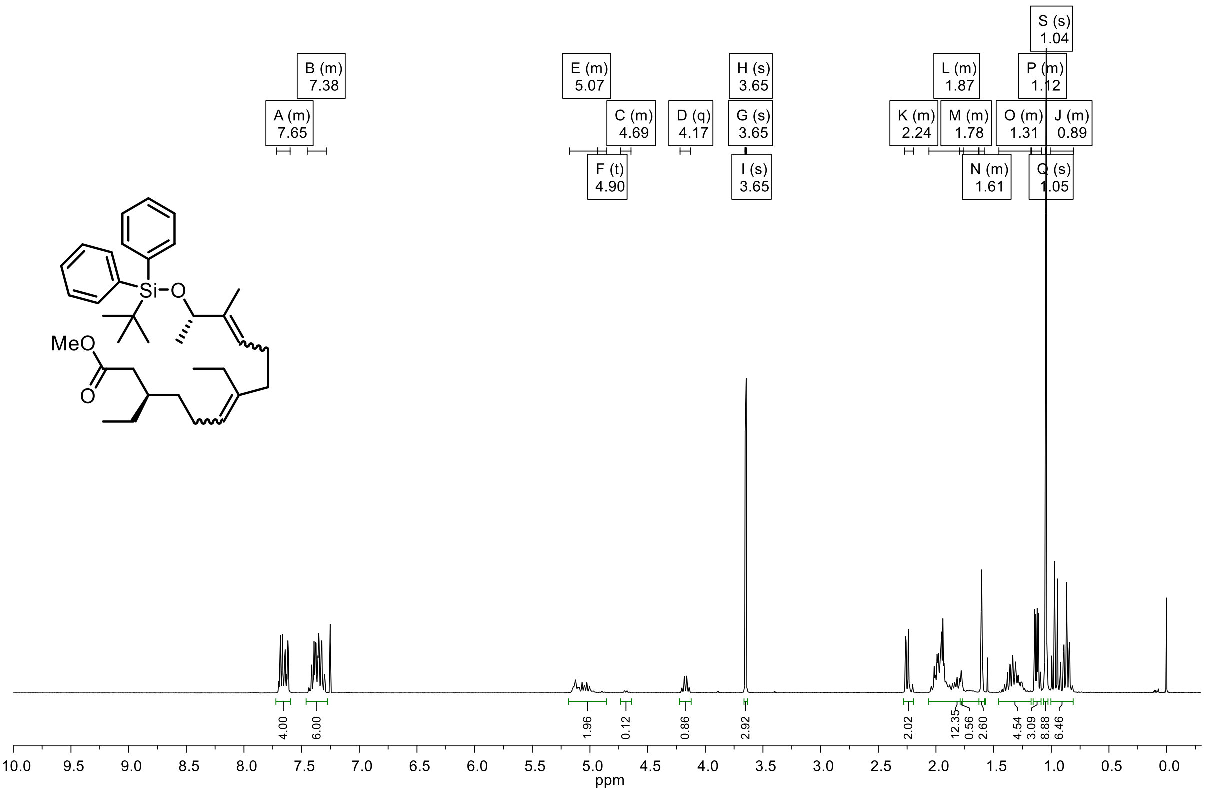

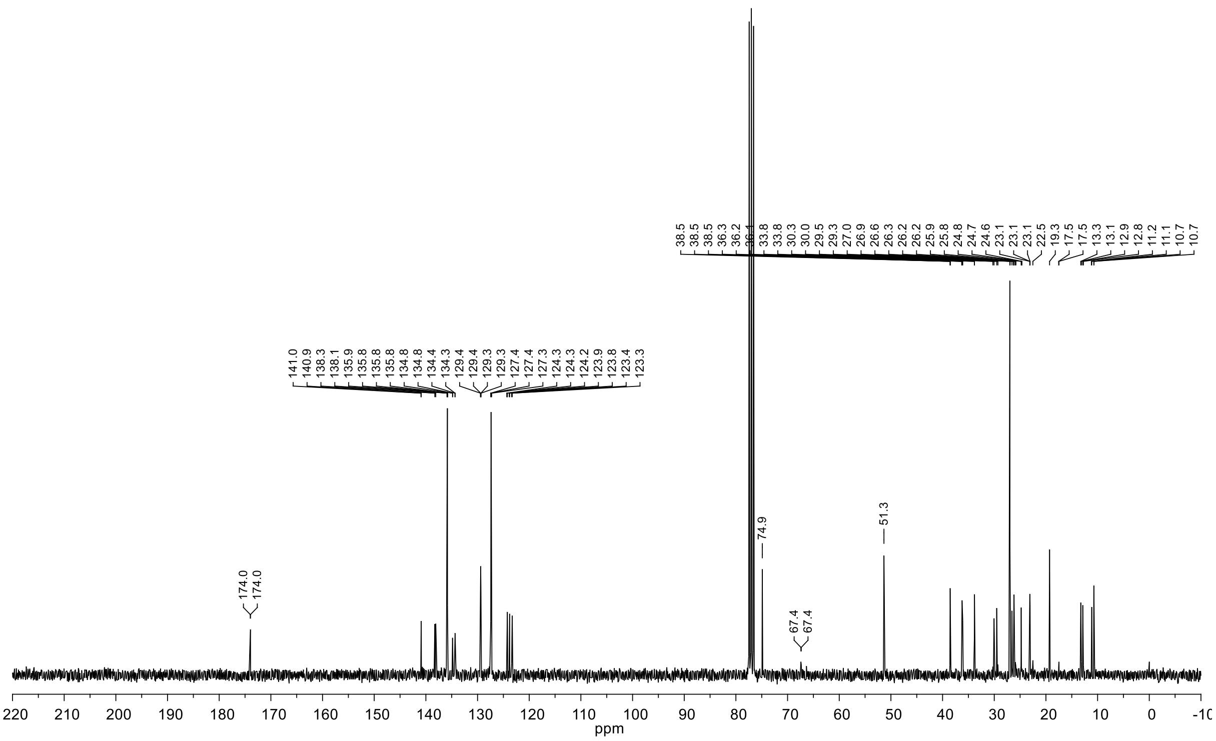

Fig. S26: Top: ^{1}H NMR (300 MHz, $CDCl_3$) and bottom: ^{13}C NMR spectrum (75 MHz, $CDCl_3$) of **41**.

Methyl (3*S*,12*S*)-12-((*tert*-butyldiphenylsilyl)oxy)-3,7-diethyl-11-methyltrideca-6,10-di-enoate (**41’**)

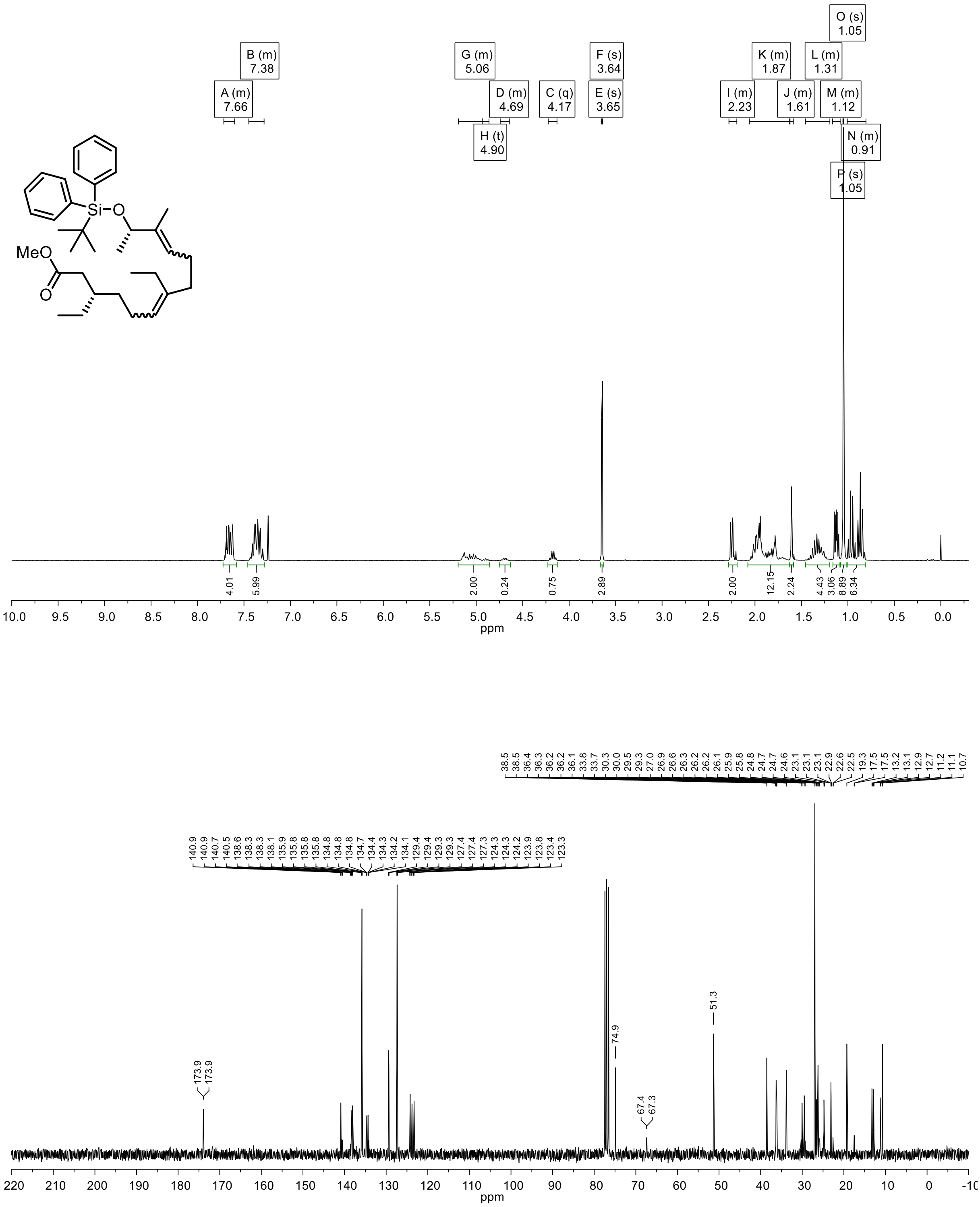

Fig. S27: Top: ^{1}H NMR (300 MHz, $CDCl_3$) and bottom: ^{13}C NMR spectrum (75 MHz, $CDCl_3$) of **41’**.

Methyl (3*R*,12*S*)-12-((*tert*-butyldiphenylsilyl)oxy)-3,7,11-trimethyltrideca-6,10-dien-oate (**44**)

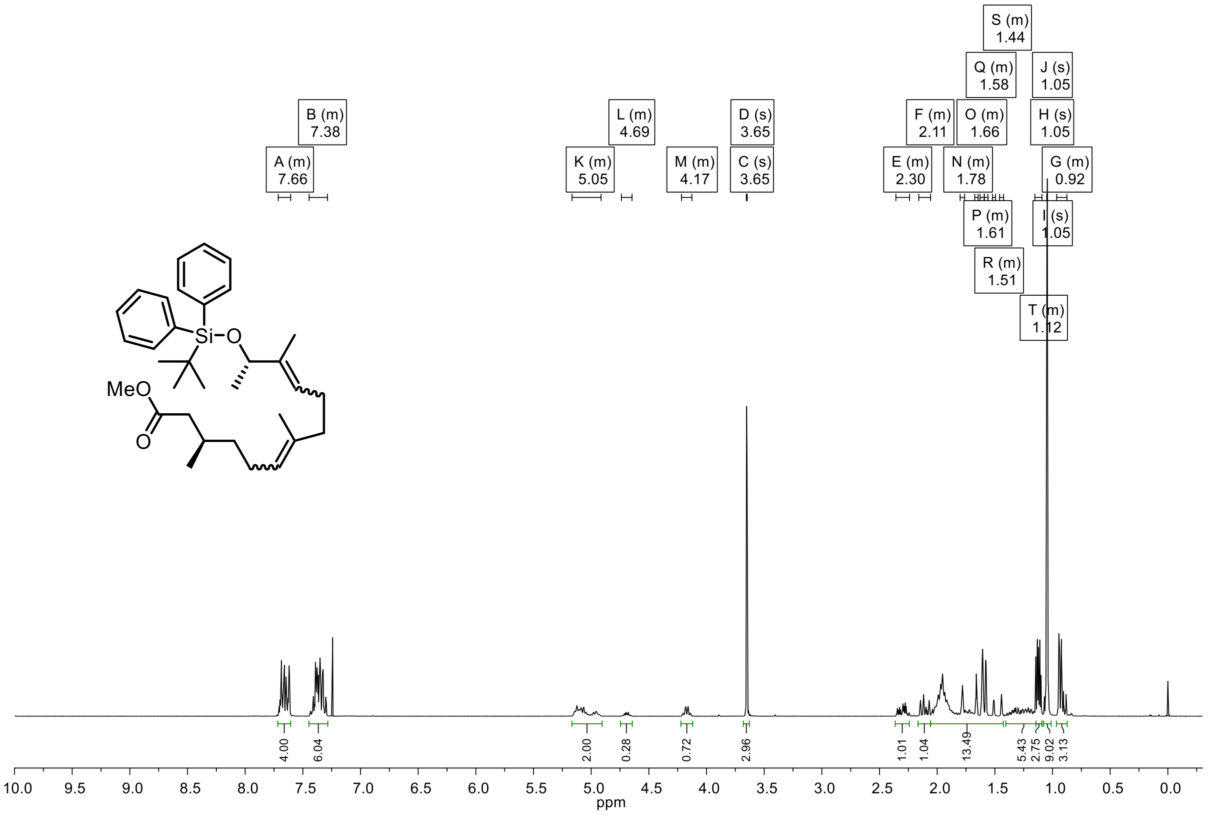

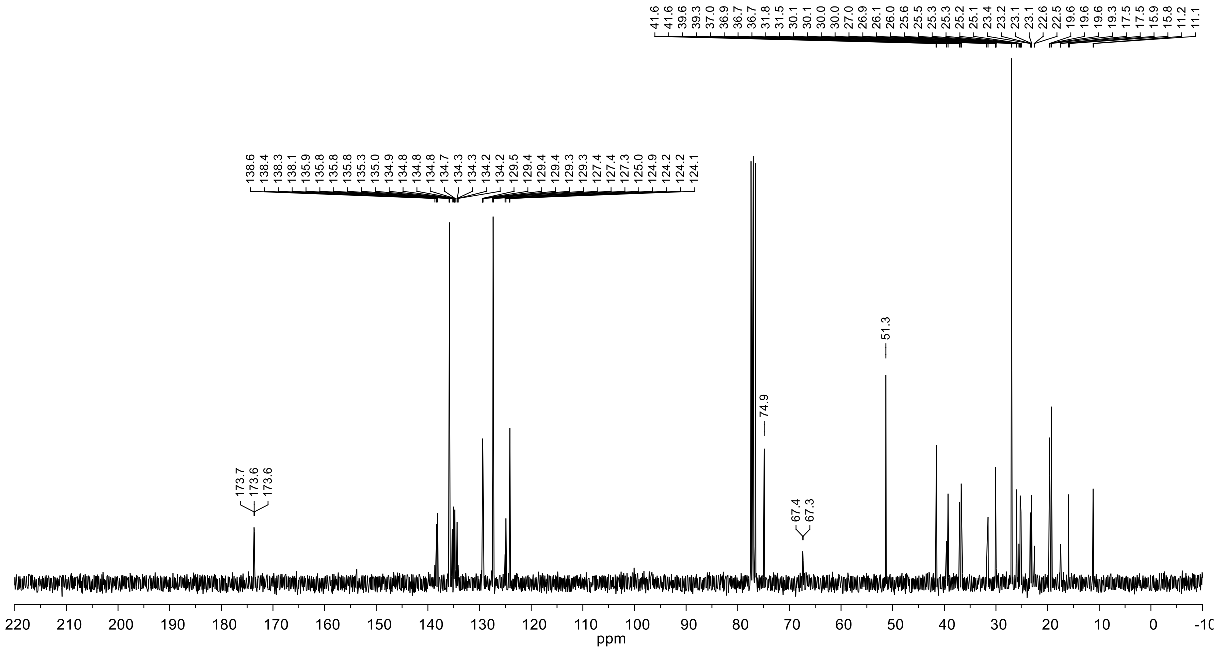

Fig. S28: Top: ^{1}H NMR (300 MHz, $CDCl_3$) and bottom: ^{13}C NMR spectrum (75 MHz, $CDCl_3$) of **44**.

Methyl (3*R*,12*S*)-12-((*tert*-butyldiphenylsilyl)oxy)-7-ethyl-3,11-dimethyltrideca-6,10-dienoate (**45**)

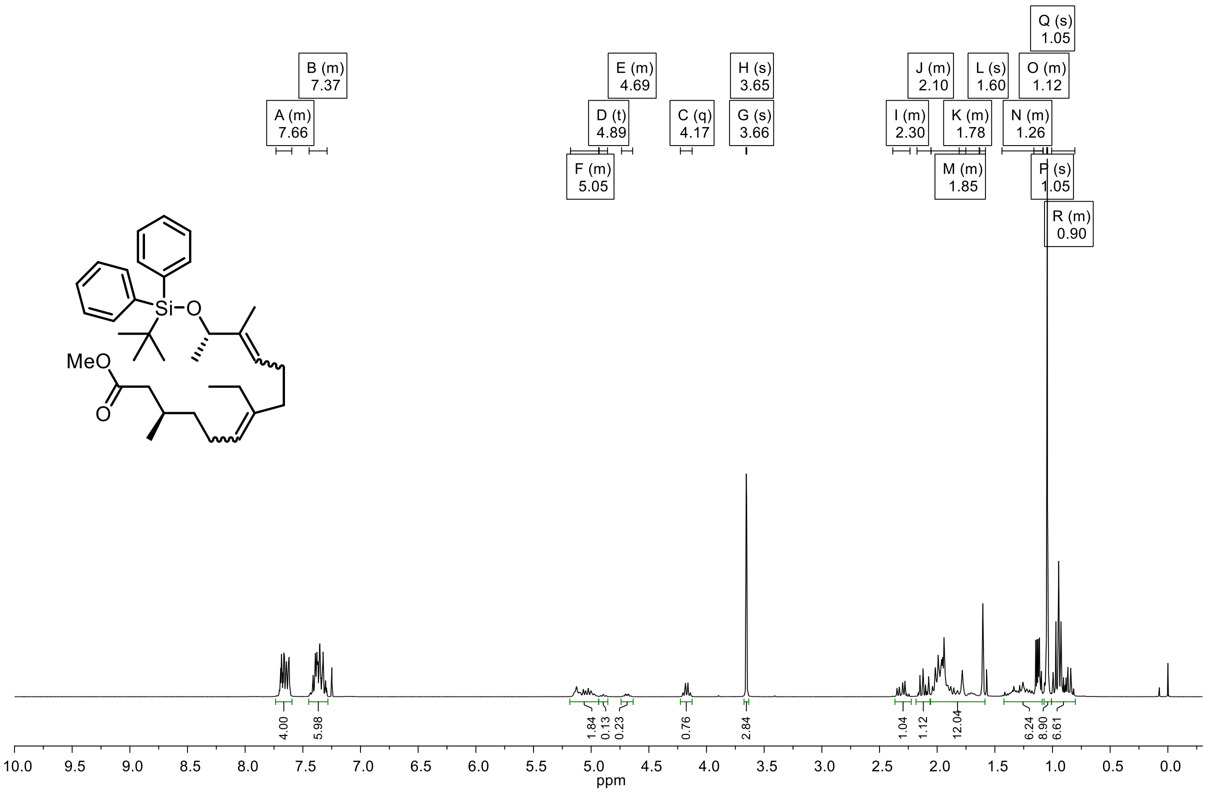

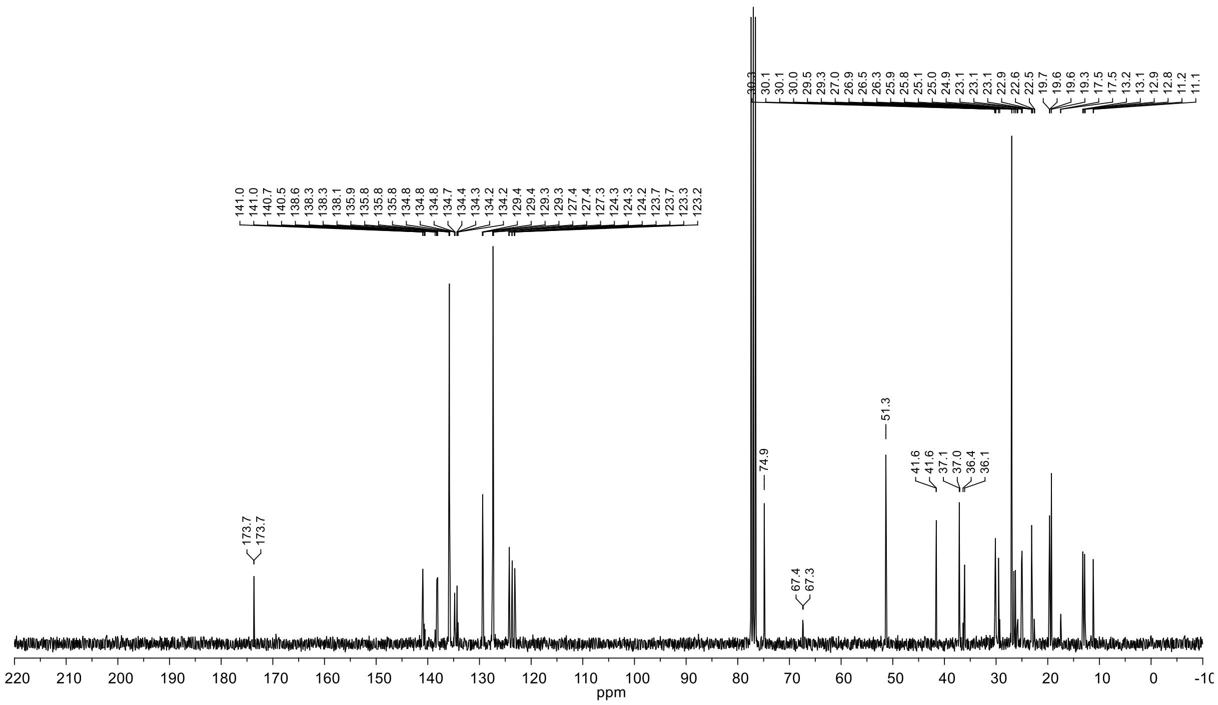

Fig. S29: Top: ^{1}H NMR (300 MHz, $CDCl_3$) and bottom: ^{13}C NMR spectrum (75 MHz, $CDCl_3$) of **45**.

Methyl (3*R*,12*S*)-12-((*tert*-butyldiphenylsilyl)oxy)-3-ethyl-7,11-dimethyltrideca-6,10-dienoate (**46**)

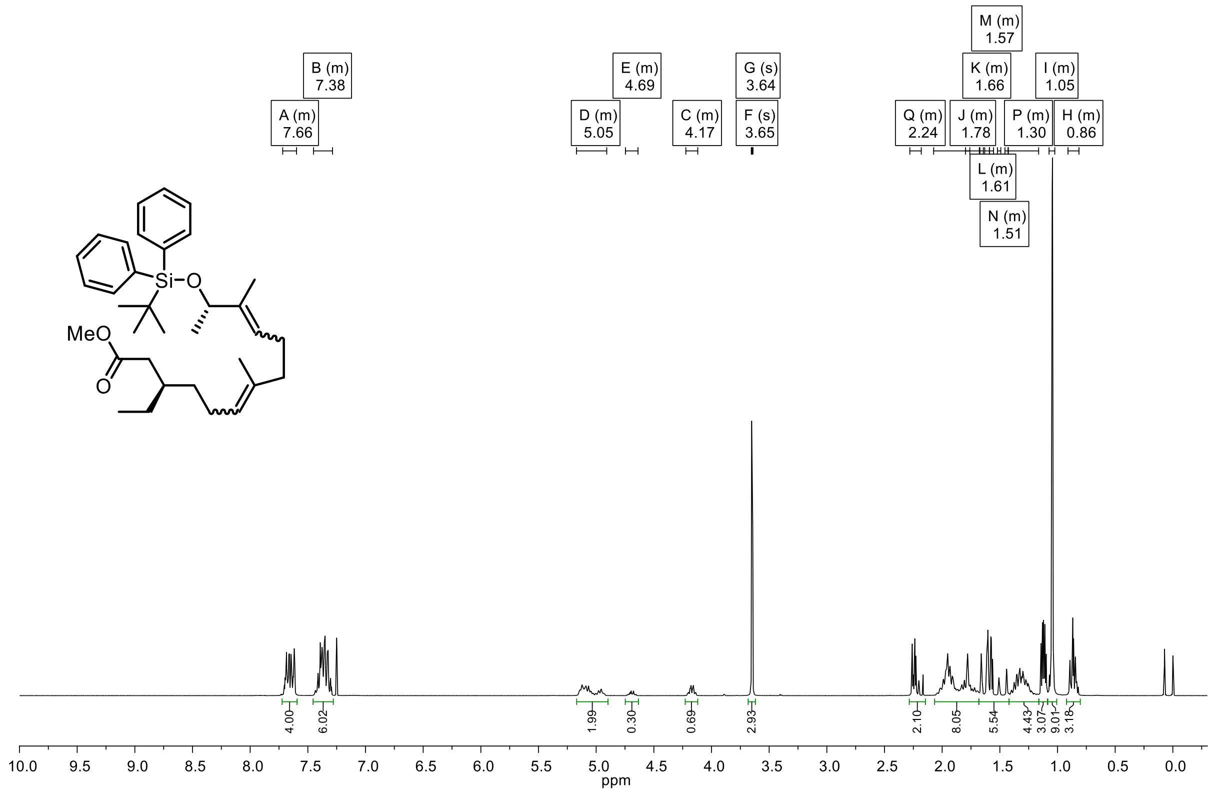

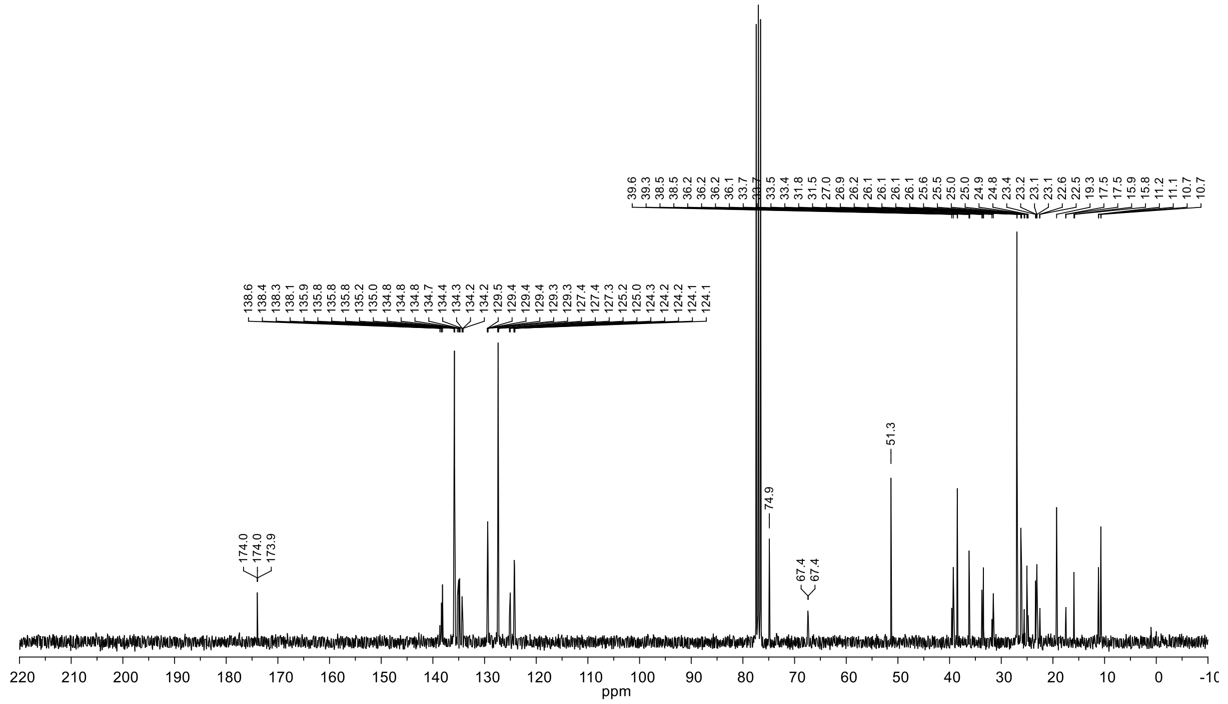

Fig. S30: Top: ^{1}H NMR (300 MHz, $CDCl_3$) and bottom: ^{13}C NMR spectrum (75 MHz, $CDCl_3$) of **46**.

Methyl (3*R*,12*S*)-3,7-diethyl-12-hydroxy-11-methyltrideca-6,10-dienoate (**s7**)

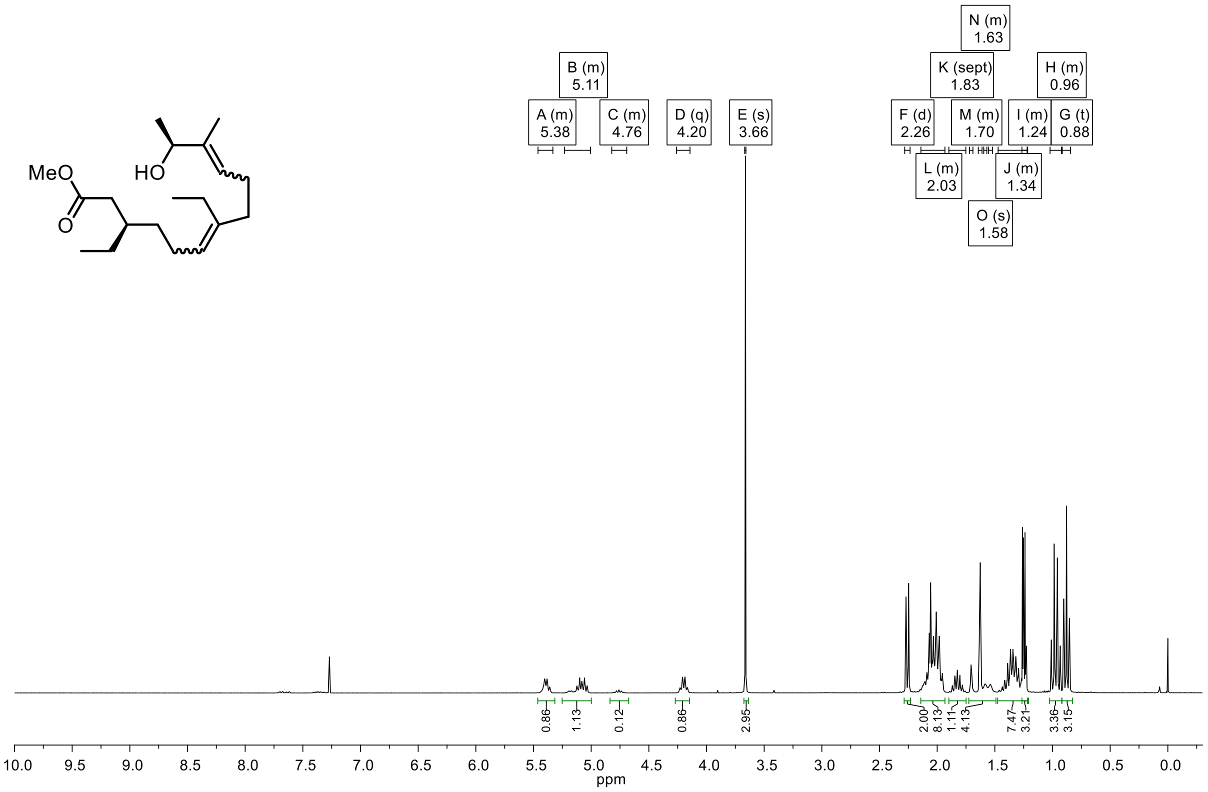

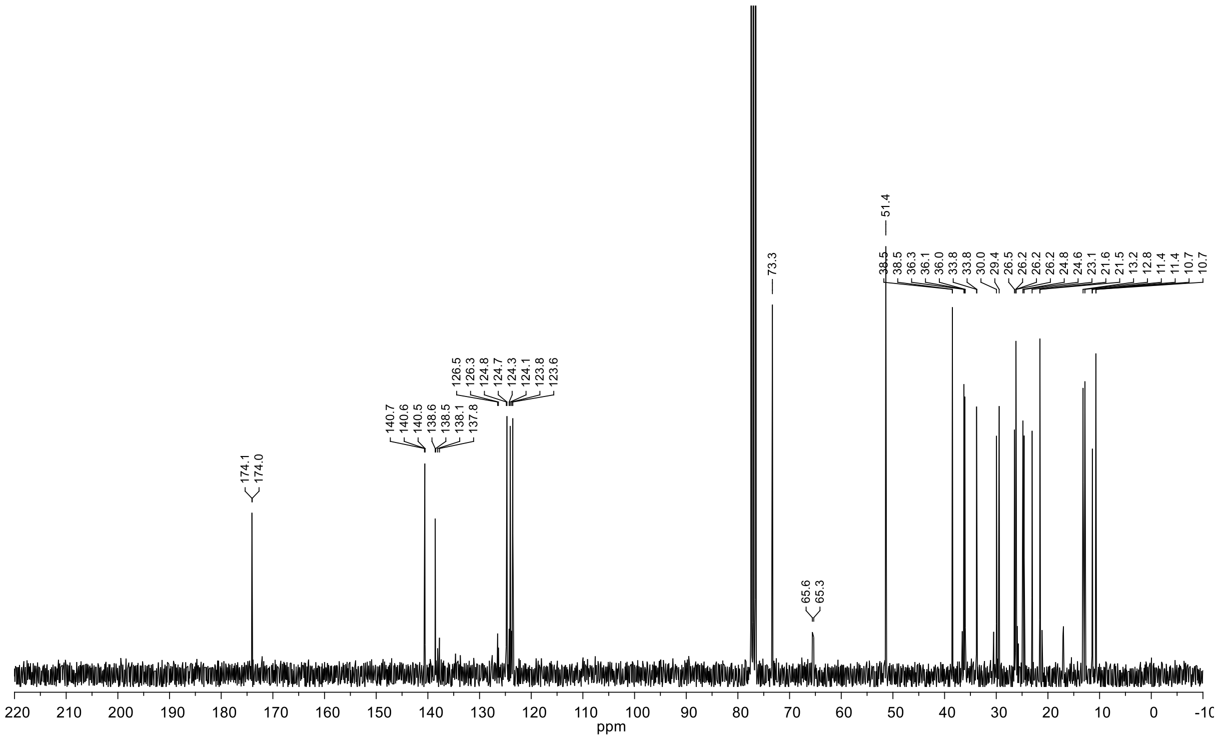

Fig. S31: Top: ^{1}H NMR (300 MHz, $CDCl_3$) and bottom: ^{13}C NMR spectrum (75 MHz, $CDCl_3$) of **s7**.

Methyl (3*S*,12*S*)-3,7-diethyl-12-hydroxy-11-methyltrideca-6,10-dienoate (**s7'**)

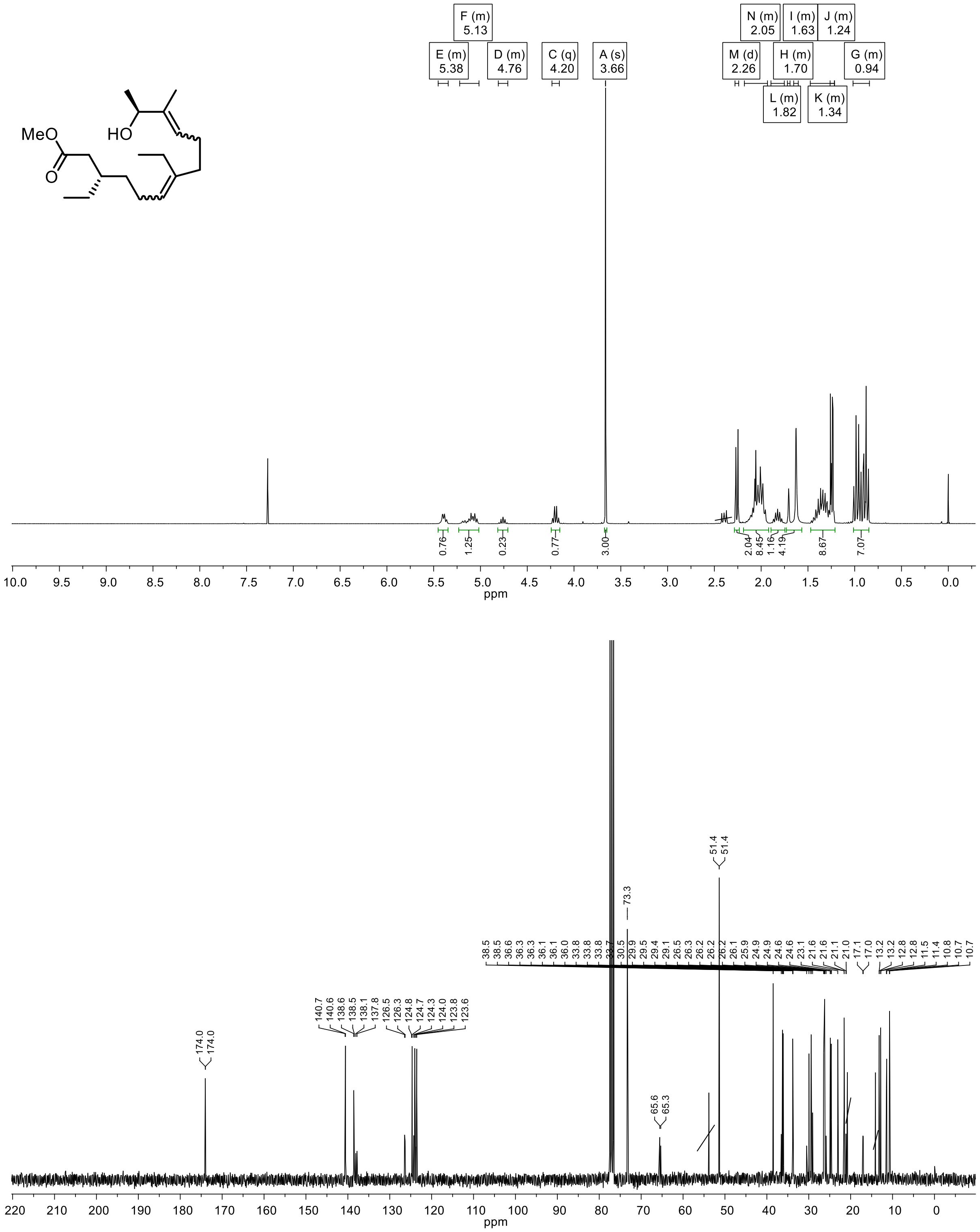

Fig. S32: Top: ^{1}H NMR (300 MHz, $CDCl_3$) and bottom: ^{13}C NMR spectrum (75 MHz, $CDCl_3$) of **s7'**.

Methyl (3*R*,12*S*)-12-hydroxy-3,7,11-trimethyltrideca-6,10-dienoate (**s8**)

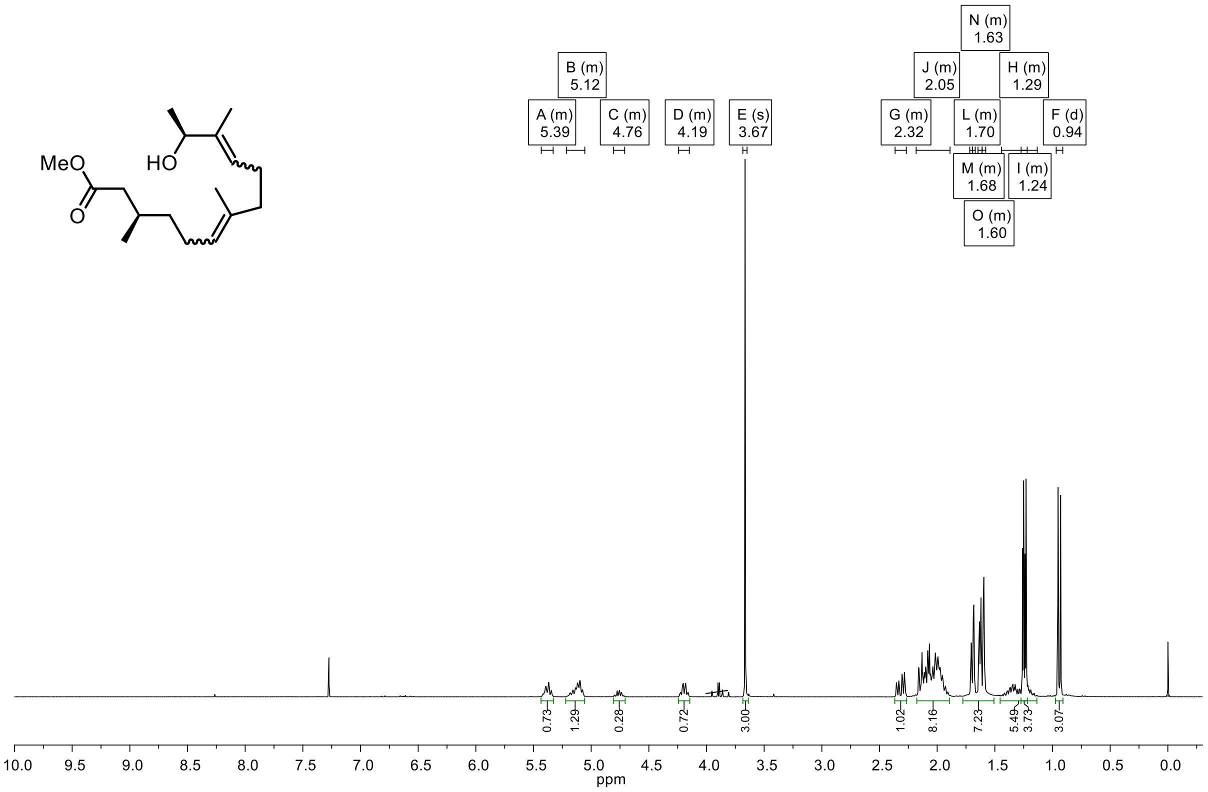

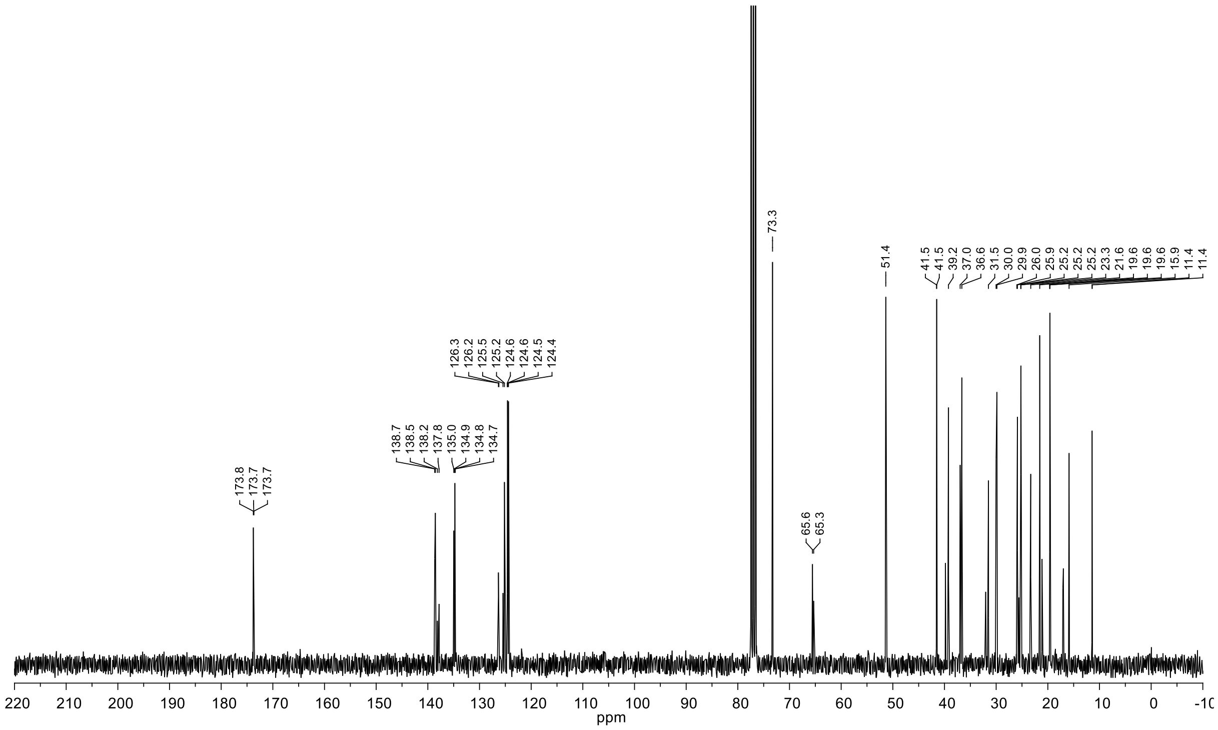

Fig. S33: Top: ^{1}H NMR (300 MHz, $CDCl_3$) and bottom: ^{13}C NMR spectrum (75 MHz, $CDCl_3$) of **s8**.

Methyl (3*R*,12*S*)-7-ethyl-12-hydroxy-3,11-dimethyltrideca-6,10-dienoate (**s9**)

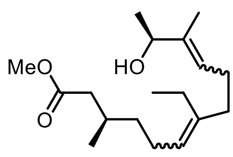

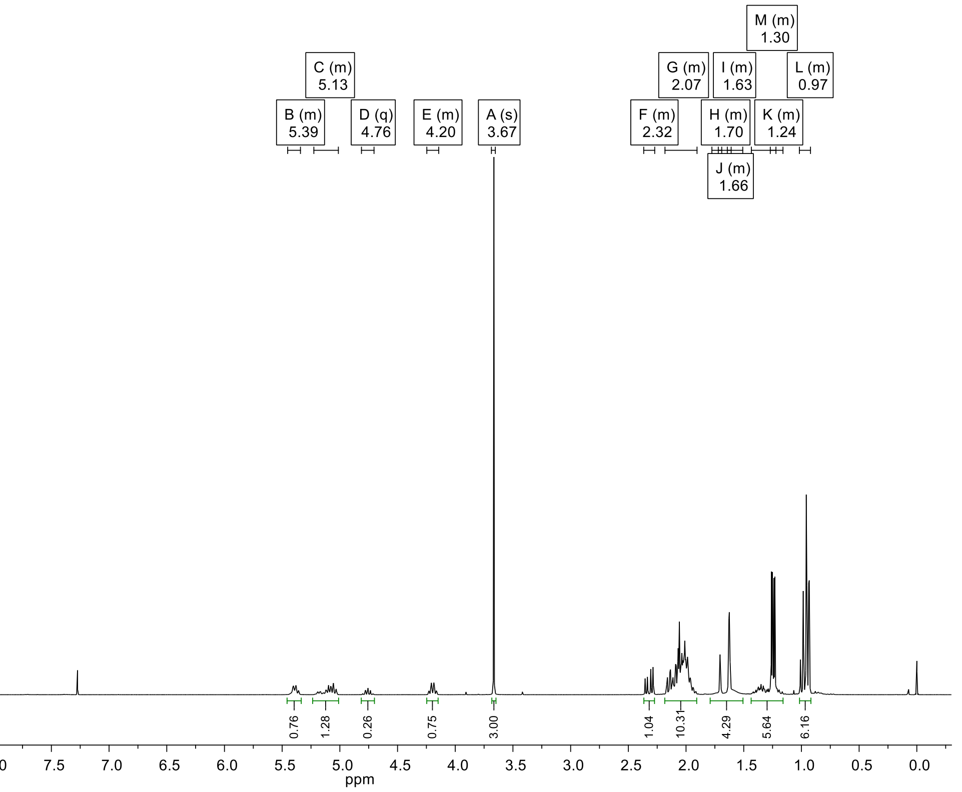

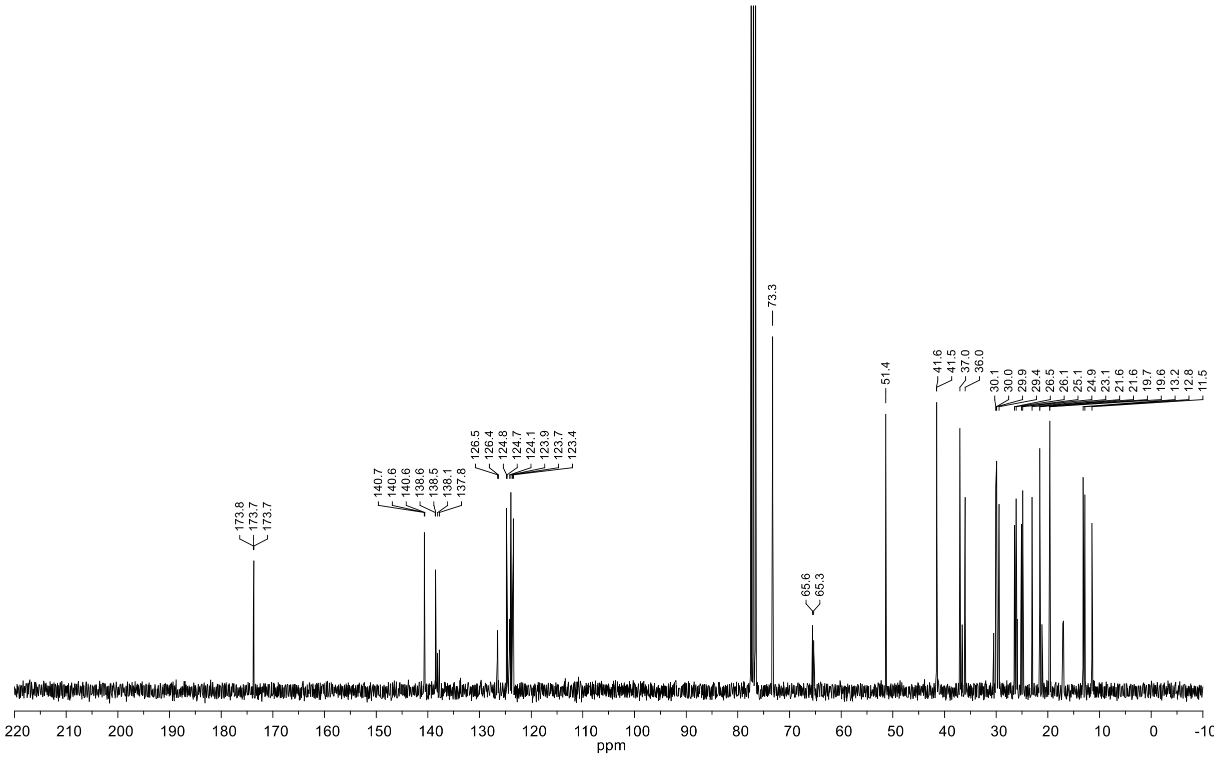

Fig. S34: Top: ^{1}H NMR (300 MHz, $CDCl_3$) and bottom: ^{13}C NMR spectrum (75 MHz, $CDCl_3$) of **s9**.

Methyl (3*R*,12*S*)-3-ethyl-12-hydroxy-7,11-dimethyltrideca-6,10-dienoate (**s10**)

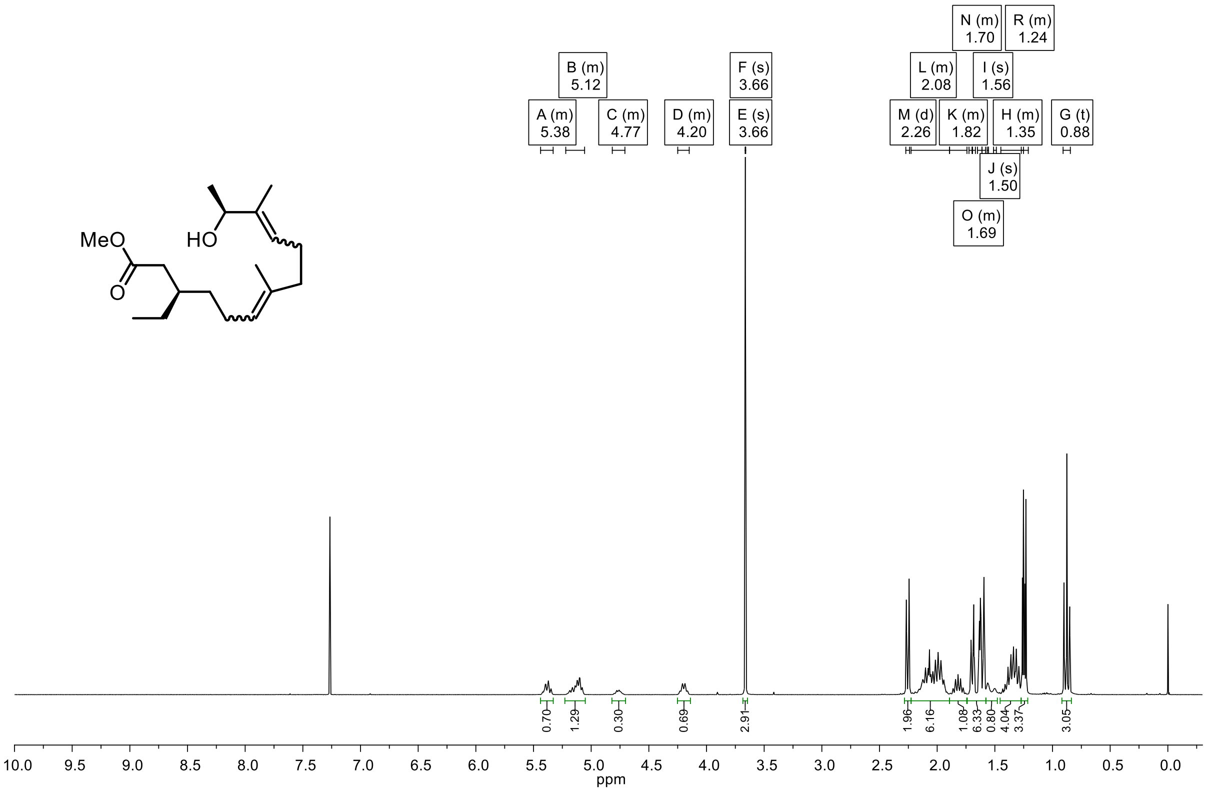

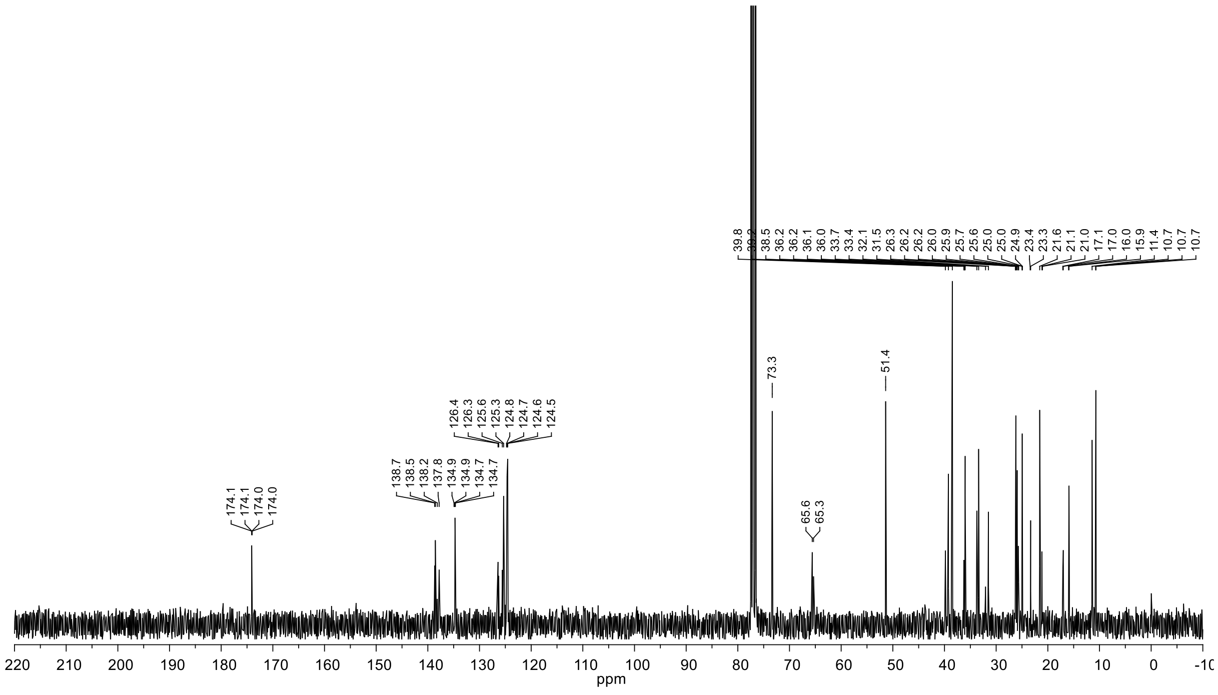

Fig. S35: Top: ^{1}H NMR (300 MHz, $CDCl_3$) and bottom: ^{13}C NMR spectrum (75 MHz, $CDCl_3$) of **s10**.

(3*R*,6*E*,10*E*,12*S*)-3,7-Diethyl-11-methyl-6,10-tridecadien-12-olide (**9**)

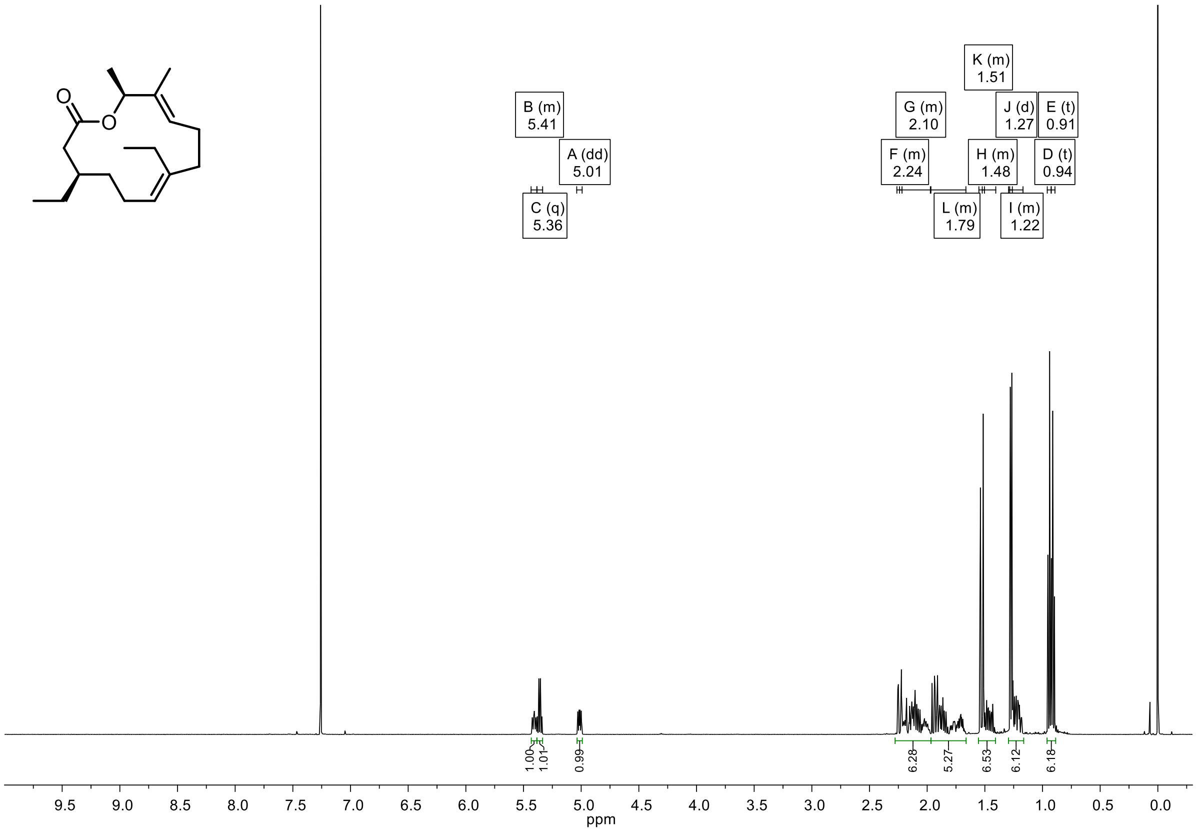

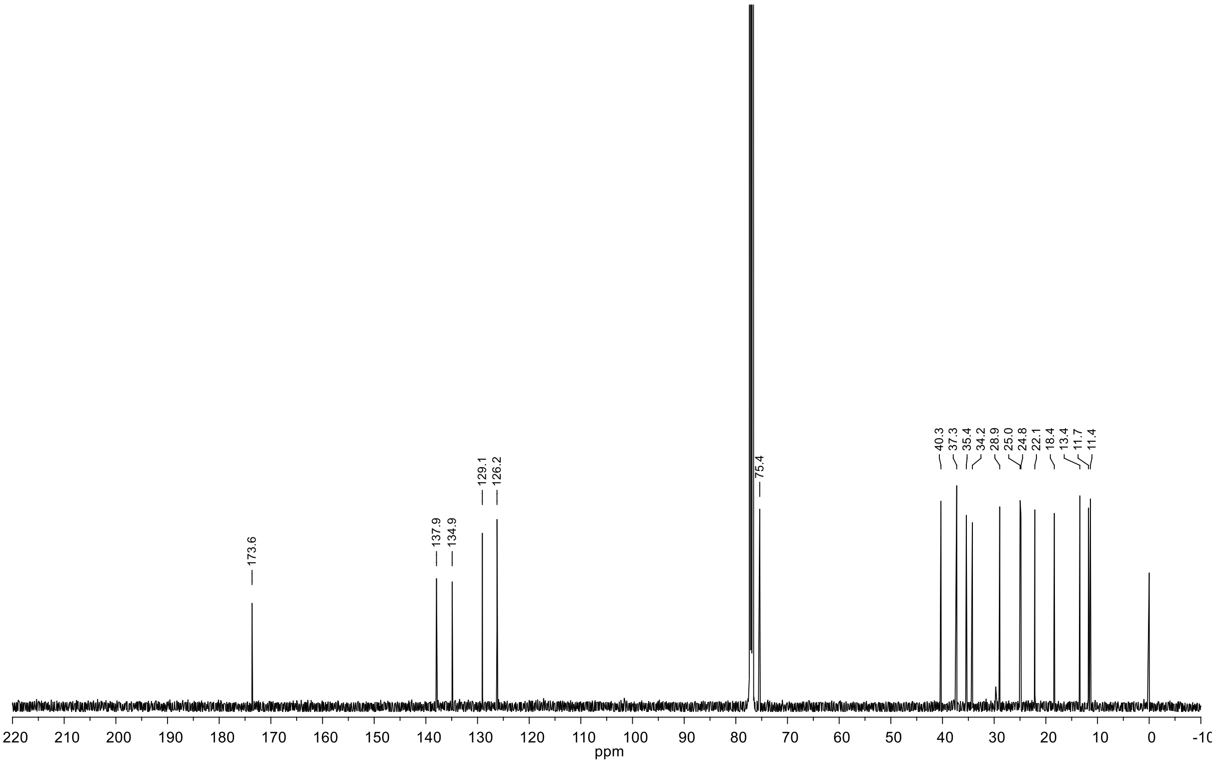

Fig. S36: Top: ^{1}H NMR (500 MHz, $CDCl_3$) and bottom: ^{13}C NMR spectrum (125 MHz, $CDCl_3$) of **9**.

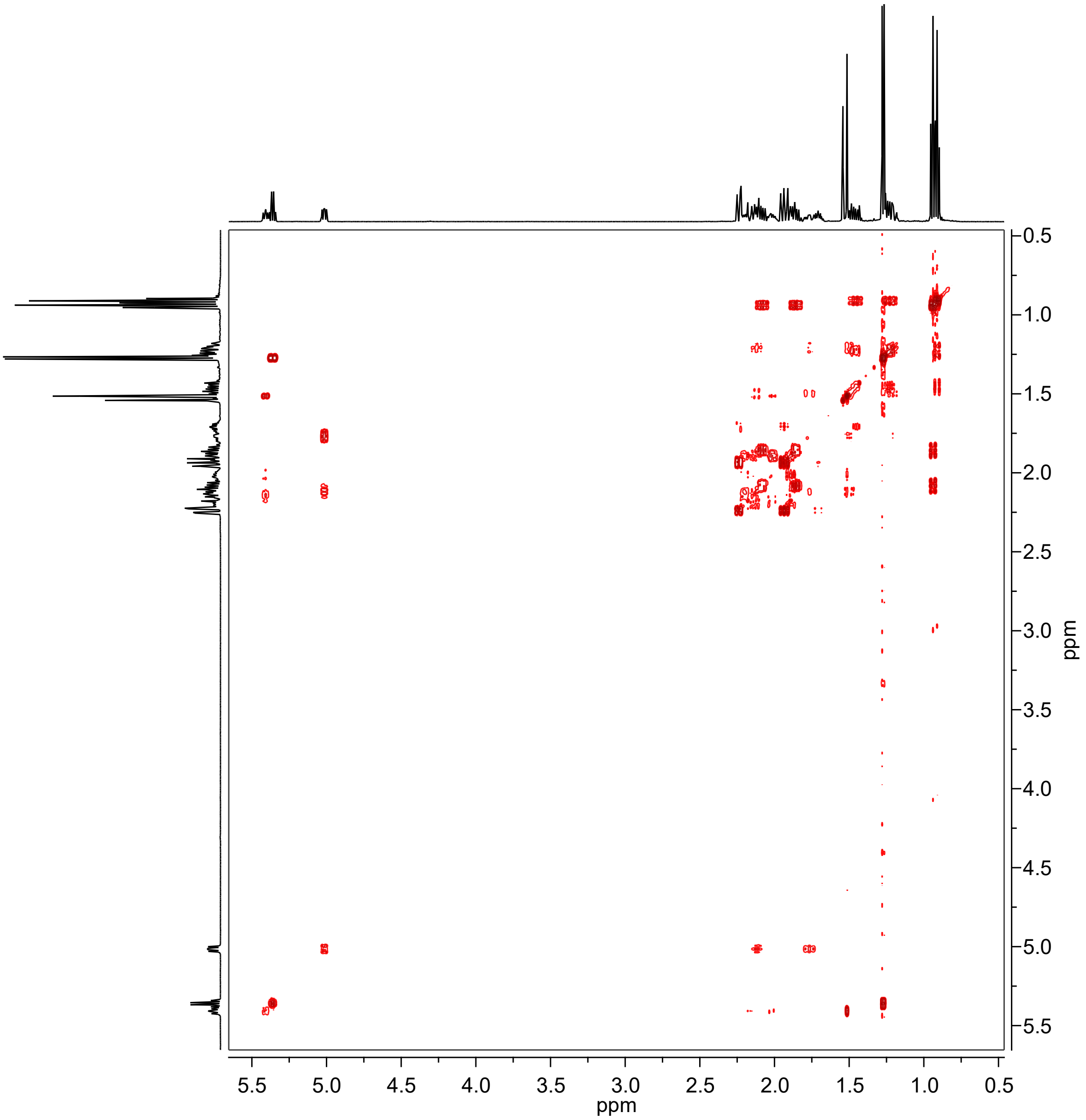

Fig. S37: ^{1}H,^{1}H COSY NMR spectrum (500 MHz, $CDCl_3$) of **9**.

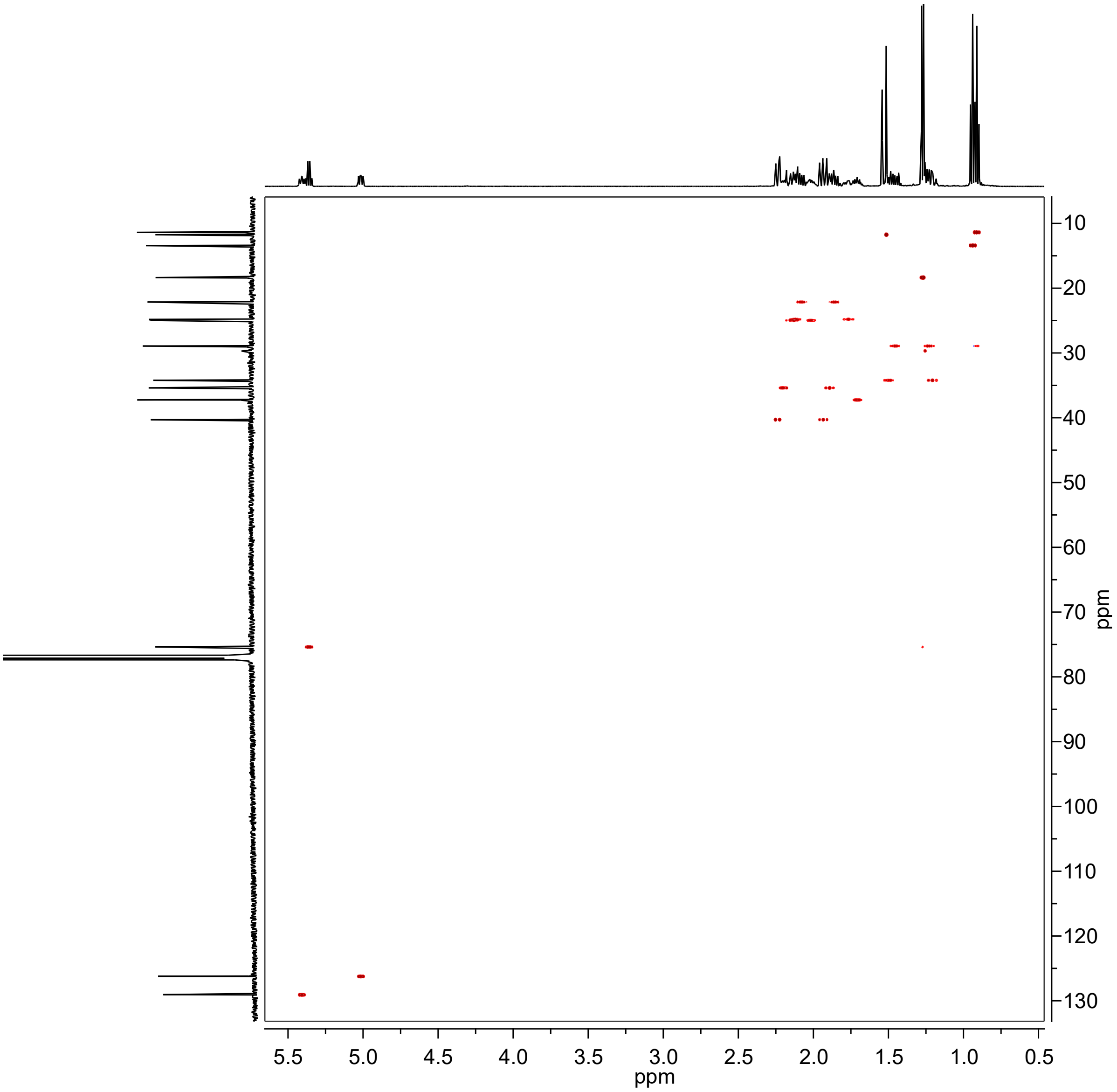

Fig. S38: $^{1}H,^{13}C$ HSQC NMR spectrum (500/125 MHz, $CDCl_3$) of **9**.

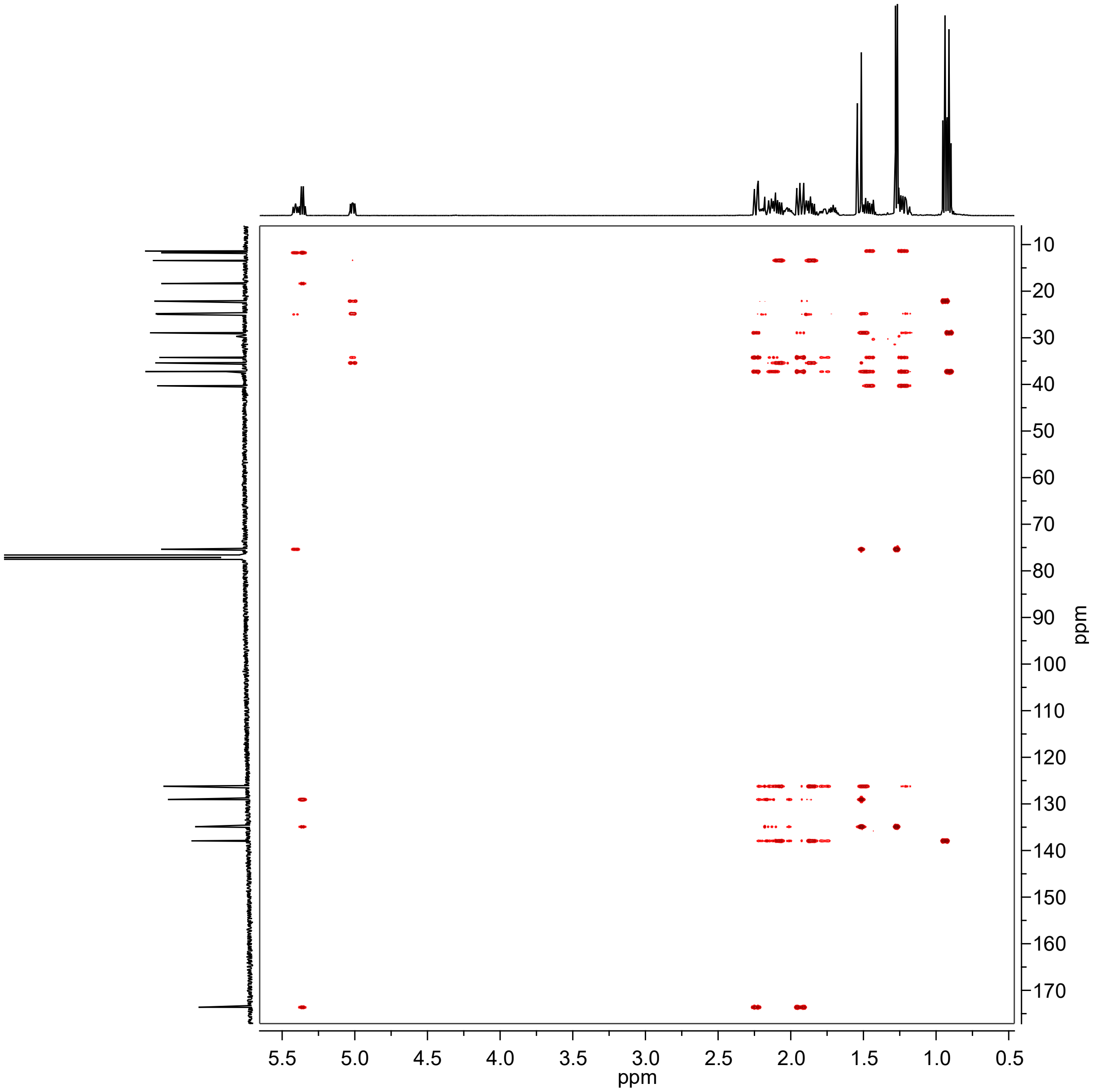

Fig. S39: ^{1}H,^{13}C HMBC NMR spectrum (500/125 MHz, $CDCl_3$) of **9**.

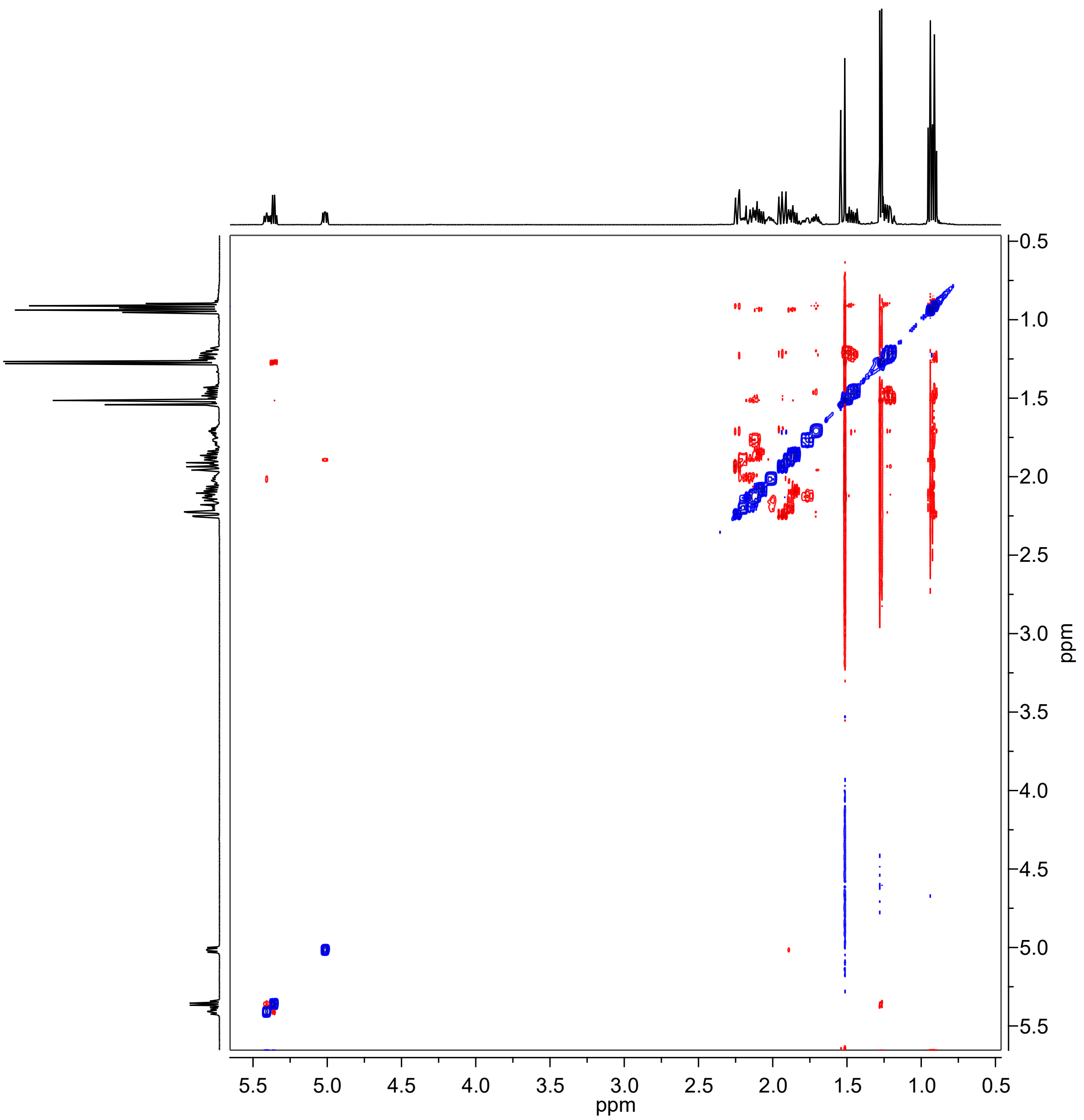

Fig. S40: ^{1}H,^{1}H NOESY NMR spectrum (500 MHz, $CDCl_3$) of **9**.

(3*S*,6*E*,10*E*,12*S*)-3,7-Diethyl-11-methyl-6,10-tridecadien-12-olide (**9’**)

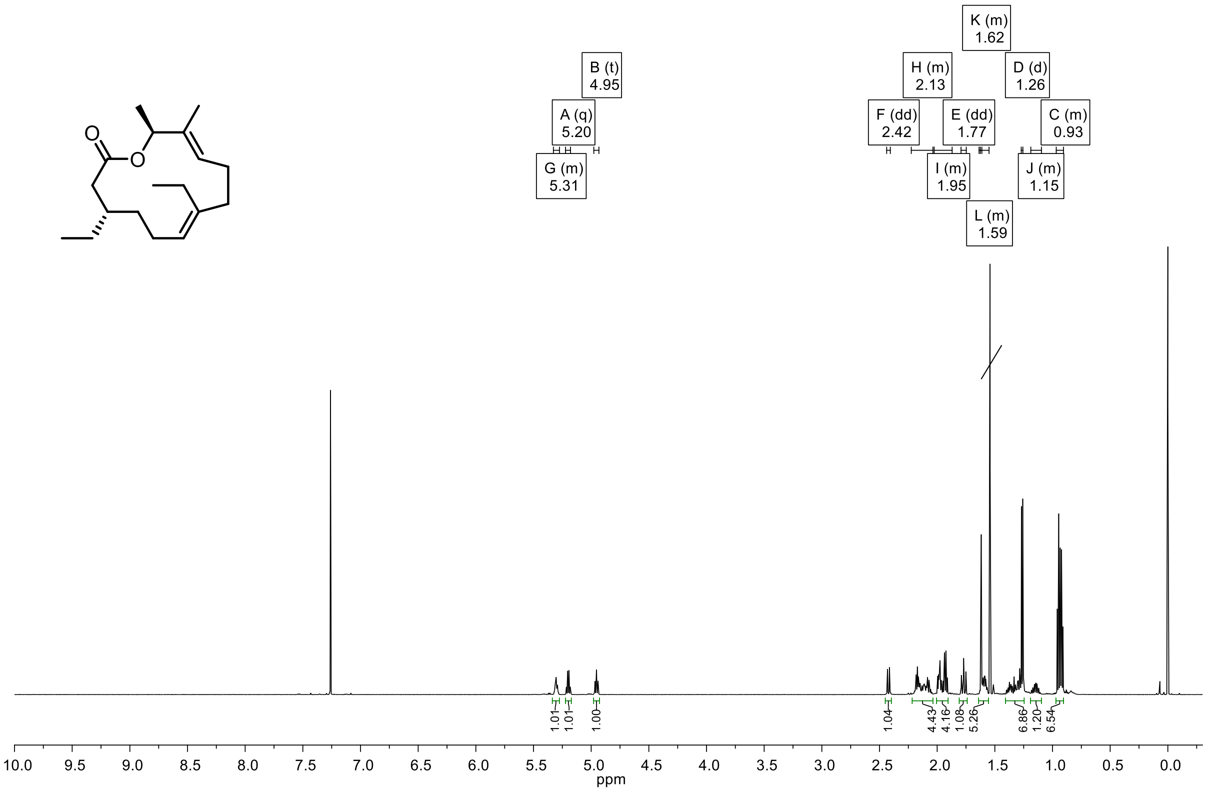

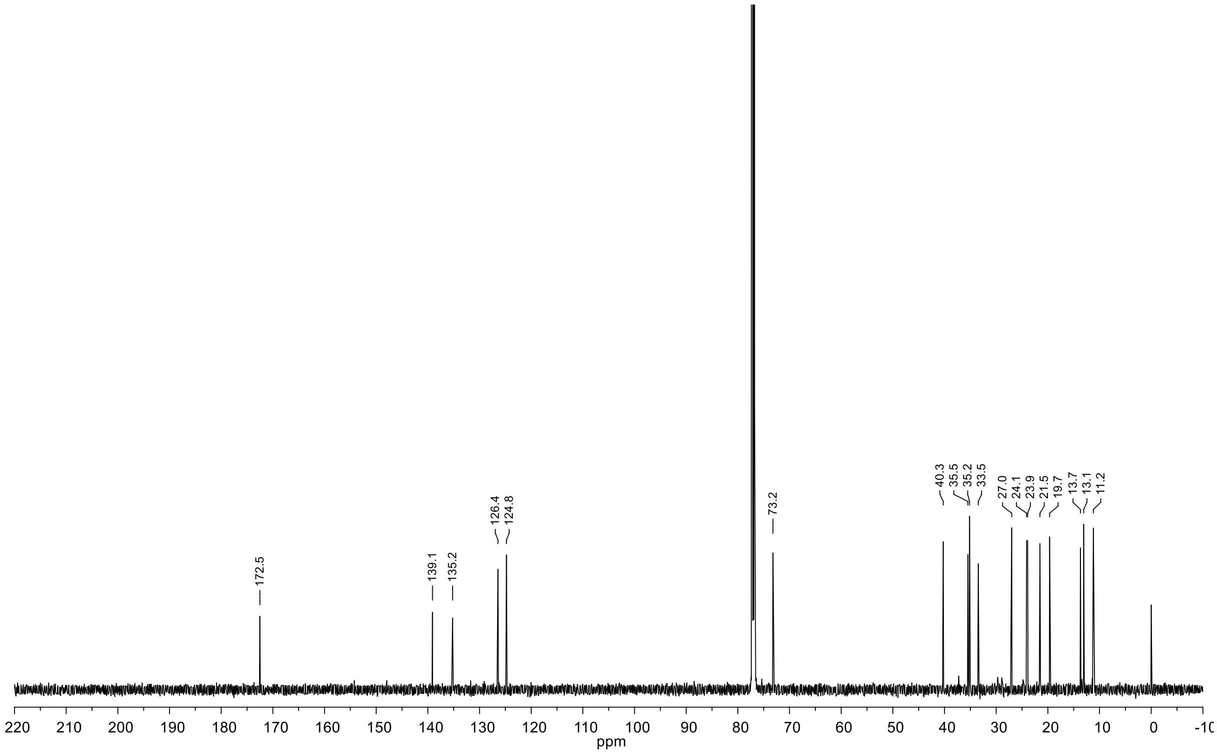

Fig. S41: Top: ^{1}H NMR (600 MHz, $CDCl_3$) and bottom: ^{13}C NMR spectrum (150 MHz, $CDCl_3$) of **9’**.

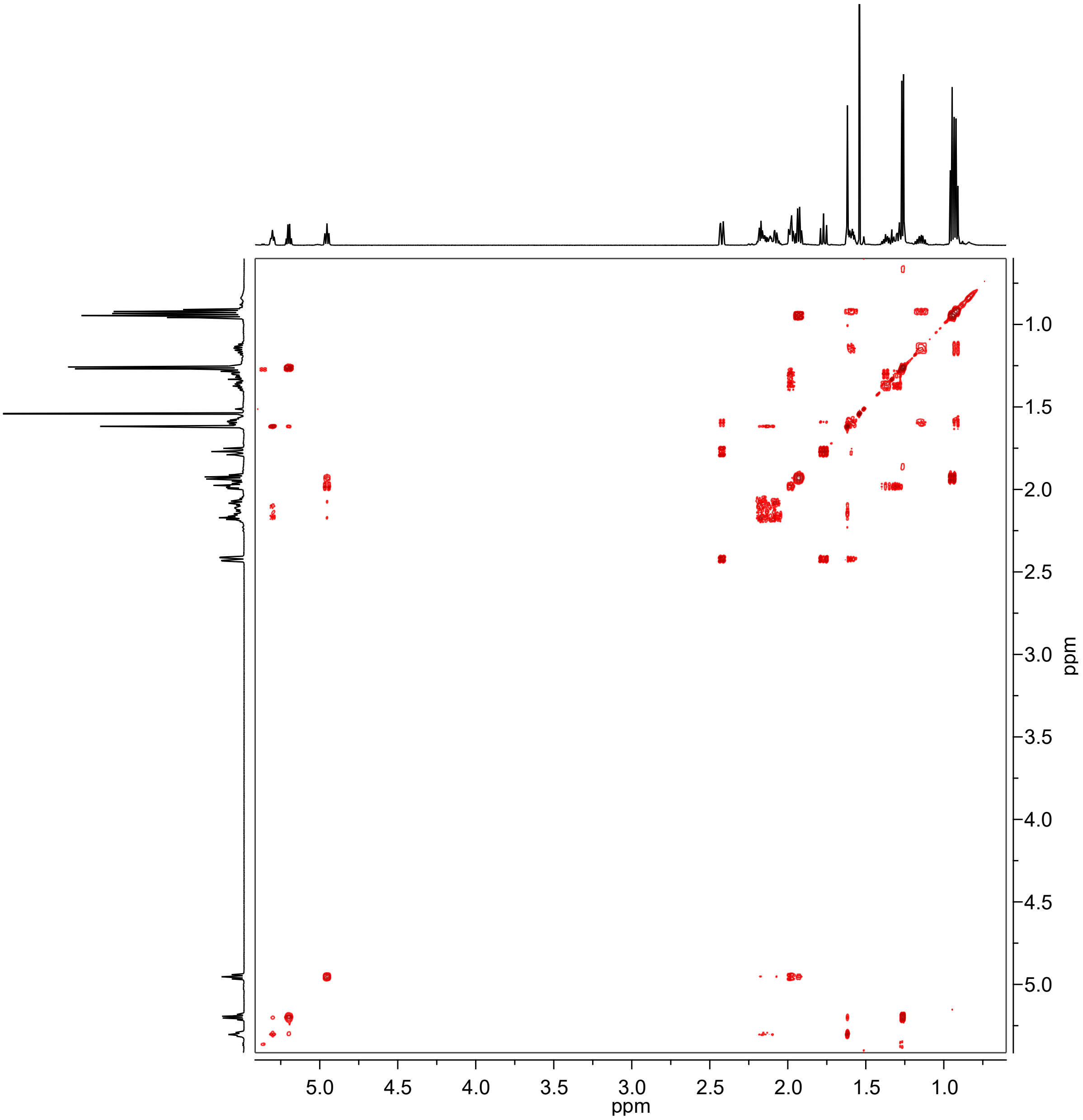

Fig. S42: $^{1}H,^{1}H$ COSY NMR spectrum (600 MHz, $CDCl_3$) of **9'**.

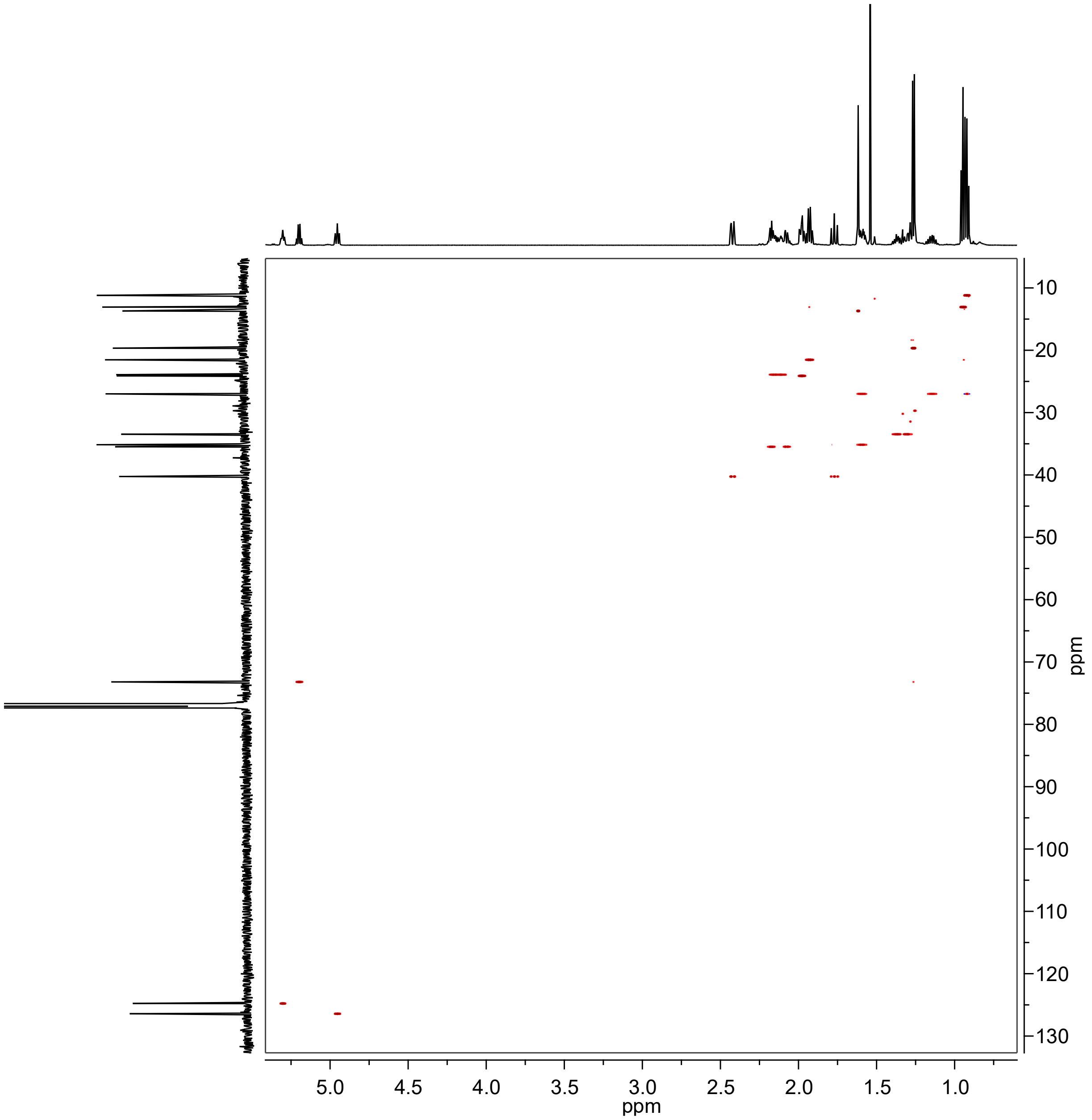

Fig. S43: $^{1}H,^{13}C$ HSQC NMR spectrum (600/150 MHz, $CDCl_3$) of **9’**.

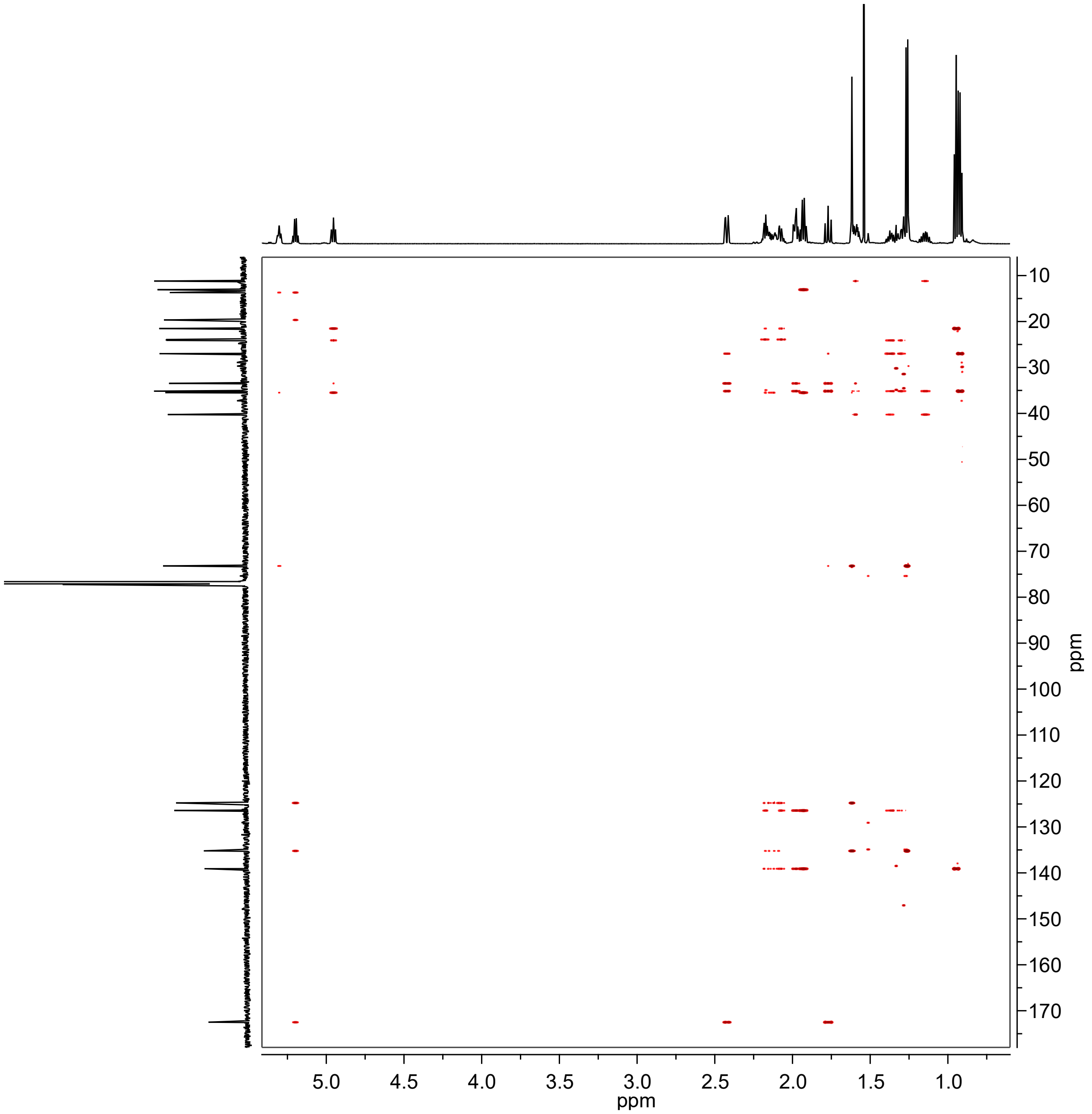

Fig. S44: ^{1}H,^{13}C HMBC NMR spectrum (600/150 MHz, $CDCl_3$) of **9'**.

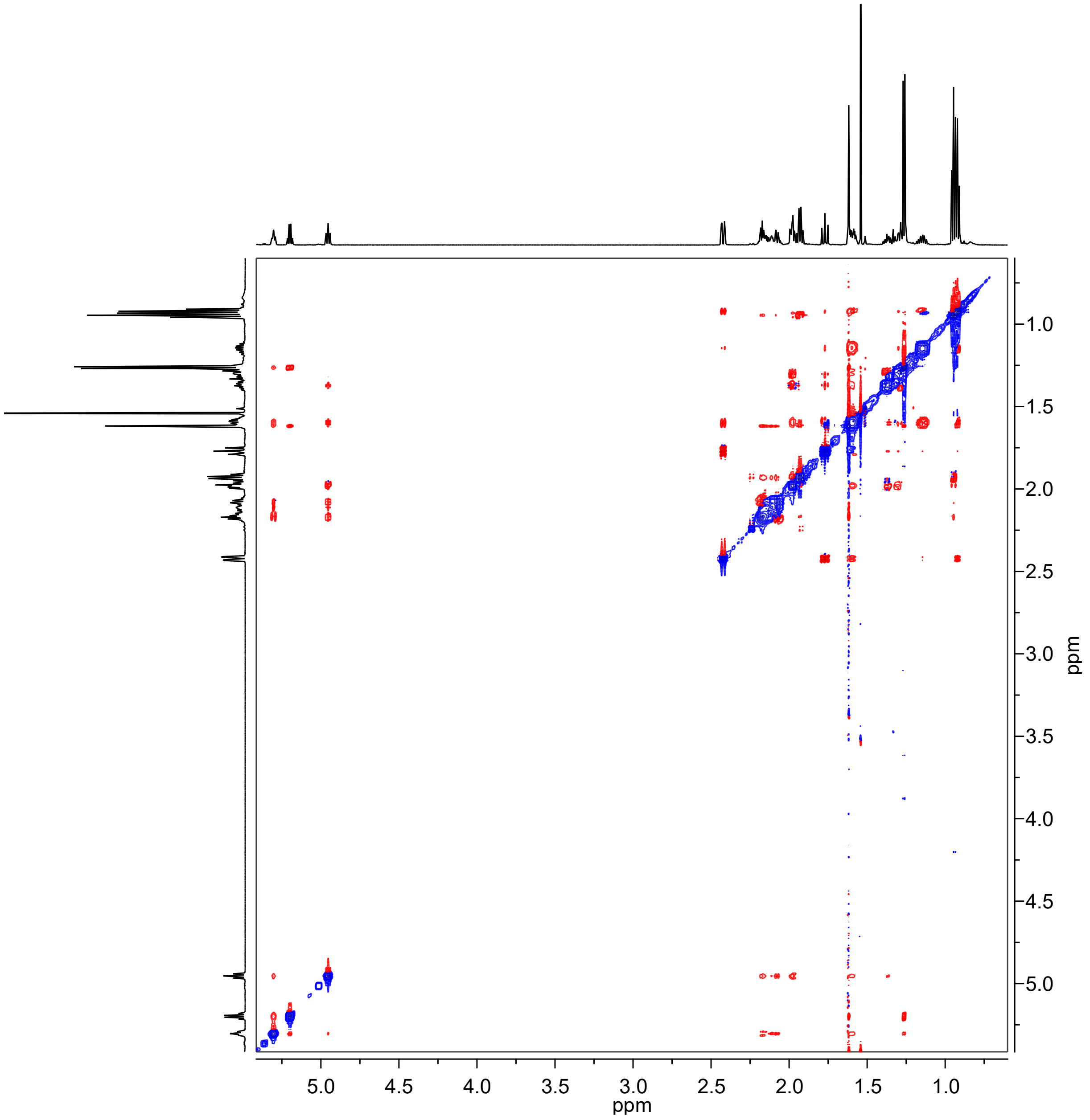

Fig. S45: $^{1}H,^{1}H$ NOESY NMR spectrum (600 MHz, $CDCl_3$) of **9'**.

(3*R*,6*E*,10*E*,12*S*)-3,7,11-Trimethyl-6,10-tridecadien-12-olide (**6**)

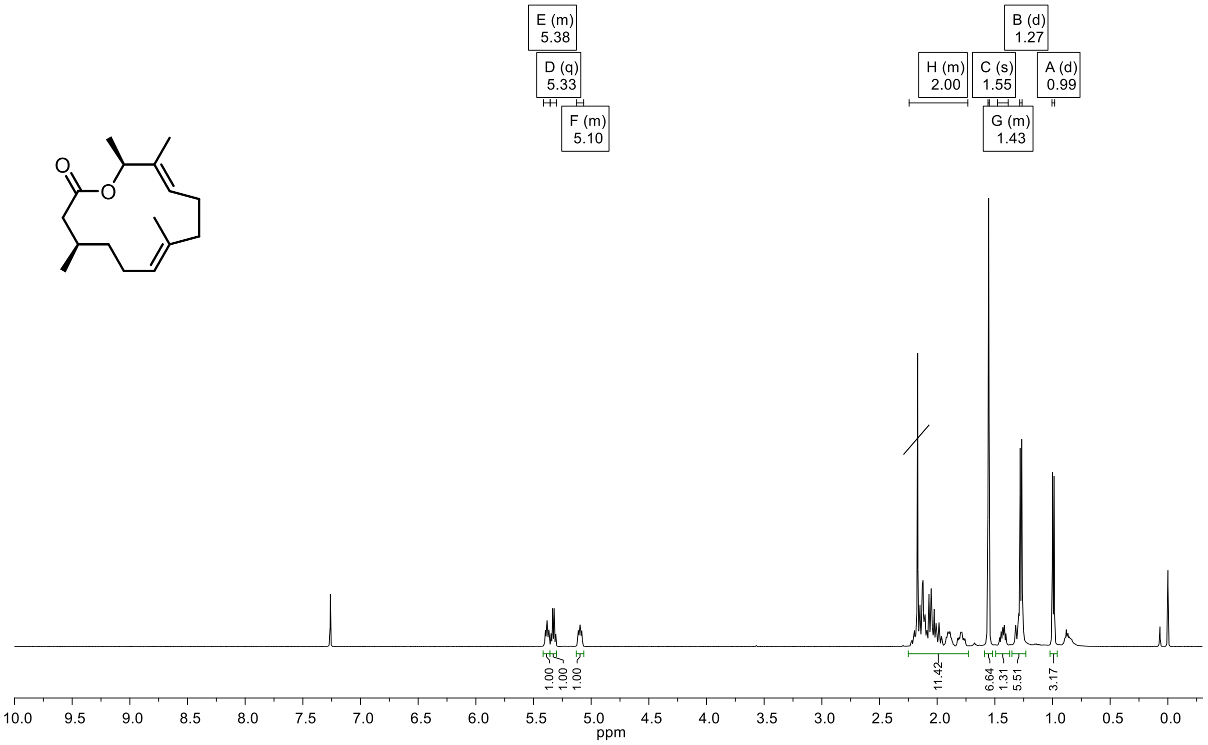

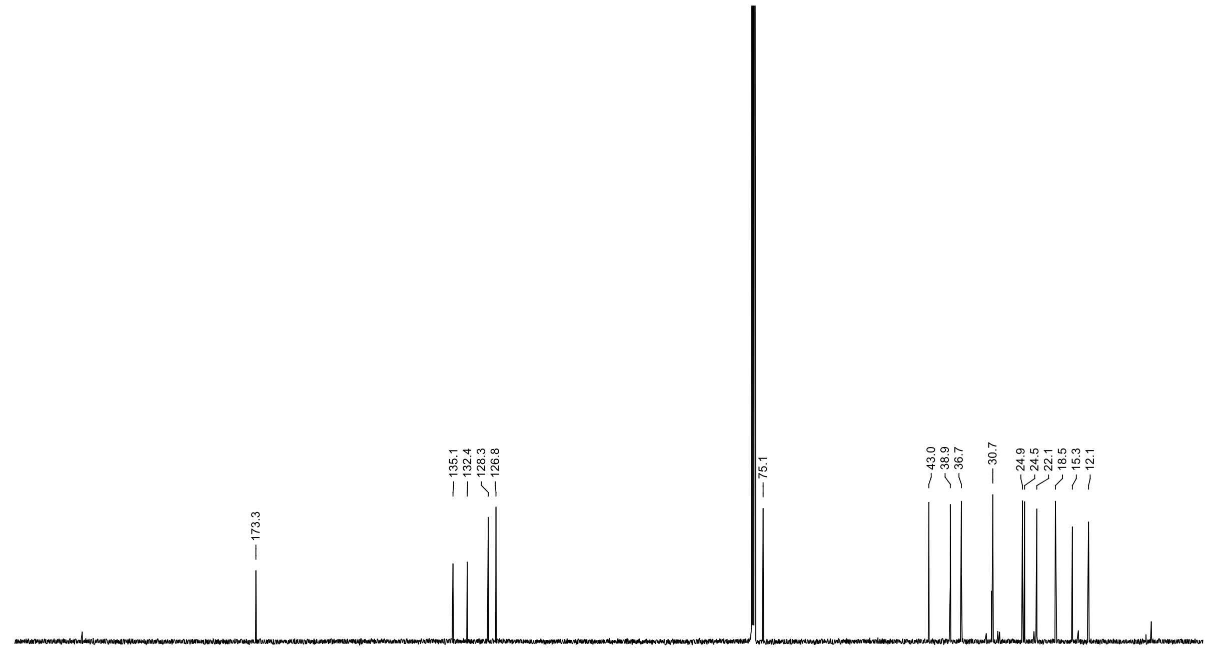

Fig. S46: Top: ^{1}H NMR (400 MHz, $CDCl_3$) and bottom: ^{13}C NMR spectrum (100 MHz, $CDCl_3$) of **6**.

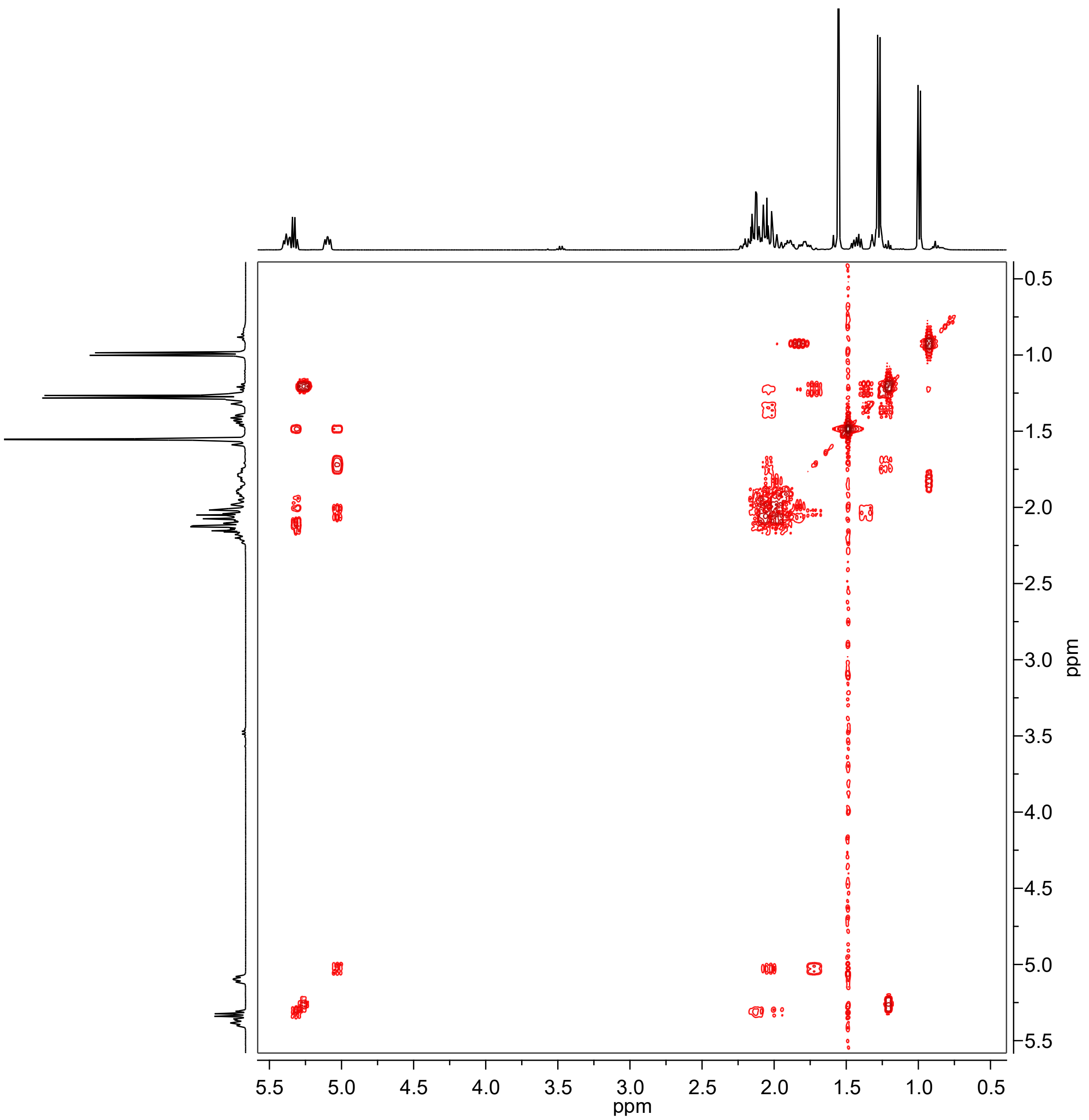

Fig. S47: ^{1}H,^{1}H COSY NMR spectrum (400 MHz, $CDCl_3$) of **6**.

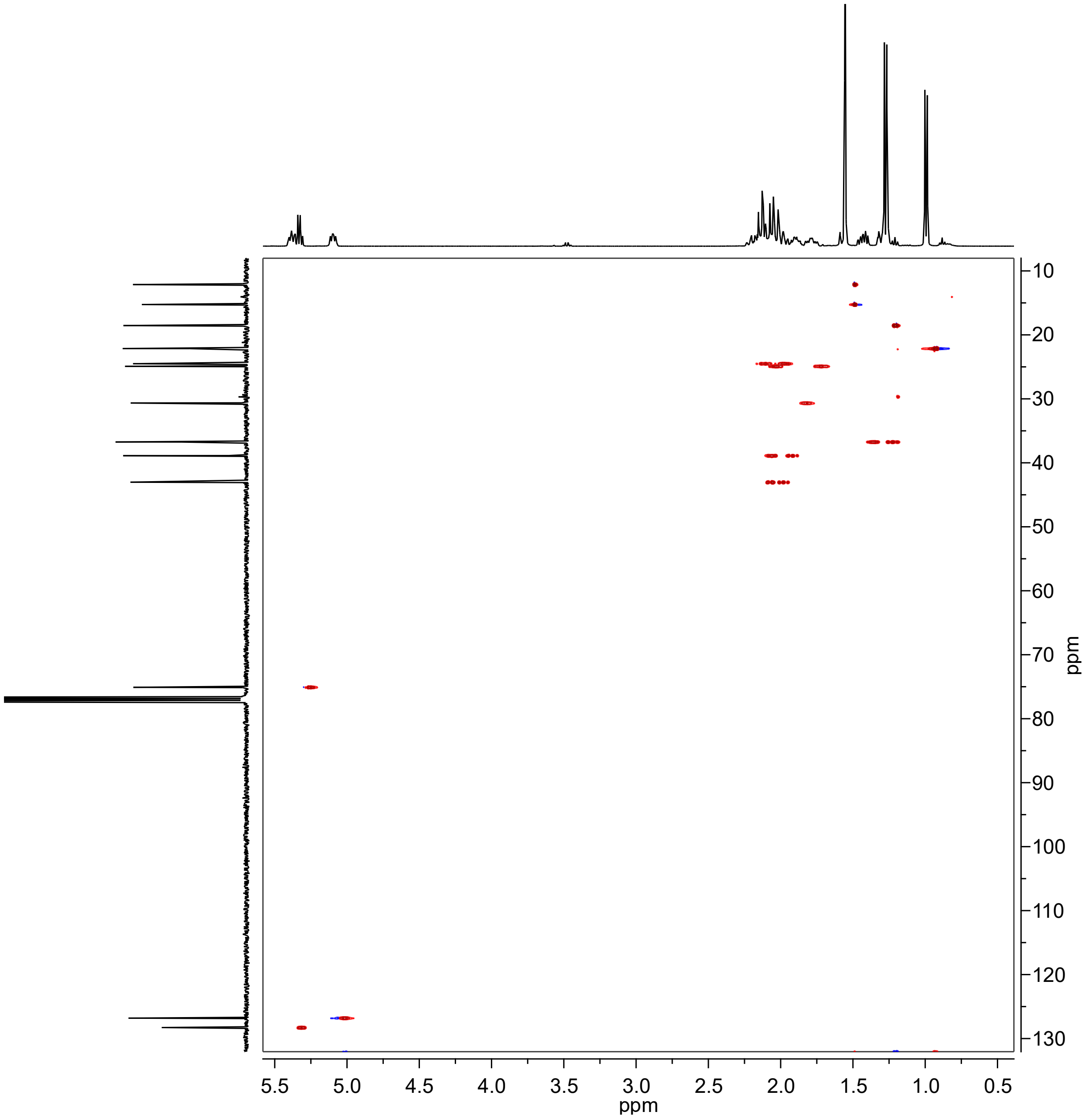

Fig. S48: ^{1}H,^{13}C HSQC NMR spectrum (400/100 MHz, $CDCl_3$) of **6**.

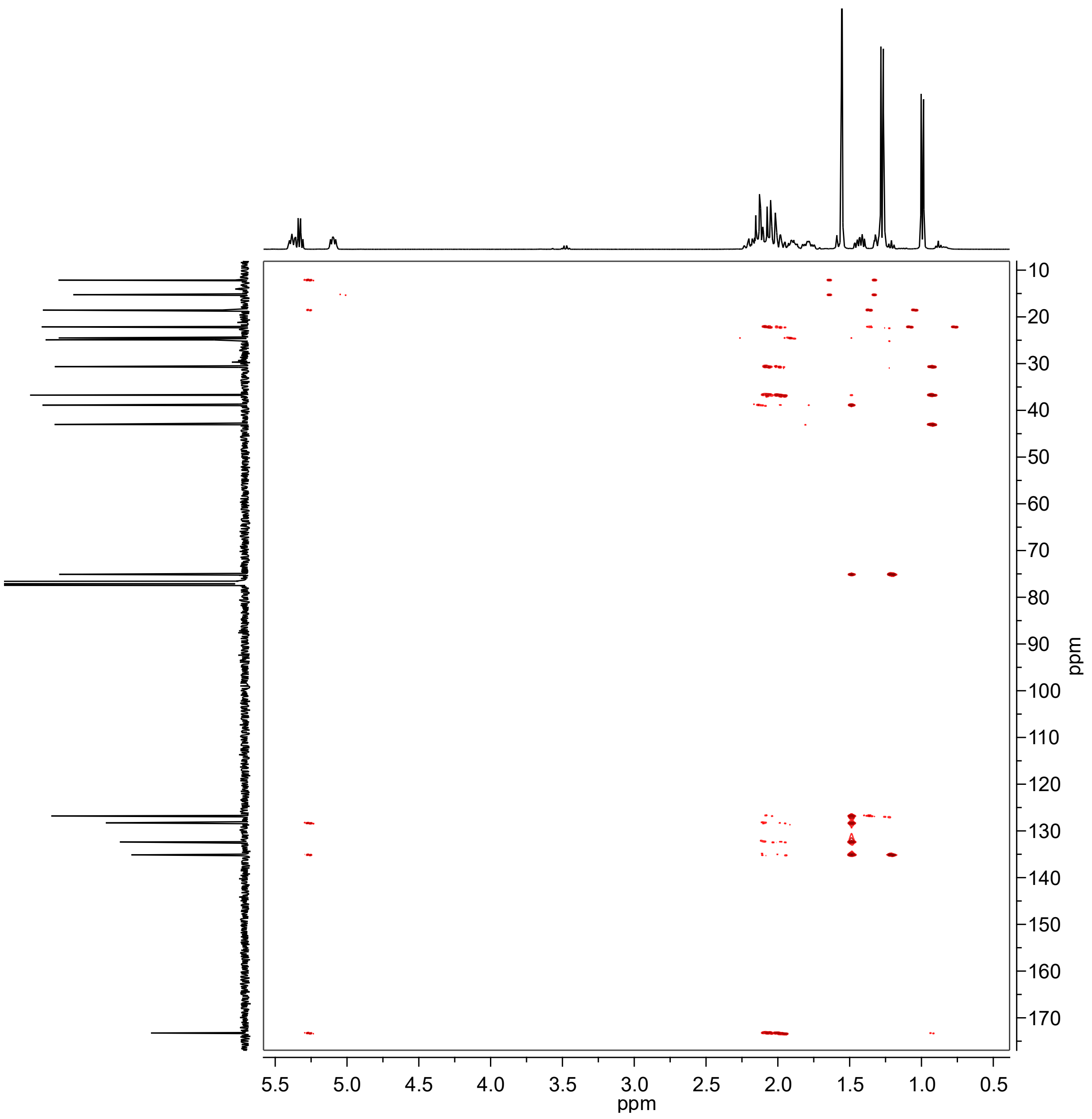

Fig. S49: ^{1}H,^{13}C HMBC NMR spectrum (400/100 MHz, $CDCl_3$) of **6**.

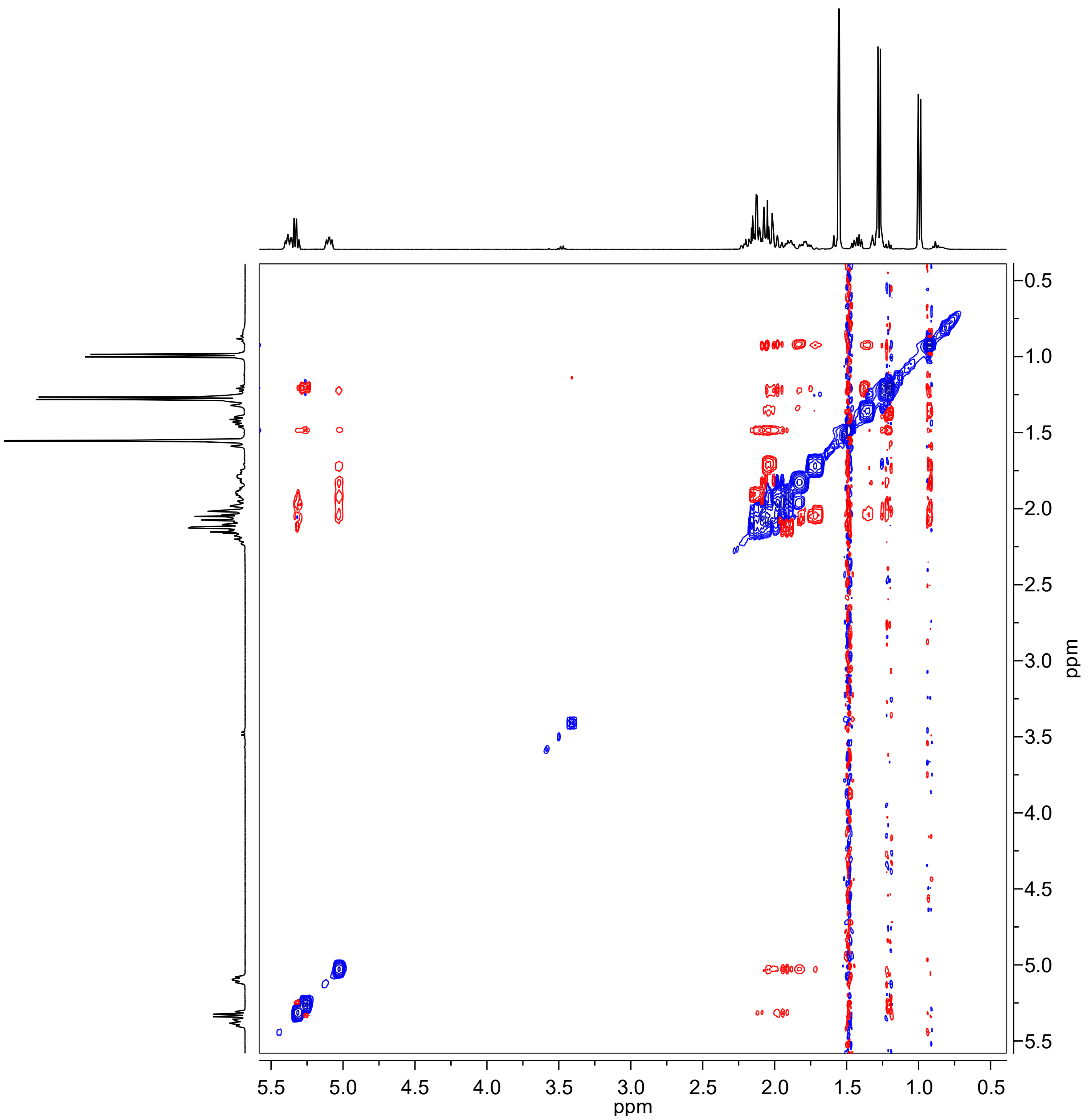

Fig. S50: $^{1}H,^{1}H$ NOESY NMR spectrum (400 MHz, $CDCl_3$) of **6**.

(3*R*,6*E*,10*E*,12*S*)-7-Ethyl-3,11-dimethyl-6,10-tridecadien-12-olide (**7**)

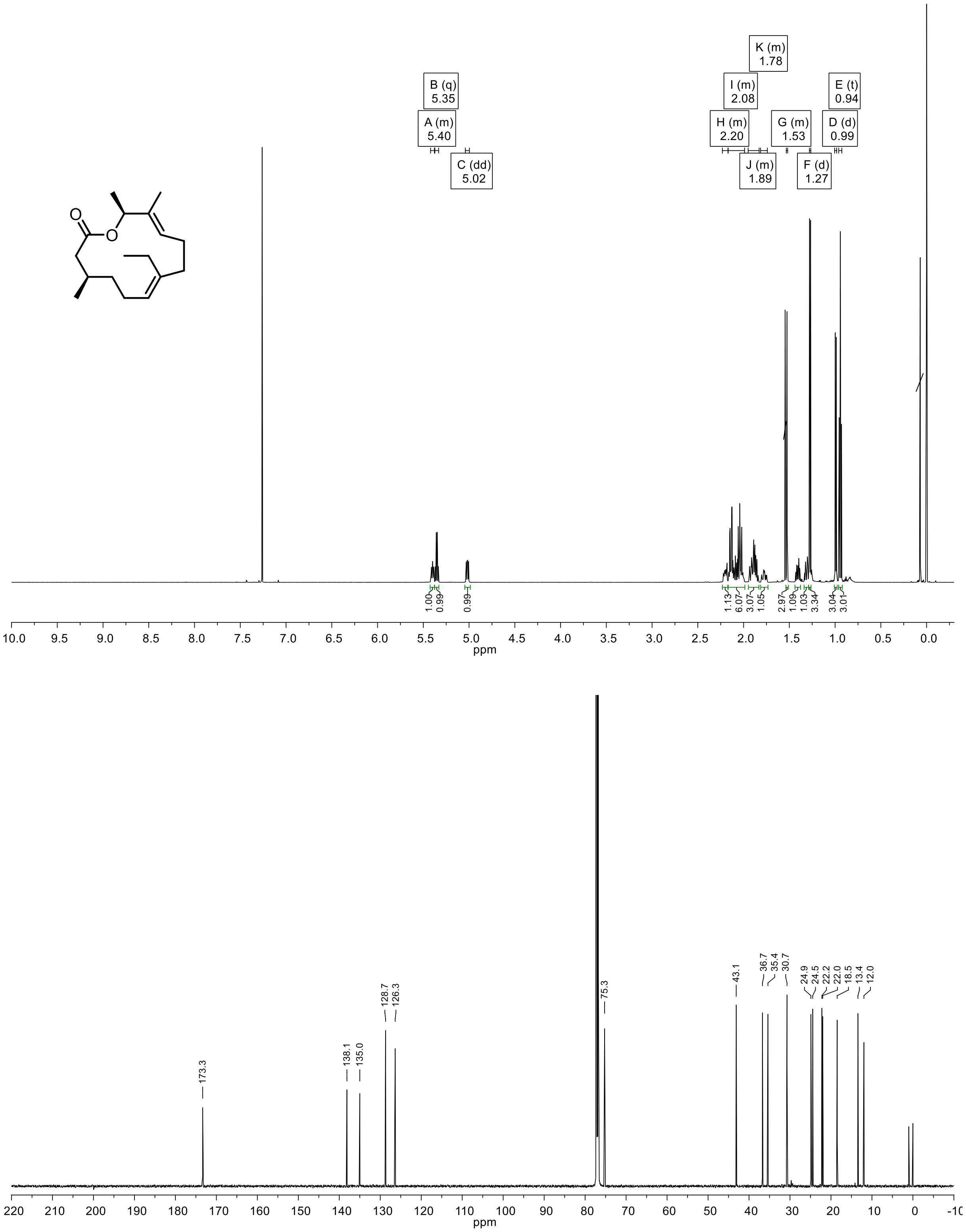

Fig. S51: Top: ^{1}H NMR (600 MHz, $CDCl_3$) and bottom: ^{13}C NMR spectrum (150 MHz, $CDCl_3$) of **7**.

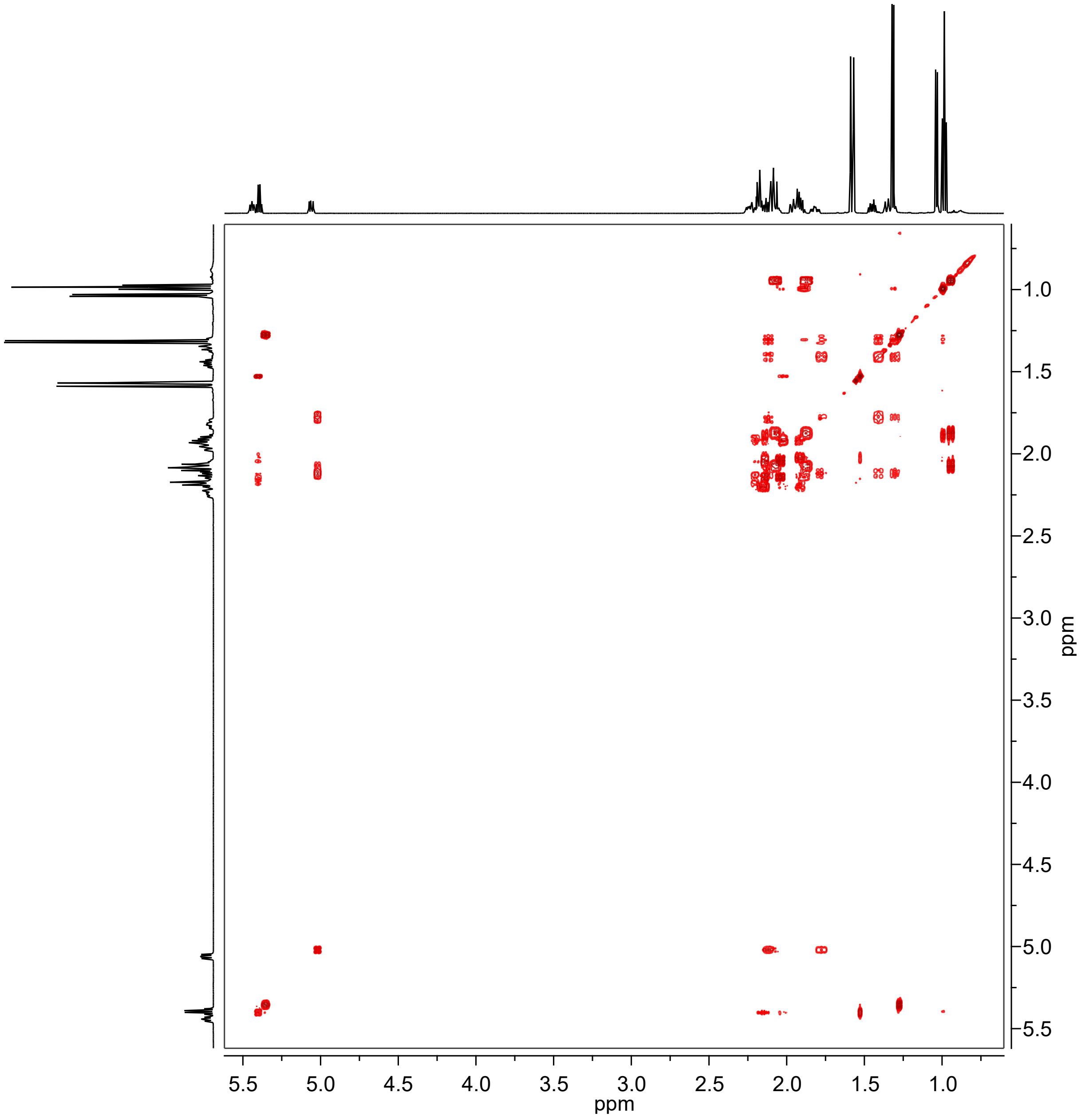

Fig. S52: $^1H,^1H$ COSY NMR spectrum (600 MHz, $CDCl_3$) of **7**.

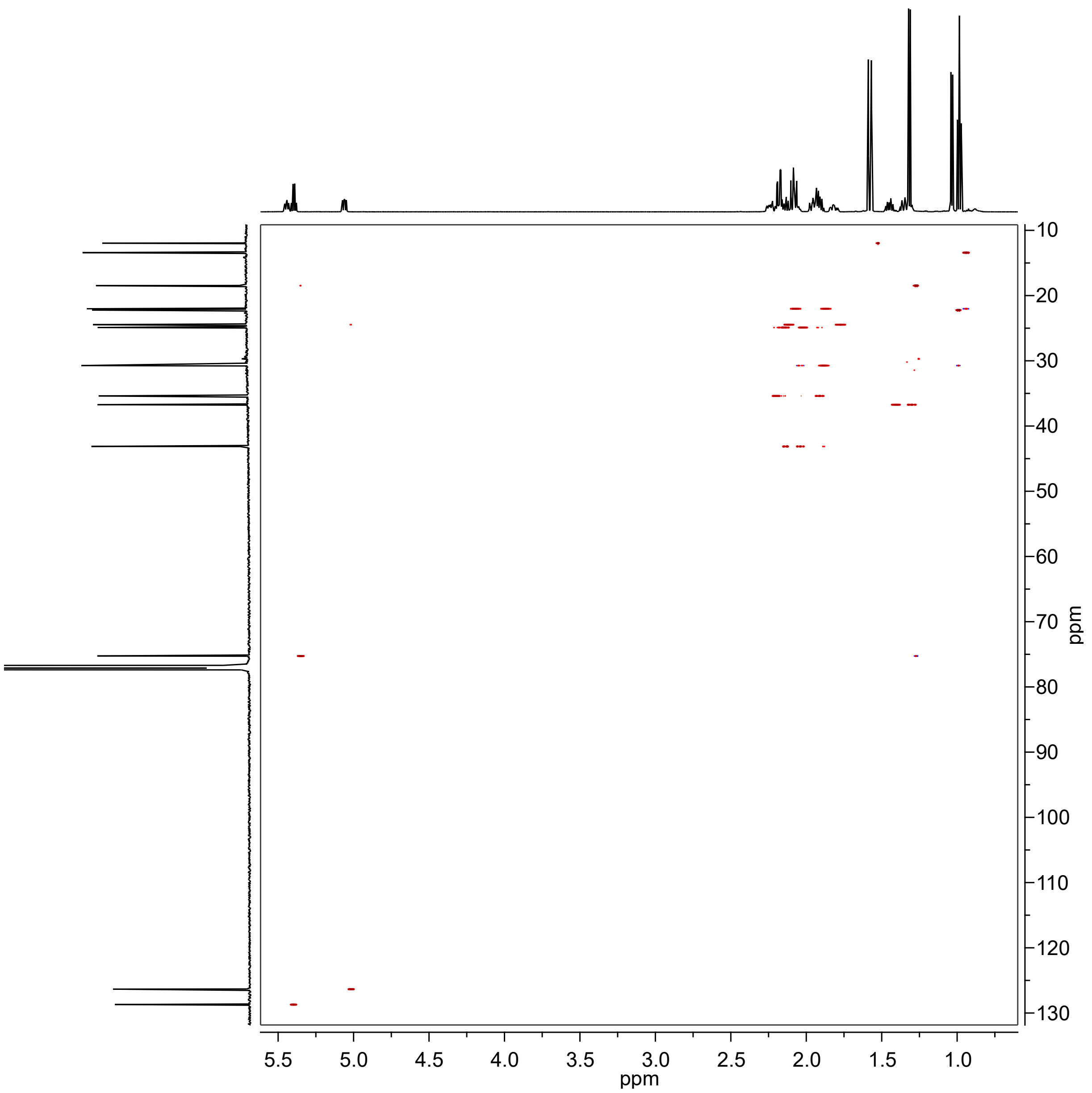

Fig. S53: $^{1}H,^{13}C$ HSQC NMR spectrum (600/150 MHz, $CDCl_3$) of **7**.

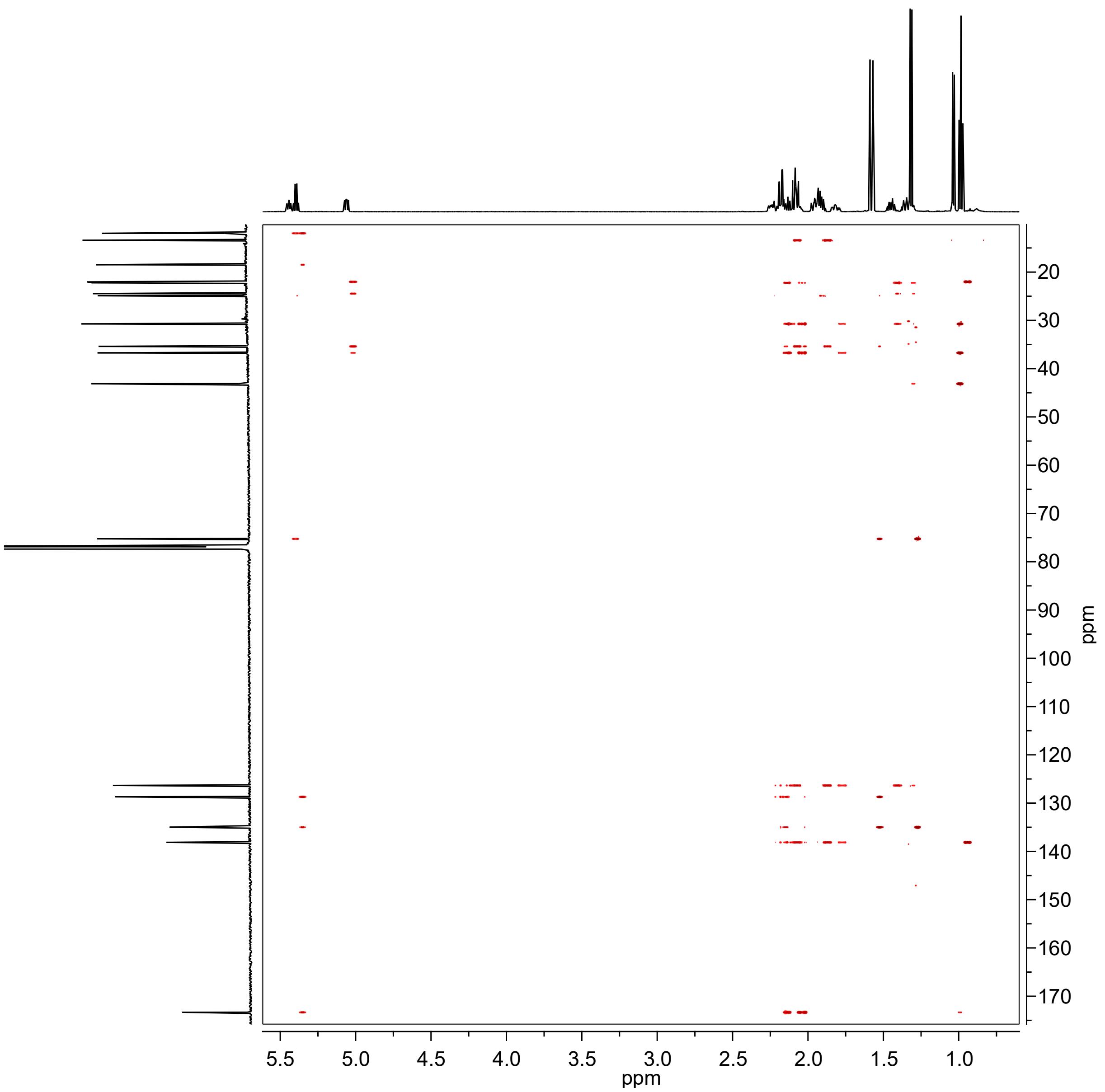

Fig. S54: ^{1}H,^{13}C HMBC NMR spectrum (600/150 MHz, $CDCl_3$) of **7**.

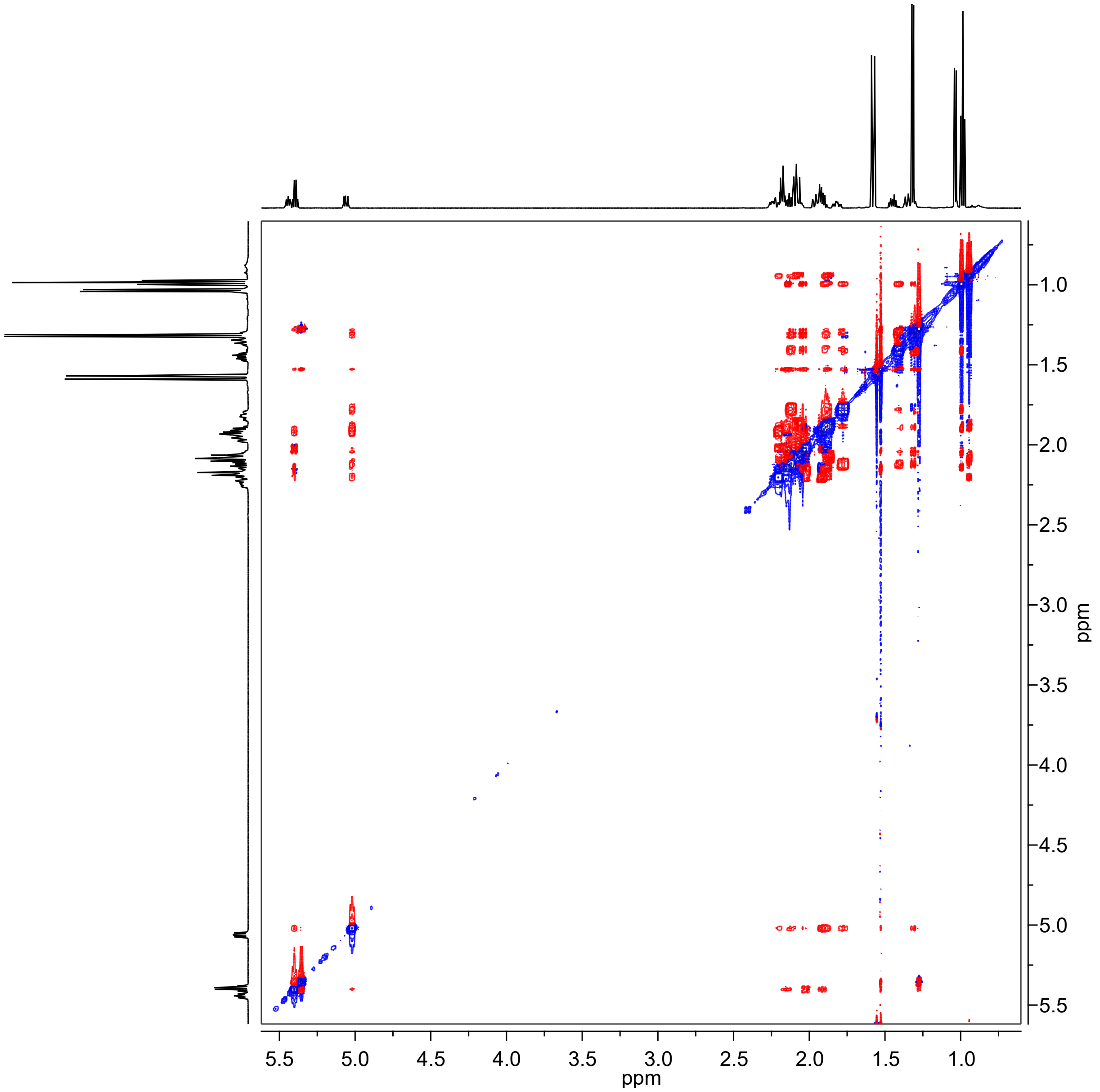

Fig. S55: ^{1}H,^{1}H NOESY NMR spectrum (600 MHz, $CDCl_3$) of **7**.

(3*R*,12*S*)-3-Ethyl-7,11-dimethyl-6,10-tridecadien-12-olide (**8**)

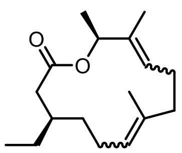

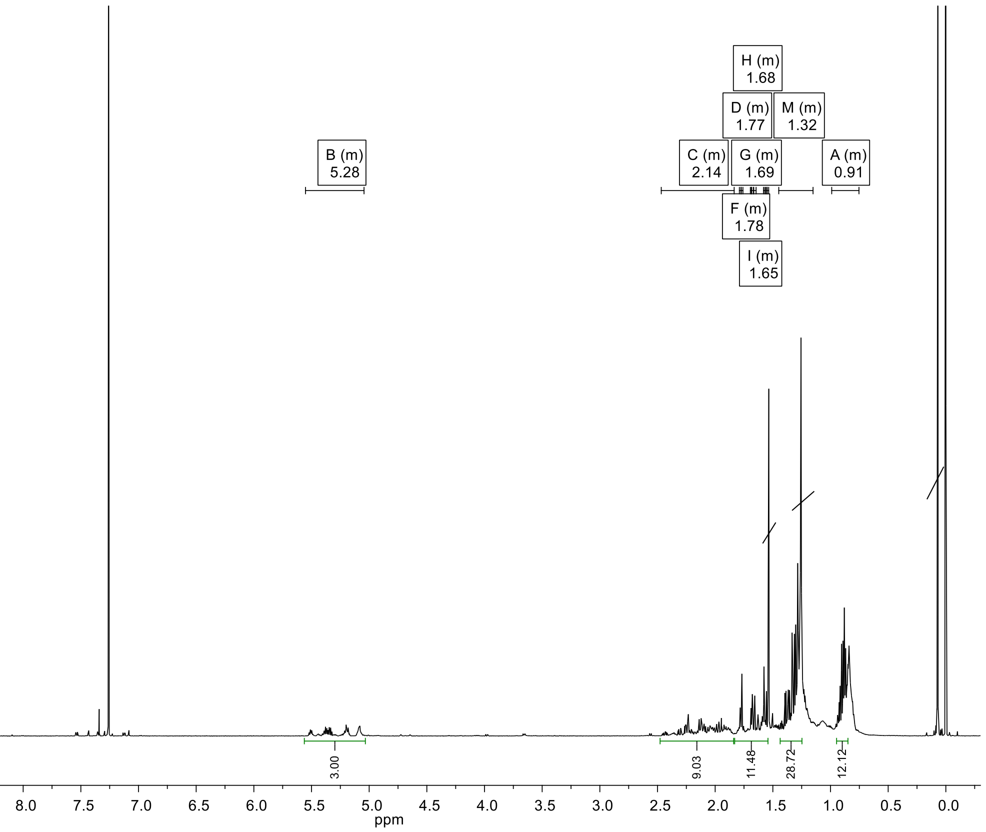

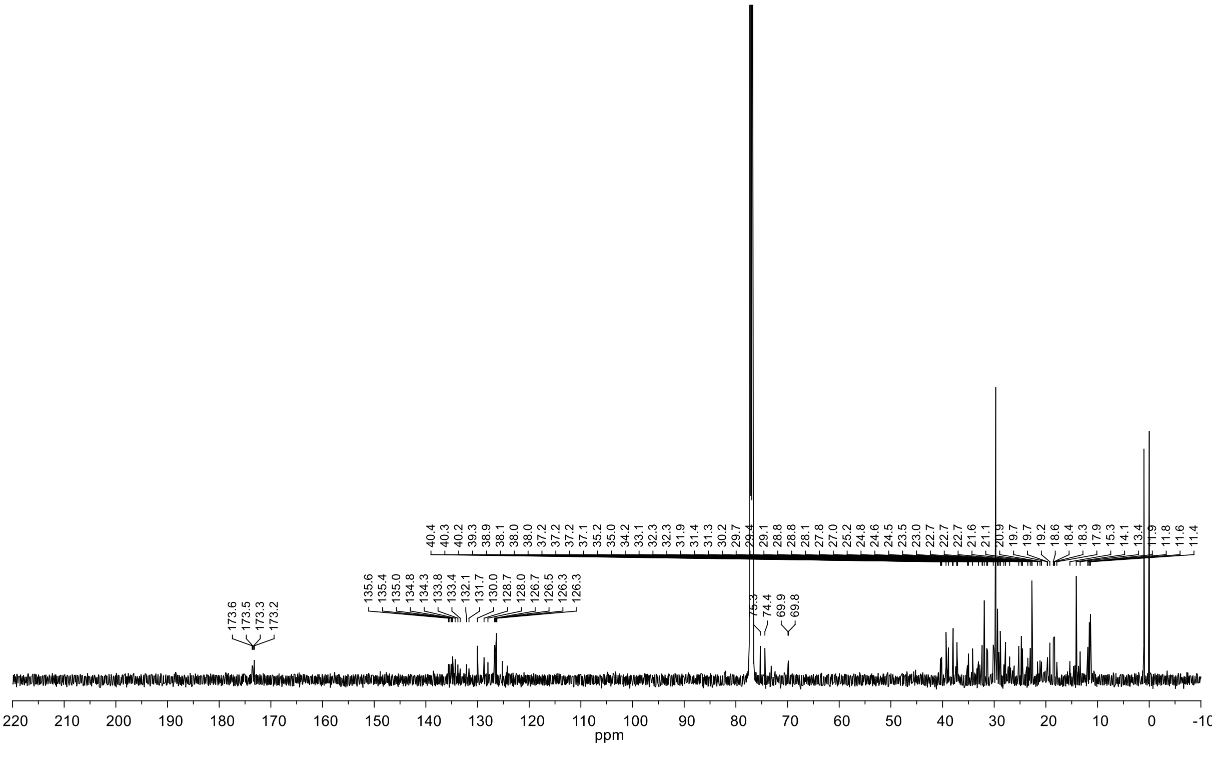

Fig. S56: Top: ^{1}H NMR (600 MHz, $CDCl_3$) and bottom: ^{13}C NMR spectrum (150 MHz, $CDCl_3$) of **8**.
